# Protopeptide backbone affects assembly in aqueous solutions

**DOI:** 10.1073/pnas.2500503122

**Published:** 2025-09-30

**Authors:** Sarah Fisher, Yishi Ezerzer, Rotem Edri, Daniil Akulenko, Eliav Marland, Moran Frenkel-Pinter

**Affiliations:** ^a^Institute of Chemistry, The Hebrew University of Jerusalem, 9190401, Israel; ^b^The Center for Nanoscience and Nanotechnology, The Hebrew University of Jerusalem, 9190401, Israel; ^c^Casali Center of Applied Chemistry, The Hebrew University of Jerusalem, Jerusalem 9190401, Israel

**Keywords:** origins of life, prebiotic chemistry, depsipeptides, chemical evolution, liquid-liquid phase separation

## Abstract

One of the most fascinating mysteries in the field of origins of life concerns the driving force that led to the selection of today’s 20 universal _L_-alpha amino acids in biology. An essential aspect of life’s emergence involves the formation of compartments, which offer encapsulation for target molecules and provide protection from hydrolysis in aqueous environments. Thus, polymers capable of assembly may have had a chemical evolutionary advantage over polymers that lacked this ability. We postulated that primordial peptide assembly could be one of the driving forces that led to the chemical selection of alpha amino acids in life today. To test this hypothesis, we generated depsipeptides, oligomers composed of ester bonds and peptide bonds that form readily under mild drying conditions, as model prebiotic peptides. However, it is unknown whether depsipeptides form assemblies in an aqueous environment similarly to peptides and proteins. To test the hypothesis that depsipeptides with alpha backbones will form assemblies more readily than beta backbones, we synthesized depsipeptides using a matrix of eight alpha- and beta-hydroxy acids and six alpha-, beta-, and gamma-amino acids. The reaction products were analyzed by microscopy and a physical stability analyzer to study assembly formation as well as various analytical techniques for chemical analysis. Our results demonstrate assembly formation in depsipeptide systems containing hydrophobic hydroxy acids and indicate that depsipeptide assemblies containing alpha hydroxy acid backbones are significantly more stable than beta analogs. Overall, our results offer an assembly-driven mode of selection for the alpha backbone in present-day biology.

## Significance

The question of how the protein backbone was selected during early chemical evolution remains one of the most fascinating and puzzling mysteries in origins-of-life research. In this paper, we investigate how the chemical and physical properties of primordial peptide backbones, which contain both peptide and ester bonds, could have influenced the transition from simple molecules to biologically relevant polymers through the formation of compartment-like structures. We demonstrate how the nature of early polymerization and self-assembly may have constrained and guided the emergence of peptides as central components of life. Overall, our results propose an assembly-driven model of selection for the modern protein backbone over alternative analogs, offering insights into the transition from prebiotic chemistry to early biochemistry

Prolonged chemical and geological processes preceded the emergence of life on Earth approximately 4 billion years ago (1–3). These processes involved a gradual increase in the complexity and diversity of organic molecules, eventually leading to today’s biomolecules (4–6). It is likely that small molecules and short heterogeneous oligomers were continuously synthesized and degraded on prebiotic Earth prior to the emergence of the larger and more complex structures of present-day biomolecules (7, 8). All life forms share essential groups of organic molecules, namely proteins, polysaccharides, nucleic acids, and lipids. Proteins are highly evolved linear polymers composed of 20 proteinaceous alpha-_L_-amino acids linked to one another in a covalent amide bond. The enigma of protein evolution is the driving force that led to the selection of the polypeptide backbone and its 20 amino acid building blocks (9–18).

It is well established that early Earth contained a rich variety of amino acid isomers, including alpha-, beta-, and gamma-amino acids (19). These amino acids originated from both exogenous deliveries, as indicated by traces found on meteorites, as well as endogenous synthesis on prebiotic Earth (20–27). However, out of hundreds and possibly thousands of amino acids that were present, only a small subset was selected for biology. Moreover, various amino acids were probably selected at later stages of chemical evolution when a more evolved metabolism enabled the synthesis of more complex amino acids that were not initially available abiotically. The mechanisms underlying the selection of today’s amino acids are one of the biggest questions in the field of origins of life (28). More specifically, the driving forces leading to the selection of alpha amino acids over other isomers remain unknown. Using condensation-dehydration reactions of mixtures of alpha- and beta-amino acids and alpha- and beta- hydroxy acids, it has been demonstrated that chemical reactivity alone cannot explain the selection of alpha amino acids over beta-amino acids (29). Hence, it is likely that other mechanisms that favored the incorporation of alpha amino acids over beta amino acids in biology must have been at work (30, 31).

Peptide bond formation in aqueous media is both thermodynamically and kinetically unfavored and thus does not occur spontaneously (32, 33). To explain abiotic peptide formation, several hypotheses have been suggested, including salt-induced peptide formation (34, 35), hydrothermal vent out-of-equilibrium systems (36, 37), aminonitrile coupling (33), catalysis of amino acid condensation by minerals (38), oxazolone-mediated peptide chain extension (39), and wet-dry cycling in small water ponds (40). An alternative route involves the formation of primordial peptide-like molecules through intrinsic catalysis using small prebiotic molecules (41, 42). Depsipeptides, which are oligomers composed of amide bonds and ester bonds, could have served as protopeptides. Depsipeptides form spontaneously under mild conditions in mixtures of hydroxy acids and amino acids, where preformed ester bonds between hydroxy acids lower the activation energy barrier for peptide bond formation (43–50). Dry-wet cycling leads to enrichment in peptide bonds over ester bonds in the product oligomers (43). This suggests a plausible abiotic pathway by which early protopeptides have gradually transitioned toward amide-rich backbones.

It is well known that polypeptides can assemble into a variety of secondary, tertiary, and quaternary structures, and even short peptides and single amino acids can form membranes and coacervates (51–64). Such structures could serve as compartments and eventually evolve into functioning protocells (65–78). These assemblies increase kinetic stability and attenuate hydrolysis (79). Hence, self-assembly plays a significant role in the synthesis-hydrolysis cycles and confers an advantage for the accumulation of a selected subset of assembled molecular species over others that are incapable of assembly. Brack demonstrated the formation of proteinaceous polypeptides that self-assemble into beta-sheets, reporting that assembled peptides exhibited lower degradation rates compared to nonproteinaceous unstructured polypeptides (31). While self-assembly of polyesters, another model protopeptide system, has been demonstrated (51), it is unknown whether depsipeptide oligomers can self-assemble in aqueous solutions and whether their assembly will be backbone-dependent.

Here, we hypothesized that self-assembly of depsipeptides could have contributed to the selection of the peptide backbone throughout chemical evolution due to differences in assembly stability. To test this hypothesis, we generated depsipeptides using a matrix of eight alpha- and beta-hydroxy acids and six alpha-, beta-, and gamma-amino acids. The reaction products were chemically analyzed using ATR-FTIR, ESI-MS, and HPLC. Depsipeptide formation was observed in all reaction mixtures, with alpha derivatives showing a greater variety and abundance of products than analogous beta derivatives. Assembly formation and stability were investigated using light microscopy and accelerated stability testing (LUMiSizer®), respectively. Our results revealed that several products of drying reactions between amino acids and hydroxy acids form microdroplet assemblies in aqueous solutions. The products that formed assemblies are mostly composed of alpha hydroxy acids with aliphatic or aromatic residues. In contrast, linear hydroxy acids such as glycolic acid or 3-hydroxypropionic acid did not form any assemblies. Analysis using LUMiSizer® revealed that alpha depsipeptide oligomers exhibit greater stability than their beta counterparts. These structural differences between alpha- and beta-primordial peptide product mixtures could act as a selection force and may provide insights into the origins of the selected building blocks of life in current biochemistry.

## Results

**Fig. 1.**
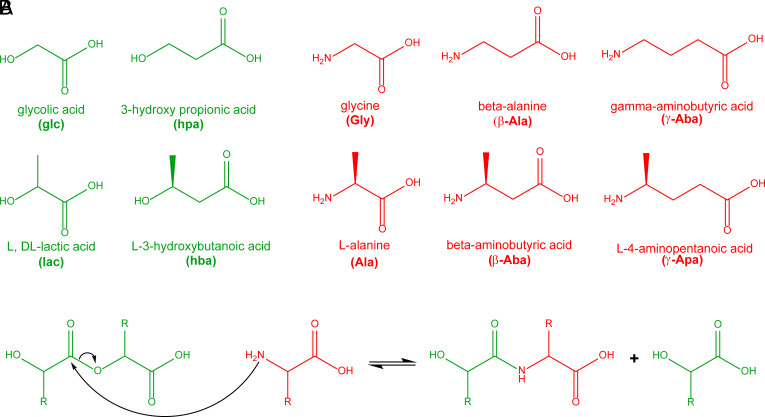
(*A*) Chemical structure of the studied monomers—four hydroxy acids (alpha and beta) are shown in *green,* and six amino acids (alpha, beta, and gamma) are shown in *red*. (*B*) Ester-amide exchange reaction between an ester and an amino acid results in peptide bond formation.

**Fig. 2.**
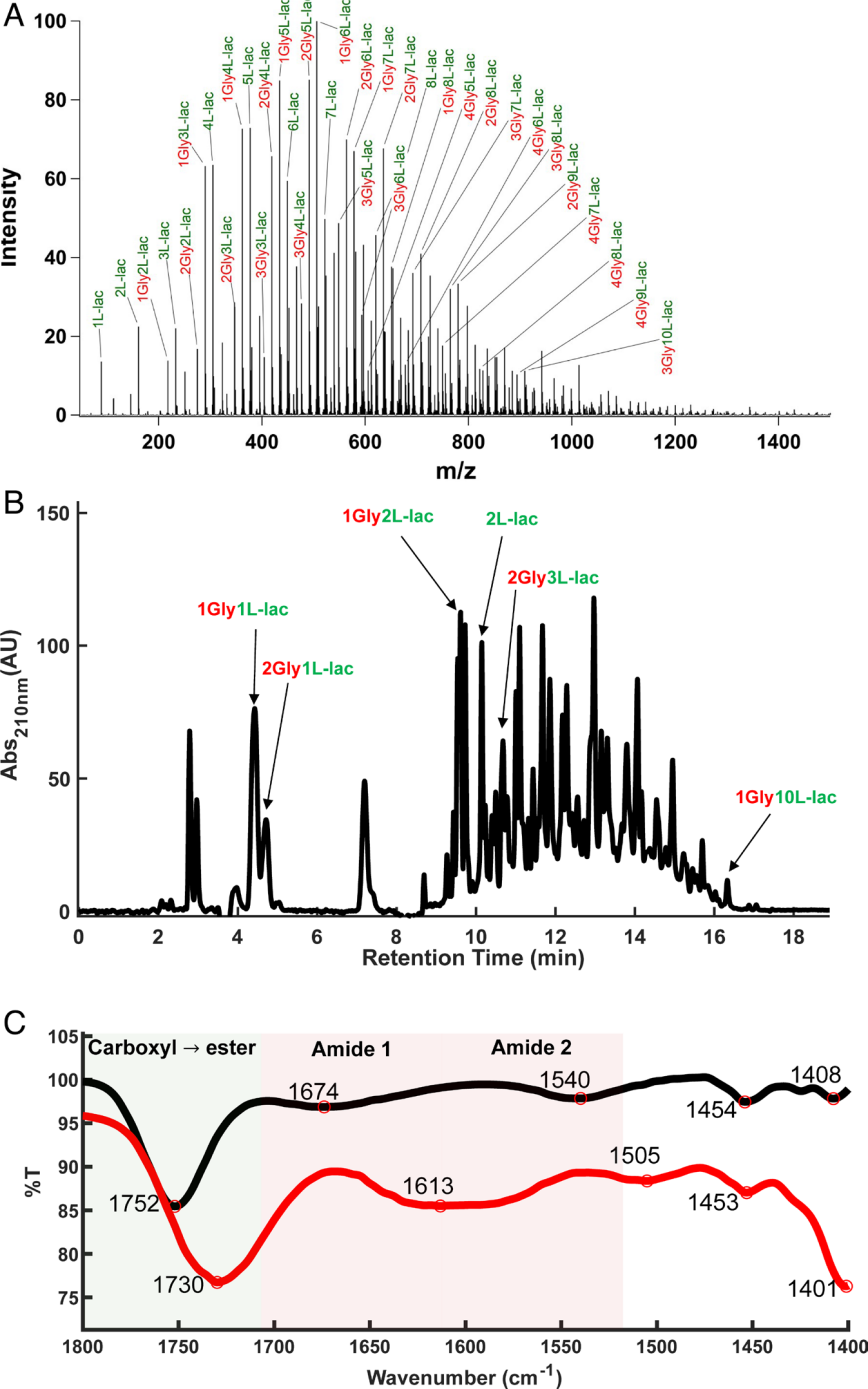
Chemical analysis of products resulting from dry-down of _L_-lac and Gly for 1 wk at 85 °C. The dry products were resuspended in an aqueous solution of 20% acetonitrile in water (v/v) and analyzed by (*A*) ESI-mass spectrometry, (*B*) C18-UV-HPLC, and (*C*) ATR-FTIR. Dried-down sample (black) overlaid with the fresh control monomer mixture (red).

To study depsipeptide assembly in aqueous solutions and the effect of the depsipeptide backbone on assembly, we performed one-step dry-down reactions using various hydroxy and amino acid monomers. Specifically, we characterized depsipeptides with various backbones derived from one-step dry-down reactions of a single hydroxy acid (alpha or beta) with a single amino acid (alpha, beta, or gamma) at a 5:1 molar ratio (hydroxy acid: amino acid) for 1 wk at 85 °C (Fig. 1). In this matrix, a total of five hydroxy acids were used: glycolic acid (glc), _L_-lactic acid (_L_-lac), _DL_-lactic acid (_DL_-lac), 3-hydroxypropionic acid (hpa), and 3-hydroxybutanoic acid (hba). We investigated reactions involving the following six amino acids: glycine (Gly), _L_-alanine (Ala), β-alanine (β-Ala), β-aminobutyric acid (β-Aba), γ-aminobutyric acid (γ-Aba), and 4-aminopentanoic acid (γ-Apa). This matrix of hydroxy and amino acids allowed us to explore how the difference between the depsipeptide monomer backbone modulates oligomerization and assembly. Methylated α- or β-carbons of the amino and hydroxy acids were included to test the effect of an aliphatic side-chain in proximity to the nucleophilic amine (in amino acids) or alcohol (in hydroxy acids) on the assembly propensity of the resulting depsipeptides. Reaction products were dissolved in a 4:1 water:acetonitrile mixture and characterized via a variety of analytical techniques, including high-performance liquid chromatography (HPLC), direct inject ESI-MS, and ATR-FTIR (Fig. 2 and *SI Appendix*, Figs. S1–S90).

The results indicate that all 30 reaction mixtures formed depsipeptides and polyesters (*SI Appendix*, Figs. S1–S90). For example, FTIR analysis of a reaction between _L_-lac and Gly demonstrated the formation of ester bonds (indicated by a shift in the carbonyl region from 1715 cm ^−1^ to 1739 cm^−1^), as well as spectral changes in the amide Ⅰ and amide Ⅱ regions. MS analysis also confirmed the formation of depsipeptides in all reaction mixtures (*SI Appendix*, Figs. S1–S30). Evidently, some reaction mixtures were more reactive than others and gave rise to a greater diversity of products and/ or longer polymers. In general, HPLC and ESI-MS analyses indicated that glc is the most reactive hydroxy acid, and alpha- and beta-amino acids are more reactive than gamma amino acids (*SI Appendix*, Figs. S61–S90). Notably, all samples were only partially soluble in the solvent mixture. Following dry-down, we observed the formation of rigid dry substances in reactions involving alpha hydroxy acids with alpha amino acids, while exchanging alpha monomers with beta monomers in the oligomers reduced this rigidity significantly. We hypothesize that this change is due to differences in hydrogen bonding propensity and the nature of the resulting oligomers. Control reactions that contained hydroxy acids alone resulted in the formation of long polyester oligomers. By contrast, control reactions involving alpha amino acids did not result in any product formation, while beta- and gamma-amino acids produced only a small number of dimers and trimers (*SI Appendix*, Figs. S91–S96).

**Fig. 3.**
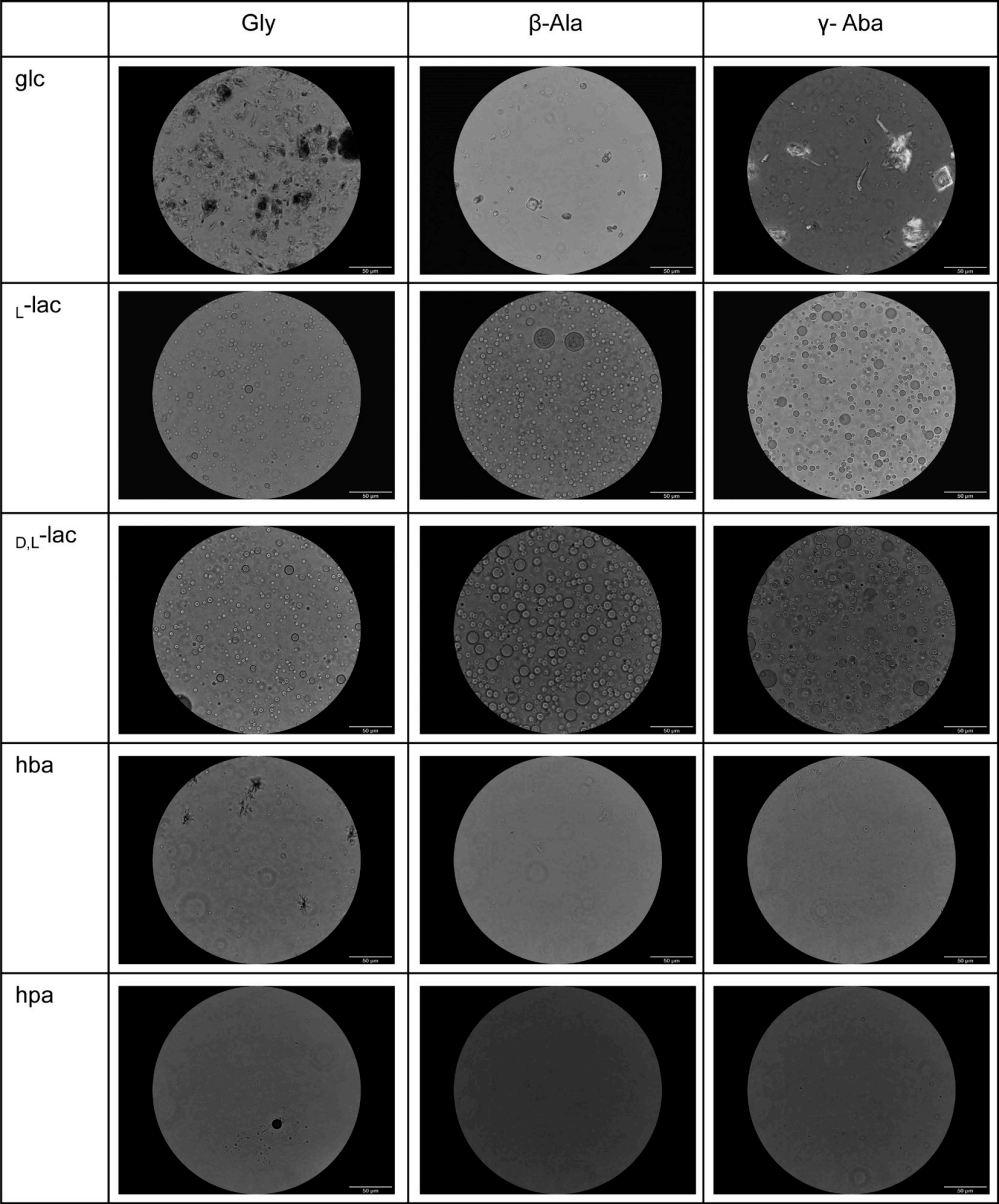
Microscopy imaging of dry-down products of five hydroxy acids: _L_-lactic acid, _DL_-lactic acid, glycolic acid, hba, and hpa, and amino acids in water:acetonitrile (80:20) media. Samples of _L_-lactic acid and _DL_-lactic acid form microdroplet assemblies (Scale bar, 50 μm).

To test whether the resulting reaction products are capable of spontaneous assembly in aqueous solutions, we examined the samples that were reconstituted in an aqueous solution containing 20% acetonitrile and 80% water [similarly to polyester systems (51)] under a light microscope. We observed that all reaction products resulting from dry-down reactions of either _L_-lac or _DL_-lac formed assemblies, regardless of the identity of the amino acid in the mixture (Fig. 3 and *SI Appendix*, Figs. S98–S127). Reaction products that resulted from drying down with the other hydroxy acids (glc, hpa, hba) did not assemble (Fig. 3 and *SI Appendix*, Figs. S98–S127). These results indicate that microdroplet formation requires reaction products that contain alpha hydroxy acids with hydrophobic side chains. Notably, no significant morphological difference was observed between _DL_-lac and _L_-lac assemblies. As expected, fresh control mixtures of lac and amino acids prior to drying did not exhibit any microdroplet formation (*SI Appendix*, Fig. S128).

**Fig. 4.**
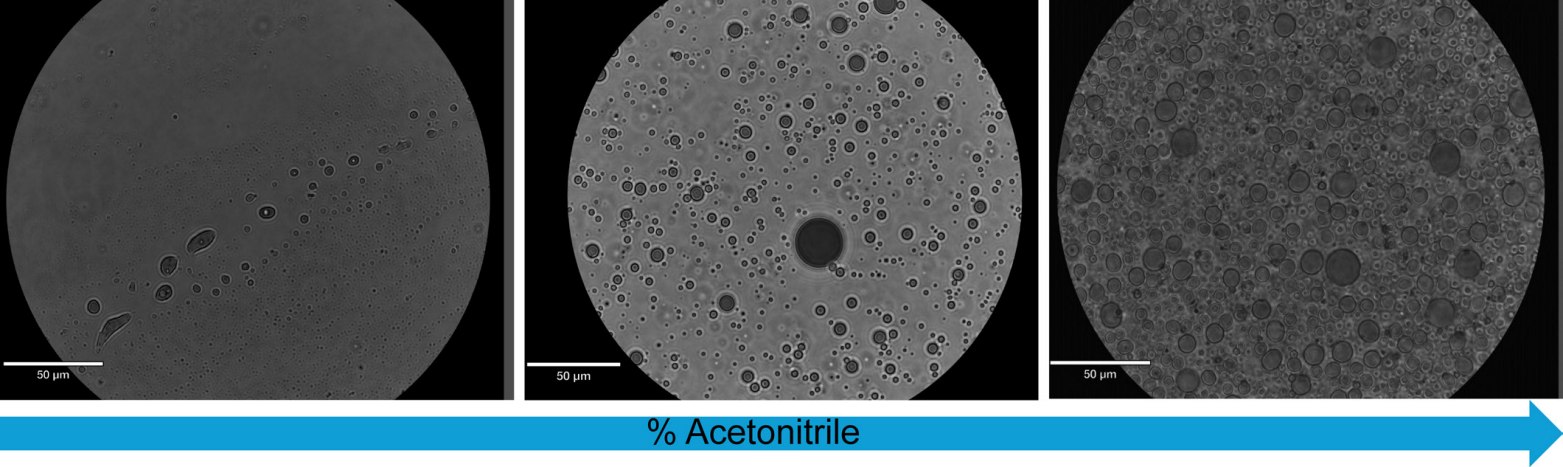
Solvent effect on structure formation of products resulting from dry-down of _L_-lac and Gly for 1 wk at 85 °C as evident in bright-field microscopy imaging. Different ratios of water:acetonitrile solutions were used to resuspend the dry samples.

To study the formation of assemblies further, we proceeded to examine the reaction products of _L_-lac and Gly at varying concentrations (100, 200, and 400 mM Gly, containing 0.5 M, 1 M, and 2 M lac, respectively), aqueous solvent compositions (no acetonitrile, 20% acetonitrile, and 40% acetonitrile) (Fig. 4), and various molar ratios of hydroxy acids and amino acids (5:1, 2:1, and 1:1, in favor of lac) (*SI Appendix*, Figs. S129–S131). We observed that decreasing solvent polarity, by increasing the percentage of acetonitrile, resulted in a higher density of microdroplets. Assembly formation was more prominent in a solvent mixture of 20% acetonitrile and 80% water compared to pure water, while 40% acetonitrile led to a higher density and polydispersity of assemblies (Fig. 4). This effect is likely due to the poor water solubility of the dried product and the interactions between the products and solvent. Increasing the overall amino acid/hydroxy acid concentration or the molar ratios between hydroxy acid and amino acid led to a higher density of assemblies. We also found that microdroplet assemblies are visible even after incubation at room temperature for 7 wk as well as after freezing and thawing (*SI Appendix*, Fig. S132). To explore the concentration limits of assembly formation, we examined assemblies of _L_-lac and Gly based depsipeptides at varying concentrations, ranging from 10 mM to 100 mM in 10 mM increments. As expected, the number of observable assemblies decreased at lower concentrations (*SI Appendix*, Fig. S133). Additionally, we investigated the effect of the drying temperature on assembly formation. To that end, we incubated mixtures of _L_ -lac and Gly at five different temperatures: 95, 85, 75, 65, and 55 °C for 1 wk under drying conditions. After 1 wk of drying, we resuspended the products in an aqueous solution of 20% acetonitrile in water (v/v) and tested assembly formation under the microscope. We found that assemblies formed at temperatures of 75 °C and above (*SI Appendix*, Fig. S134).

To examine the importance of ester bonds within product mixtures for microdroplet formation and their kinetic stability, we performed a hydrolysis reaction under conditions in which ester bonds preferentially hydrolyze over amide bonds (80). To that end, we rehydrated a dry sample of _L_-lac and Gly in water and incubated it for 1 wk at 85 °C with a closed cap and a layer of silicon oil on top to prevent evaporation of water. MS spectra confirmed complete ester hydrolysis (*SI Appendix*, Figs. S135 and S136). Following hydrolysis, no assemblies were found under light microscopy imaging, indicating the importance of ester bonds in microdroplet formation.

**Fig. 5.**
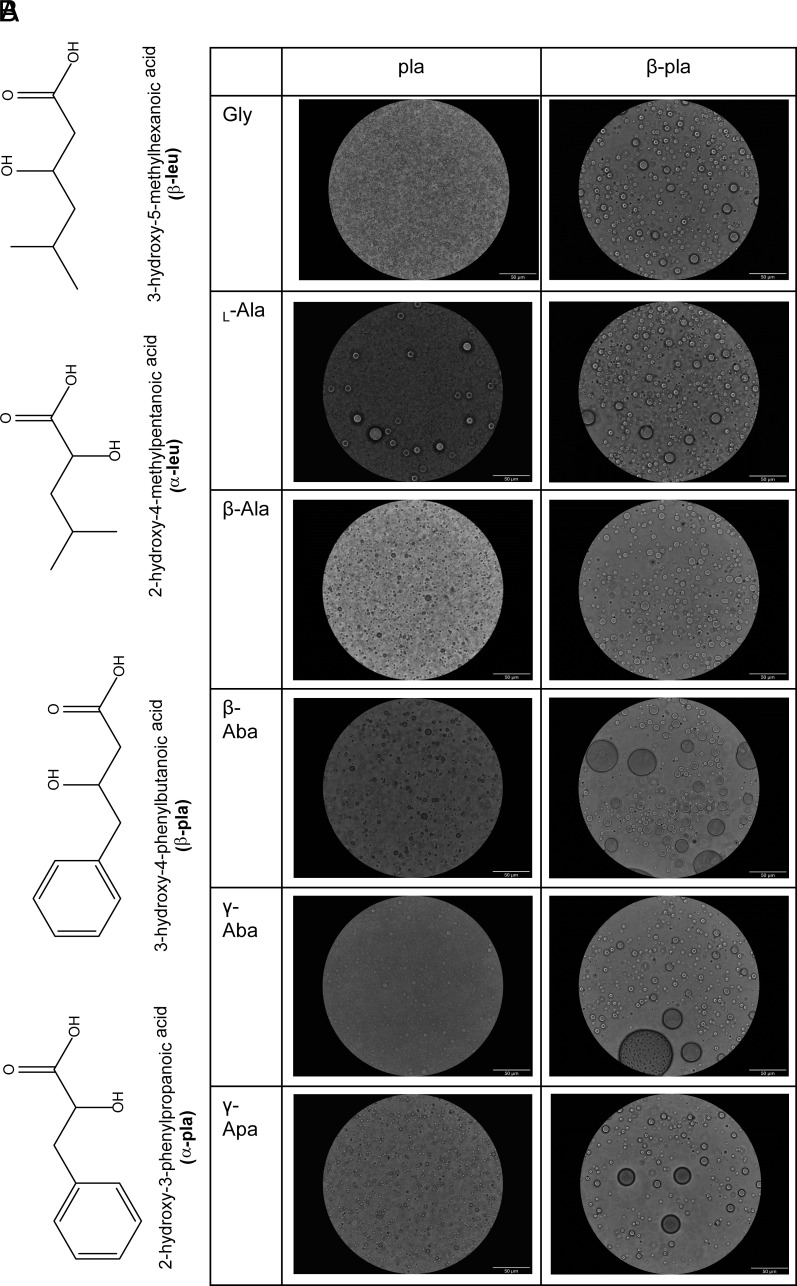
(*A*) Chemical structure of additional four hydroxy acids (alpha and beta) used in this study. (*B*) Microscopy imaging of dry-down products of α-pla and β-pla and six amino acids in water:acetonitrile (80:20) media. All samples form microdroplet assemblies. (Scale bar, 50 μm.)

To explore further the effect of the backbone and side-chain hydrophobicity on microdroplet formation, we carried out reactions with four additional hydrophobic hydroxy acids, two of which were alpha and two of which were beta: α-phenyllactic acid (α-pla), α-leucic acid (α-leu), and their beta derivatives (β-pla and β-leu, respectively) (Fig. 5*A*). The four additional hydroxy acids were reacted with the same six amino acids from the first screen (Fig. 1) at a 5:1 molar ratio (hydroxy acid: amino acid) for 1 wk at 85 °C. Having observed that chirality does not significantly affect microdroplet formation in the case of lactic acid, as both _L_-lac and _DL_- lac formed similar assemblies, we performed reactions for the additional hydroxy acids using racemic mixtures of these hydroxy acids. Products appeared rigid in alpha-monomer-containing depsipeptides, with apparent rigidity decreasing as alpha monomers were replaced by beta monomers, similarly to the results from our previous matrix. Analysis by HPLC, ESI-MS, and FTIR exhibited depsipeptide formation in all samples (*SI Appendix*, Figs. S137–S208). Polyester formation was detected in the dry control samples (*SI Appendix*, Figs. S209–S211).

We observed assembly formation in all samples from the hydrophobic depsipeptide matrix, with some changes in either density or polydispersity of assemblies formed between alpha-, beta-, and gamma-derivative depsipeptides (Fig. 5*B* and *SI Appendix*, Figs. S212–S236). In general, all polyester controls exhibited robust assembly formation and tended to be more turbid than their corresponding depsipeptide samples. Fresh control samples of alpha hydroxy acid monomers in the presence or absence of Gly prior to drying did not exhibit microdroplet formation, while a few undefined small features were observed for the beta analogs. These findings suggest that the side-chain hydrophobicity (branched or aromatic) significantly promotes assembly. While all samples from the hydrophobic depsipeptide matrix formed assemblies, we observed that during the reconstitution of the samples, assemblies derived from beta hydroxy acids underwent phase-separation much faster than their alpha counterparts. This behavior led us to hypothesize that there are significant physical stability differences between the assemblies of alpha- and beta-hydroxy acid backbones.

**Fig. 6.**
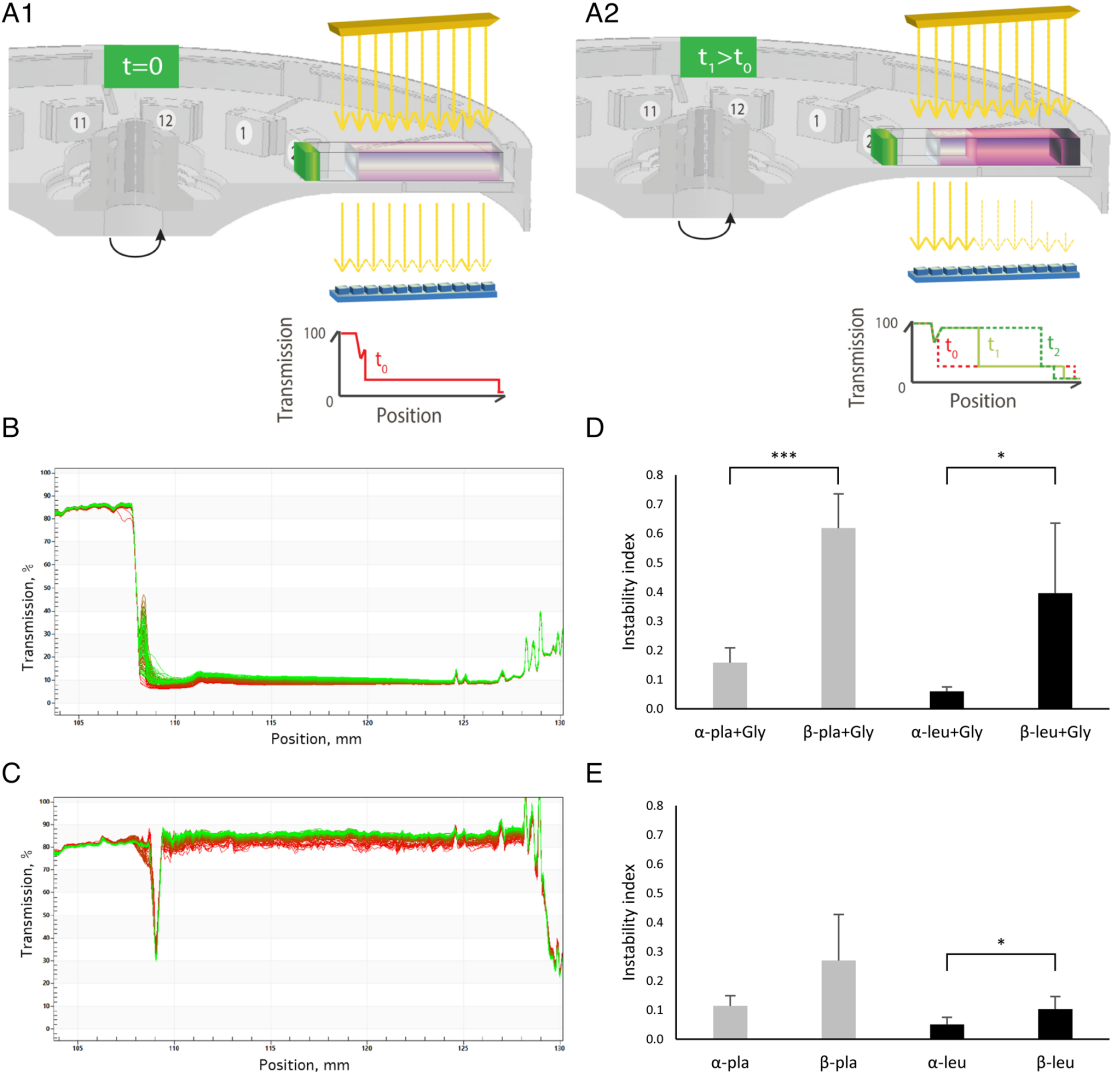
(*A*) An illustration of a multisample analytical centrifuge LUMiSizer® using the STEP™-Technology (Space- and Time-resolved Extinction Profiles) facilitates measurement of the intensity of the transmitted light as a function of time and position over the entire sample length simultaneously. Illustration provided by L.U.M. GmbH (Berlin, Germany). (A1) Typical sample at t = 0. (A2) Typical sample at t1 > t0. (*B*) Transmission profile of α-leu + Gly reaction products. (*C*) Transmission profile of β-leu + Gly reaction products. (*D* and *E*) Instability index profiles of some depsipeptide systems (*D*), and their corresponding polyester systems (*E*). The values provided in the figure are the mean values of 4 to 7 replicate analyses with SD. *P*-value was calculated via *t*-test analysis (* indicates *P* < 0.05, ***P < 0.001).

In order to examine the physical stability of the assemblies formed by alpha- and beta-product backbones, we conducted LUMiSizer® measurements in which the transmission of NIR light is measured through multiple positions along the cuvette while samples are subjected to centripetal forces (Fig. 6). We focused on the mixtures of alpha/beta leu and pla in the absence or presence of Gly. The transmission profiles of the samples and the calculated instability indices are shown in Fig. 6 and *SI Appendix*, Figs. S240–S247. Polyesters and depsipeptides of beta-hydroxy acid backbones exhibited lower turbidity and higher transmission than the corresponding alpha backbones, as indicated by the transmission profiles. In the case of pla, beta backbones underwent physical changes to a greater extent than the alpha backbones. Additionally, beta backbones were found to have significantly higher instability indexes than their alpha-backbone counterparts. Interestingly, most cases of depsipeptide formation resulted in a significant decrease in turbidity compared to polyester controls and seemed to have a greater destabilization effect, particularly in the case of beta-hydroxy acid backbones, as indicated by the instability index.

## Discussion

In this study, we investigated the assembly properties of various hydrophobic and predominantly aliphatic depsipeptide backbones. Our objective was to explore whether protopeptides are capable of self-assembly into compartment-like structures, and whether this assembly could have been a driving force for prebiotic chemical selection of the alpha backbone of extant proteins. We found that some protopeptides assemble in aqueous solutions with subtle variations in characteristics depending on their backbone. For hydroxy acid monomers with a moderately hydrophobic side-chain, only alpha-product derivatives assembled, whereas for more hydrophobic derivatives, both alpha- and beta-products exhibited assembly. While both hydrophobic alpha- and beta-product derivatives exhibited assembly, alpha derivatives exhibited slower phase-separation in solution than their beta counterparts, suggesting the formation of more stable structures. Indeed, analysis using LUMiSizer® revealed greater physical stability of alpha- over beta-depsipeptide assemblies in solution. This suggests a possible selection pressure for alpha-protein backbone and a richer chemical space that could have been explored during early chemical evolution, offering insights into the origins of the biological molecules we observe in life today.

Our results indicate that assembly formation is dependent first and foremost on the identity of the hydroxy acid, with some changes in either density or polydispersity of assemblies formed between alpha-, beta-, and gamma-derivative depsipeptides. Microdroplet formation requires reaction products that contain hydroxy acids with a hydrophobic side-chain and sufficient conversion of monomers into longer oligomers. In the first matrix, only the hydrophobic alpha hydroxy acid formed assemblies (lac), suggesting that both backbone and hydrophobicity are crucial for assembly formation. Yet, in the second matrix, which contained considerably more hydrophobic hydroxy acids than the first, both the alpha- and beta hydroxy acids formed assemblies, even though the drying product oligomers of the second matrix were shorter than those of the first. These results indicate that monomer hydrophobicity compensates for oligomer chain length.

While our results indicate that assembly formation is dependent on the identity of the hydroxy acid, the polymerization of the depsipeptides depends on the backbone of the amino acid monomers used (α, β, or γ), in agreement with previous findings (29). In some cases, we observed changes in either density or polydispersity of assemblies formed between alpha-, beta-, and gamma-derivative depsipeptides. These observations could be attributed to degree of polymerization as well as the hydrophobic nature of the assemblies. In the case of aromatic hydroxy acids, assembly was probably promoted due to both π–π stacking interactions and hydrophobic effects (81, 82). These assemblies might facilitate unique and complex chemistries in a low-water environment (compared to the outer aqueous environment) that could have been crucial for the emergence of life and the first protocells. Notably, in smaller assemblies, the surface area-to-volume ratio is significantly higher than that of larger assemblies, making them more efficient for exchanging materials between the protocell and its environment. While these assemblies are likely unstructured (29), it is possible that an increase in structural order will emerge upon enrichment of amide bonds over ester bonds (43). A natural consequence of an increase in polymer stability and folding would be the emergence of function such as catalysis. It remains to be seen whether such increase in structural organization could give rise to rudimentary catalytic activity within such compartments.

Our study demonstrates and characterizes assembly properties of prebiotic depsipeptide systems. In addition, it makes a direct comparison between alpha- and beta-protopeptide backbones in the context of assembly. Our findings shed light on the role of depsipeptides, not only as molecular hubs for prebiotic peptide synthesis (9) but also as potential constructors of new microenvironments. Self-assembly capability is a crucial prerequisite of life (83, 84). Our results demonstrate that depsipeptides readily form structures that could have provided a possible life-supporting environment in the prebiotic era. In addition, our results demonstrate that microdroplet formation is not significantly influenced by chirality, as both _L_-lac and _DL_- lac formed assemblies. It is possible that other depsipeptide mixtures will exhibit chirality-based differences in assembly morphology. Moreover, while our reactions were only carried out for 1 wk under drying conditions, it is possible that following prolonged reactions involving wet-dry cycling, in which amide bonds will be enriched over ester bonds (43), some chiral-based differences will be observed. Furthermore, we have shown that ester bonds were crucial for assembly formation; indeed, directed ester hydrolysis resulted in a lack of assembly formation. This observation could be either attributed to the importance of ester bonds for assembly or to the length of the oligomers themselves.

Investigating the effect of different backbones on the physical stability of formed structures revealed that phase separation of assemblies was significantly backbone-dependent. Interestingly, beta derivatives underwent phase separation faster than alpha derivatives, suggesting that the corresponding alpha derivatives remain dispersed in the solution for a longer duration. We further studied the physical stability of the structures using an LUMiSizer® analytical centrifuge. The transmission profiles as well as the extracted instability indices indicated that alpha backbones are indeed more physically stable than beta backbones. Furthermore, while both depsipeptides and polyesters exhibited comparable physical stability in the alpha form, depsipeptide products of beta backbones were significantly less stable than the corresponding beta esters. These observations can be attributed to the fact that beta backbones introduce more degrees of freedom and alterations in hydrogen bonding compared to alpha backbones (85–89). These observations draw an intriguing picture in the context of protein evolution and offer a possible key mechanism underlying the predominance of alpha monomers in modern proteins.

Contemporary proteins are composed of _L_-alpha-amino acids bound by peptide bonds, suggesting that the selection of a homochiral alpha peptide backbone offered evolutionary advantages. The prebiotic milieu contained a rich variety of building blocks, and many peptide-like backbones could have been possible. Different factors may have affected the selection of the peptide backbone, including the chemical reactivity of the monomers, structural stability, and functionality (14). In the context of structural stability, it has been reported that peptides of alpha backbones form more stable structures in aqueous solutions than their beta counterparts (89). In the context of chemical reactivity, beta monomers were found to react to a similar or even greater extent than alpha monomers (29). This raises questions regarding the evolutionary selection of alpha backbones in a complex mixture of building blocks with various backbones. Our work provides a unique perspective on this conflict.

**Fig. 7.**
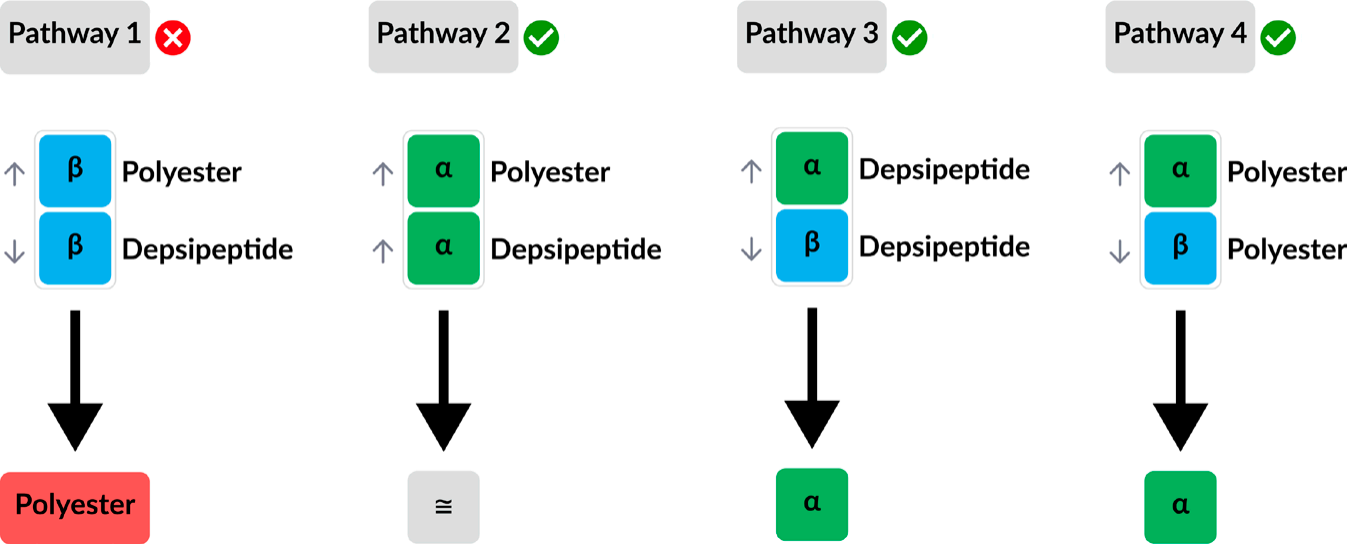
Schematic representation of evolutionary pathways for the selection of α-amino acids, depicted by comparing bond type (alpha- versus beta-) and bond nature (amide versus ester). Four possible pathways are presented: two pathways (Pathway 3 and Pathway 4) focus on the selection of alpha- versus beta-backbones, and two pathways focus on the selection of amide- versus ester- bonds. The comparison illustrated in both pathways 3 and 4 indicates the selection of alpha backbones over beta backbones. The comparison between alpha polyesters and alpha depsipeptides described in pathway 2 revealed no advantage of one bond over the other. However, the corresponding pathway 1 comparing beta polyesters versus beta depsipeptides results in the selection of polyesters. The eventual selection of an amide bond over an ester bond suggests that pathway 1 is not possible.

Here, we suggest a mechanism in which the evolution of the protopeptide bond type (amide/ ester) and the evolution of the backbone linkage (alpha /beta) were intertwined (Fig. 7). By comparing alpha- or beta-backbones of both depsipeptides and polyesters, we have demonstrated that alpha backbones form more stable assemblies than beta backbones. We have also demonstrated that for beta backbones, polyester assemblies are more stable than depsipeptides. These two facts each shed light on the process of selection of alpha backbones over beta backbones in the evolution of peptides; the alpha backbone is favorable because it facilitates the formation of stable assemblies of depsipeptides and polyesters alike, and the beta backbone is unfavorable because it would facilitate the selection of esters over the more chemically stable amide bonds, making it a nonviable pathway for protein evolution. Hence, coevolution of the polymer backbone and bond type may have been a key factor in the selection of the alpha backbone during early protein evolution.

Fahnestock and Rich have shown that the ribosome can catalyze ester bond formation (90). Given this promiscuity of the ribosome in incorporating alpha hydroxy acids in addition to the canonical incorporation of alpha amino acids, it is plausible that early proteins were rich in ester bonds (i.e., polydepsipeptides). Ester linkages likely imposed significant constraints on the ability of such early polymers to form stable tertiary structures, as they disrupt backbone hydrogen-bonding interactions due to the lack of hydrogen bond donors (91–93). Amide bonds were likely favored over esters during protein evolution because they are thermodynamically stable, conformationally constrained, more resistant to hydrolysis compared to ester bonds (80, 94, 95), and contain matched hydrogen bond donors and acceptors that enable the extensive hydrogen-bonding networks required for complex protein structure. Taken together, it is likely that evolutionary selection for the amide backbone arose from the superior chemical and structural properties of amide bonds compared to ester bonds. Nonetheless, such depsipeptides may have played a critical role in early biopolymer evolution. The transient and destabilizing nature of ester bonds could have limited long-term structural stability but simultaneously enabled a more dynamic behavior of primitive polymers. The balance between ester and amide bonds in early polymers may have offered a unique compromise: Esters provided chemical flexibility, ease of synthesis, and susceptibility to exchange, while amide bonds offered durability and structural order. Together, mixed ester and amide backbones could have created a chemical landscape conducive to selection, favoring sequences and linkages that supported assembly, catalysis, self-replication, or other primitive functions. Thus, the early presence of esters might have enabled a crucial intermediate stage in the emergence of functional peptides, bridging the gap between dynamic assemblies and the stable, folded proteins central to life today.

Overall, this study sheds light on the unique structural and chemical properties of alpha backbones as critical factors in the early stages of life’s protopeptide evolution. The prolonged physical stability through self-assembly of alpha depsipeptides suggests that these structures were well suited for further complexification of chemical species during chemical evolution. Additionally, the importance of hydrophobicity in assembly formation highlights the selective advantage of specific monomers. These assemblies and their associated physical stability may have offered the ideal microenvironment and necessary timescales for chemical reactions on early Earth, i.e., “survival of the fittest” (79, 96–98). Our results suggest a possible assembly-driven selection mechanism for the fittest molecules during chemical evolution, which may have led to the emergence of the first protocells. Together, these findings support the hypothesis that the predominance of alpha amino acids in modern biology may have originated from their superior ability to form the physically stable, compartment-like structures essential for the development of early life.

## Materials and Methods

### Materials.

All chemicals were of analytical grade, including glycolic acid (#124737, Merck), L-lactic acid (#199257, Merck), DL-lactic acid (#W261106, Merck), 3-hydroxypropionic acid (#792659, Merck), (S)-3-hydroxybutyric acid (#54925, Merck), 3-phenyllactic acid (#P7251, Merck), 2-Hydroxy-4-methylpentanoic acid (#219819, Merck), 3-hydroxy-4-phenylbutanoic acid (#BD01029150, BLD pharma), 3-hydroxy-5-methylhexanoic acid (#BD727028, BLD pharma), glycine (#G7126, Merck), L-alanine (#A7627, Merck), beta-Alanine (#146064, Merck), (S)-3-aminobutyric acid (#757454, Merck), gamma-aminobutyric acid (#A2129, Merck), and (S)-4-aminopentanoic acid HCl (#BD01430551, BLD pharma).

### Single Step Dry-Down Reactions.

Aqueous solutions of a single hydroxy acid and aqueous solutions of a single amino acid were mixed in a 5:1 molar ratio (in favor of the hydroxy acid) and dried with an open cap at 85 °C for 7 d under unbuffered conditions (pH~2-4). Dried samples containing glycolic acid, lactic acid, 3-hydroxypropionic acid, and 3-hydroxybutanoic acid were then resuspended with 500 µL 4:1 (vol/vol) water:acetonitrile solution to a final concentration of 100 mM (referring to starting amino acid concentration). Dried samples containing 3-phenyllactic acid (α-pla), 3-hydroxy-4-phenylbutanoic acid (β-pla), 2-Hydroxy-4-methylpentanoic (α-leu), and 3-hydroxy-5-methylhexanoic (β-leu) acid were resuspended with 500 µL 1:1 (vol/vol) water:acetonitrile solution to a final concentration of 40 mM (referring to starting amino acid concentration). All samples were then sonicated for 30 s and vortexed. As a control, dry-down reactions were performed with either a single hydroxy acid alone or a single amino acid alone.

### Electrospray Ionization–Mass Spectrometry (ESI–MS).

MS spectra were acquired using an Agilent Infinity Lab LC/MSD XT (G6135C) single quadrupole mass spectrometer with a source fragmentation voltage of 70 V in both negative and positive modes. Samples of glycolic acid, lactic acid, 3-hydroxypropionic acid, and 3-hydroxybutanoic acid were diluted in water to 1 mM (referring to starting amino acid concentration). Samples of phenyllactic acid, 3-hydroxy-4-phenylbutanoic acid, 2-Hydroxy-4-methylpentanoic, and 3-Hydroxy-5-methylhexanoic acid were diluted in water–acetonitrile mixture 1:1 to 0.25 mM (referring to the original amino acid concentration). The diluted samples were directly infused into the mass spectrometer using the following parameters: Running solvent: LC–MS grade water with a flow rate of 0.5 mL/min. Injection volume: 10 μL. Scan range: 50 to 1,500 m/z. MS data were processed using a suite of macros in Igor Pro 9.0.

### HPLC.

All runs were performed on an Agilent infinity II 1260 HPLC system from Agilent Technologies (CA, USA), equipped with a quaternary pump (G7104C), autosampler (G7167A), a column compartment (G7116A), and a DAD UV-vis detector (G7115A). The samples were separated on an Agilent InfinityLab Poroshell 120A EC-C18 3.0 × 150 mm, 2.7 μm particle size analytical column. The following conditions were used: The mobile phases consisted of water with 0.1% formic acid (solvent A) and acetonitrile (solvent B). Column compartment was set at 30 °C.

Samples of glycolic acid, lactic acid, 3-hydroxypropionic acid, and 3-hydroxybutanoic acid were diluted in water to 10 mM (referring to the original amino acid concentration). A 30 min gradient was set: 0 to 3 min, isocratic at 100% A, 3 to 15 min, linear from 100% A to 20% A + 80% B, 15 to 15.1 min, linear from 20% A + 80% B to 100% B, 15.1 to 21 min, isocratic at 100% B, 21 to 21.1 min, linear from 100% B to 100% A, 21.1 to 30 min, isocratic at 100% A (re-equilibration). Flow rate: 0.3 mL/ min.

Samples of phenyllactic acid, 3-hydroxy-4-phenylbutanoic acid, 2-Hydroxy-4-methylpentanoic, and 3-Hydroxy-5-methylhexanoic acid, were diluted in water–acetonitrile mixture 1:1 to 2.5 mM (referring to the original amino acid concentration). A 50 min gradient was set: 0 to 5 min, isocratic at 100% A, 5 to 25 min, linear from 100% A to 20% A + 80% B, 25 to 25.1 min, linear from 20% A + 80% B to 100% B, 25.1 to 35 min, isocratic at 100% B, 35 to 35.1 min, linear from 100% B to 100% A, 35.1 to 50 min, isocratic at 100% A (re-equilibration). Flow rate: 0.3 mL/ min. The chromatographic data were collected and processed using OpenLab CDS software and MATLAB.

### ATR–FTIR Spectroscopy.

IR spectra were analyzed using a Jasco (Tokyo, Japan) FT/IR-4X1 Spectrometer. Prior to analysis, 20 μL of the 100 mM samples, or 50 μL of the 40 mM samples (referring to starting amino acid concentration), were placed on either aluminum foil (samples of phenyllactic acid, 3-hydroxy-4-phenylbutanoic acid, 2-Hydroxy-4-methylpentanoic acid, and 3-hydroxy-5-methylhexanoic acid) or on Durapore® hydrophobic PVDF membranes with a pore size of 0.22 µm (#GVHP04700, Millipore Sigma) (samples of glycolic acid, lactic acid, 3-hydroxypropionic acid and 3-hydroxybutanoic acid, and also 3-phenyllactic acid + γ-Aba, 3-phenyllactic acid + γ-Apa and 2-Hydroxy-4-methylpentanoic + Ala) and allowed to dry fully. The membrane/ aluminum foil was then placed in an Attenuated Total Reflectance (ATR) sample chamber for analysis of the dried sample. Spectra were signal averaged (60 scans per spectrum) and background-subtracted and ranged from 400 to 4,000 cm^−1^. Spectra were analyzed using MATLAB.

### Ester Bonds Hydrolysis Reactions.

Dried samples of lactic acid and Gly (50 μmol Gly, 250 μmol lac) were rehydrated with 500 μL of water, followed by 30 s sonication and vortexing. Each sample was then separated into two aliquots—one served as a nonhydrolyzed control and was stored at −80 °C and the other was covered with a layer of silicone oil (Merck, #378356) to prevent evaporation and remained with a closed cap at 85 °C for 7 d. The samples were then resuspended with water and acetonitrile to a final volume of 4:1 (vol/vol) prior to chemical and structural analysis.

### Light Microscopy Imaging.

2 µL samples were placed on microscope glass slides. The cover glass was then taped to a slide with double-sided adhesive tape to avoid coalescence. Images were taken using an ECHO Revolve microscope (ECHO, San Diego CA) equipped with an 8MP CMOS Color camera, with a magnification of X60 Olympus® air objective. To test whether assemblies are still visible after prolonged incubation at room temperature, we incubated dried products of L-lactic acid and glycine for 7 wk under ambient conditions. Following incubation, samples were vortexed and sonicated for 1 min prior to light microscopy observation. To test whether assemblies are still visible after freezing and thawing, dried products of L-lactic acid and glycine that had been incubated for 7 wk under ambient conditions were frozen at −80 °C for one day and thawed, followed by vortex and sonication for 1 min prior to light microscopy observation.

### Accelerated Stability Analysis.

Measurements were performed on a multisample analytical centrifuge—LUMiSizer® (L.U.M. GmbH, Berlin, Germany) using the STEP™Technology (Space- and Time-resolved Extinction Profiles), which facilitates measurement of the intensity of the transmitted light as a function of time and position over the entire sample length simultaneously. Briefly, samples of 400 µL were placed in a 2 mm PA cell (catalog PA 110-134XY) and then subjected to 800 rpm rotor speed at 25 °C. A total of 150 profiles were recorded at 865 nm in intervals of 5 s. Instability indices were calculated using the SEPView® software.

## Supplementary Material

Appendix 01 (PDF)

## Data Availability

All study data are included in the article and/or *SI Appendix*. All code used in this study is available on Github (99–101).
