# Supplementary material for: Protopeptide backbone affects assembly in aqueous solutions
Source: Proc Natl Acad Sci U S A. 2025 Sep 30;122(40):e2500503122. doi: 10.1073/pnas.2500503122 (PMC12519194; doi:10.1073/pnas.2500503122)
Supplement: Supplementary file 1 — Appendix 01 (PDF) [file pnas.2500503122.sapp.pdf]

Supplementary Information

Proto-peptide Backbone Affects Assembly in Aqueous Solutions

Sarah Fisher[a‡], Yishi Ezerzer[a‡], Rotem Edri[a], Daniil Akulenko[a], Eliav Marland[a], and Moran Frenkel-Pinter[a,b*]

[‡] Authors contributed equally

[a] Institute of Chemistry, The Hebrew University of Jerusalem, Israel 9190401

[b] The Center for Nanoscience and Nanotechnology, The Hebrew University of Jerusalem, Israel 9190401

[*] Corresponding author:

Dr. Moran Frenkel-Pinter
The Center for Nanoscience and Nanotechnology
Institute of Chemistry
The Hebrew University of Jerusalem
Edmond J. Safra Campus
Jerusalem 9190401, Israel
Ph: (+972)-2-6584171
moran.fp@mail.huji.ac.il

Table of Contents

Supplementary Figures 14

Supplementary Figure S1. ESI-MS spectrum of a dry-down reaction of samples A1 – Glycolic acid and glycine. 14

Supplementary Figure S2. ESI-MS spectrum of a dry-down reaction of samples A2 – Glycolic acid and L-alanine. 15

Supplementary Figure S3. ESI-MS spectrum of a dry-down reaction of samples A3 – Glycolic acid and β-alanine. 16

Supplementary Figure S4. ESI-MS spectrum of a dry-down reaction of samples A4– Glycolic acid and β-aminobutyric acid. 17

Supplementary Figure S5. ESI-MS spectrum of a dry-down reaction of samples A5– Glycolic acid and γ-aminobutyric acid. 18

Supplementary Figure S6. ESI-MS spectrum of a dry-down reaction of samples A6– Glycolic acid and 4-aminopentanoic acid. 19

Supplementary Figure S7. ESI-MS spectrum of a dry-down reaction of samples A7– L-lactic acid and glycine. 20

Supplementary Figure S8. ESI-MS spectrum of a dry-down reaction of samples A8– L-lactic acid and L-alanine. 21

Supplementary Figure S9. ESI-MS spectrum of a dry-down reaction of samples A9– L-lactic acid and β-alanine. 22

Supplementary Figure S10. ESI-MS spectrum of a dry-down reaction of samples A10 – L-lactic acid and β-aminobutyric acid. 23

Supplementary Figure S11. ESI-MS spectrum of a dry-down reaction of samples A11 – L-lactic acid and γ-aminobutyric acid. 24

Supplementary Figure S12. ESI-MS spectrum of a dry-down reaction of samples A12 – L-lactic acid and 4-aminopentanoic acid. 25

Supplementary Figure S13. ESI-MS spectrum of a dry-down reaction of samples A13 – Hydroxybutyric acid and glycine. 26

Supplementary Figure S14. ESI-MS spectrum of a dry-down reaction of samples A14 – Hydroxybutyric acid and L-alanine. 27

Supplementary Figure S15. ESI-MS spectrum of a dry-down reaction of samples A15 – Hydroxybutyric acid and β-alanine. 28

Supplementary Figure S16. ESI-MS spectrum of a dry-down reaction of samples A16 – Hydroxybutyric acid and β-aminobutyric acid. 29

Supplementary Figure S17. ESI-MS spectrum of a dry-down reaction of samples A17 – Hydroxybutyric acid and γ-aminobutyric acid. 30

Supplementary Figure S18. ESI-MS spectrum of a dry-down reaction of samples A18 – Hydroxybutyric acid and 4-aminopentanoic acid. 31

Supplementary Figure S19. ESI-MS spectrum of a dry-down reaction of samples A19 – Hydroxypropionic acid and glycine. 32

Supplementary Figure S20. ESI-MS spectrum of a dry-down reaction of samples A20 – Hydroxypropionic acid and L-alanine. 33
Supplementary Figure S21. ESI-MS spectrum of a dry-down reaction of samples A21 – Hydroxypropionic acid and β-alanine. 34
Supplementary Figure S22. ESI-MS spectrum of a dry-down reaction of samples A22 – Hydroxypropionic acid and β-aminobutyric acid. 35
Supplementary Figure S23. ESI-MS spectrum of a dry-down reaction of samples A23 – Hydroxypropionic acid and γ-aminobutyric acid. 36
Supplementary Figure S24. ESI-MS spectrum of a dry-down reaction of samples A24 – Hydroxypropionic acid and 4-aminopentanoic acid. 37
Supplementary Figure S25. ESI-MS spectrum of a dry-down reaction of samples A25 – DL-lactic acid and glycine. 38
Supplementary Figure S26. ESI-MS spectrum of a dry-down reaction of samples A26 – DL-lactic acid and L-alanine. 39
Supplementary Figure S27. ESI-MS spectrum of a dry-down reaction of samples A27 – DL-lactic acid and β-alanine. 40
Supplementary Figure S28. ESI-MS spectrum of a dry-down reaction of samples A28 – DL-lactic acid and β-aminobutyric acid. 41
Supplementary Figure S29. ESI-MS spectrum of a dry-down reaction of samples A29 – DL-lactic acid and γ-aminobutyric acid. 42
Supplementary Figure S30. ESI-MS spectrum of a dry-down reaction of samples A30 – DL-lactic acid and 4-aminopentanoic acid. 43
Supplementary Figure S31. FTIR spectrum of sample A1- glycolic acid and glycine. 44
Supplementary Figure S32. FTIR spectrum of sample A2- glycolic acid and L-alanine. 45
Supplementary Figure S33. FTIR spectrum of sample A3- glycolic acid and β-alanine. 46
Supplementary Figure S34. FTIR spectrum of sample A4- glycolic acid and β-aminobutyric acid. 47
Supplementary Figure S35. FTIR spectrum of sample A5- glycolic acid and γ-aminobutyric acid. 48
Supplementary Figure S36. FTIR spectrum of sample A6- glycolic acid and 4-aminopentanoic acid. 49
Supplementary Figure S37. FTIR spectrum of sample A7- L-lactic acid and glycine. 50
Supplementary Figure S38. FTIR spectrum of sample A8- L-lactic acid and L-alanine. 51
Supplementary Figure S39. FTIR spectrum of sample A9- L-lactic acid and β-alanine. 52
Supplementary Figure S40. FTIR spectrum of sample A10- L-lactic acid and β-aminobutyric acid. 53
Supplementary Figure S41. FTIR spectrum of sample A11- L-lactic acid and γ-aminobutyric acid. 54
Supplementary Figure S42. FTIR spectrum of sample A12- L-lactic acid and 4-aminopentanoic acid. 55

Supplementary Figure S43. FTIR spectrum of sample A13- Hydroxybutyric acid and glycine. 56
Supplementary Figure S44. FTIR spectrum of sample A14- Hydroxybutyric acid and L-alanine. 57
Supplementary Figure S45. FTIR spectrum of sample A15- Hydroxybutyric acid and β-alanine. 58
Supplementary Figure S46. FTIR spectrum of sample A16- Hydroxybutyric acid and β-aminobutyric acid. 59
Supplementary Figure S47. FTIR spectrum of sample A17- Hydroxybutyric acid and γ-aminobutyric acid. 60
Supplementary Figure S48. FTIR spectrum of sample A18- Hydroxybutyric acid and 4-aminopentanoic acid. 61
Supplementary Figure S49. FTIR spectrum of sample A19- Hydroxypropionic acid and glycine. 62
Supplementary Figure S50. FTIR spectrum of sample A20- Hydroxypropionic acid and L-alanine. 63
Supplementary Figure S51. FTIR spectrum of sample A21- Hydroxypropionic acid and β-alanine. 64
Supplementary Figure S52. FTIR spectrum of sample A22- Hydroxypropionic acid and β-aminobutyric acid. 65
Supplementary Figure S53. FTIR spectrum of sample A23- Hydroxypropionic acid and γ-aminobutyric acid. 66
Supplementary Figure S54. FTIR spectrum of sample A24- Hydroxypropionic acid and 4-aminopentanoic acid. 67
Supplementary Figure S55. FTIR spectrum of sample A25- DL-lactic acid and glycine. 68
Supplementary Figure S56. FTIR spectrum of sample A26- DL-lactic acid and L-alanine. 69
Supplementary Figure S57. FTIR spectrum of sample A27- DL-lactic acid and β-alanine. 70
Supplementary Figure S58. FTIR spectrum of sample A28- DL-lactic acid and β-aminobutyric acid 71
Supplementary Figure S59. FTIR spectrum of sample A29- DL-lactic acid and γ-aminobutyric acid. 72
Supplementary Figure S60. FTIR spectrum of sample A30- DL-lactic acid and 4-aminopentanoic acid. 73
Supplementary Figure S61. HPLC overlay chromatogram of 3 repeats of glycolic acid and glycine reaction products. 74
Supplementary Figure S62. HPLC overlay chromatogram of 3 repeats of glycolic acid and L-alanine reaction products. 75
Supplementary Figure S63. HPLC overlay chromatogram of 3 repeats of glycolic acid and β-alanine reaction products. 76
Supplementary Figure S64. HPLC overlay chromatogram of 3 repeats of glycolic acid and β-aminobutyric acid reaction products. 77

Supplementary Figure S65. HPLC overlay chromatogram of 3 repeats of glycolic acid and γ-aminobutyric acid reaction products. 78
Supplementary Figure S66. HPLC overlay chromatogram of 3 repeats of glycolic acid and 4-aminopentanoic acid reaction products. 79
Supplementary Figure S67. HPLC overlay chromatogram of 3 repeats of L-lactic acid and glycine reaction products. 80
Supplementary Figure S68. HPLC overlay chromatogram of 3 repeats of L-lactic acid and L-alanine 81
Supplementary Figure S69. HPLC overlay chromatogram of 3 repeats of L-lactic acid and β-alanine reaction products. 82
Supplementary Figure S70. HPLC overlay chromatogram of 3 repeats of L-lactic acid and β-aminobutyric acid reaction products. 83
Supplementary Figure S71. HPLC overlay chromatogram of 3 repeats of L-lactic acid and γ-aminobutyric acid reaction products. 84
Supplementary Figure S72. HPLC overlay chromatogram of 3 repeats of L-lactic acid and 4-aminopentanoic acid reaction products. 85
Supplementary Figure S73. HPLC overlay chromatogram of 3 repeats of Hydroxybutyric acid and glycine reaction products. 86
Supplementary Figure S74. HPLC overlay chromatogram of 3 repeats of Hydroxybutyric acid and L-alanine reaction products. 87
Supplementary Figure S75. HPLC overlay chromatogram of 3 repeats of Hydroxybutyric acid and β-alanine reaction products. 88
Supplementary Figure S76. HPLC overlay chromatogram of 3 repeats of Hydroxybutyric acid and β-aminobutyric acid reaction products. 89
Supplementary Figure S77. HPLC overlay chromatogram of 3 repeats of Hydroxybutyric acid and γ-aminobutyric acid reaction products. 90
Supplementary Figure S78. HPLC overlay chromatogram of 3 repeats of Hydroxybutyric acid and 4-aminopentanoic acid reaction products. 91
Supplementary Figure S79. HPLC overlay chromatogram of 3 repeats of Hydroxypropionic acid and glycine reaction products. 92
Supplementary Figure S80. HPLC overlay chromatogram of 3 repeats of Hydroxypropionic acid and L-alanine reaction products. 93
Supplementary Figure S81. HPLC overlay chromatogram of 3 repeats of Hydroxypropionic acid and β-alanine reaction products. 94
Supplementary Figure S82. HPLC overlay chromatogram of 3 repeats of Hydroxypropionic acid and β-aminobutyric acid reaction products. 95
Supplementary Figure S83. HPLC overlay chromatogram of 3 repeats of Hydroxypropionic acid and γ-aminobutyric acid reaction products. 96
Supplementary Figure S84. HPLC overlay chromatogram of 3 repeats of Hydroxypropionic acid and 4-aminopentanoic acid reaction products. 97

Supplementary Figure S85. HPLC overlay chromatogram of 3 repeats of DL-lactic acid and glycine reaction products. 98

Supplementary Figure S86. HPLC overlay chromatogram of 3 repeats of DL-lactic acid and L-alanine reaction products. 99

Supplementary Figure S87. HPLC overlay chromatogram of 3 repeats of DL-lactic acid and β-alanine reaction products. 100

Supplementary Figure S88. HPLC overlay chromatogram of 3 repeats of DL-lactic acid and β-aminobutyric acid reaction products. 101

Supplementary Figure S89. HPLC overlay chromatogram of 3 repeats of DL-lactic acid and γ-aminobutyric acid reaction products. 102

Supplementary Figure S90. HPLC overlay chromatogram of 3 repeats of DL-lactic acid and 4-aminopentanoic acid reaction products. 103

Supplementary Table S1. List of samples 104

Supplementary Figure S91. ESI-MS of dry down of amino acid control samples – Glycine, Alanine, β-Alanine, β-Aminobutyric acid, γ-Aminobutyric acid, 4-Aminopentanoic acid. 105

Supplementary Figure S92. ESI-MS of dry down of Hydroxy acid control samples – Glycolic acid, L-lactic acid, Hydroxybutyric acid, Hydroxypropionic acid, DL-lactic acid. 106

Supplementary Figure S93. FTIR spectrum of amino acid control samples. 107

Supplementary Figure S94. FTIR spectrum of Hydroxy acid control samples. 108

Supplementary Figure S95. HPLC of dry down and fresh amino acid control samples – Glycine, Alanine, β-Alanine, β-Aminobutyric acid, γ-Aminobutyric acid, 4-Aminopentanoic acid. 109

Supplementary Figure S96. HPLC of dry down and fresh Hydroxy acid control samples – Glycolic acid, L-lactic acid, Hydroxybutyric acid, Hydroxypropionic acid, DL-lactic acid. 110

Supplementary Figure S97. – Visual appearance 113

Supplementary Figure S98. Brightfield microscopy imaging of depsipeptides resulting from dry-down reactions between glycolic acid and Glycine 114

Supplementary Figure S99. Brightfield microscopy imaging of depsipeptides resulting from dry-down reactions between glycolic acid and L-alanine 115

Supplementary Figure S100. Brightfield microscopy imaging of depsipeptides resulting from dry-down reactions between glycolic acid and β-alanine 116

Supplementary Figure S101. Brightfield microscopy imaging of depsipeptides resulting from dry-down reactions between glycolic acid and β-aminobutyric acid 117

Supplementary Figure S102. Brightfield microscopy imaging of depsipeptides resulting from dry-down reactions between glycolic acid and γ-aminobutyric acid 118

Supplementary Figure S103. Brightfield microscopy imaging of depsipeptides resulting from dry-down reactions between glycolic acid and 4-aminopentanoic acid 119

Supplementary Figure S104. Brightfield microscopy imaging of depsipeptides resulting from dry-down reactions between L-lactic acid and Glycine 120

Supplementary Figure S105. Brightfield microscopy imaging of depsipeptides resulting from dry-down reactions between L-lactic acid and L-alanine 121

Supplementary Figure S106. Brightfield microscopy imaging of depsipeptides resulting from dry-down reactions between L-lactic acid and β-alanine... 122

Supplementary Figure S107. Brightfield microscopy imaging of depsipeptides resulting from dry-down reactions between L-lactic acid and β-aminobutyric acid 123

Supplementary Figure S108. Brightfield microscopy imaging of depsipeptides resulting from dry-down reactions between L-lactic acid and γ-aminobutyric acid.................................... 124

Supplementary Figure S109. Brightfield microscopy imaging of depsipeptides resulting from dry-down reactions between L-lactic acid and 4-aminopentanoic acid............................... 125

Supplementary Figure S110. Brightfield microscopy imaging of depsipeptides resulting from dry-down reactions between Hydroxybutyric acid and glycine 126

Supplementary Figure S111. Brightfield microscopy imaging of depsipeptides resulting from dry-down reactions between Hydroxybutyric acid and L-alanine.. 127

Supplementary Figure S112. Brightfield microscopy imaging of depsipeptides resulting from dry-down reactions between Hydroxybutyric acid and β-alanine 128

Supplementary Figure S113. Brightfield microscopy imaging of depsipeptides resulting from dry-down reactions between Hydroxybutyric acid and β-aminobutyric acid 129

Supplementary Figure S114. Brightfield microscopy imaging of depsipeptides resulting from dry-down reactions between Hydroxybutyric acid and γ-aminobutyric acid....................... 130

Supplementary Figure S115. Brightfield microscopy imaging of depsipeptides resulting from dry-down reactions between Hydroxybutyric acid and 4-aminopentanoic acid 131

Supplementary Figure S116. Brightfield microscopy imaging of depsipeptides resulting from dry-down reactions between Hydroxypropionic acid and glycine 132

Supplementary Figure S117. Brightfield microscopy imaging of depsipeptides resulting from dry-down reactions between Hydroxypropionic acid and L-alanine.................................... 133

Supplementary Figure S118. Brightfield microscopy imaging of depsipeptides resulting from dry-down reactions between Hydroxypropionic acid and β-alanine 134

Supplementary Figure S119. Brightfield microscopy imaging of depsipeptides resulting from dry-down reactions between Hydroxypropionic acid and β-aminobutyric acid 135

Supplementary Figure S120. Brightfield microscopy imaging of depsipeptides resulting from dry-down reactions between Hydroxypropionic acid and γ-aminobutyric acid................... 136

Supplementary Figure S121. Brightfield microscopy imaging of depsipeptides resulting from dry-down reactions between Hydroxypropionic acid and 4-aminopentanoic acid 137

Supplementary Figure S122. Brightfield microscopy imaging of depsipeptides resulting from dry-down reactions between DL-lactic acid and glycine 138

Supplementary Figure S123. Brightfield microscopy imaging of depsipeptides resulting from dry-down reactions between DL-lactic acid and L-alanine... 139

Supplementary Figure S124. Brightfield microscopy imaging of depsipeptides resulting from dry-down reactions between DL-lactic acid and β-alanine 140

Supplementary Figure S125. Brightfield microscopy imaging of depsipeptides resulting from dry-down reactions between DL-lactic acid and β-aminobutyric acid 141
Supplementary Figure S126. Brightfield microscopy imaging of depsipeptides resulting from dry-down reactions between DL-lactic acid and γ-aminobutyric acid 142
Supplementary Figure S127. Brightfield microscopy imaging of depsipeptides resulting from dry-down reactions between DL-lactic acid and 4-aminopentanoic acid 143
Supplementary Figure S128. Brightfield microscopy imaging of fresh control samples of L-lactic acid and all amino 144
Supplementary Figure S129. Brightfield microscopy imaging of depsipeptides in 100% aqueous solutions at various concentrations. 145
Supplementary Figure S130. Brightfield microscopy imaging of depsipeptides in 8 146
Supplementary Figure S131. Brightfield microscopy imaging of depsipeptides in 60 147
Supplementary Figure S132. Brightfield microscopy imaging of depsipeptides demonstrates stability of microdroplet structures. 148
Supplementary Figure S133. Concentration-dependent assembly formation of L-Lactic acid & Glycine. 149
Supplementary Figure S134. Temperature-dependent assembly formation of L-Lactic acid & Glycine. 150
Supplementary Figure S135. Hydrolysis of L-lactic acid and Glycine negative MS spectrum. 151
Supplementary Figure S136. Hydrolysis of L-lactic acid negative MS spectrum. 152
Supplementary Figure S137. ESI-MS spectrum of a dry-down reaction of sample NA1 – Phenyllactic acid and glycine. 153
Supplementary Figure S138. ESI-MS spectrum of a dry-down reaction of sample NA2 – Phenyllactic acid and L-alanine. 154
Supplementary Figure S139. ESI-MS spectrum of a dry-down reaction of sample NA3 – Phenyllactic acid and β-alanine. 155
Supplementary Figure S140. ESI-MS spectrum of a dry-down reaction of sample NA4 – Phenyllactic acid and β-aminobutyric acid. 156
Supplementary Figure S141. ESI-MS spectrum of a dry-down reaction of sample NA5 – Phenyllactic acid and γ-aminobutyric acid. 157
Supplementary Figure S142. ESI-MS spectrum of a dry-down reaction of sample NA6 – Phenyllactic acid and 4-aminopentanoic acid. 158
Supplementary Figure S143. ESI-MS spectrum of a dry-down reaction of sample NA7 – Leucic acid and Glycine. 159
Supplementary Figure S144. ESI-MS spectrum of a dry-down reaction of sample NA8 – Leucic acid and L-alanine. 160
Supplementary Figure S145. ESI-MS spectrum of a dry-down reaction of sample NA9 – Leucic acid and β-alanine. 161

Supplementary Figure S146. ESI-MS spectrum of a dry-down reaction of sample NA10 – Leucic acid and β-aminobutyric acid. 162
Supplementary Figure S147. ESI-MS spectrum of a dry-down reaction of sample NA11 – Leucic acid and γ-aminobutyric acid. 163
Supplementary Figure S148. ESI-MS spectrum of a dry-down reaction of sample NA12 – Leucic acid and 4-aminopentanoic acid. 164
Supplementary Figure S149. ESI-MS spectrum of a dry-down reaction of sample NA13 – β-Phenyllactic acid and Glycine. 165
Supplementary Figure S150. ESI-MS spectrum of a dry-down reaction of sample NA14 – β-Phenyllactic acid and L-alanine. 166
Supplementary Figure S151. ESI-MS spectrum of a dry-down reaction of sample NA15 – β-Phenyllactic acid and β-alanine. 167
Supplementary Figure S152. ESI-MS spectrum of a dry-down reaction of sample NA16 – β-Phenyllactic acid and β-aminobutyric acid. 168
Supplementary Figure S153. ESI-MS spectrum of a dry-down reaction of sample NA17 – β-Phenyllactic acid and γ-aminobutyric acid. 169
Supplementary Figure S154. ESI-MS spectrum of a dry-down reaction of sample NA18 – β-Phenyllactic acid and 4-aminopentanoic acid. 170
Supplementary Figure S155. ESI-MS spectrum of a dry-down reaction of sample NA19 – β-Leucic acid and Glycine. 171
Supplementary Figure S156. ESI-MS spectrum of a dry-down reaction of sample NA20 – β-Leucic acid and L-alanine. 172
Supplementary Figure S157. ESI-MS spectrum of a dry-down reaction of sample NA21 – β-Leucic acid and β-alanine. 173
Supplementary Figure S158. ESI-MS spectrum of a dry-down reaction of sample NA22 – β-Leucic acid and β-aminobutyric acid. 174
Supplementary Figure S159. ESI-MS spectrum of a dry-down reaction of sample NA23 – β-Leucic acid and γ-aminobutyric acid. 175
Supplementary Figure S160. ESI-MS spectrum of a dry-down reaction of sample NA24 – β-Leucic acid and 4-aminopentanoic acid. 176
Supplementary Figure S161. FTIR spectrum of sample NA1- Phenyllactic acid and Glycine. 177
Supplementary Figure S162. FTIR spectrum of sample NA2- Phenyllactic acid and L-alanine. 178
Supplementary Figure S163. FTIR spectrum of sample NA3- Phenyllactic acid and β-alanine. 179
Supplementary Figure S164. FTIR spectrum of sample NA4- Phenyllactic acid and β-aminobutyric acid. 180
Supplementary Figure S165. FTIR spectrum of sample NA5- Phenyllactic acid and γ-aminobutyric acid. 181
Supplementary Figure S166. FTIR spectrum of sample NA6- Phenyllactic acid and 4-aminopentanoic acid. 182

Supplementary Figure S167. FTIR spectrum of sample NA7- Leucic acid and Glycine. 183
Supplementary Figure S168. FTIR spectrum of sample NA8- Leucic acid and L-alanine....... 184
Supplementary Figure S169. FTIR spectrum of sample NA9- Leucic acid and β-alanine. 185
Supplementary Figure S170. FTIR spectrum of sample NA10- Leucic acid and β-aminobutyric acid. 186
Supplementary Figure S171. FTIR spectrum of sample NA11- Leucic acid and γ-aminobutyric acid. 187
Supplementary Figure S172. FTIR spectrum of sample NA12- Leucic acid and 4-aminopentanoic acid. 188
Supplementary Figure S173. FTIR spectrum of sample NA13- β-Phenyllactic acid and Glycine. 189
Supplementary Figure S174. FTIR spectrum of sample NA14- β-Phenyllactic acid and L-alanine. 190
Supplementary Figure S175. FTIR spectrum of sample NA15- β-Phenyllactic acid and β-alanine. 191
Supplementary Figure S176. FTIR spectrum of sample NA16- β-Phenyllactic acid and β-aminobutyric acid. 192
Supplementary Figure S177. FTIR spectrum of sample NA17- β-Phenyllactic acid and γ-aminobutyric acid. 193
Supplementary Figure S178. FTIR spectrum of sample NA18- β-Phenyllactic acid and 4-aminopentanoic acid. 194
Supplementary Figure S179. FTIR spectrum of sample NA19- β-Leucic acid and Glycine. ... 195
Supplementary Figure S180. FTIR spectrum of sample NA20- β-Leucic acid and L-alanine. 196
Supplementary Figure S181. FTIR spectrum of sample NA21- β-Leucic acid and β-alanine. 197
Supplementary Figure S182. FTIR spectrum of sample NA22- β-Leucic acid and β-aminobutyric acid. 198
Supplementary Figure S183. FTIR spectrum of sample NA23- β-Leucic acid and γ-aminobutyric acid. 199
Supplementary Figure S184. FTIR spectrum of sample NA24- β-Leucic acid and 4-aminopentanoic acid. 200
Supplementary Figure S185. HPLC overlay chromatogram of 3 repeats of Phenyllactic acid and Glycine. 201
Supplementary Figure S186. HPLC overlay chromatogram of 3 repeats of Phenyllactic acid and L-alanine. 202
Supplementary Figure S187. HPLC overlay chromatogram of 3 repeats of Phenyllactic acid and β-alanine. 203
Supplementary Figure S188. HPLC overlay chromatogram of 3 repeats of Phenyllactic acid and β-aminobutyric acid. 204
Supplementary Figure S189. HPLC overlay chromatogram of 3 repeats of Phenyllactic acid and γ-aminobutyric acid. 205

Supplementary Figure S190. HPLC overlay chromatogram of 3 repeats of Phenyllactic acid and 4-aminopentanoic acid. 206
Supplementary Figure S191. HPLC overlay chromatogram of 3 repeats of Leucic acid and Glycine. 207
Supplementary Figure S192. HPLC overlay chromatogram of 3 repeats of Leucic acid and L-alanine. 208
Supplementary Figure S193. HPLC overlay chromatogram of 3 repeats of Leucic acid and β-alanine. 209
Supplementary Figure S194. HPLC overlay chromatogram of 3 repeats of Leucic acid and β-aminobutyric acid. 210
Supplementary Figure S195. HPLC overlay chromatogram of 3 repeats of Leucic acid and γ-aminobutyric acid. 211
Supplementary Figure S196. HPLC overlay chromatogram of 3 repeats of Leucic acid and 4-aminopentanoic acid. 212
Supplementary Figure S197. HPLC overlay chromatogram of 3 repeats of β-Phenyllactic acid and Glycine. 213
Supplementary Figure S198. HPLC overlay chromatogram of 3 repeats of β-Phenyllactic acid and L-alanine. 214
Supplementary Figure S199. HPLC overlay chromatogram of 3 repeats of β-Phenyllactic acid and β-alanine. 215
Supplementary Figure S200. HPLC overlay chromatogram of 3 repeats of β-Phenyllactic acid and β-aminobutyric acid. 216
Supplementary Figure S201. HPLC overlay chromatogram of 3 repeats of β-Phenyllactic acid and γ-aminobutyric acid. 217
Supplementary Figure S202. HPLC overlay chromatogram of 3 repeats of β-Phenyllactic acid and 4-aminopentanoic acid. 218
Supplementary Figure S203. HPLC overlay chromatogram of 3 repeats of β-Leucic acid and Glycine. 219
Supplementary Figure S204. HPLC overlay chromatogram of 3 repeats of β-Leucic acid and L-alanine. 220
Supplementary Figure S205. HPLC overlay chromatogram of 3 repeats of β-Leucic acid and β-alanine. 221
Supplementary Figure S206. HPLC overlay chromatogram of 3 repeats of β-Leucic acid and β-aminobutyric acid. 222
Supplementary Figure S207. HPLC overlay chromatogram of 3 repeats of β-Leucic acid and γ-aminobutyric acid. 223
Supplementary Figure S208. HPLC overlay chromatogram of 3 repeats of β-Leucic acid and 4-aminopentanoic acid. 224
Supplementary Figure S209. ESI-MS spectra of dry down of Hydroxy acid control samples – Phenyllactic acid, Leucic acid, β-Phenyllactic acid, β-Leucic acid. 225

Supplementary Figure S210. HPLC chromatograms of dry down Hydroxy acid control samples – Phenyllactic acid, Leucic acid, β-Phenyllactic acid, β-Leucic acid. 226

Supplementary Figure S211. FTIR overlay spectrum of dry down and fresh control samples of Hydroxy acids – Phenyllactic acid, Leucic acid, β-Phenyllactic acid, β-Leucic acid. 227

Supplementary Table S2. List of samples 228

Supplementary Figure S212. – Visual appearance 230

Supplementary Figure S213. Brightfield microscopy imaging of depsipeptides resulting from dry-down reactions of Phenyllactic acid and Glycine 231

Supplementary Figure S214. Brightfield microscopy imaging of depsipeptides resulting from dry-down reactions of Phenyllactic acid and L-alanine 232

Supplementary Figure S215. Brightfield microscopy imaging of depsipeptides resulting from dry-down reactions of Phenyllactic acid and β-alanine... 233

Supplementary Figure S216. Brightfield microscopy imaging of depsipeptides resulting from dry-down reactions of Phenyllactic acid and β-aminobutyric acid 234

Supplementary Figure S217. Brightfield microscopy imaging of depsipeptides resulting from dry-down reactions of Phenyllactic acid and γ-aminobutyric acid...................................... 235

Supplementary Figure S218. Brightfield microscopy imaging of depsipeptides resulting from dry-down reactions of Phenyllactic acid and 4-aminopentanoic acid.................................. 236

Supplementary Figure S219. Brightfield microscopy imaging of depsipeptides resulting from dry-down reactions of Leucic acid and Glycine 237

Supplementary Figure S220. Brightfield microscopy imaging of depsipeptides resulting from dry-down reactions of Leucic acid and L-alanine 238

Supplementary Figure S221. Brightfield microscopy imaging of depsipeptides resulting from dry-down reactions of Leucic acid and β-alanine 239

Supplementary Figure S222. Brightfield microscopy imaging of depsipeptides resulting from dry-down reactions of Leucic acid and β-aminobutyric acid.. 240

Supplementary Figure S223. Brightfield microscopy imaging of depsipeptides resulting from dry-down reactions of Leucic acid and γ-aminobutyric acid 241

Supplementary Figure S224. Brightfield microscopy imaging of depsipeptides resulting from dry-down reactions of Leucic acid and 4-aminopentanoic acid 242

Supplementary Figure S225. Brightfield microscopy imaging of depsipeptides resulting from dry-down reactions of β-Phenyllactic acid and Glycine... 243

Supplementary Figure S226. Brightfield microscopy imaging of depsipeptides resulting from dry-down reactions of β-Phenyllactic acid and L-alanine... 244

Supplementary Figure S227. Brightfield microscopy imaging of depsipeptides resulting from dry-down reactions of β-Phenyllactic acid and β-alanine 245

Supplementary Figure S228. Brightfield microscopy imaging of depsipeptides resulting from dry-down reactions of β-Phenyllactic acid and β-aminobutyric acid 246

Supplementary Figure S229. Brightfield microscopy imaging of depsipeptides resulting from dry-down reactions of β-Phenyllactic acid and γ-aminobutyric acid 247

Supplementary Figure S230. Brightfield microscopy imaging of depsipeptides resulting from dry-down reactions of β-Phenyllactic acid and 4-aminopentanoic acid 248
Supplementary Figure S231. Brightfield microscopy imaging of depsipeptides resulting from dry-down reactions of β-Leucic acid and Glycine.. 249
Supplementary Figure S232. Brightfield microscopy imaging of depsipeptides resulting from dry-down reactions of β-Leucic acid and L-alanine 250
Supplementary Figure S233. Brightfield microscopy imaging of depsipeptides resulting from dry-down reactions of β-Leucic acid and β-alanine 251
Supplementary Figure S234. Brightfield microscopy imaging of depsipeptides resulting from dry-down reactions of β-Leucic acid and β-aminobutyric acid 252
Supplementary Figure S235. Brightfield microscopy imaging of depsipeptides resulting from dry-down reactions of β-Leucic acid and γ-aminobutyric acid.. 253
Supplementary Figure S236. Brightfield microscopy imaging of depsipeptides resulting from dry-down reactions of β-Leucic acid and 4-aminopentanoic acid....................................... 254
Supplementary Figure S237. Brightfield microscopy imaging of polyesters resulting from dry-down reactions of hydroxy acid from the first batch alone... 255
Supplementary Figure S238. Brightfield microscopy imaging of polyesters resulting from dry-down reactions of hydroxy acid from the second batch alone 256
Supplementary Figure S239. Brightfield microscopy imaging of fresh hydroxy acid control samples.. 257
Supplementary Figure S240. LUMiSizer® transmission profile of α-Phenyllactic acid dry down products... 258
Supplementary Figure S241. LUMiSizer® transmission profile of β-Phenyllactic acid dry down products... 259
Supplementary Figure S242. LUMiSizer® transmission profile of α-Leucic acid dry down products... 260
Supplementary Figure S243. LUMiSizer® transmission profile of β-Leucic acid dry down products... 261
Supplementary Figure S244. LUMiSizer® transmission profile of α-Phenyllactic acid + Glycine dry down products. 262
Supplementary Figure S245. LUMiSizer® transmission profile of β-Phenyllactic acid + Glycine dry down products. 263
Supplementary Figure S246. LUMiSizer® transmission profile of α-Leucic acid + Glycine dry down products.. 264
Supplementary Figure S247. LUMiSizer® transmission profile of β-Leucic acid + Glycine dry down products.. 265

Supplementary Figures

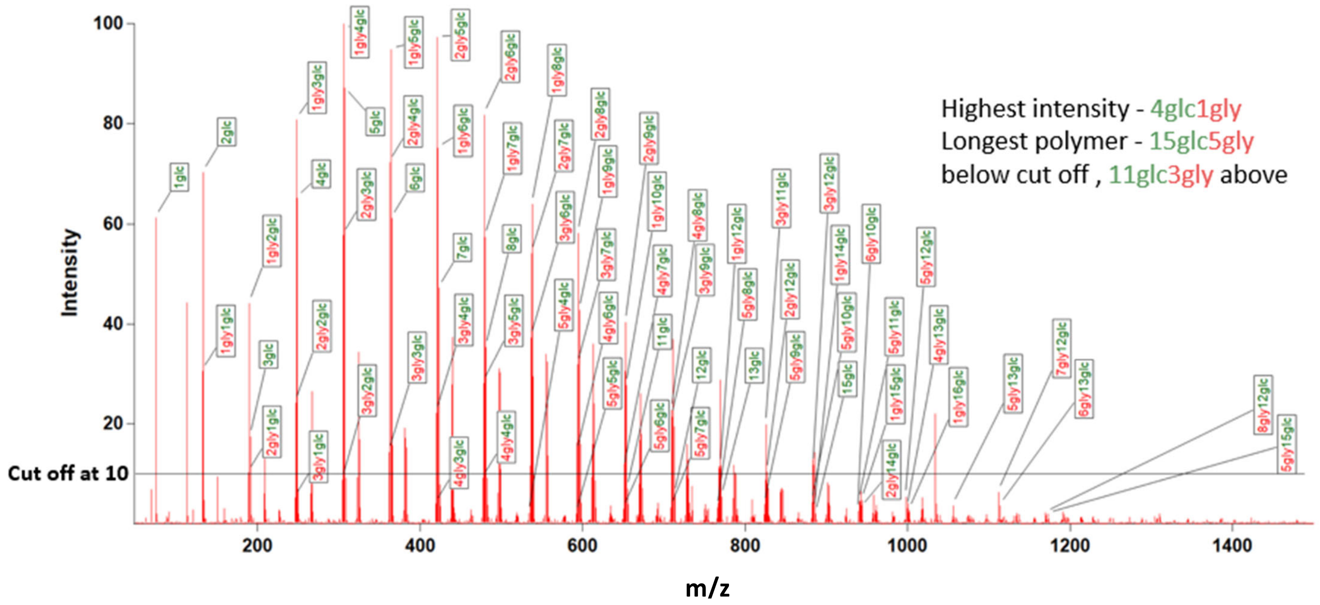

Supplementary Figure S1. ESI-MS spectrum of a dry-down reaction of samples A1 – Glycolic acid and glycine. Glycolic acid (glc) and Glycine (gly) were dried down at a 5:1 molar ratio, in favor of glc, for 7 days at 85 °C. Dry down products were then resuspended in an aqueous solution of 20% acetonitrile in water (v/v). The resulting products were analyzed by negative-mode ESI-MS, indicating a variety of depsipeptides. glc is labeled in green, gly is labeled in red.

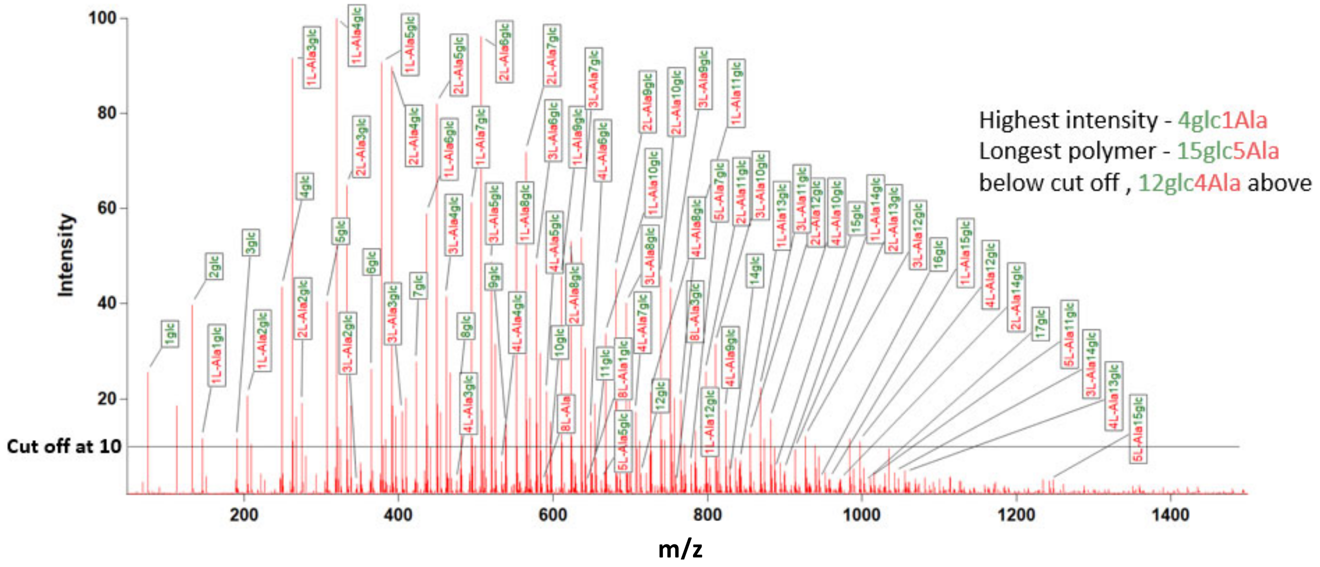

Supplementary Figure S2. ESI-MS spectrum of a dry-down reaction of samples A2 – Glycolic acid and L-alanine. Glycolic acid (glc) and L-Alanine (ala) were dried down at a 5:1 molar ratio, in favor of glc, for 7 days at 85 °C. Dry down products were then resuspended in an aqueous solution of 20% acetonitrile in water (v/v). The resulting products were analyzed by negative-mode ESI-MS, indicating a variety of depsipeptides. glc is labeled in green, ala is labeled in red.

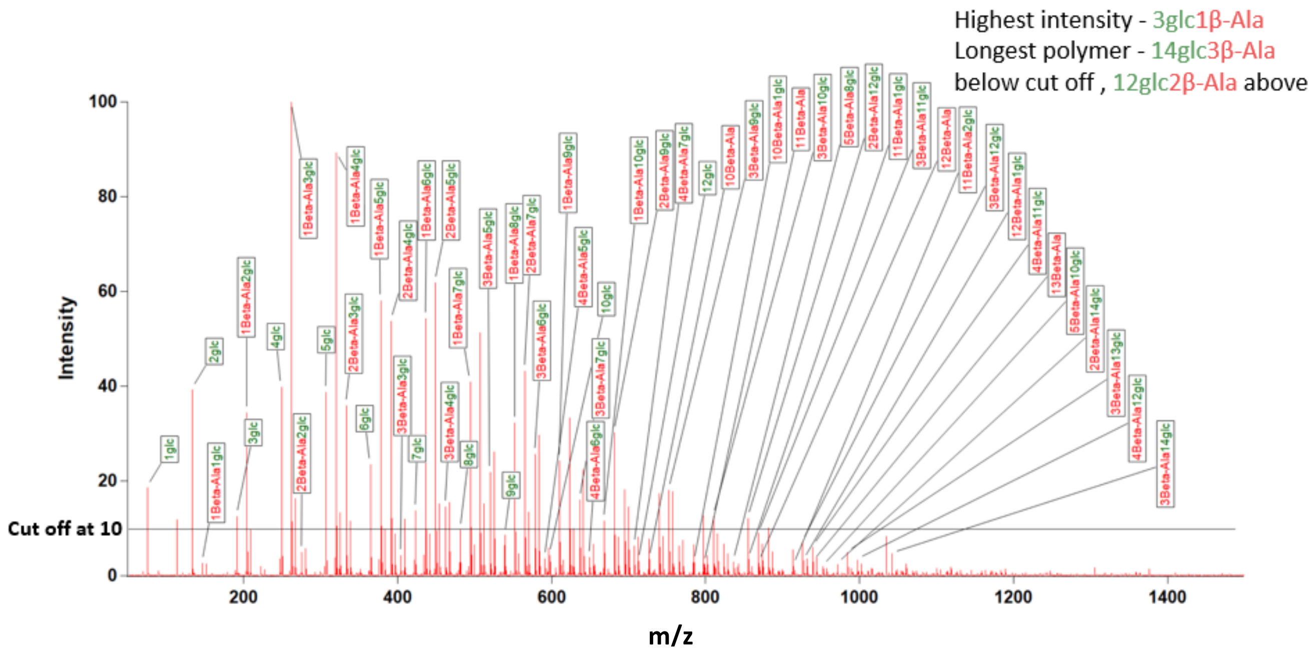

Supplementary Figure S3. ESI-MS spectrum of a dry-down reaction of samples A3 – Glycolic acid and β-alanine. Glycolic acid (glc) and β-Alanine (β-ala) were dried down at a 5:1 molar ratio, in favor of glc, for 7 days at 85 °C. Dry down products were then resuspended in an aqueous solution of 20% acetonitrile in water (v/v). The resulting products were analyzed by negative-mode ESI-MS, indicating a variety of depsipeptides. glc is labeled in green, β-ala is labeled in red.

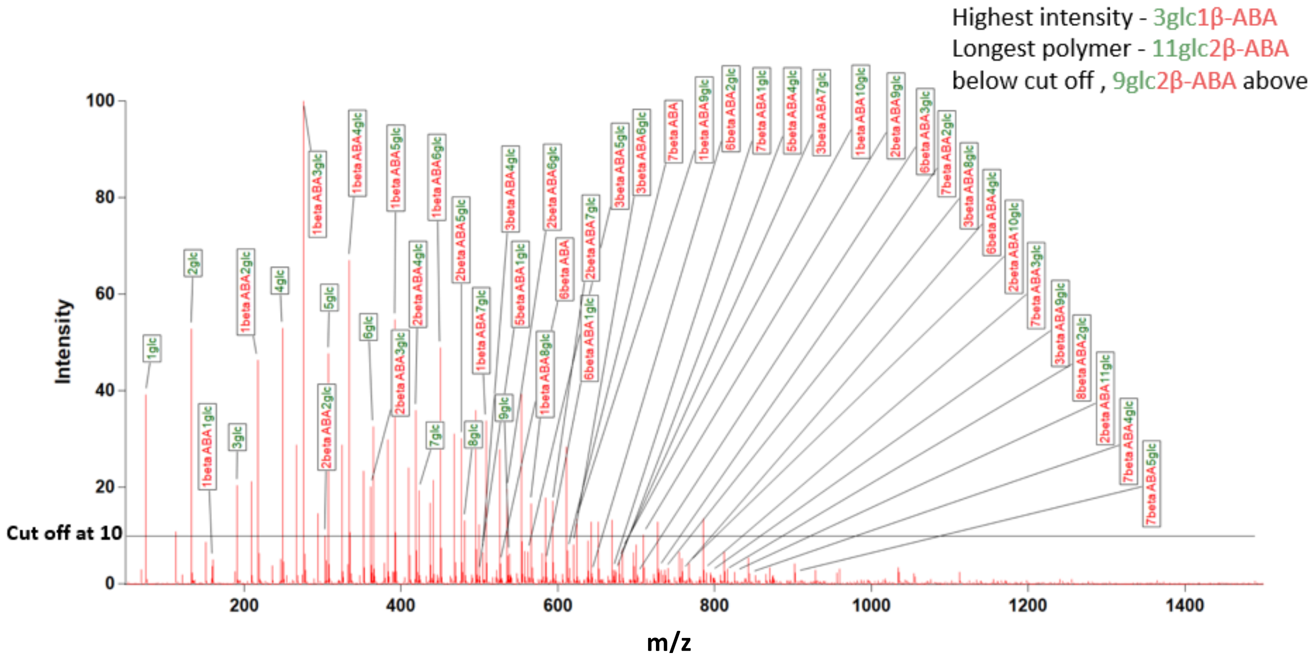

Supplementary Figure S4. ESI-MS spectrum of a dry-down reaction of samples A4– Glycolic acid and β-aminobutyric acid. Glycolic acid (glc) and β-Aminobutyric acid (β-aba) were dried down at a 5:1 molar ratio, in favor of glc, for 7 days at 85 °C. Dry down products were then resuspended in an aqueous solution of 20% acetonitrile in water (v/v). The resulting products were analyzed by negative-mode ESI-MS, indicating a variety of depsipeptides. glc is labeled in green, β-aba is labeled in red.

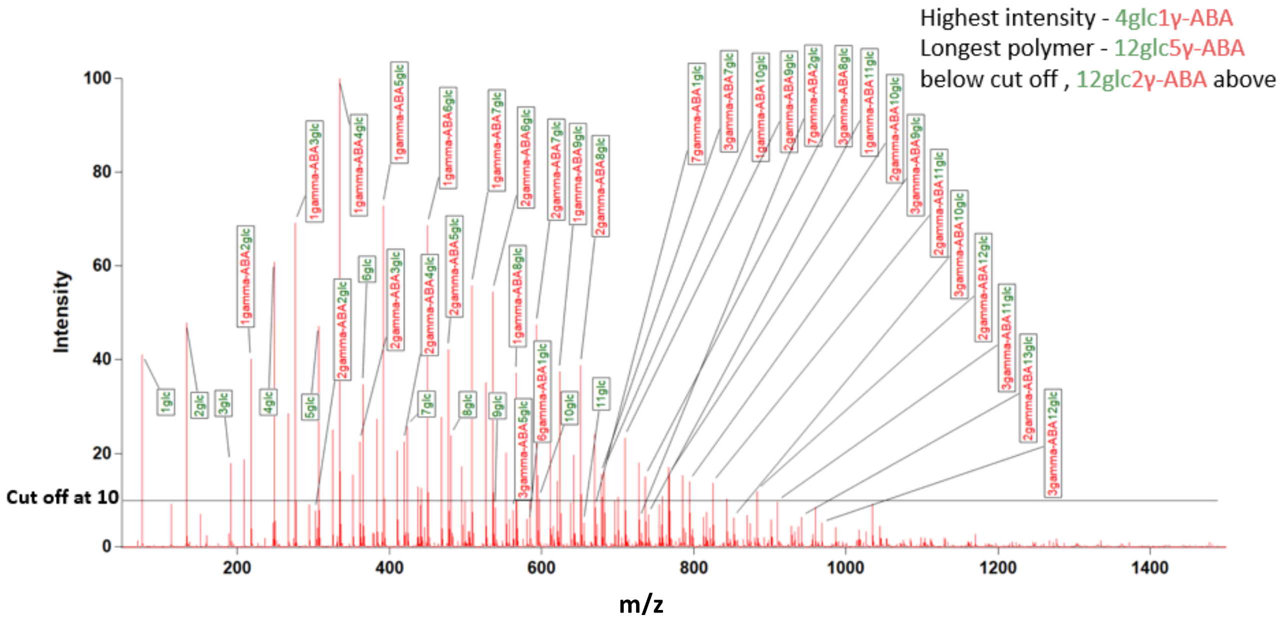

Supplementary Figure S5. ESI-MS spectrum of a dry-down reaction of samples A5– Glycolic acid and γ-aminobutyric acid. Glycolic acid (glc) and γ-Aminobutyric acid (γ-aba) were dried down at a 5:1 molar ratio, in favor of glc, for 7 days at 85 °C. Dry down products were then resuspended in an aqueous solution of 20% acetonitrile in water (v/v). The resulting products were analyzed by negative-mode ESI-MS, indicating a variety of depsipeptides. glc is labeled in green, γ-aba is labeled in red.

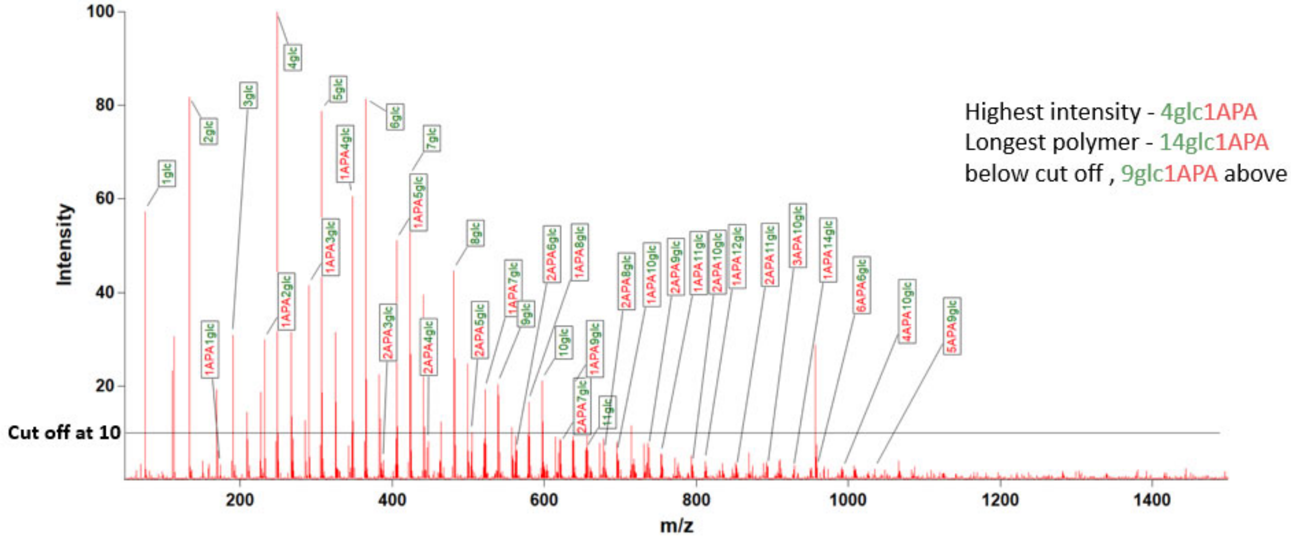

Supplementary Figure S6. ESI-MS spectrum of a dry-down reaction of samples A6– Glycolic acid and 4-aminopentanoic acid. Glycolic acid (glc) and 4-Aminopentanoic acid (Apa) were dried down at a 5:1 molar ratio, in favor of glc, for 7 days at 85 °C. Dry down products were then resuspended in an aqueous solution of 20% acetonitrile in water (v/v). The resulting products were analyzed by negative-mode ESI-MS, indicating a variety of depsipeptides. glc is labeled in green, Apa is labeled in red.

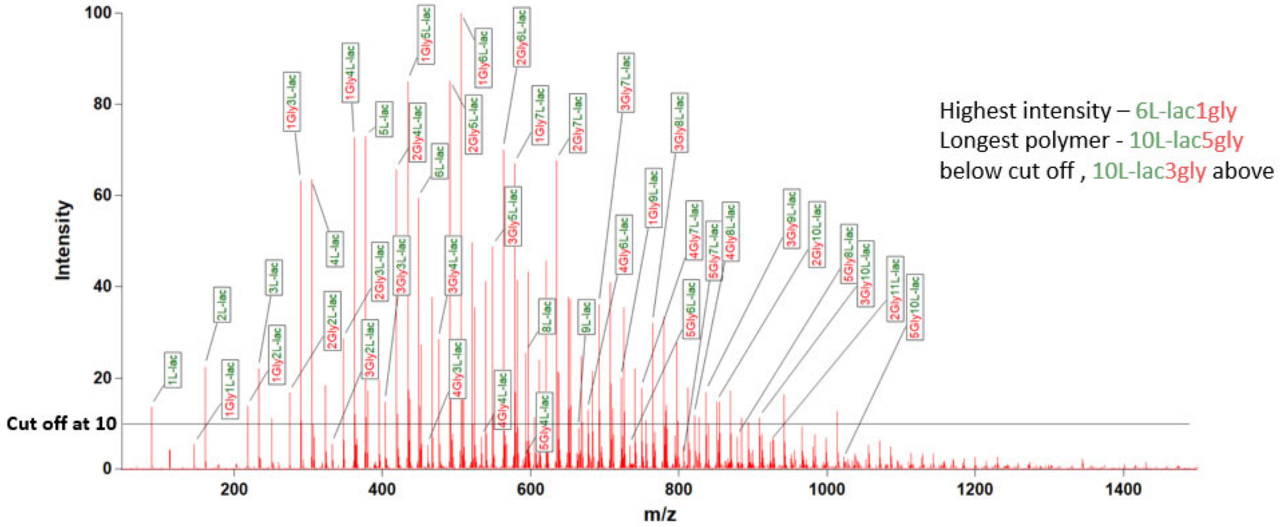

Supplementary Figure S7. ESI-MS spectrum of a dry-down reaction of samples A7– L-lactic acid and glycine. L-lactic acid (lac) and glycine (gly) were dried down at a 5:1 molar ratio, in favor of lac, for 7 days at 85 °C. Dry down products were then resuspended in an aqueous solution of 20% acetonitrile in water (v/v). The resulting products were analyzed by negative-mode ESI-MS, indicating a variety of depsipeptides. lac is labeled in green, gly is labeled in red.

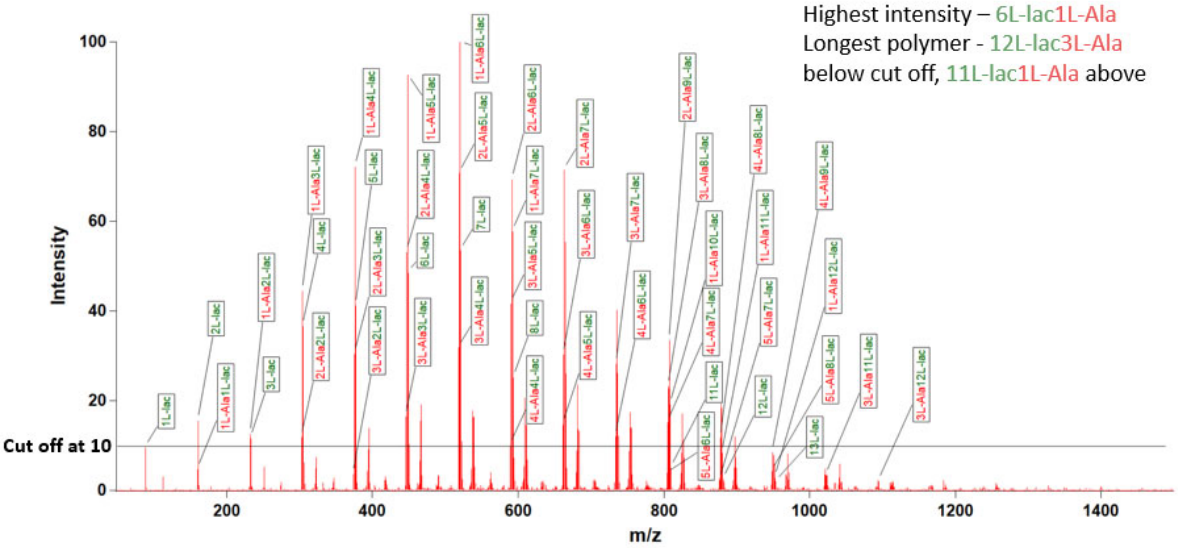

Supplementary Figure S8. ESI-MS spectrum of a dry-down reaction of samples A8– L-lactic acid and L-alanine. L-lactic acid (lac) and L-alanine (ala) were dried down at a 5:1 molar ratio, in favor of lac, for 7 days at 85 °C. Dry down products were then resuspended in an aqueous solution of 20% acetonitrile in water (v/v). The resulting products were analyzed by negative-mode ESI-MS, indicating a variety of depsipeptides. lac is labeled in green, ala is labeled in red.

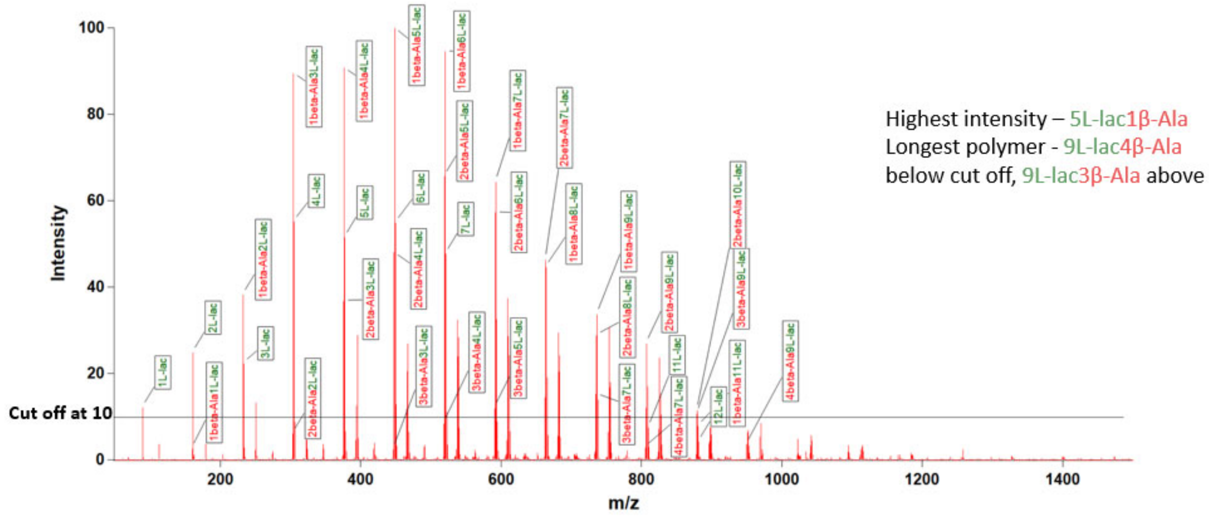

Supplementary Figure S9. ESI-MS spectrum of a dry-down reaction of samples A9– L-lactic acid and β-alanine. L-lactic acid (lac) and β-alanine (β-ala) were dried down at a 5:1 molar ratio, in favor of lac, for 7 days at 85 °C. Dry down products were then resuspended in an aqueous solution of 20% acetonitrile in water (v/v). The resulting products were analyzed by negative-mode ESI-MS, indicating a variety of depsipeptides. lac is labeled in green, β-ala is labeled in red.

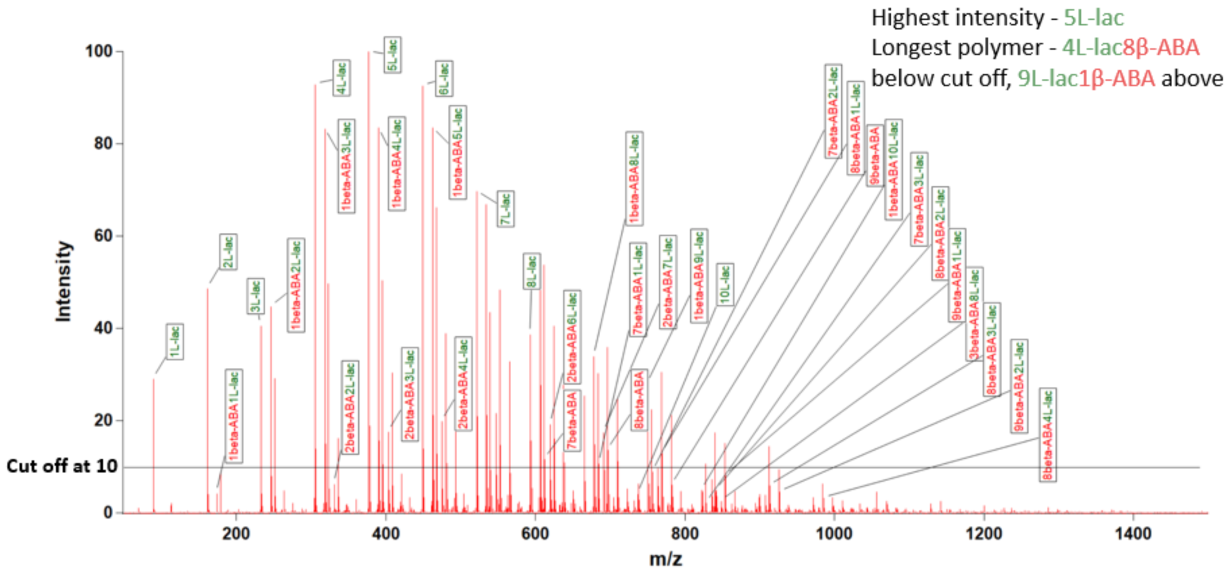

Supplementary Figure S10. ESI-MS spectrum of a dry-down reaction of samples A10 – L-lactic acid and β-aminobutyric acid. L-lactic acid (lac) and β-aminobutyric acid (β-aba) were dried down at a 5:1 molar ratio, in favor of lac, for 7 days at 85 °C. Dry down products were then resuspended in an aqueous solution of 20% acetonitrile in water (v/v). The resulting products were analyzed by negative-mode ESI-MS, indicating a variety of depsipeptides. lac is labeled in green, β-aba is labeled in red.

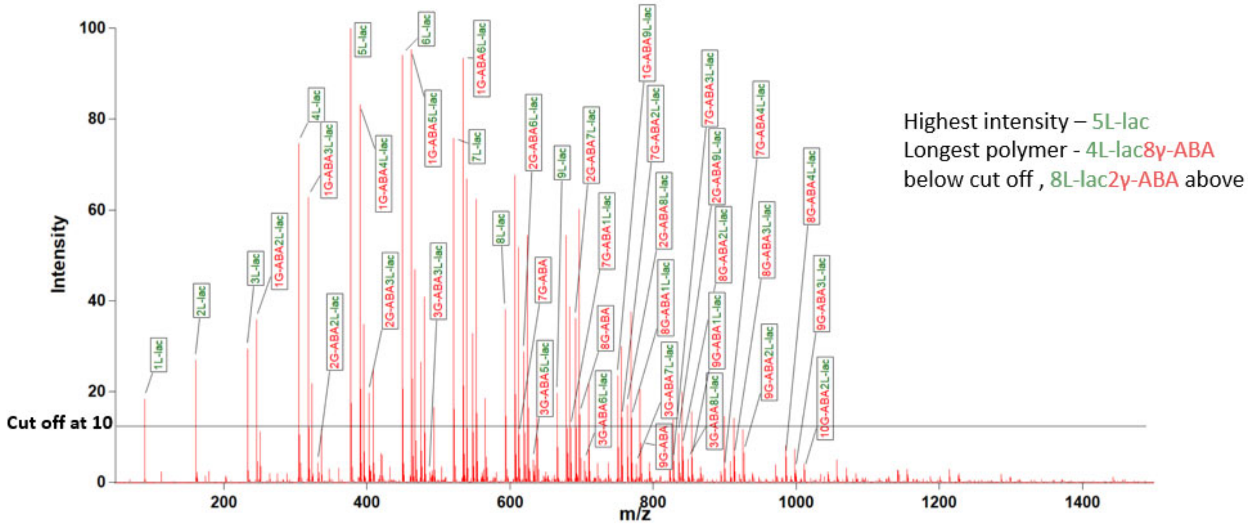

Supplementary Figure S11. ESI-MS spectrum of a dry-down reaction of samples A11 – L-lactic acid and γ-aminobutyric acid. L-lactic acid (lac) and γ-aminobutyric acid (γ-aba) were dried down at a 5:1 molar ratio, in favor of lac, for 7 days at 85 °C. Dry down products were then resuspended in an aqueous solution of 20% acetonitrile in water (v/v). The resulting products were analyzed by negative-mode ESI-MS, indicating a variety of depsipeptides. lac is labeled in green, γ-aba is labeled in red.

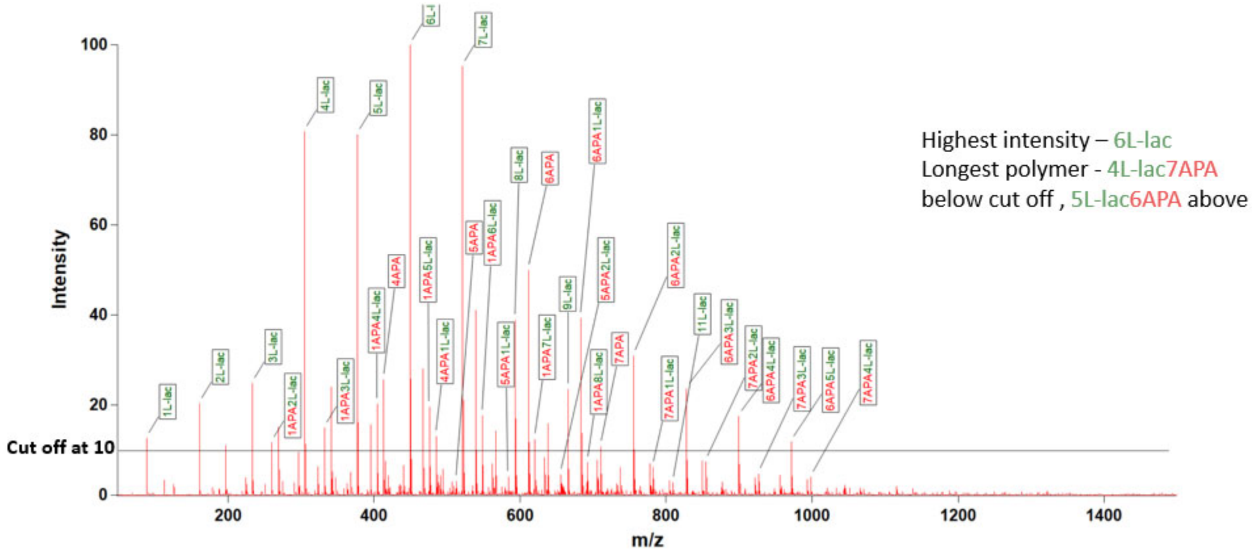

Supplementary Figure S12. ESI-MS spectrum of a dry-down reaction of samples A12 – L-lactic acid and 4-aminopentanoic acid. L-lactic acid (lac) and 4-aminopentanoic acid (Apa) were dried down at a 5:1 molar ratio, in favor of lac, for 7 days at 85 °C. Dry down products were then resuspended in an aqueous solution of 20% acetonitrile in water (v/v). The resulting products were analyzed by negative-mode ESI-MS, indicating a variety of depsipeptides. lac is labeled in green, Apa is labeled in red.

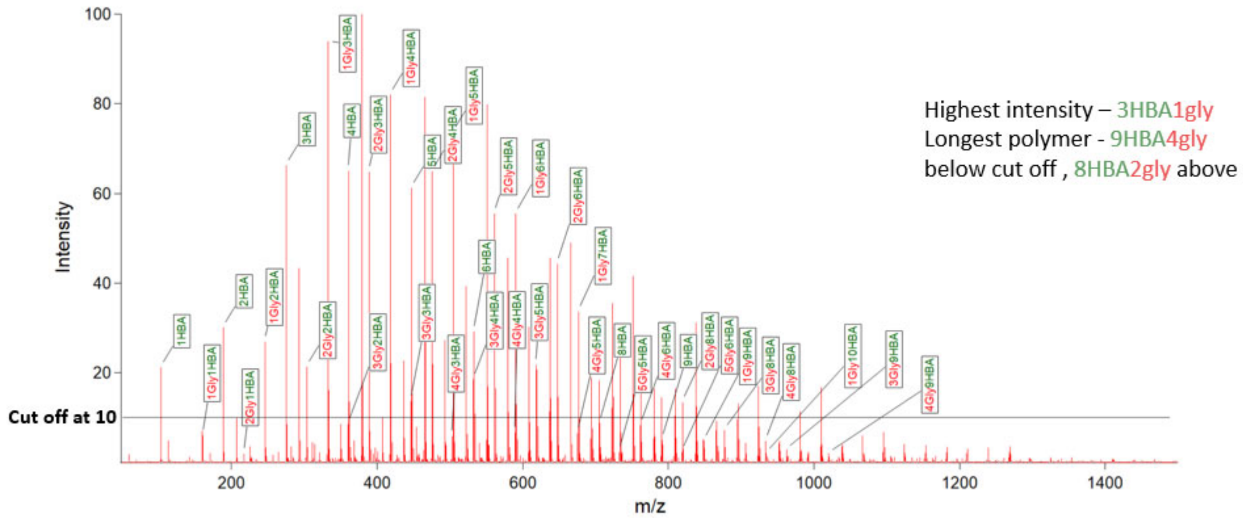

Supplementary Figure S13. ESI-MS spectrum of a dry-down reaction of samples A13 – Hydroxybutyric acid and glycine. Hydroxybutyric acid (hba) and glycine (gly) were dried down at a 5:1 molar ratio, in favor of hba, for 7 days at 85 °C. Dry down products were then resuspended in an aqueous solution of 20% acetonitrile in water (v/v). The resulting products were analyzed by negative-mode ESI-MS, indicating a variety of depsipeptides. hba is labeled in green, gly is labeled in red.

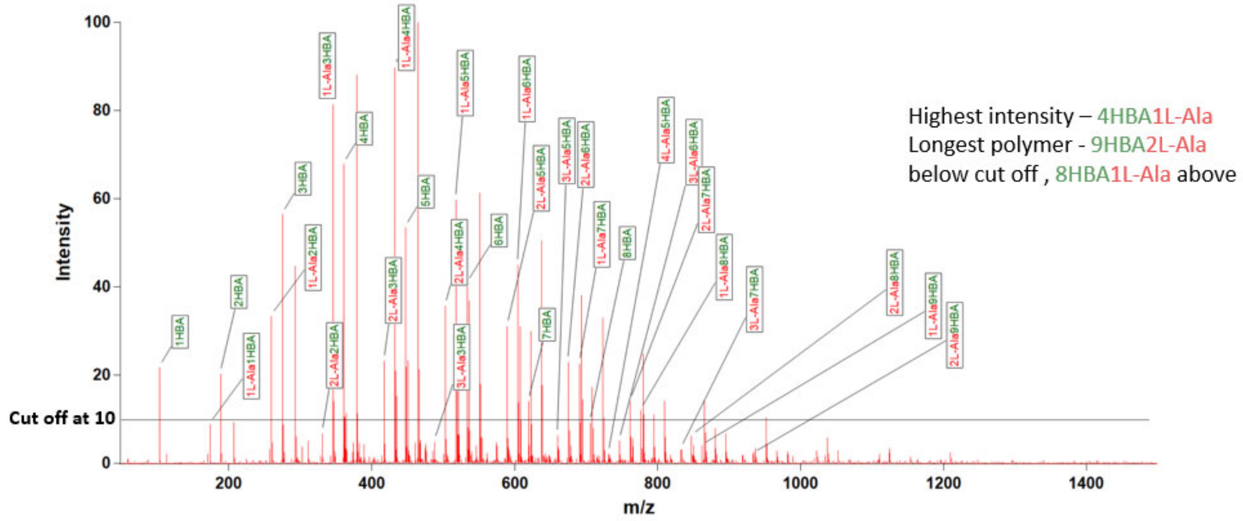

Supplementary Figure S14. ESI-MS spectrum of a dry-down reaction of samples A14 – Hydroxybutyric acid and L-alanine. Hydroxybutyric acid (hba) and L-alanine (ala) were dried down at a 5:1 molar ratio, in favor of hba, for 7 days at 85 °C. Dry down products were then resuspended in an aqueous solution of 20% acetonitrile in water (v/v). The resulting products were analyzed by negative-mode ESI-MS, indicating a variety of depsipeptides. hba is labeled in green, ala is labeled in red.

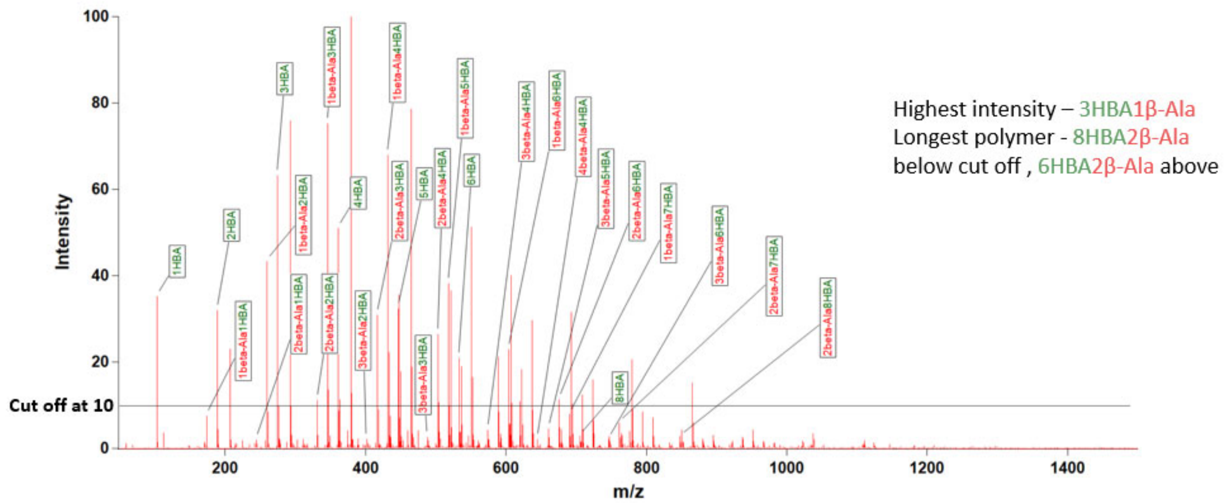

Supplementary Figure S15. ESI-MS spectrum of a dry-down reaction of samples A15 – Hydroxybutyric acid and β-alanine. Hydroxybutyric acid (hba) and β-alanine (β-ala) were dried down at a 5:1 molar ratio, in favor of hba, for 7 days at 85 °C. Dry down products were then resuspended in an aqueous solution of 20% acetonitrile in water (v/v). The resulting products were analyzed by negative-mode ESI-MS, indicating a variety of depsipeptides. hba is labeled in green, β-ala is labeled in red.

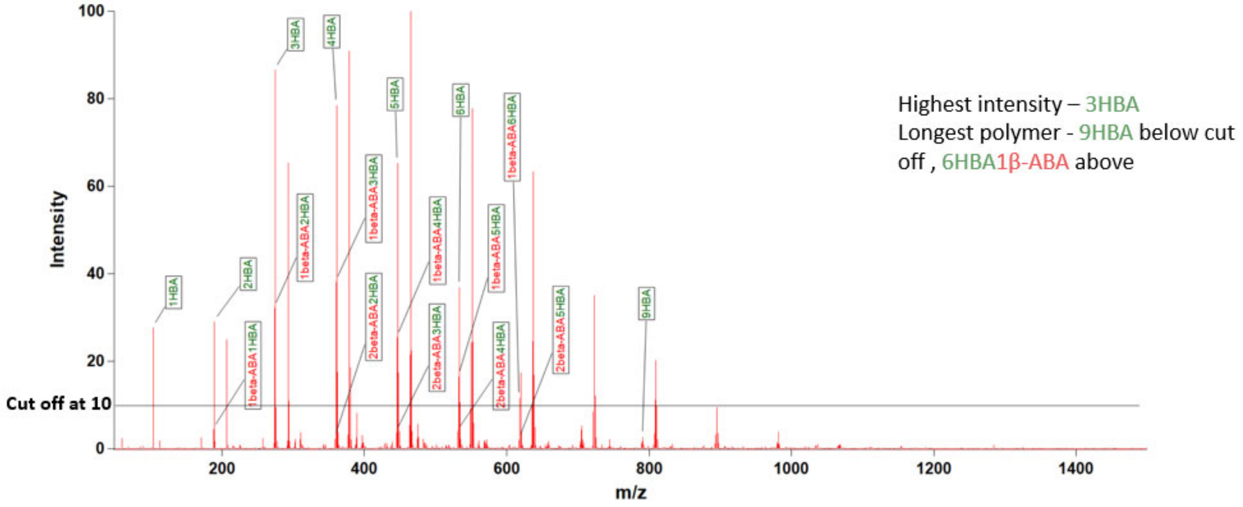

Supplementary Figure S16. ESI-MS spectrum of a dry-down reaction of samples A16 – Hydroxybutyric acid and β-aminobutyric acid. Hydroxybutyric acid (hba) and β aminobutyric acid (β-aba) were dried down at a 5:1 molar ratio, in favor of hba, for 7 days at 85 °C. Dry down products were then resuspended in an aqueous solution of 20% acetonitrile in water (v/v).. The resulting products were analyzed by negative-mode ESI-MS, indicating a variety of depsipeptides. hba is labeled in green, β-aba is labeled in red.

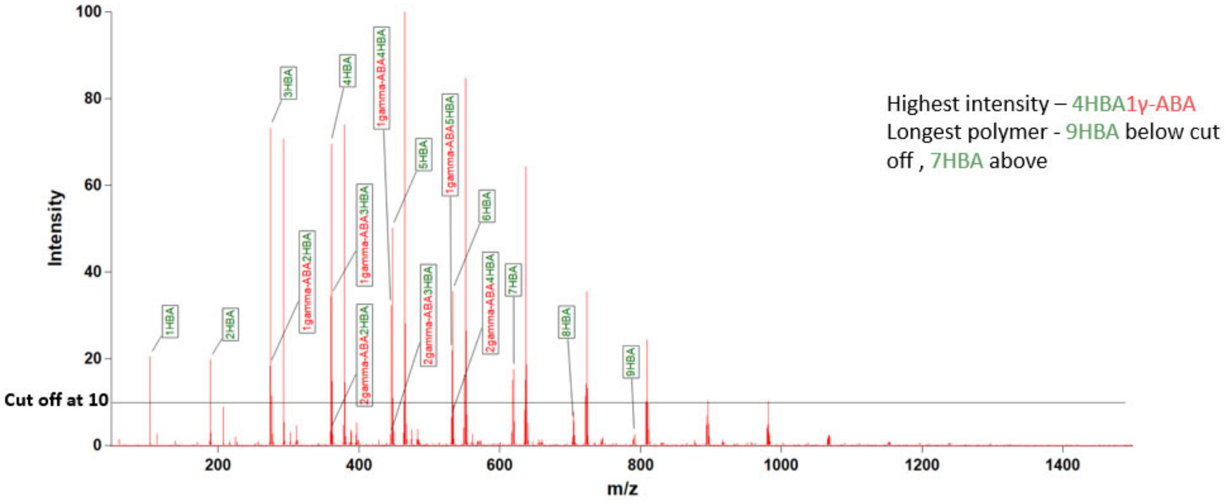

Supplementary Figure S17. ESI-MS spectrum of a dry-down reaction of samples A17 – Hydroxybutyric acid and γ-aminobutyric acid. Hydroxybutyric acid (hba) and γ-aminobutyric acid (γ-aba) were dried down at a 5:1 molar ratio, in favor of hba, for 7 days at 85 °C. Dry down products were then resuspended in an aqueous solution of 20% acetonitrile in water (v/v). The resulting products were analyzed by negative-mode ESI-MS, indicating a variety of depsipeptides. hba is labeled in green, γ-aba is labeled in red.

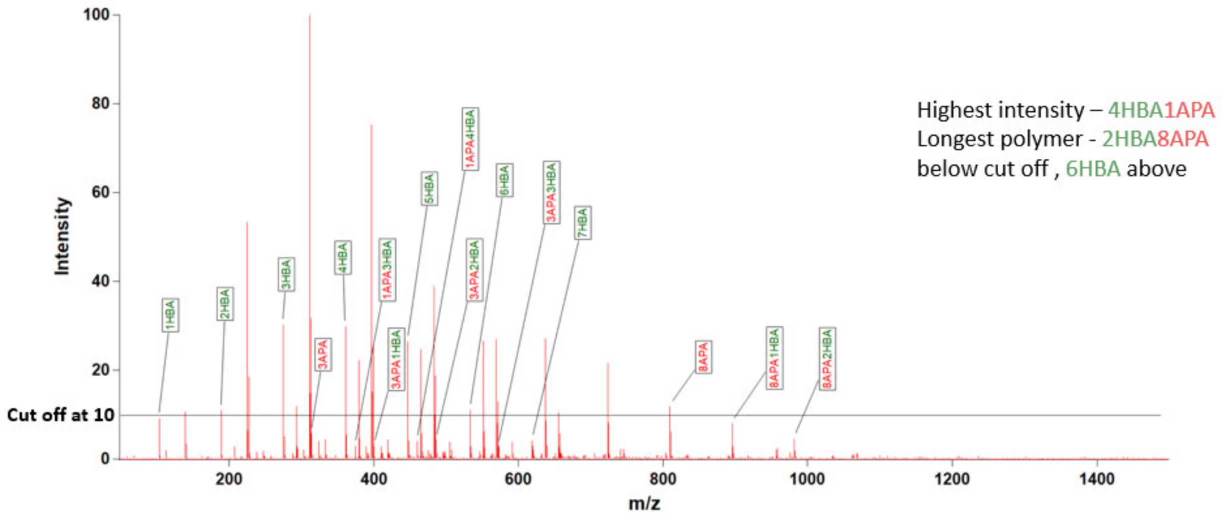

Supplementary Figure S18. ESI-MS spectrum of a dry-down reaction of samples A18 – Hydroxybutyric acid and 4-aminopentanoic acid. Hydroxybutyric acid (hba) and 4-aminopentanoic acid (Apa) were dried down at a 5:1 molar ratio, in favor of hba, for 7 days at 85 °C. Dry down products were then resuspended in an aqueous solution of 20% acetonitrile in water (v/v). The resulting products were analyzed by negative-mode ESI-MS, indicating a variety of depsipeptides. hba is labeled in green, Apa is labeled in red.

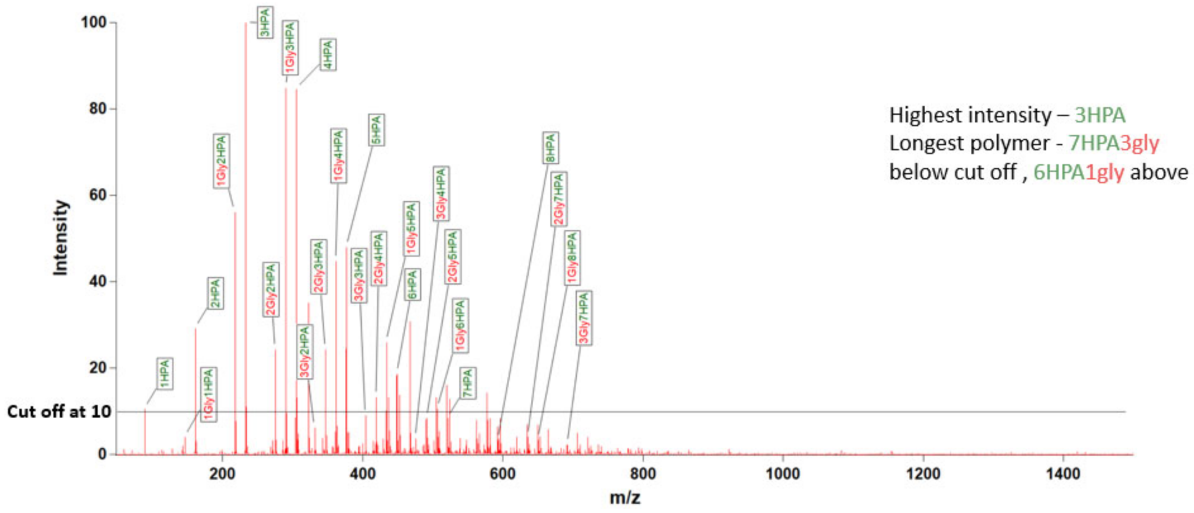

Supplementary Figure S19. ESI-MS spectrum of a dry-down reaction of samples A19 – Hydroxypropionic acid and glycine. Hydroxypropionic acid (hpa) and glycine (gly) were dried down at a 5:1 molar ratio, in favor of hpa, for 7 days at 85 °C. Dry down products were then resuspended in an aqueous solution of 20% acetonitrile in water (v/v). The resulting products were analyzed by negative-mode ESI-MS, indicating a variety of depsipeptides. hpa is labeled in green, gly is labeled in red.

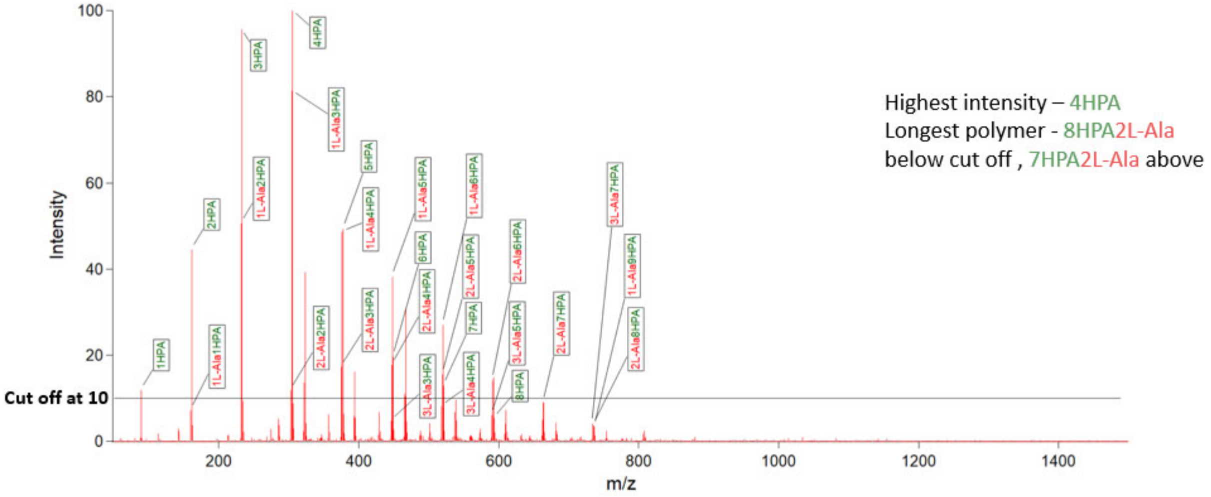

Supplementary Figure S20. ESI-MS spectrum of a dry-down reaction of samples A20 – Hydroxypropionic acid and L-alanine. Hydroxypropionic acid (hpa) and L-alanine (ala) were dried down at a 5:1 molar ratio, in favor of hpa, for 7 days at 85 °C. Dry down products were then resuspended in an aqueous solution of 20% acetonitrile in water (v/v). The resulting products were analyzed by negative-mode ESI-MS, indicating a variety of depsipeptides. hpa is labeled in green, ala is labeled in red.

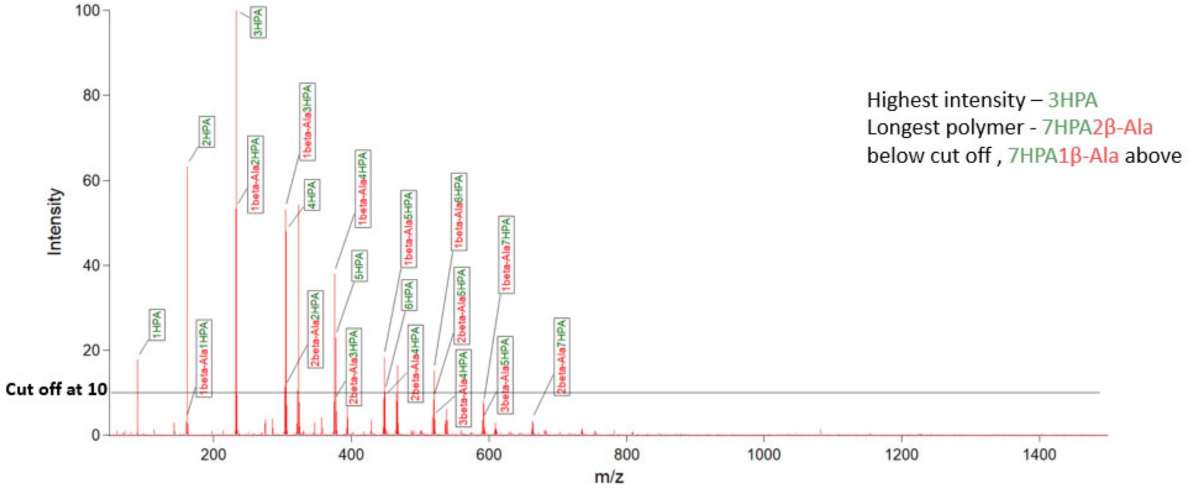

Supplementary Figure S21. ESI-MS spectrum of a dry-down reaction of samples A21 – Hydroxypropionic acid and β-alanine. Hydroxypropionic acid (hpa) and β-alanine (β-ala) were dried down at a 5:1 molar ratio, in favor of hpa, for 7 days at 85 °C. Dry down products were then resuspended in an aqueous solution of 20% acetonitrile in water (v/v). The resulting products were analyzed by negative-mode ESI-MS, indicating a variety of depsipeptides. hpa is labeled in green, β-ala is labeled in red.

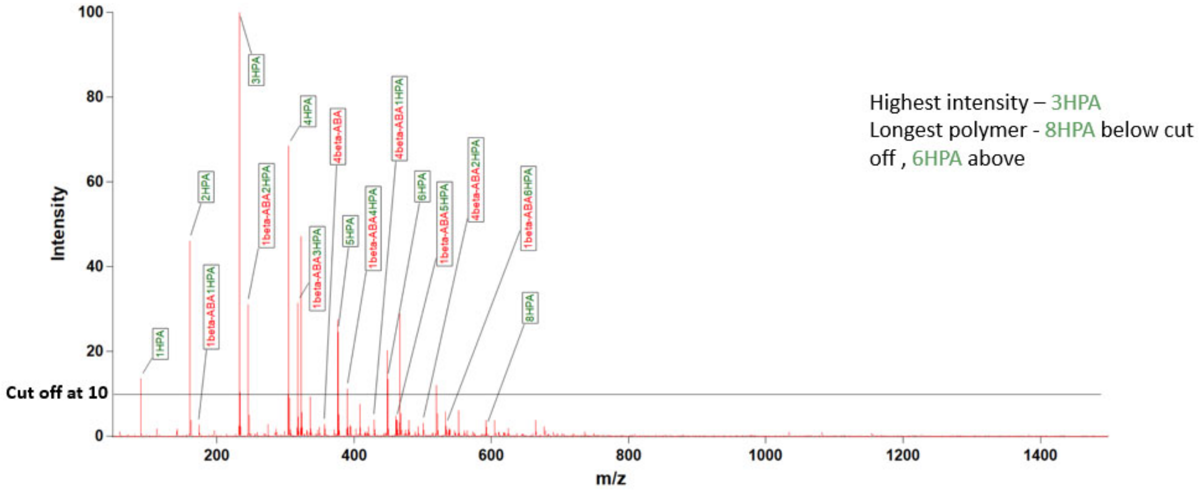

Supplementary Figure S22. ESI-MS spectrum of a dry-down reaction of samples A22 – Hydroxypropionic acid and β-aminobutyric acid. Hydroxypropionic acid (hpa) and β-aminobutyric acid (β-aba) were dried down at a 5:1 molar ratio, in favor of hpa, for 7 days at 85 °C. Dry down products were then resuspended in an aqueous solution of 20% acetonitrile in water (v/v). The resulting products were analyzed by negative-mode ESI-MS, indicating a variety of depsipeptides. hpa is labeled in green, β-aba is labeled in red.

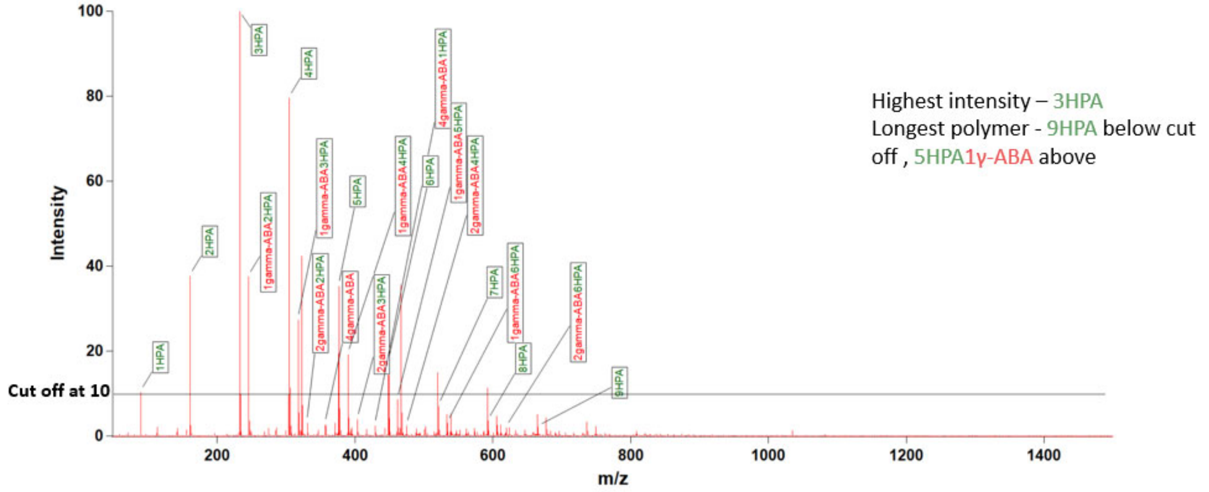

Supplementary Figure S23. ESI-MS spectrum of a dry-down reaction of samples A23 – Hydroxypropionic acid and γ-aminobutyric acid. Hydroxypropionic acid (hpa) and γ-aminobutyric acid (γ-aba) were dried down at a 5:1 molar ratio, in favor of hpa, for 7 days at 85 °C. Dry down products were then resuspended in an aqueous solution of 20% acetonitrile in water (v/v). The resulting products were analyzed by negative-mode ESI-MS, indicating a variety of depsipeptides. hpa is labeled in green, γ-aba is labeled in red.

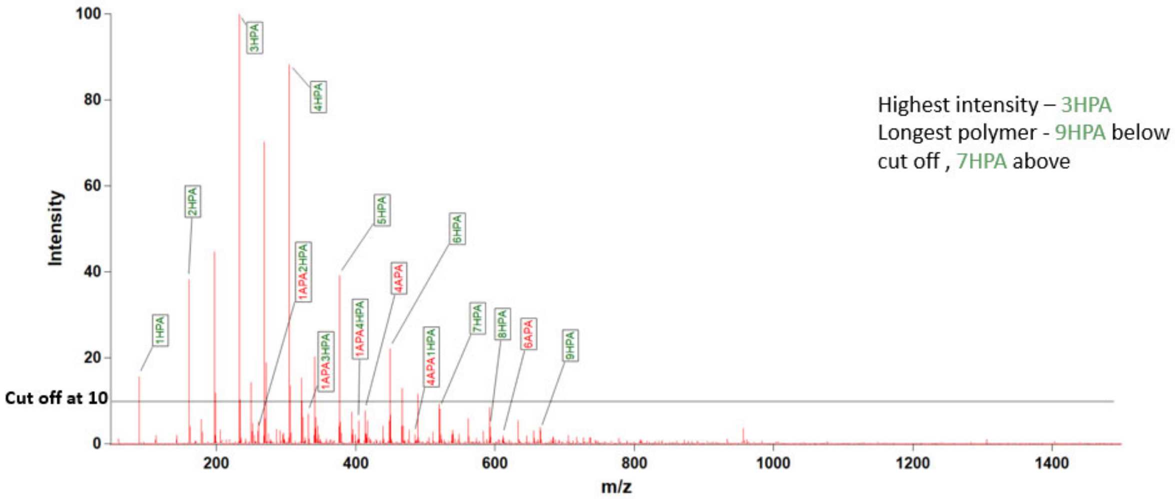

Supplementary Figure S24. ESI-MS spectrum of a dry-down reaction of samples A24 – Hydroxypropionic acid and 4-aminopentanoic acid. Hydroxypropionic acid (hpa) and 4-aminopentanoic acid (Apa) were dried down at a 5:1 molar ratio, in favor of hpa, for 7 days at 85 °C. Dry down products were then resuspended in an aqueous solution of 20% acetonitrile in water (v/v). The resulting products were analyzed by negative-mode ESI-MS, indicating a variety of depsipeptides. hpa is labeled in green, Apa is labeled in red.

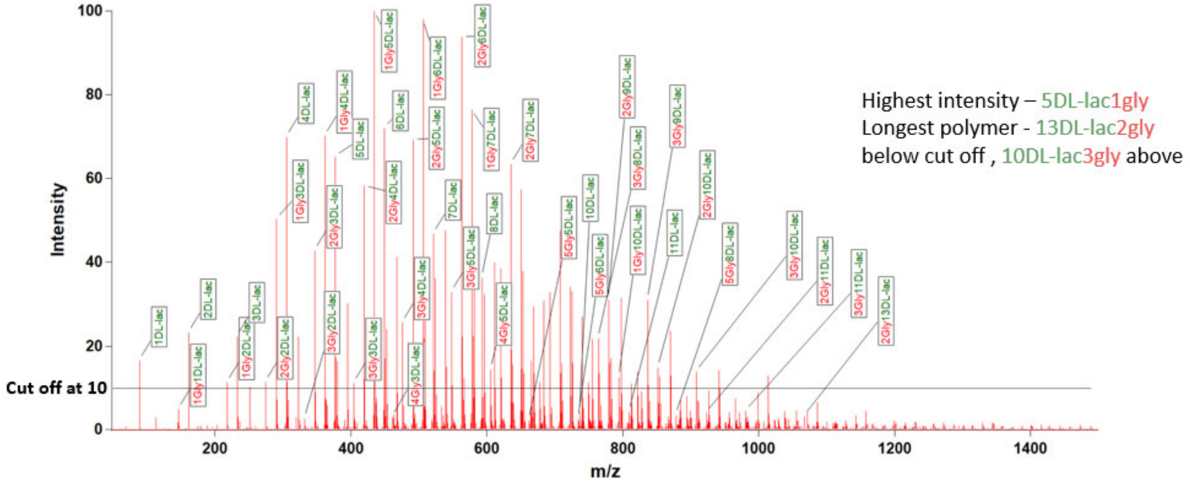

Supplementary Figure S25. ESI-MS spectrum of a dry-down reaction of samples A25 – DL-lactic acid and glycine. DL-lactic acid (DL-lac) and glycine (gly) were dried down at a 5:1 molar ratio, in favor of DL-lac, for 7 days at 85 °C. Dry down products were then resuspended in an aqueous solution of 20% acetonitrile in water (v/v). The resulting products were analyzed by negative-mode ESI-MS, indicating a variety of depsipeptides. DL-lac is labeled in green, gly is labeled in red.

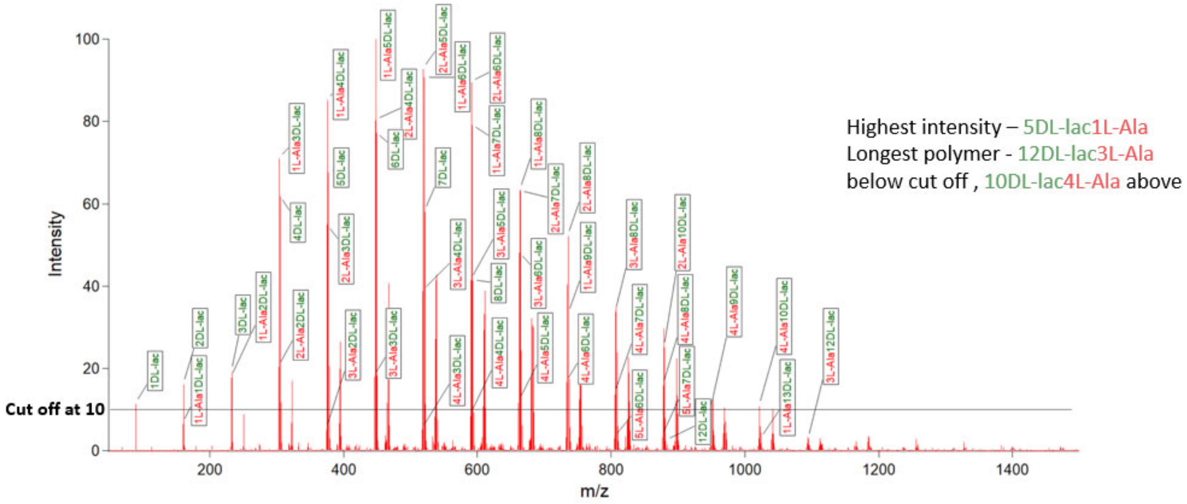

Supplementary Figure S26. ESI-MS spectrum of a dry-down reaction of samples A26 – DL-lactic acid and L-alanine. DL-lactic acid (DL-lac) and L-alanine (ala) were dried down at a 5:1 molar ratio, in favor of DL-lac, for 7 days at 85 °C. Dry down products were then resuspended in an aqueous solution of 20% acetonitrile in water (v/v). The resulting products were analyzed by negative-mode ESI-MS, indicating a variety of depsipeptides. DL-lac is labeled in green, ala is labeled in red.

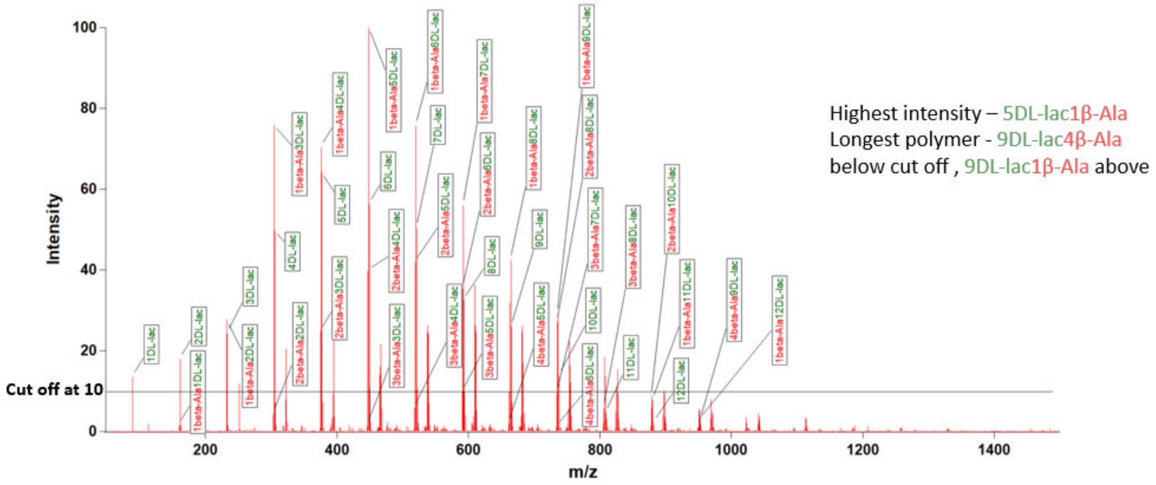

Supplementary Figure S27. ESI-MS spectrum of a dry-down reaction of samples A27 – DL-lactic acid and β-alanine. DL-lactic acid (DL-lac) and β-alanine (β-ala) were dried down at a 5:1 molar ratio, in favor of DL-lac, for 7 days at 85 °C. Dry down products were then resuspended in an aqueous solution of 20% acetonitrile in water (v/v). The resulting products were analyzed by negative-mode ESI-MS, indicating a variety of depsipeptides. DL-lac is labeled in green, β-ala is labeled in red.

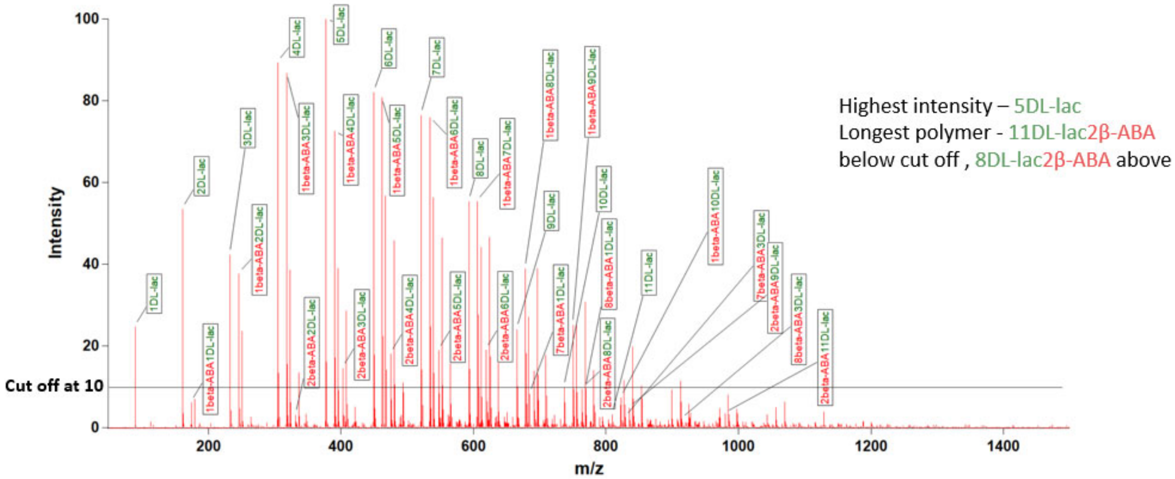

Supplementary Figure S28. ESI-MS spectrum of a dry-down reaction of samples A28 – DL-lactic acid and β-aminobutyric acid. DL-lactic acid (DL-lac) and β-aminobutyric acid (β-aba) were dried down at a 5:1 molar ratio, in favor of DL-lac, for 7 days at 85 °C. Dry down products were then resuspended in an aqueous solution of 20% acetonitrile in water (v/v). The resulting products were analyzed by negative-mode ESI-MS, indicating a variety of depsipeptides. DL-lac is labeled in green, β-aba is labeled in red.

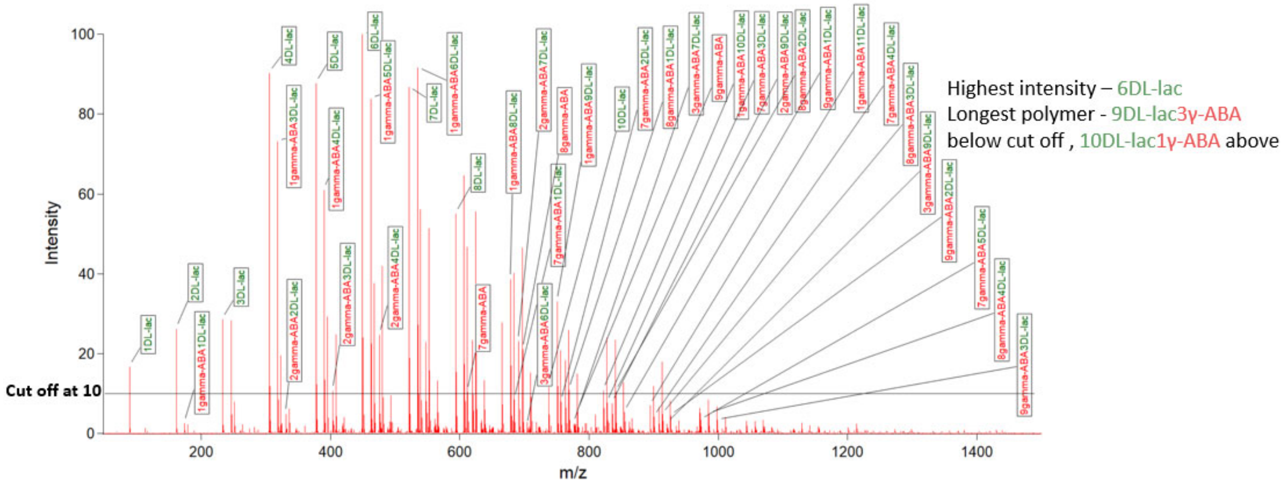

Supplementary Figure S29. ESI-MS spectrum of a dry-down reaction of samples A29 – DL-lactic acid and γ-aminobutyric acid. DL-lactic acid (DL-lac) and γ-aminobutyric acid (β-aba) were dried down at a 5:1 molar ratio, in favor of DL-lac, for 7 days at 85 °C. Dry down products were then resuspended in an aqueous solution of 20% acetonitrile in water (v/v). The resulting products were analyzed by negative-mode ESI-MS, indicating a variety of depsipeptides. DL-lac is labeled in green, γ-aba is labeled in red.

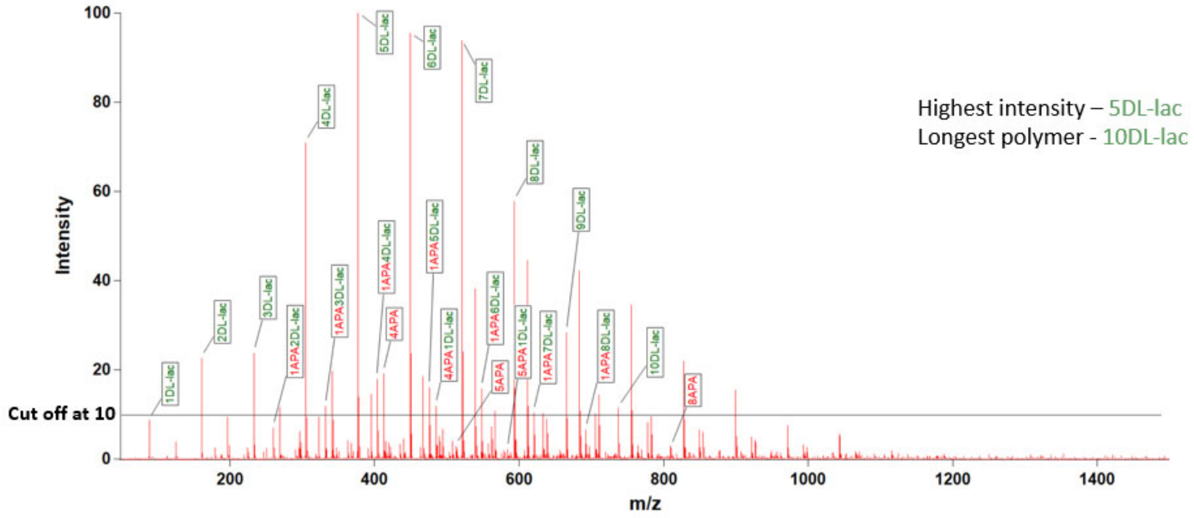

Supplementary Figure S30. ESI-MS spectrum of a dry-down reaction of samples A30 – DL-lactic acid and 4-aminopentanoic acid. DL-lactic acid (DL-lac) and 4-aminopentanoic acid (Apa) were dried down at a 5:1 molar ratio, in favor of DL-lac, for 7 days at 85 °C. Dry down products were then resuspended in an aqueous solution of 20% acetonitrile in water (v/v). The resulting products were analyzed by negative-mode ESI-MS, indicating a variety of depsipeptides. DL-lac is labeled in green, Apa is labeled in red

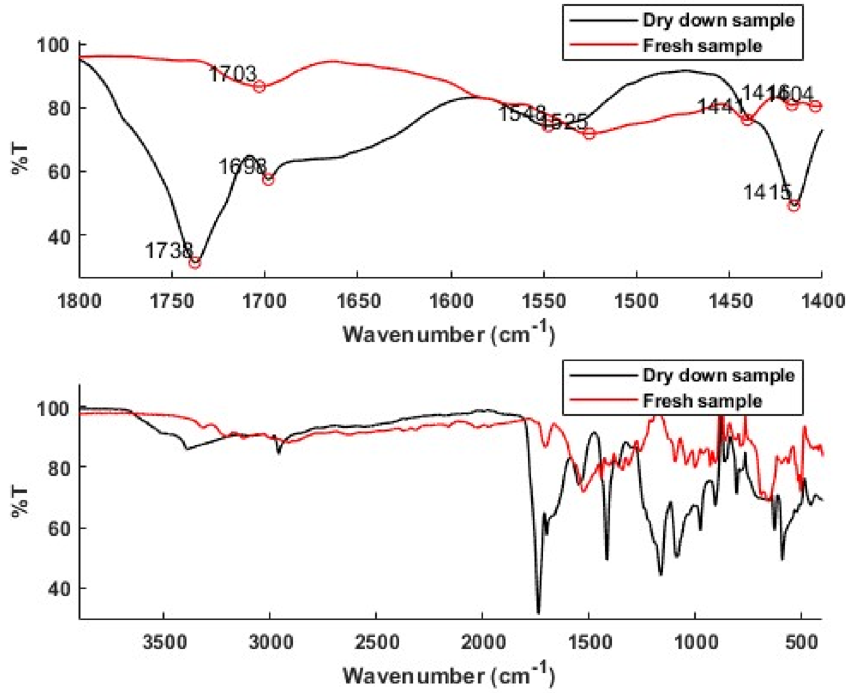

Supplementary Figure S31. FTIR spectrum of sample A1- glycolic acid and glycine. Glycolic acid (glc) and glycine (Gly) were dried down at a 5:1 molar ratio, in favor of glc, for 7 days at 85 °C (In black). Dry down products were then resuspended in an aqueous solution of 20% acetonitrile in water (v/v). Fresh monomer stock solution samples are in red.

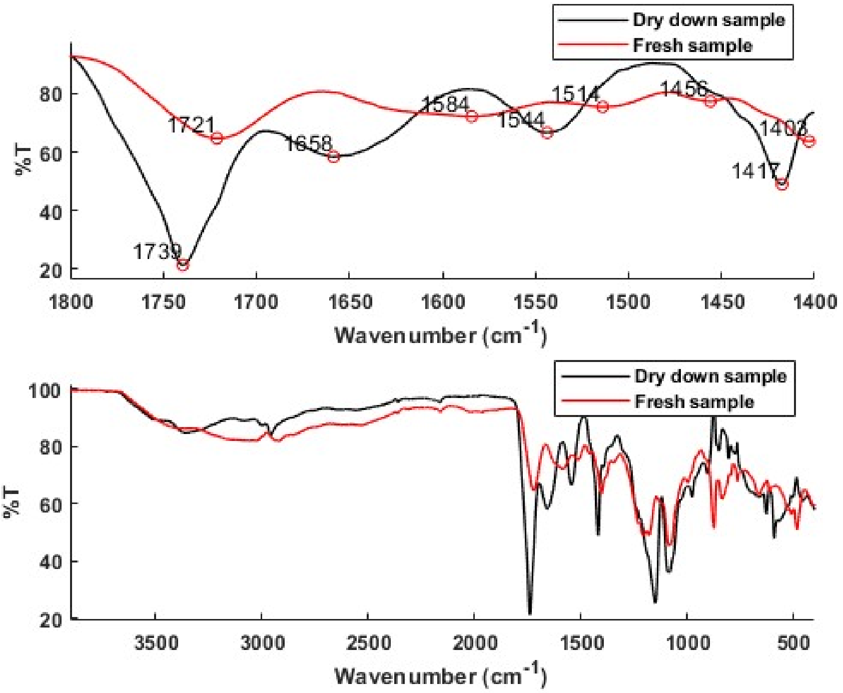

Supplementary Figure S32. FTIR spectrum of sample A2- glycolic acid and L-alanine. Glycolic acid (glc) and L-alanine (Ala) were dried down at a 5:1 molar ratio, in favor of glc, for 7 days at 85 °C (In black). Dry down products were then resuspended in an aqueous solution of 20% acetonitrile in water (v/v). Fresh monomer stock solution samples are in red.

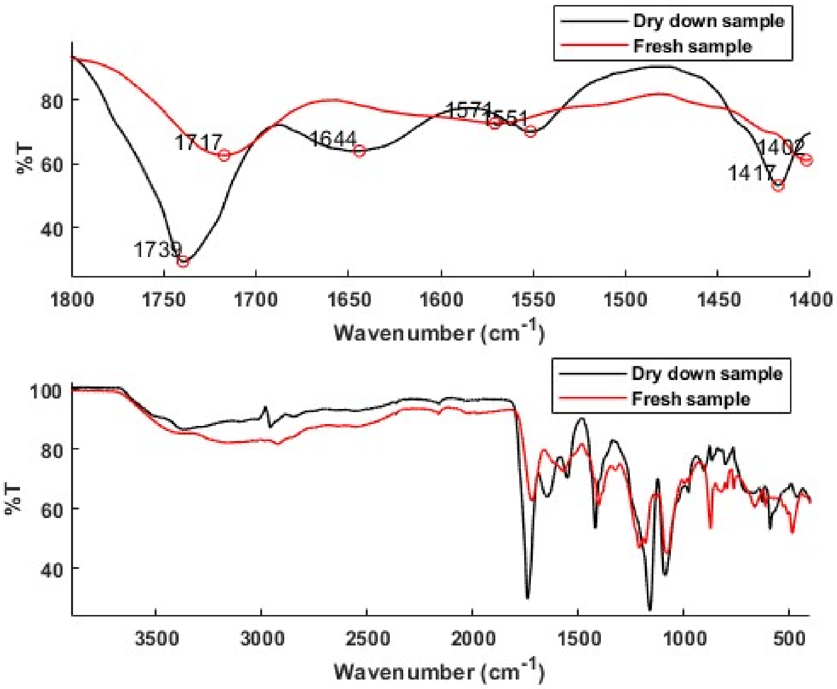

Supplementary Figure S33. FTIR spectrum of sample A3- glycolic acid and β-alanine. Glycolic acid (glc) and β-alanine (β-Ala) were dried down at a 5:1 molar ratio, in favor of glc, for 7 days at 85 °C (In black). Dry down products were then resuspended in an aqueous solution of 20% acetonitrile in water (v/v). Fresh monomer stock solution samples are in red.

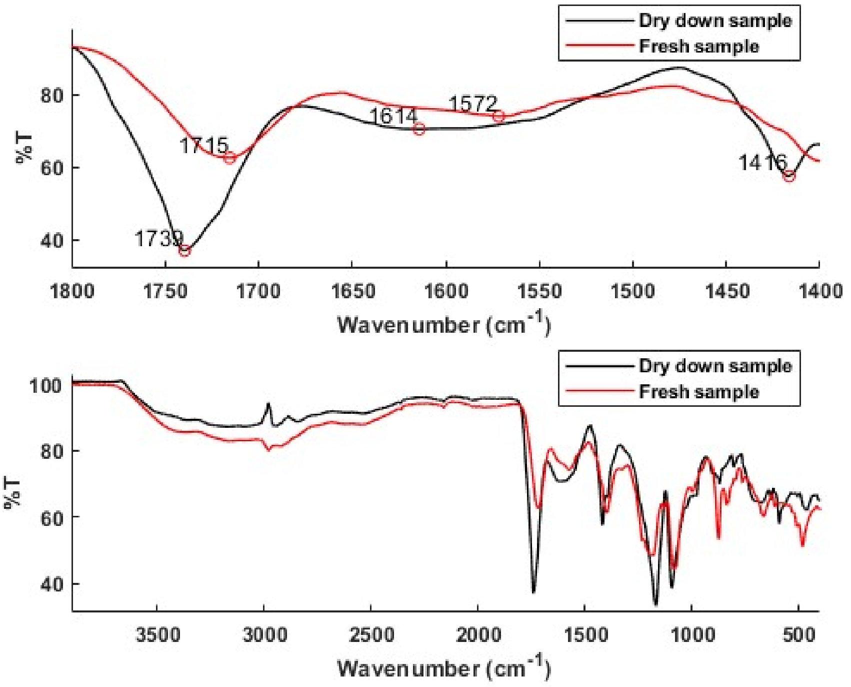

Supplementary Figure S34. FTIR spectrum of sample A4- glycolic acid and β-aminobutyric acid. Glycolic acid (glc) and β-aminobutyric acid (β-Aba) were dried down at a 5:1 molar ratio, in favor of glc, for 7 days at 85 °C (In black). Dry down products were then resuspended in an aqueous solution of 20% acetonitrile in water (v/v). Fresh monomer stock solution samples are in red.

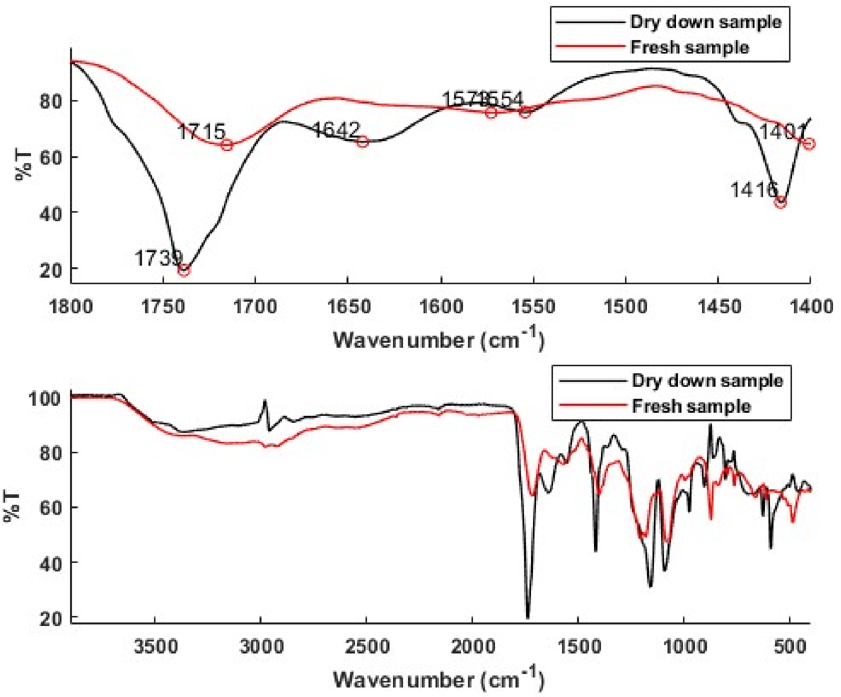

Supplementary Figure S35. FTIR spectrum of sample A5- glycolic acid and γ-aminobutyric acid. Glycolic acid (glc) and γ-aminobutyric acid (γ -Aba) were dried down at a 5:1 molar ratio, in favor of glc, for 7 days at 85 °C (In black). Dry down products were then resuspended in an aqueous solution of 20% acetonitrile in water (v/v). Fresh monomer stock solution samples are in red.

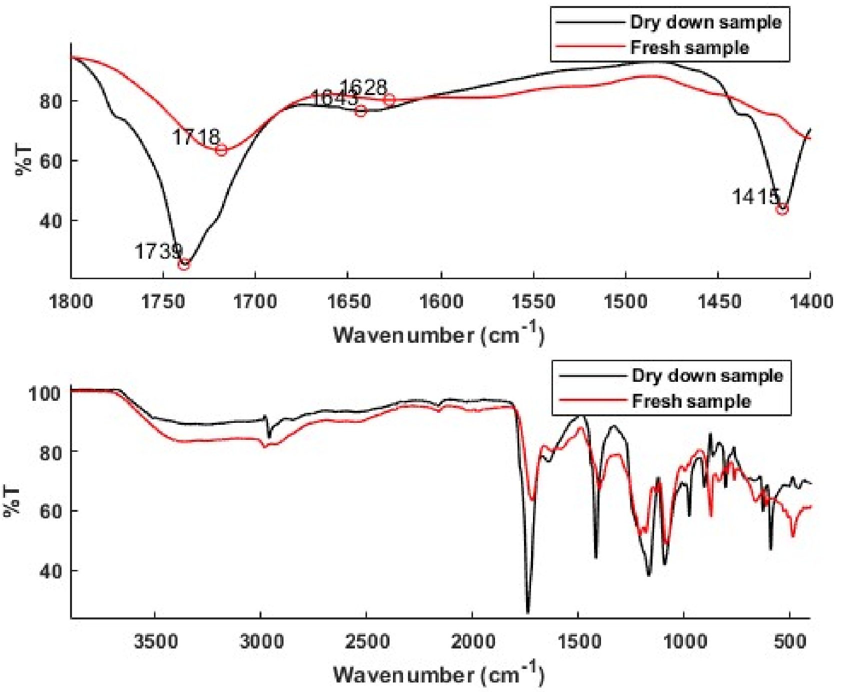

Supplementary Figure S36. FTIR spectrum of sample A6- glycolic acid and 4-aminopentanoic acid. Glycolic acid (glc) and 4-aminopentanoic acid (γ -Apa) were dried down at a 5:1 molar ratio, in favor of glc, for 7 days at 85 °C (In black). Dry down products were then resuspended in an aqueous solution of 20% acetonitrile in water (v/v). Fresh monomer stock solution samples are in red.

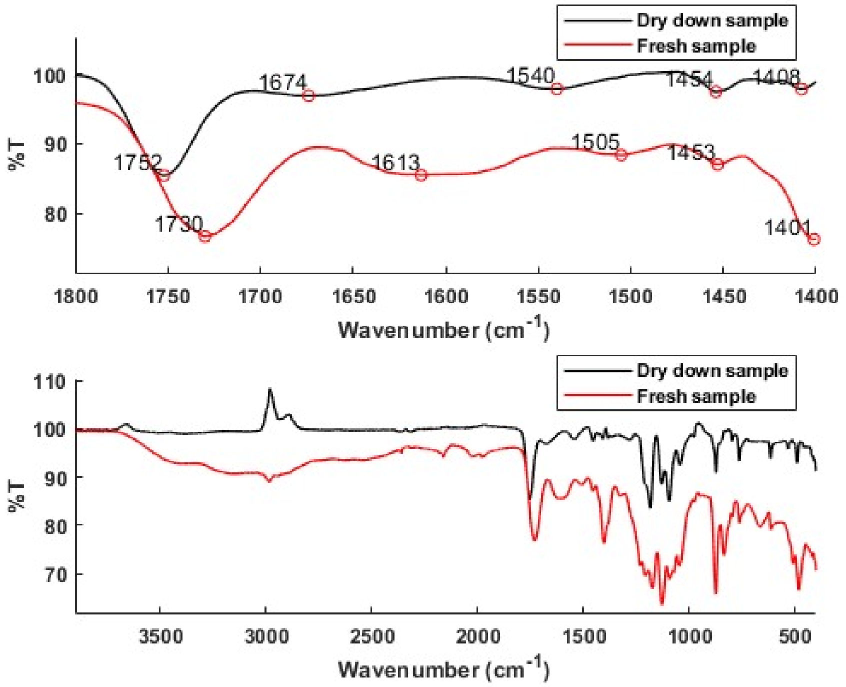

Supplementary Figure S37. FTIR spectrum of sample A7- L-lactic acid and glycine. L-lactic acid (lac) and glycine (Gly) were dried down at a 5:1 molar ratio, in favor of lac, for 7 days at 85 °C (In black). Dry down products were then resuspended in an aqueous solution of 20% acetonitrile in water (v/v). Fresh monomer stock solution samples are in red.

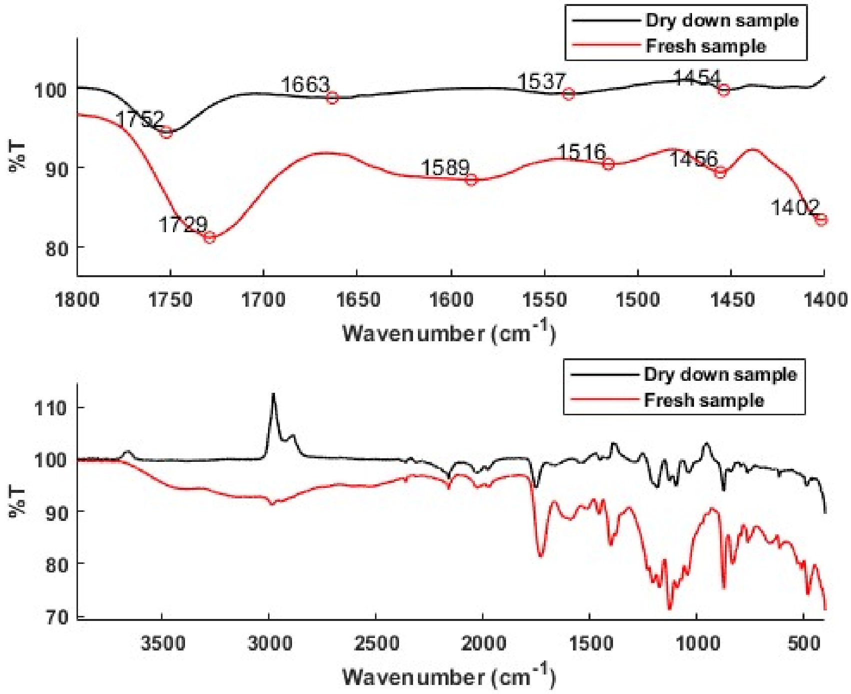

Supplementary Figure S38. FTIR spectrum of sample A8- L-lactic acid and L-alanine. L-lactic acid (lac) and L-alanine (Ala) were dried down at a 5:1 molar ratio, in favor of lac, for 7 days at 85 °C (In black). Dry down products were then resuspended in an aqueous solution of 20% acetonitrile in water (v/v). Fresh monomer stock solution samples are in red.

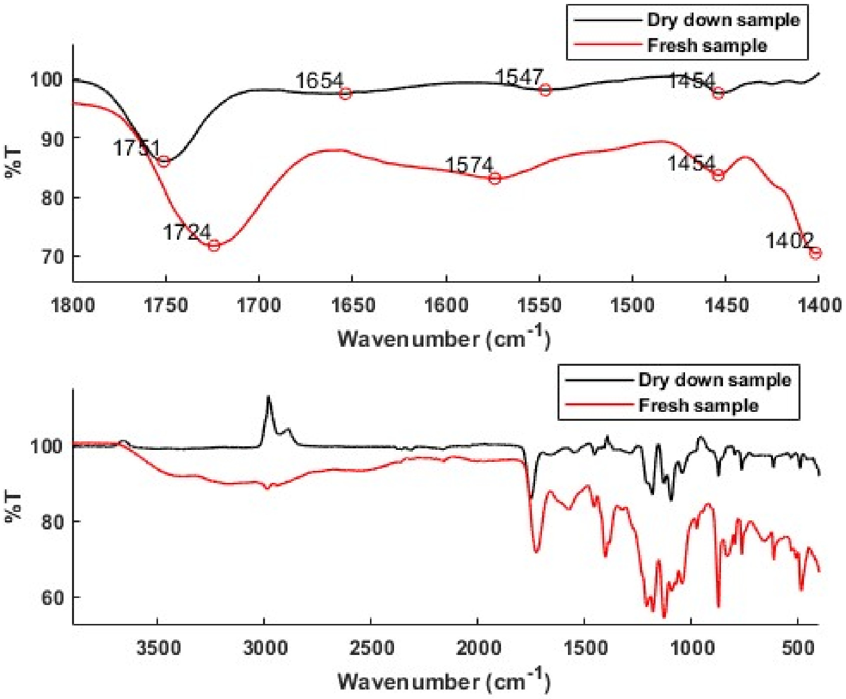

Supplementary Figure S39. FTIR spectrum of sample A9- L-lactic acid and β-alanine. L-lactic acid (lac) and β-alanine (β-Ala) were dried down at a 5:1 molar ratio, in favor of lac, for 7 days at 85 °C (In black). Dry down products were then resuspended in an aqueous solution of 20% acetonitrile in water (v/v). Fresh monomer stock solution samples are in red.

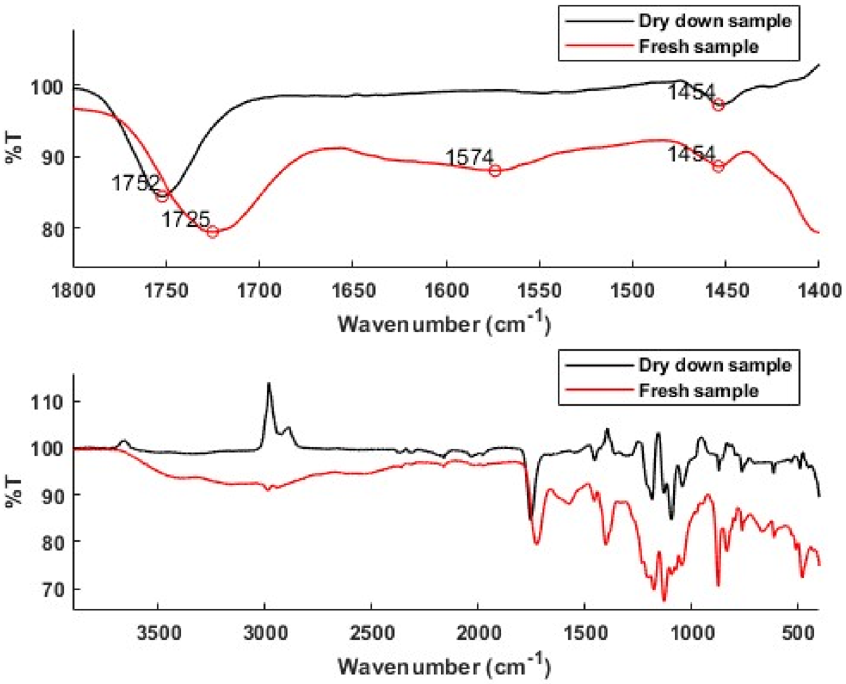

Supplementary Figure S40. FTIR spectrum of sample A10- L-lactic acid and β-aminobutyric acid. L-lactic acid (lac) and β-aminobutyric acid (β-Aba) were dried down at a 5:1 molar ratio, in favor of lac, for 7 days at 85 °C (In black). Dry down products were then resuspended in an aqueous solution of 20% acetonitrile in water (v/v). Fresh monomer stock solution samples are in red.

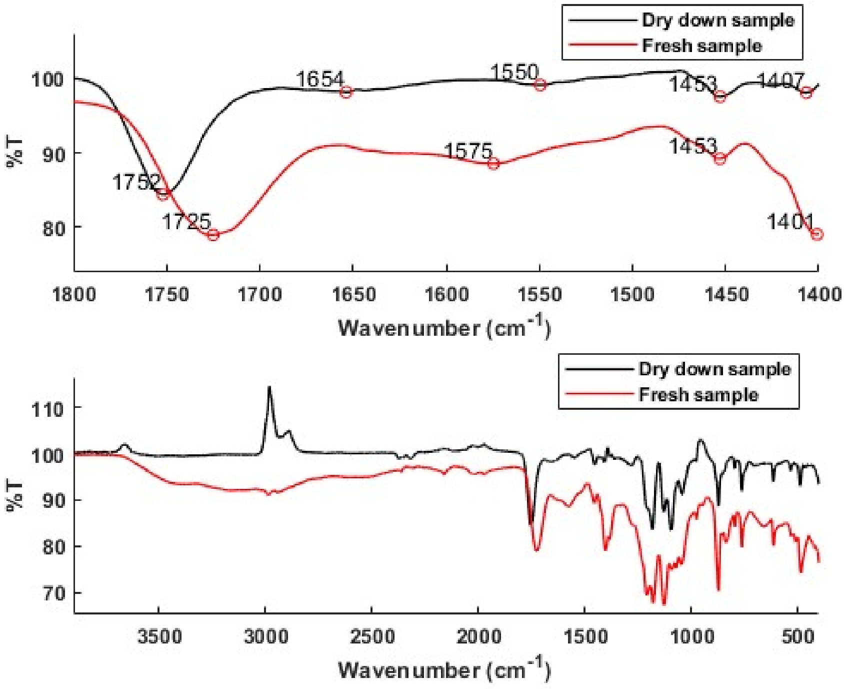

Supplementary Figure S41. FTIR spectrum of sample A11- L-lactic acid and γ-aminobutyric acid. L-lactic acid (lac) and γ-aminobutyric acid (γ -Aba) were dried down at a 5:1 molar ratio, in favor of lac, for 7 days at 85 °C (In black). Dry down products were then resuspended in an aqueous solution of 20% acetonitrile in water (v/v). Fresh monomer stock solution samples are in red.

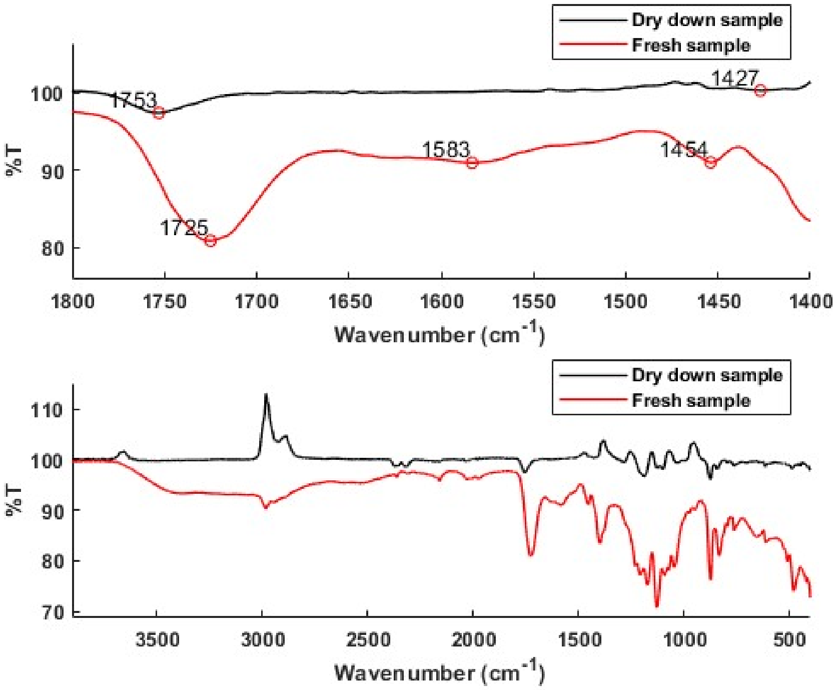

Supplementary Figure S42. FTIR spectrum of sample A12- L-lactic acid and 4-aminopentanoic acid. L-lactic acid (lac) and 4-aminopentanoic acid (γ -Apa) were dried down at a 5:1 molar ratio, in favor of lac, for 7 days at 85 °C (In black). Dry down products were then resuspended in an aqueous solution of 20% acetonitrile in water (v/v). Fresh monomer stock solution samples are in red.

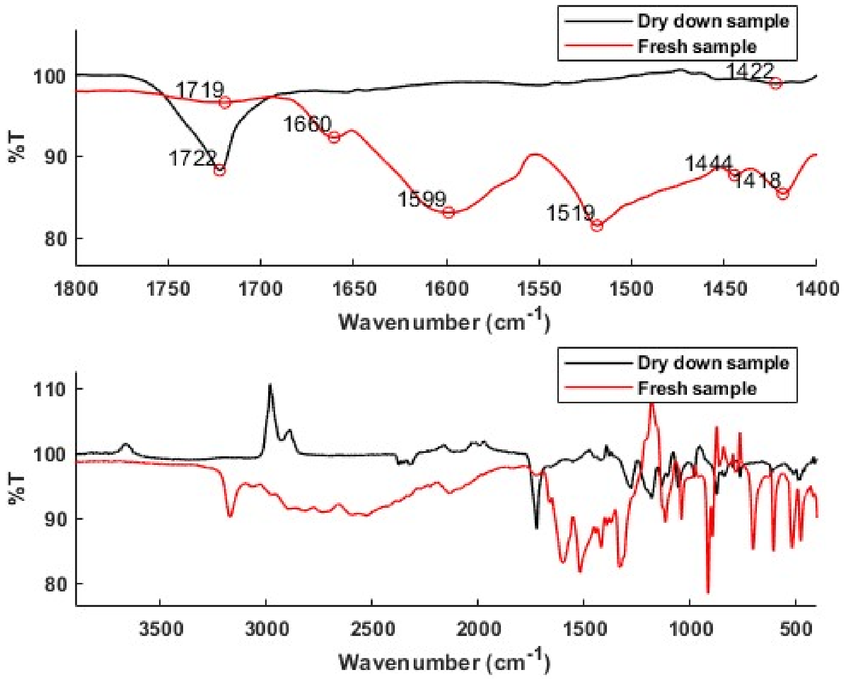

Supplementary Figure S43. FTIR spectrum of sample A13- Hydroxybutyric acid and glycine. Hydroxybutyric acid (hba) and glycine (Gly) were dried down at a 5:1 molar ratio, in favor of hba, for 7 days at 85 °C (In black). Dry down products were then resuspended in an aqueous solution of 20% acetonitrile in water (v/v). Fresh monomer stock solution samples are in red.

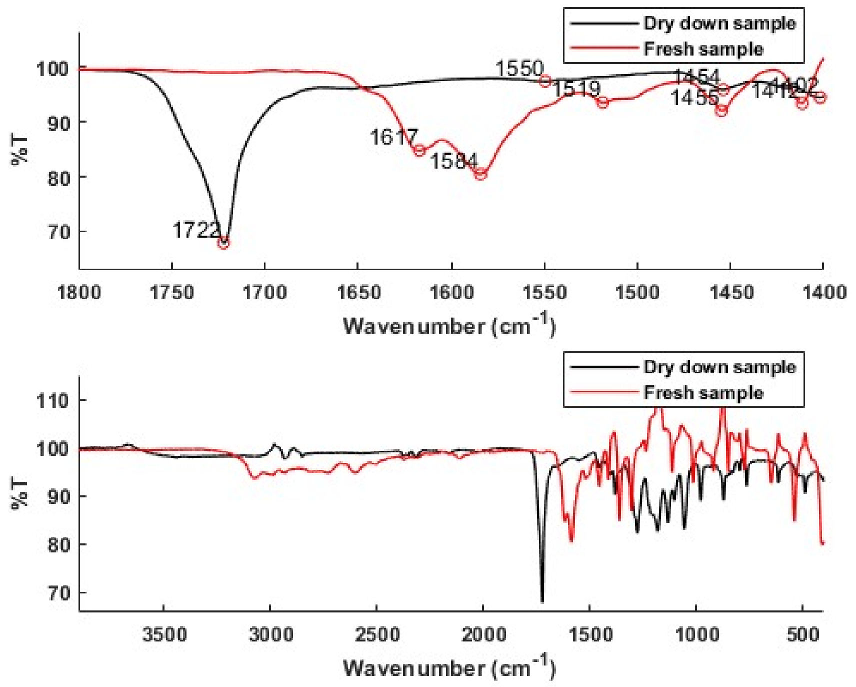

Supplementary Figure S44. FTIR spectrum of sample A14- Hydroxybutyric acid and L-alanine. Hydroxybutyric acid (hba) and L-alanine (Ala) were dried down at a 5:1 molar ratio, in favor of hba, for 7 days at 85 °C (In black). Dry down products were then resuspended in an aqueous solution of 20% acetonitrile in water (v/v). Fresh monomer stock solution samples are in red.

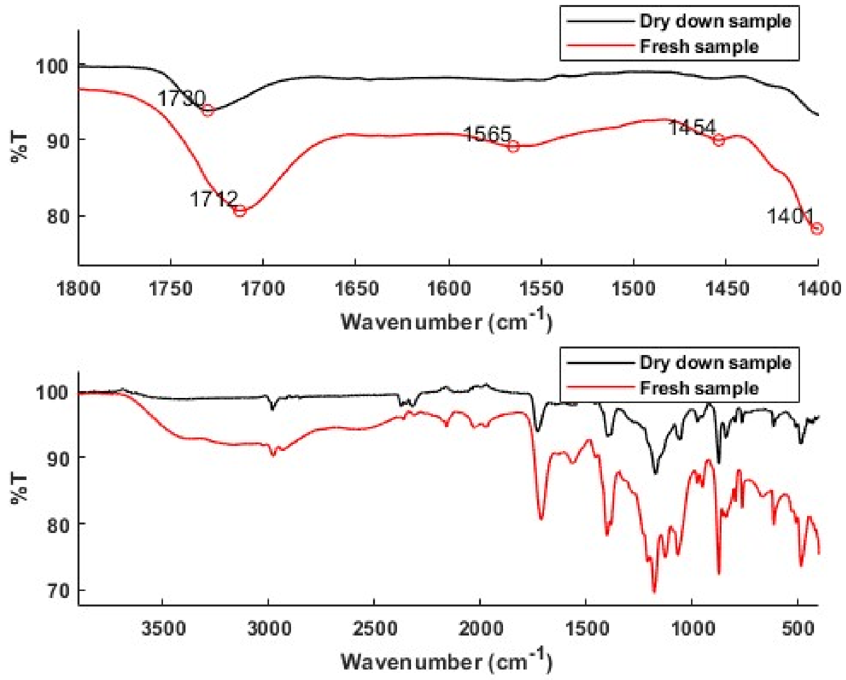

Supplementary Figure S45. FTIR spectrum of sample A15- Hydroxybutyric acid and β-alanine. Hydroxybutyric acid (hba) and β-alanine (β-Ala) were dried down at a 5:1 molar ratio, in favor of hba, for 7 days at 85 °C (In black). Dry down products were then resuspended in an aqueous solution of 20% acetonitrile in water (v/v). Fresh monomer stock solution samples are in red.

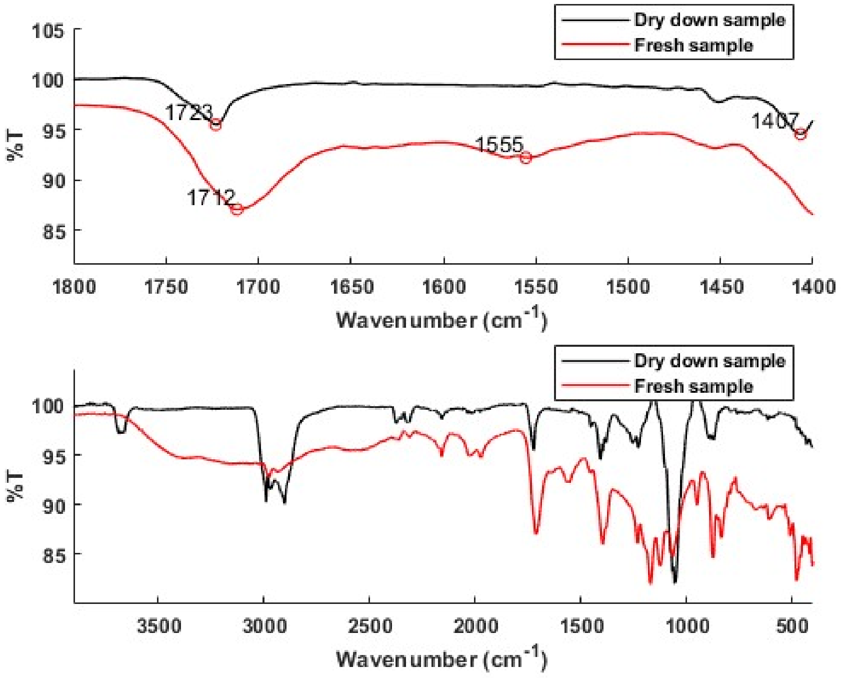

Supplementary Figure S46. FTIR spectrum of sample A16- Hydroxybutyric acid and β-aminobutyric acid. Hydroxybutyric acid (hba) and β-aminobutyric (β-Aɜa) were dried down at a 5:1 molar ratio, in favor of hba, for 7 days at 85 °C (In black). Dry down products were then resuspended in an aqueous solution of 20% acetonitrile in water (v/v). Fresh monomer stock solution samples are in red.

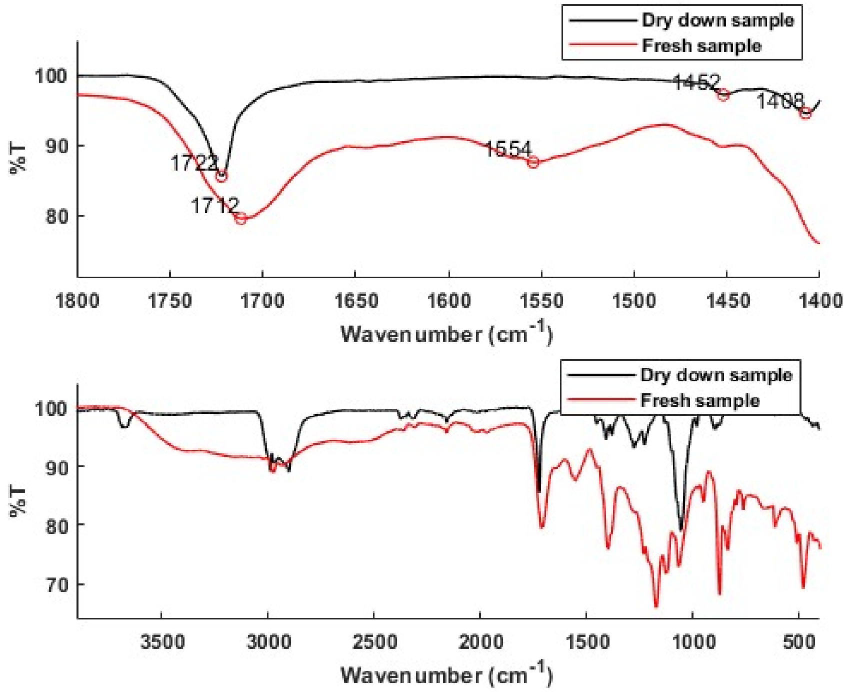

Supplementary Figure S47. FTIR spectrum of sample A17- Hydroxybutyric acid and γ-aminobutyric acid. Hydroxybutyric acid (hba) and γ-aminobutyric (γ -Aba) were dried down at a 5:1 molar ratio, in favor of hba, for 7 days at 85 °C (In black). Dry down products were then resuspended in an aqueous solution of 20% acetonitrile in water (v/v). Fresh monomer stock solution samples are in red.

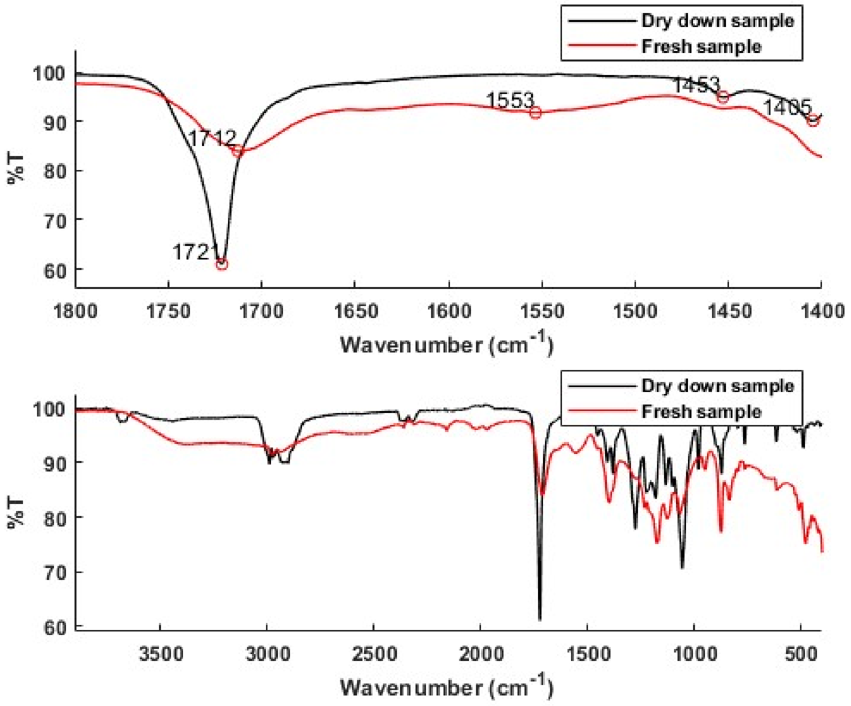

Supplementary Figure S48. FTIR spectrum of sample A18- Hydroxybutyric acid and 4-aminopentanoic acid. Hydroxybutyric acid (hba) and 4-aminopentanoic (γ-Apa) were dried down at a 5:1 molar ratio, in favor of hba, for 7 days at 85 °C (In black). Dry down products were then resuspended in an aqueous solution of 20% acetonitrile in water (v/v). Fresh monomer stock solution samples are in red.

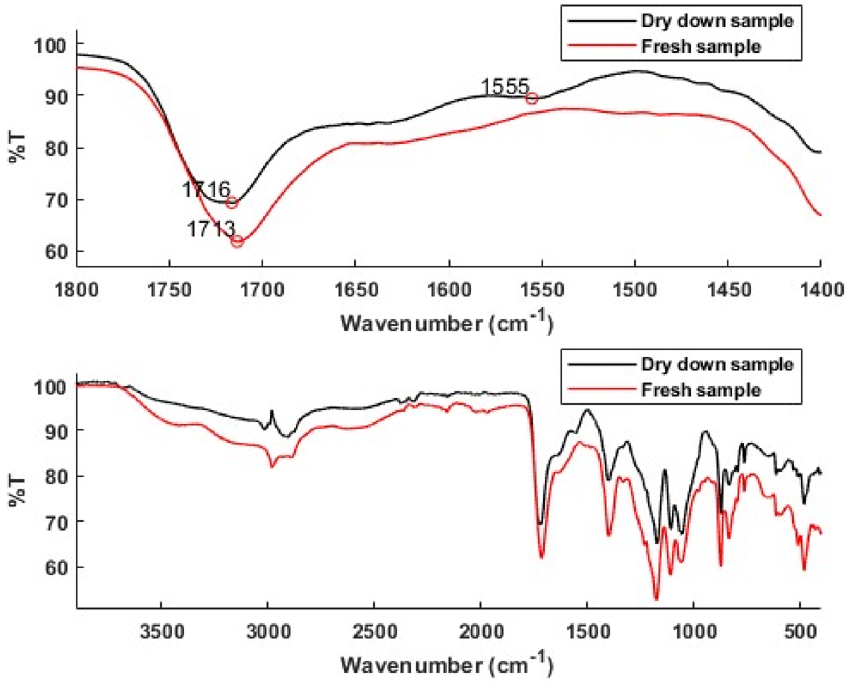

Supplementary Figure S49. FTIR spectrum of sample A19-Hydroxypropionic acid and glycine. Hydroxypropionic acid (hpa) and Glycine (Gly) were dried down at a 5:1 molar ratio, in favor of hpa, for 7 days at 85 °C (In black). Dry down products were then resuspended in an aqueous solution of 20% acetonitrile in water (v/v). Fresh monomer stock solution samples are in red.

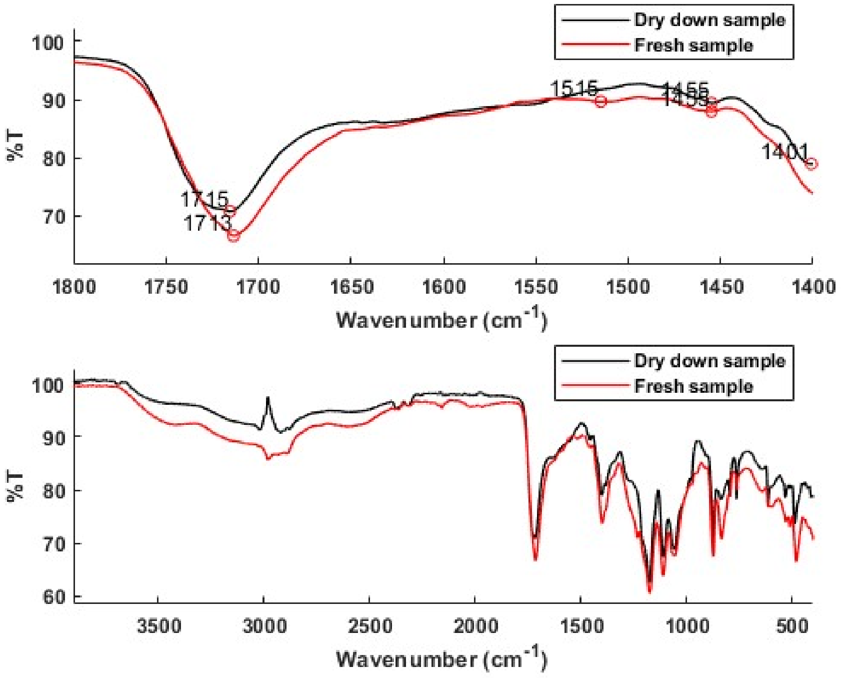

Supplementary Figure S50. FTIR spectrum of sample A20-Hydroxypropionic acid and L-alanine. Hydroxypropionic acid (hpa) and L-alanine (Ala) were dried down at a 5:1 molar ratio, in favor of hpa, for 7 days at 85 °C (In black). Dry down products were then resuspended in an aqueous solution of 20% acetonitrile in water (v/v). Fresh monomer stock solution samples are in red.

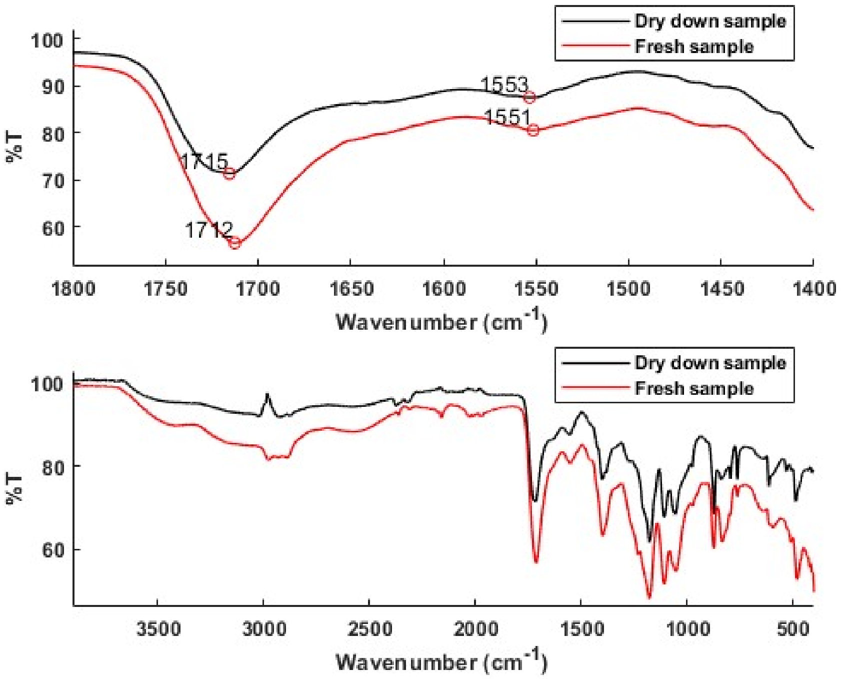

Supplementary Figure S51. FTIR spectrum of sample A21-Hydroxypropionic acid and β-alanine. Hydroxypropionic acid (hpa) and β-Alanine (β-Ala) were dried down at a 5:1 molar ratio, in favor of hpa, for 7 days at 85 °C (In black). Dry down products were then resuspended in an aqueous solution of 20% acetonitrile in water (v/v). Fresh monomer stock solution samples are in red.

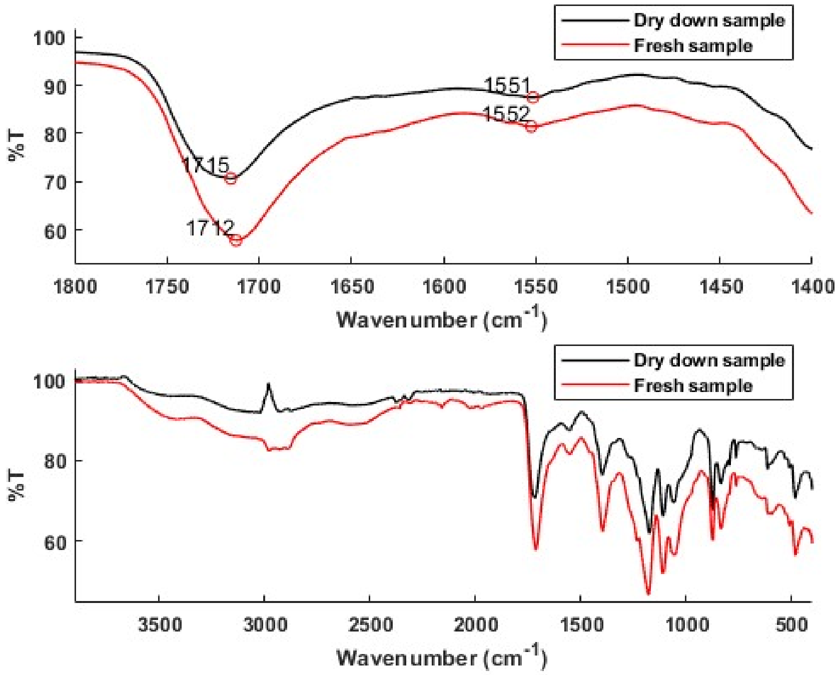

Supplementary Figure S52. FTIR spectrum of sample A22- Hydroxypropionic acid and β-aminobutyric acid. Hydroxypropionic acid (hpa) and β-aminobutyric acid (β-Aba) were dried down at a 5:1 molar ratio, in favor of hpa, for 7 days at 85 °C (In black). Dry down products were then resuspended in an aqueous solution of 20% acetonitrile in water (v/v). Fresh monomer stock solution samples are in red.

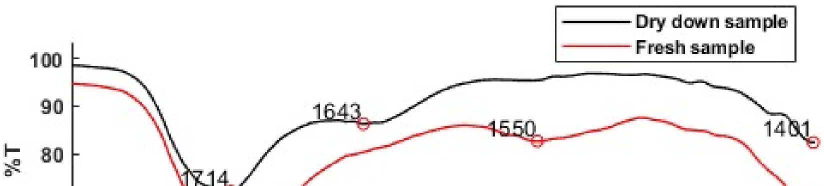

Supplementary Figure S53. FTIR spectrum of sample A23- Hydroxypropionic acid and γ-aminobutyric acid. Hydroxypropionic acid (hpa) and γ-aminobutyric acid (γ-Aba) were dried down at a 5:1 molar ratio, in favor of hpa, for 7 days at 85 °C (In black). Dry down products were then resuspended in an aqueous solution of 20% acetonitrile in water (v/v). Fresh monomer stock solution samples are in red

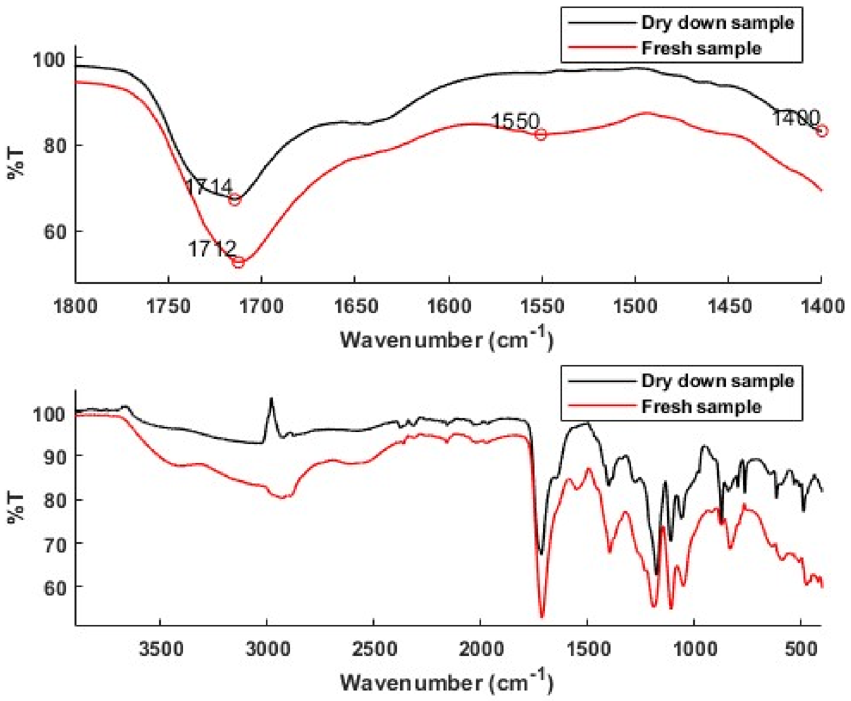

Supplementary Figure S54. FTIR spectrum of sample A24- Hydroxypropionic acid and 4-aminopentanoic acid. Hydroxypropionic acid (hpa) and 4-aminopentanoic acid (γ-Apa) were dried down at a 5:1 molar ratio, in favor of hpa, for 7 days at 85 °C (In black). Dry down products were then resuspended in an aqueous solution of 20% acetonitrile in water (v/v). Fresh monomer stock solution samples are in red

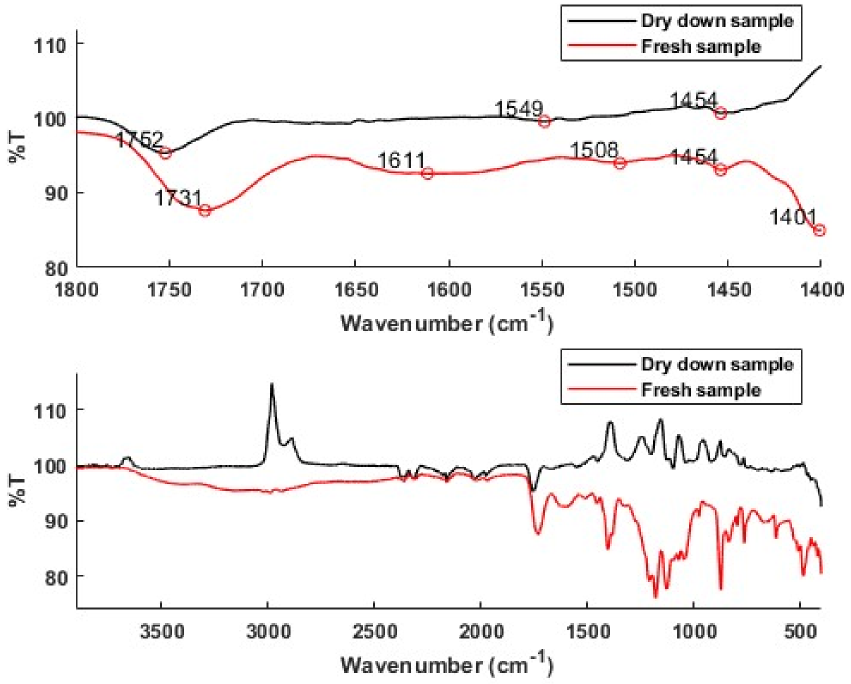

Supplementary Figure S55. FTIR spectrum of sample A25- DL-lactic acid and glycine. DL-lactic acid (DL-lac) and Glycine (Gly) were dried down at a 5:1 molar ratio, in favor of DL-lac, for 7 days at 85 °C (In black). Dry down products were then resuspended in an aqueous solution of 20% acetonitrile in water (v/v). Fresh monomer stock solution samples are in red

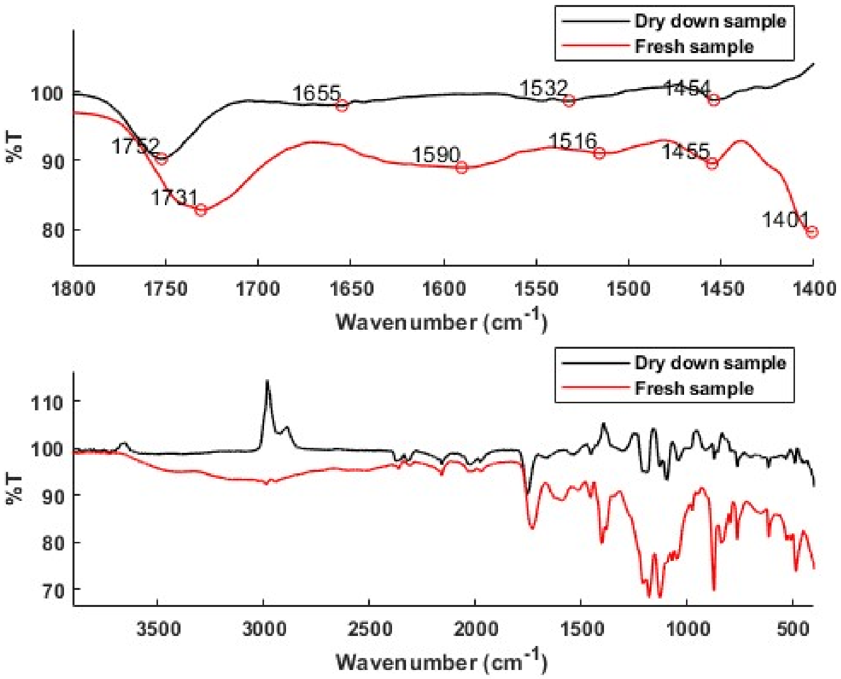

Supplementary Figure S56. FTIR spectrum of sample A26- DL-lactic acid and L-alanine. DL-lactic acid (DL-lac) and L-alanine (Ala) were dried down at a 5:1 molar ratio, in favor of DL-lac, for 7 days at 85 °C (In black). Dry down products were then resuspended in an aqueous solution of 20% acetonitrile in water (v/v). Fresh monomer stock solution samples are in red

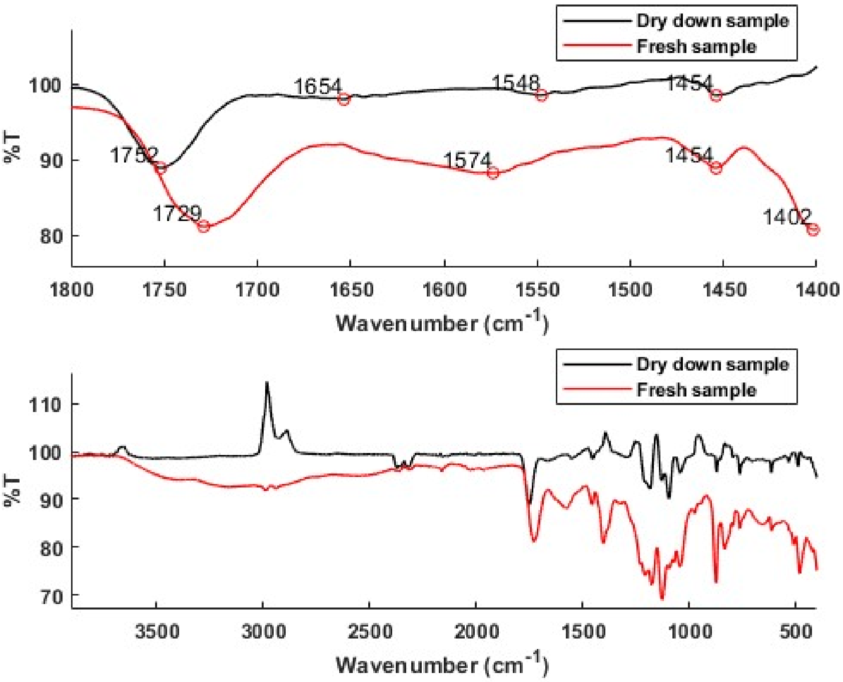

Supplementary Figure S57. FTIR spectrum of sample A27- DL-lactic acid and β-alanine. DL-lactic acid (DL-lac) and β-alanine (β-Ala) were dried down at a 5:1 molar ratio, in favor of DL-lac, for 7 days at 85 °C (In black). Dry down products were then resuspended in an aqueous solution of 20% acetonitrile in water (v/v). Fresh monomer stock solution samples are in red.

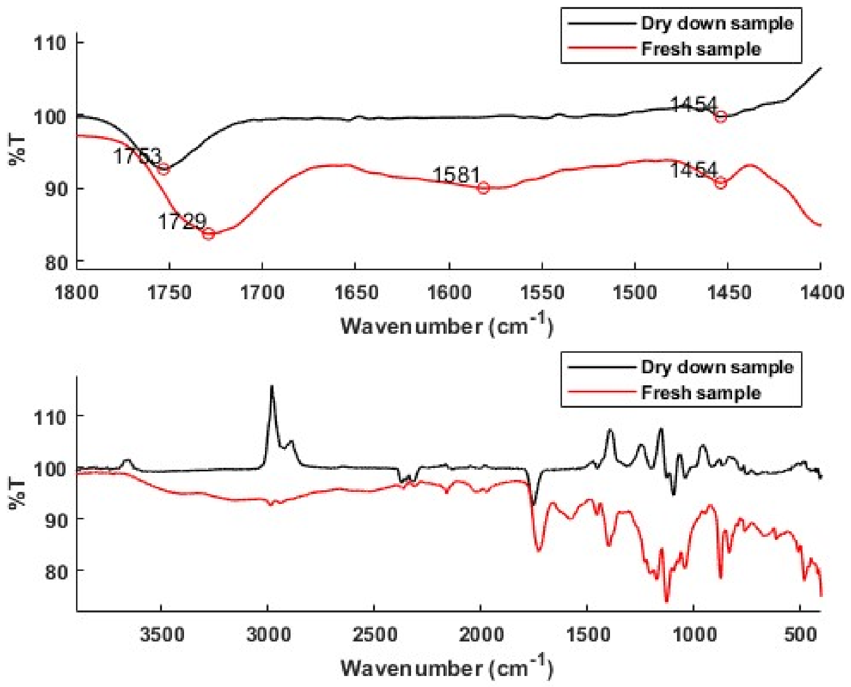

Supplementary Figure S58. FTIR spectrum of sample A28- DL-lactic acid and β-aminobutyric acid DL-lactic acid (DL-lac) and β-Aminobutyric acid (β-Aba) were dried down at a 5:1 molar ratio, in favor of DL-lac, for 7 days at 85 °C (In black). Dry down products were then resuspended in an aqueous solution of 20% acetonitrile in water (v/v). Fresh monomer stock solution samples are in red.

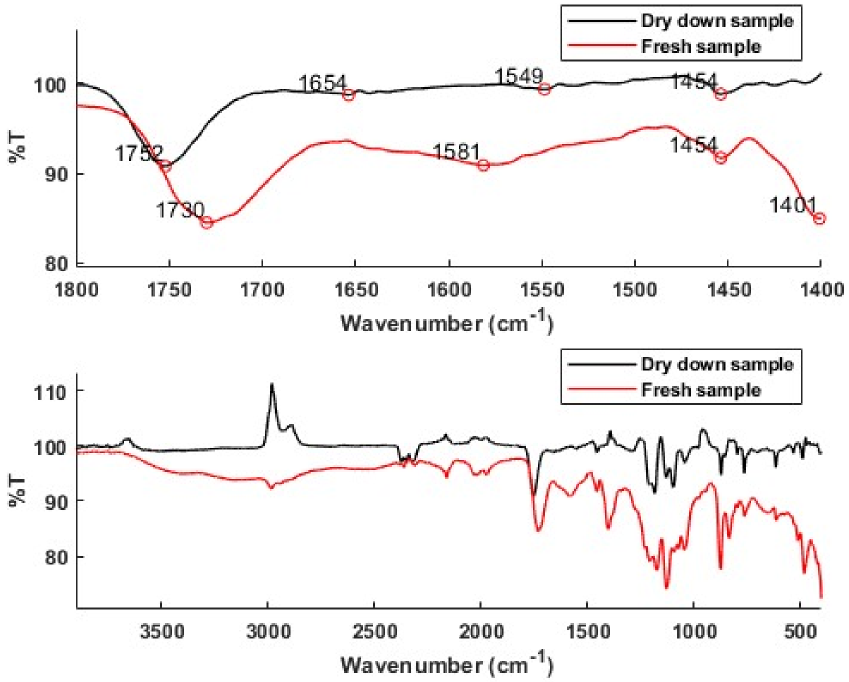

Supplementary Figure S59. FTIR spectrum of sample A29- DL-lactic acid and γ-aminobutyric acid. DL-lactic acid (DL-lac) and γ-Aminobutyric acid (γ-Aba) were dried down at a 5:1 molar ratio, in favor of DL-lac, for 7 days at 85 °C (In black). Dry down products were then resuspended in an aqueous solution of 20% acetonitrile in water (v/v). Fresh monomer stock solution samples are in red.

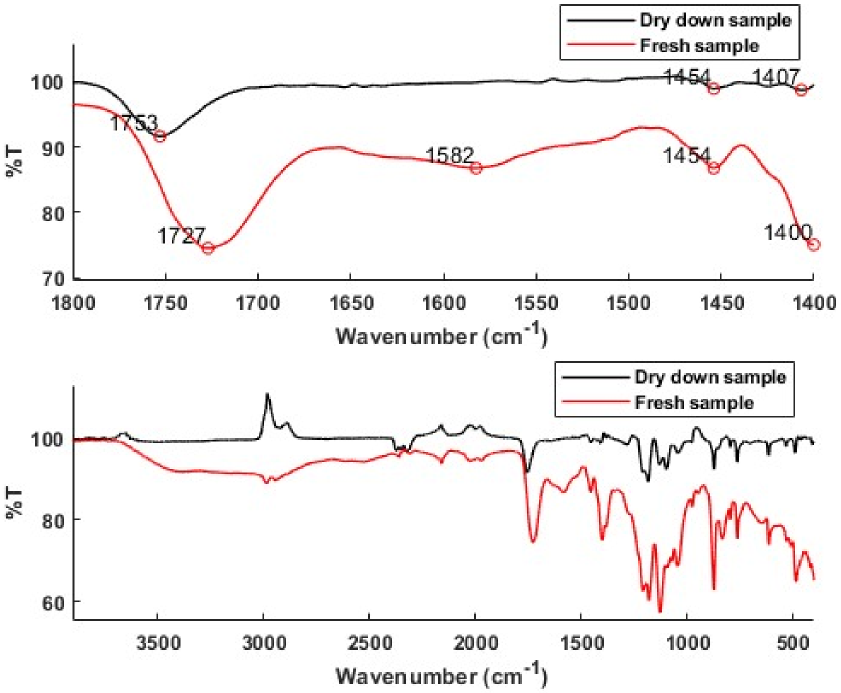

Supplementary Figure S60. FTIR spectrum of sample A30- DL-lactic acid and 4-aminopentanoic acid. DL-lactic acid (DL-lac) and 4-Aminopentanoic acid (γ-Apa) were dried down at a 5:1 molar ratio, in favor of DL-lac, for 7 days at 85 °C (In black). Dry down products were then resuspended in an aqueous solution of 20% acetonitrile in water (v/v). Fresh monomer stock solution samples are in red

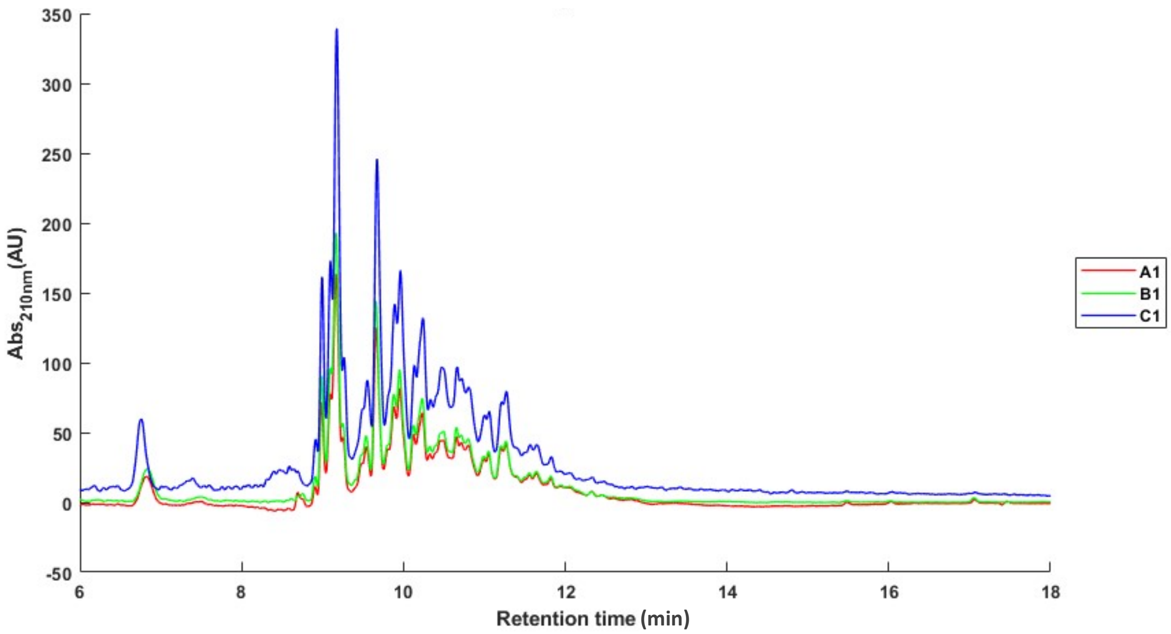

Supplementary Figure S61. HPLC overlay chromatogram of 3 repeats of glycolic acid and glycine reaction products. glycolic acid (glc) and glycine (Gly) were dried for 7 days in 85 °C, at a 5:1 molar ratio, in favor of glc. Dry down products were then resuspended in an aqueous solution of 20% acetonitrile in water (v/v). The resulting products were seperated on C18 column.

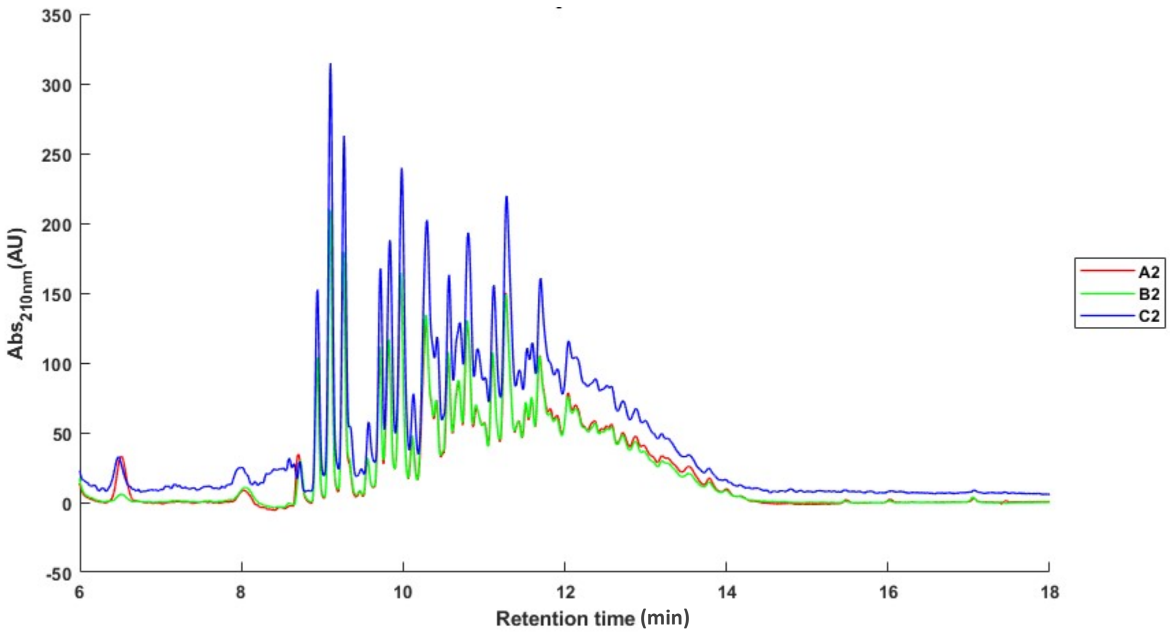

Supplementary Figure S62. HPLC overlay chromatogram of 3 repeats of glycolic acid and L-alanine reaction products. glycolic acid (glc) and L-alanine (Ala) were dried for 7 days in 85 °C, at a 5:1 molar ratio, in favor of glc. Dry down products were then resuspended in an aqueous solution of 20% acetonitrile in water (v/v). The resulting products were seperated on C18 column.

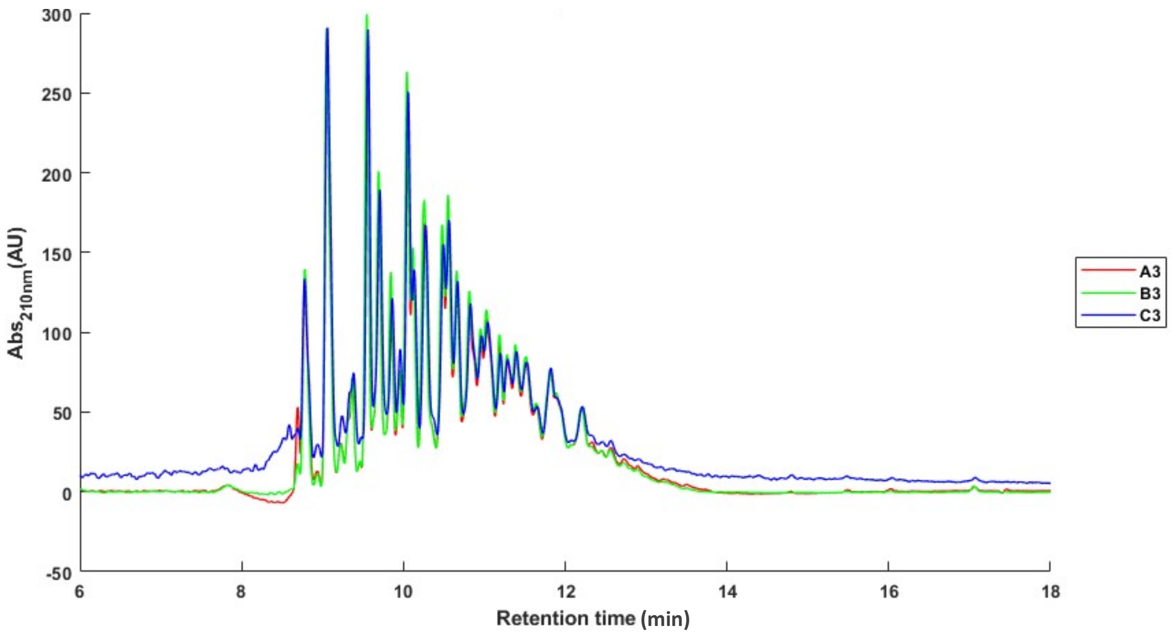

Supplementary Figure S63. HPLC overlay chromatogram of 3 repeats of glycolic acid and β-alanine reaction products. glycolic acid (glc) and β-alanine (β-Ala) were dried for 7 days in 85 °C, at a 5:1 molar ratio, in favor of glc. Dry down products were then resuspended in an aqueous solution of 20% acetonitrile in water (v/v). The resulting products were seperated on C18 column.

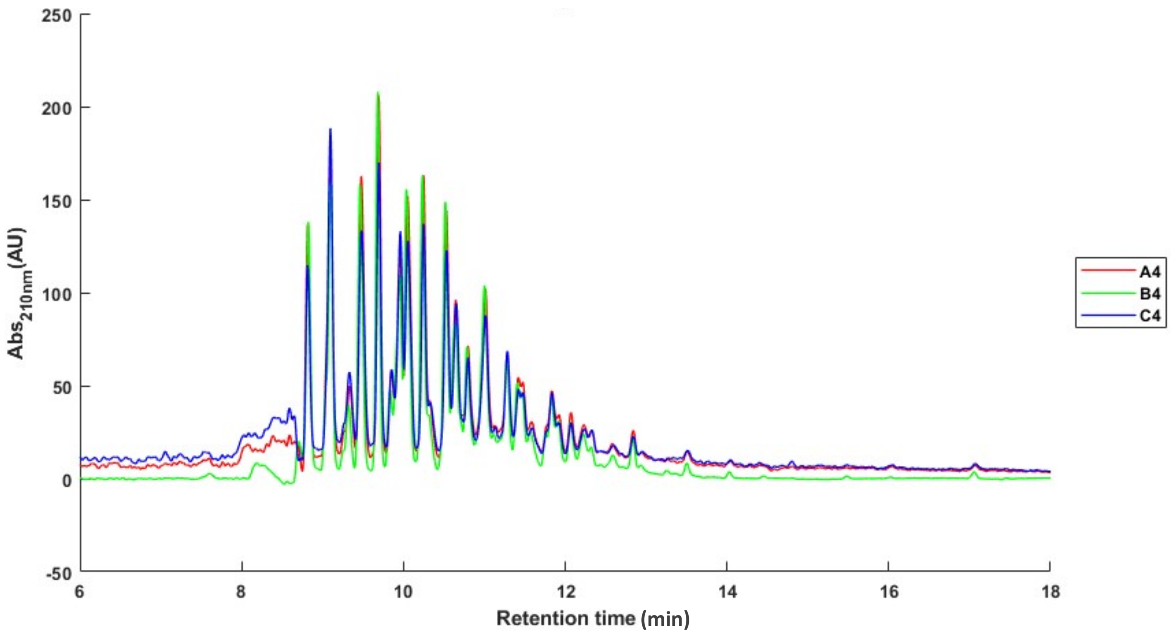

Supplementary Figure S64. HPLC overlay chromatogram of 3 repeats of glycolic acid and β-aminobutyric acid reaction products. glycolic acid (glc) and β-aminobutyric acid (β-Aba) were dried for 7 days in 85 °C, at a 5:1 molar ratio, in favor of glc. Dry down products were then resuspended in an aqueous solution of 20% acetonitrile in water (v/v). The resulting products were seperated on C18 column.

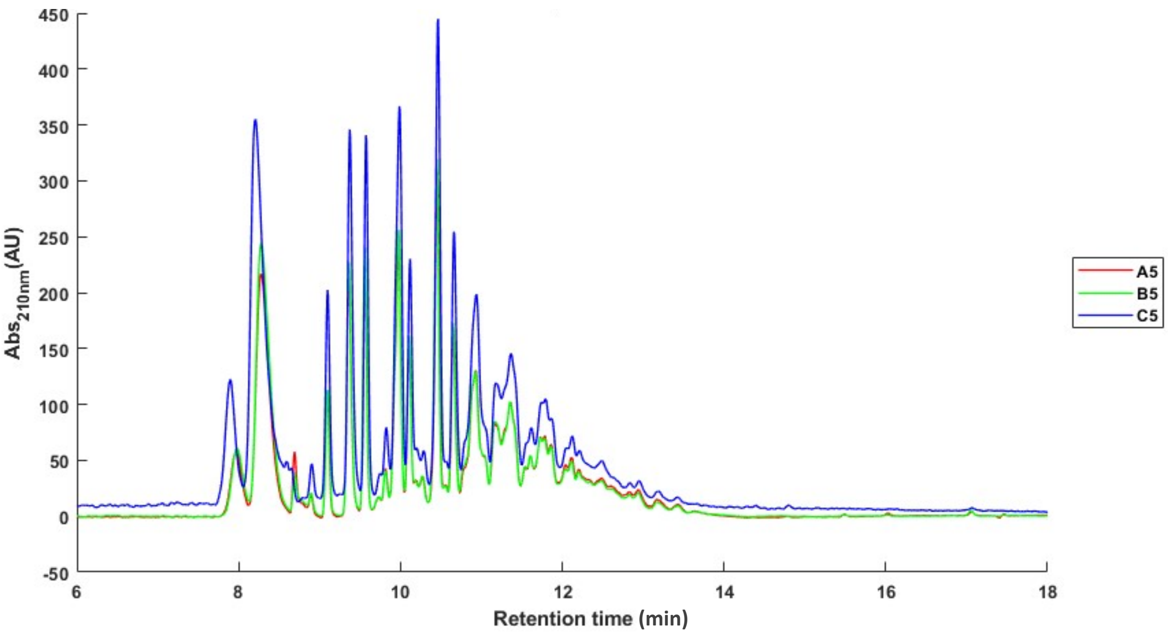

Supplementary Figure S65. HPLC overlay chromatogram of 3 repeats of glycolic acid and γ-aminobutyric acid reaction products. glycolic acid (glc) and γ-aminobutyric (γ-Aba)acid were dried for 7 days in 85 °C, at a 5:1 molar ratio, in favor of glc. Dry down products were then resuspended in an aqueous solution of 20% acetonitrile in water (v/v). The resulting products were seperated on C18 column.

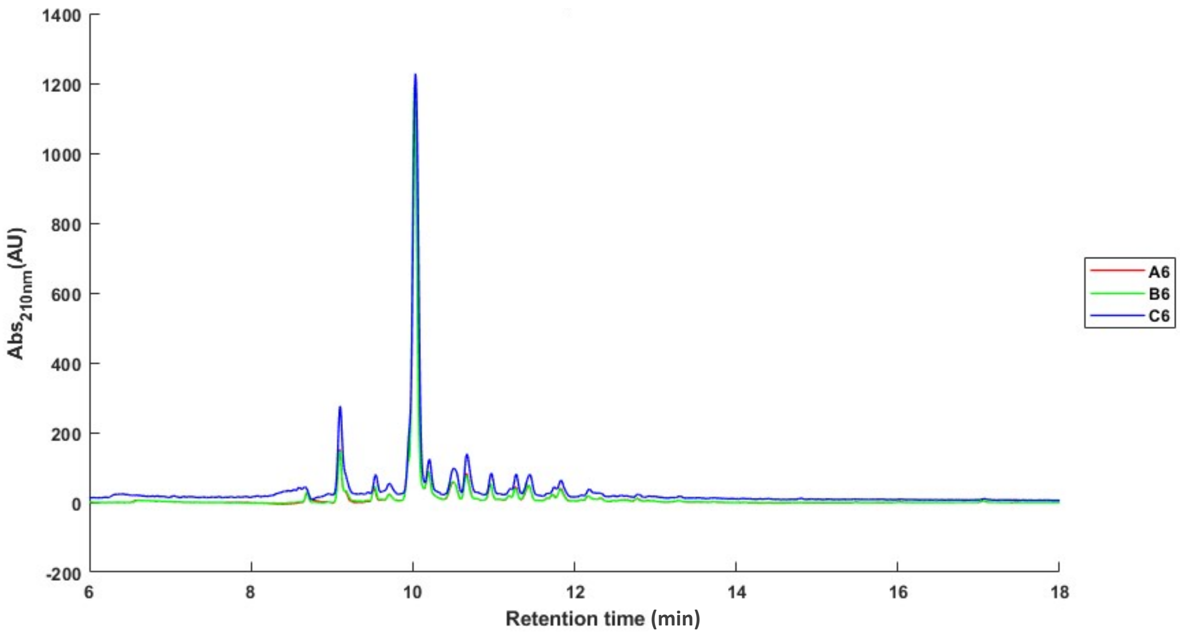

Supplementary Figure S66. HPLC overlay chromatogram of 3 repeats of glycolic acid and 4-aminopentanoic acid reaction products. glycolic acid (glc) and 4-aminopentanoic (γ-Apa) acid acid were dried for 7 days in 85 °C, at a 5:1 molar ratio, in favor of glc. Dry down products were then resuspended in an aqueous solution of 20% acetonitrile in water (v/v). The resulting products were seperated on C18 column.

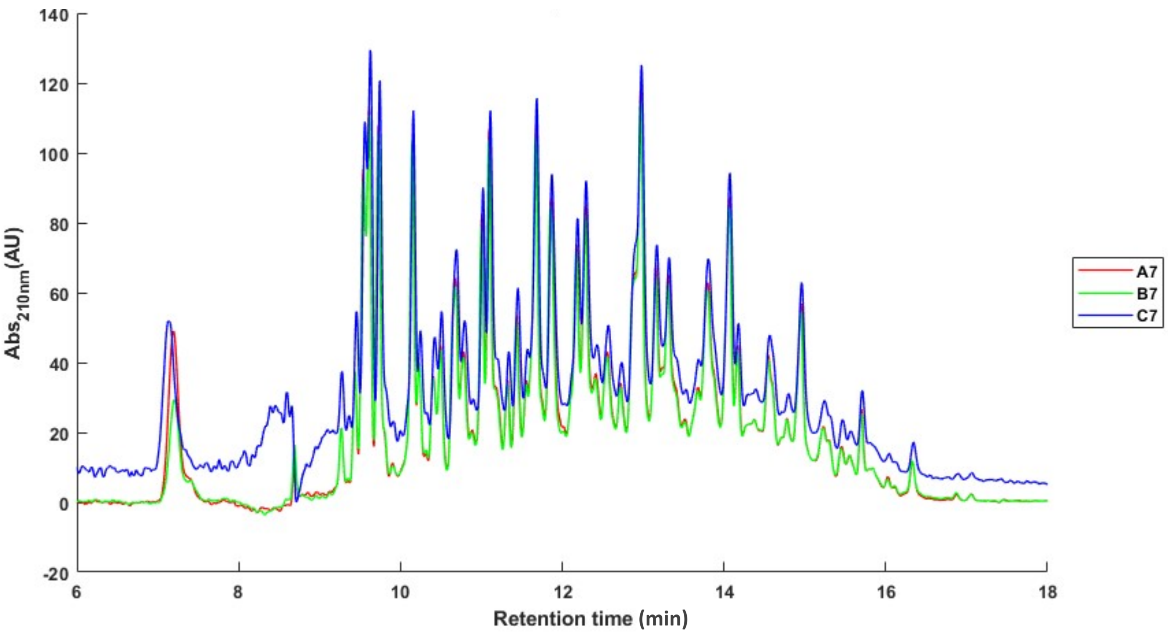

Supplementary Figure S67. HPLC overlay chromatogram of 3 repeats of L-lactic acid and glycine reaction products. L-lactic acid (lac) and glycine (Gly) were dried for 7 days in 85 °C, at a 5:1 molar ratio, in favor of lac. Dry down products were then resuspended in an aqueous solution of 20% acetonitrile in water (v/v). The resulting products were seperated on C18 column.

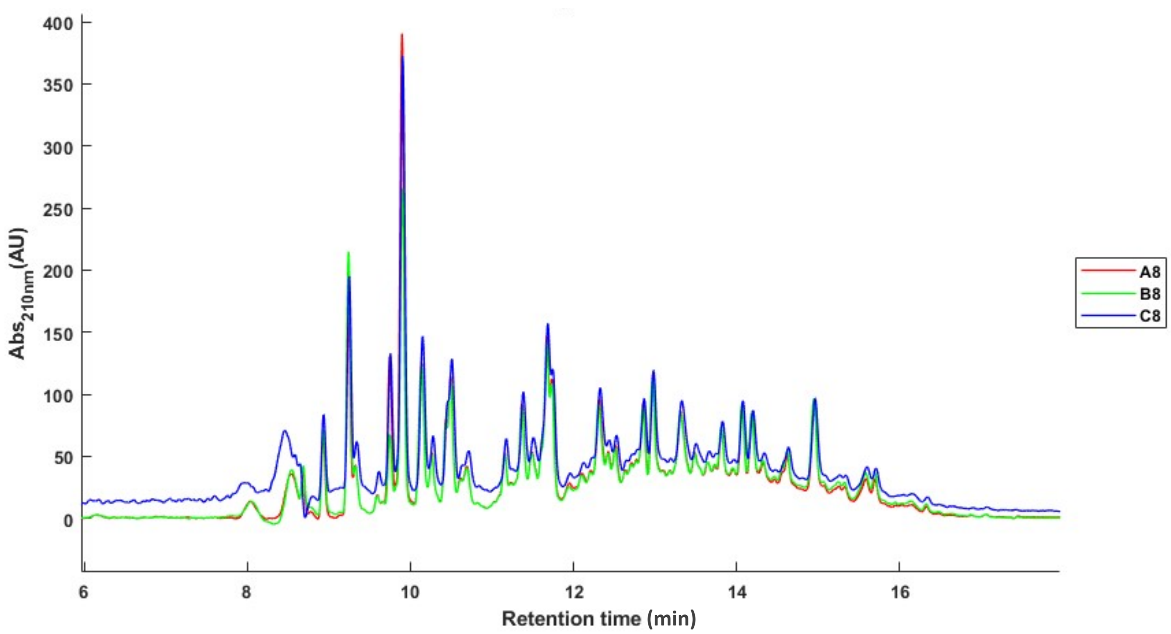

Supplementary Figure S68. HPLC overlay chromatogram of 3 repeats of L-lactic acid and L-alanine reaction products. L-lactic acid (lac) and L-alanine (Ala) were dried for 7 days in 85 °C, at a 5:1 molar ratio, in favor of lac. Dry down products were then resuspended in an aqueous solution of 20% acetonitrile in water (v/v). The resulting products were seperated on C18 column.

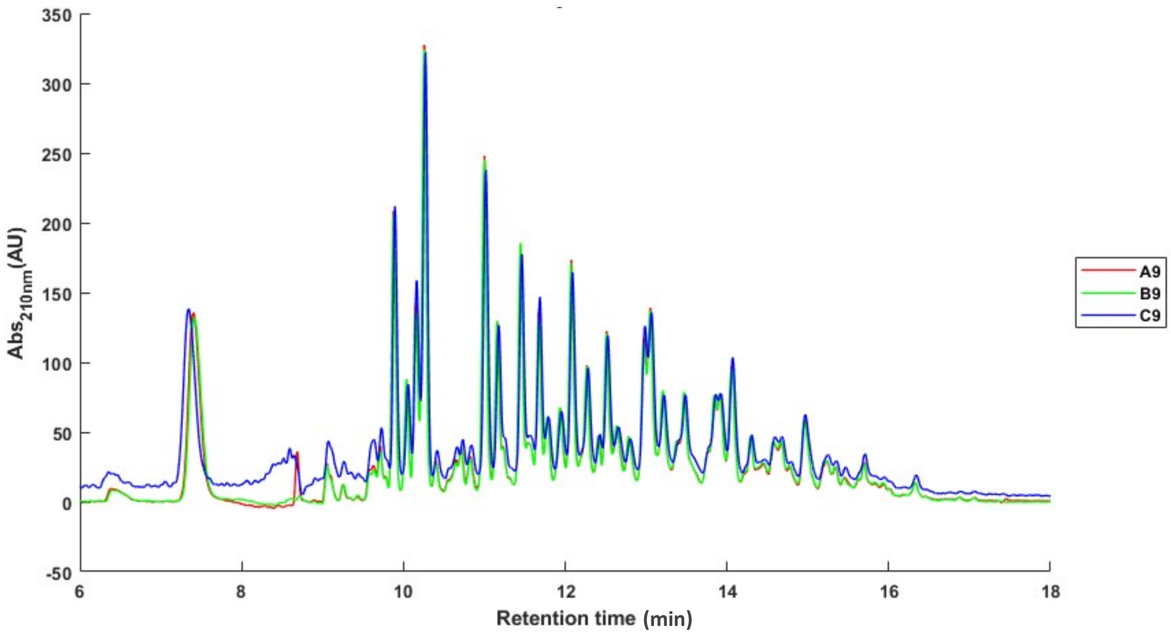

Supplementary Figure S69. HPLC overlay chromatogram of 3 repeats of L-lactic acid and β-alanine reaction products. L-lactic acid (lac) and β-alanine (β-Ala) were dried for 7 days in 85 °C, at a 5:1 molar ratio, in favor of lac. Dry down products were then resuspended in an aqueous solution of 20% acetonitrile in water (v/v). The resulting products were seperated on C18 column.

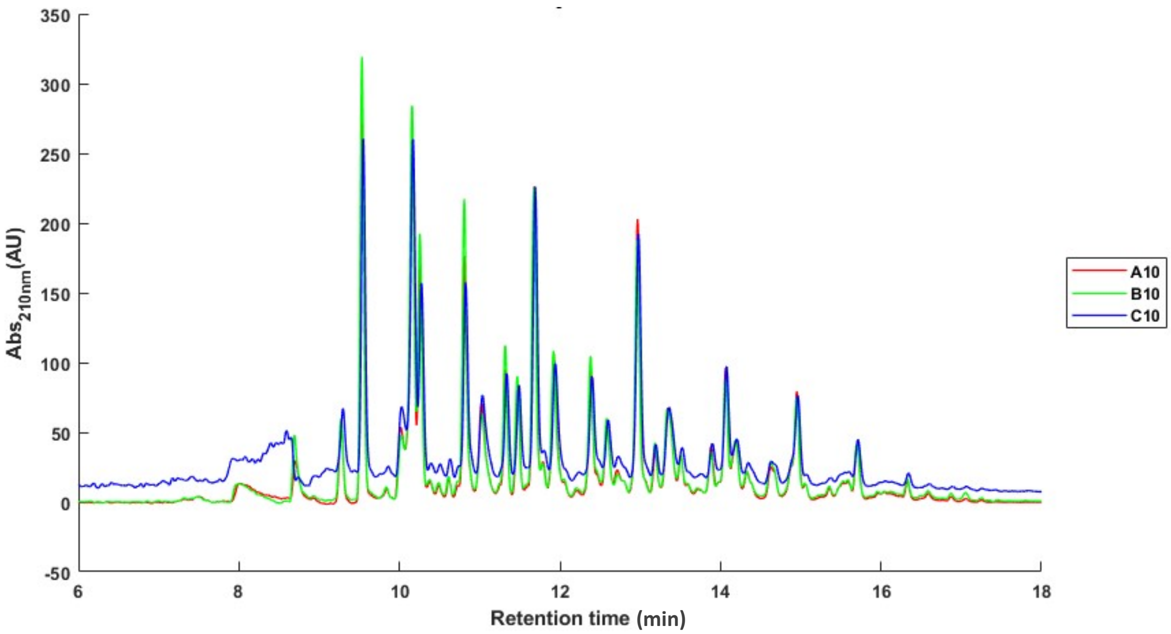

Supplementary Figure S70. HPLC overlay chromatogram of 3 repeats of L-lactic acid and β-aminobutyric acid reaction products. L-lactic acid (lac) and β-aminobutyric (β-Aba) acid were dried for 7 days in 85 °C, at a 5:1 molar ratio, in favor of lac. Dry down products were then resuspended in an aqueous solution of 20% acetonitrile in water (v/v). The resulting products were seperated on C18 column.

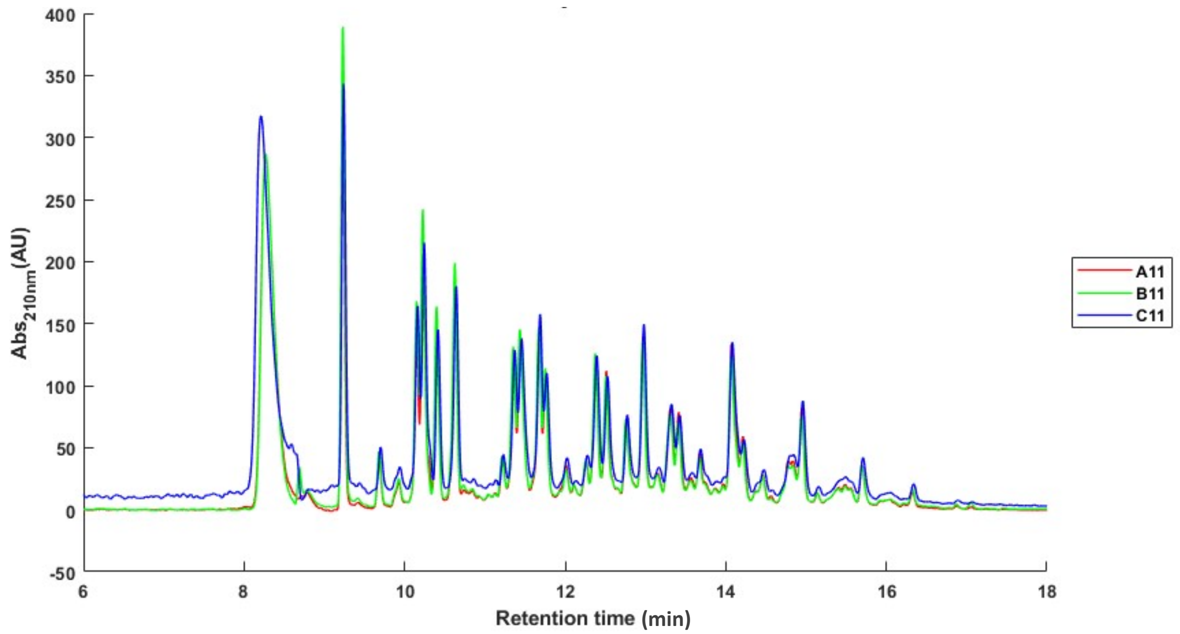

Supplementary Figure S71. HPLC overlay chromatogram of 3 repeats of L-lactic acid and γ-aminobutyric acid reaction products. L-lactic acid (lac) and γ-aminobutyric (γ-Aba) acid were dried for 7 days in 85 °C, at a 5:1 molar ratio, in favor of lac. Dry down products were then resuspended in an aqueous solution of 20% acetonitrile in water (v/v). The resulting products were seperated on C18 column.

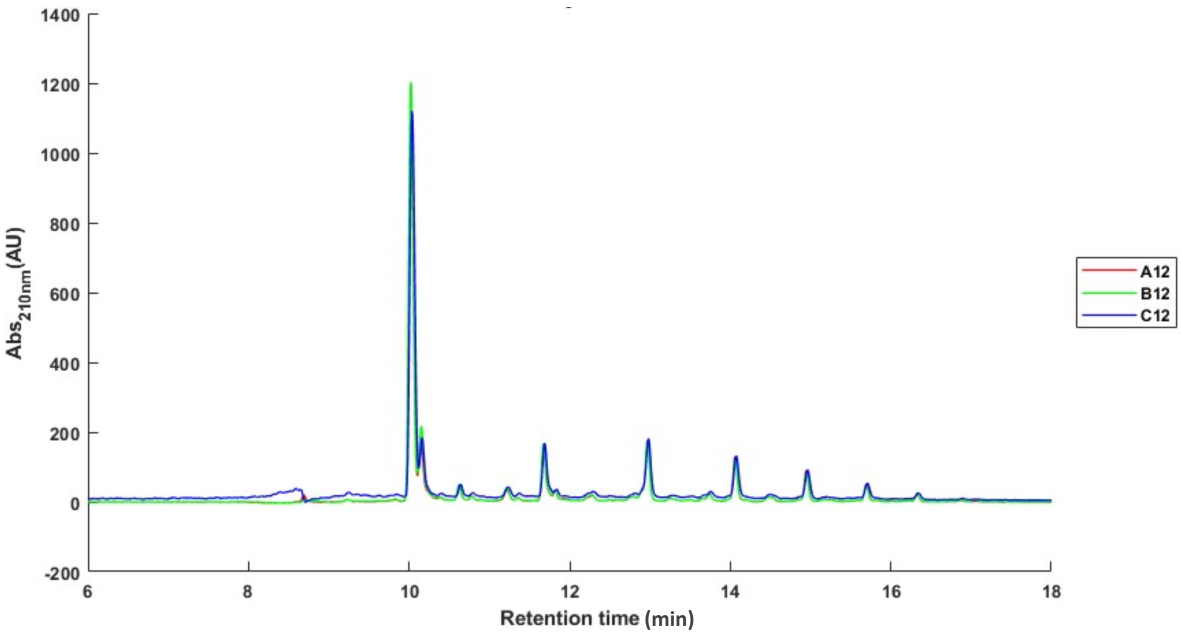

Supplementary Figure S72. HPLC overlay chromatogram of 3 repeats of L-lactic acid and 4-aminopentanoic acid reaction products. L-lactic acid (lac) and 4-aminopentanoic (γ-Apa) acid were dried for 7 days in 85 °C, at a 5:1 molar ratio, in favor of lac. Dry down products were then resuspended in an aqueous solution of 20% acetonitrile in water (v/v). The resulting products were seperated on C18 column.

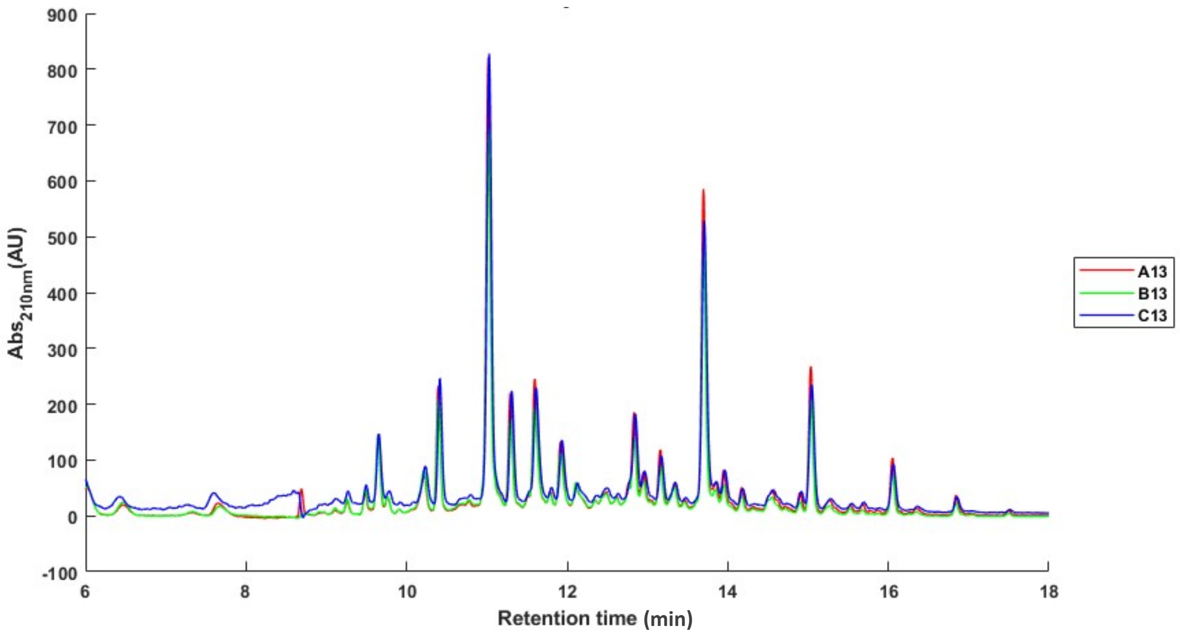

Supplementary Figure S73. HPLC overlay chromatogram of 3 repeats of Hydroxybutyric acid and glycine reaction products. Hydroxybutyric acid (hba) and glycine (Gly) were dried for 7 days in 85 °C, at a 5:1 molar ratio, in favor of hba. Dry down products were then resuspended in an aqueous solution of 20% acetonitrile in water (v/v). The resulting products were seperated on C18 column.

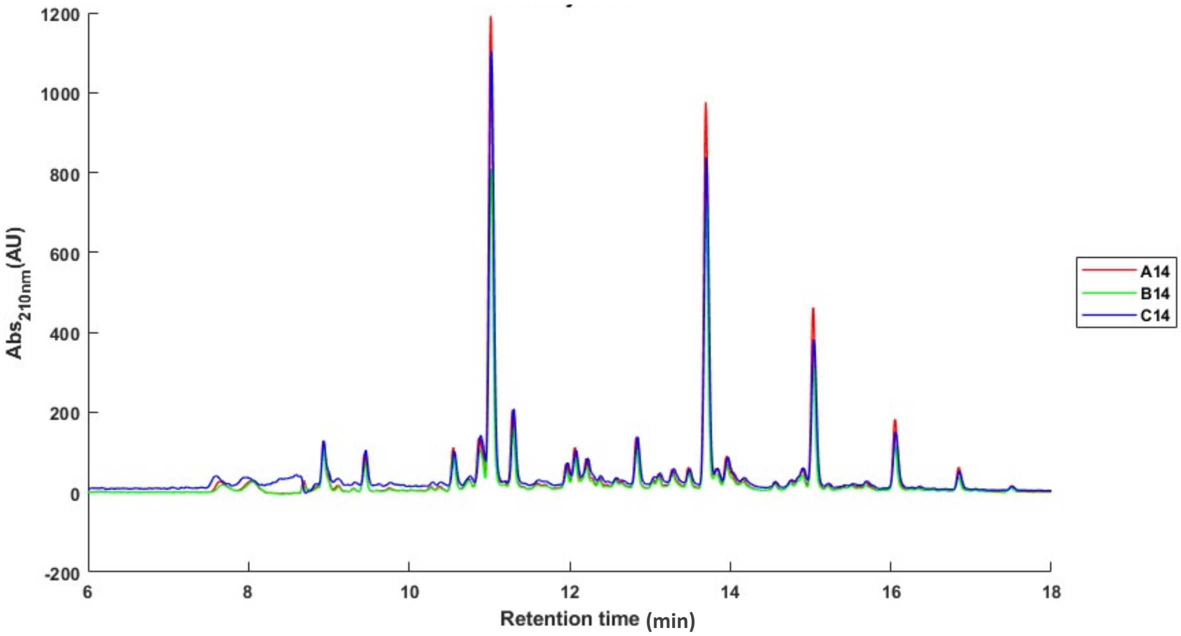

Supplementary Figure S74. HPLC overlay chromatogram of 3 repeats of Hydroxybutyric acid and L-alanine reaction products. Hydroxybutyric acid (hba) and L-alanine (Ala) were dried for 7 days in 85 °C, at a 5:1 molar ratio, in favor of hba. Dry down products were then resuspended in an aqueous solution of 20% acetonitrile in water (v/v). The resulting products were seperated on C18 column.

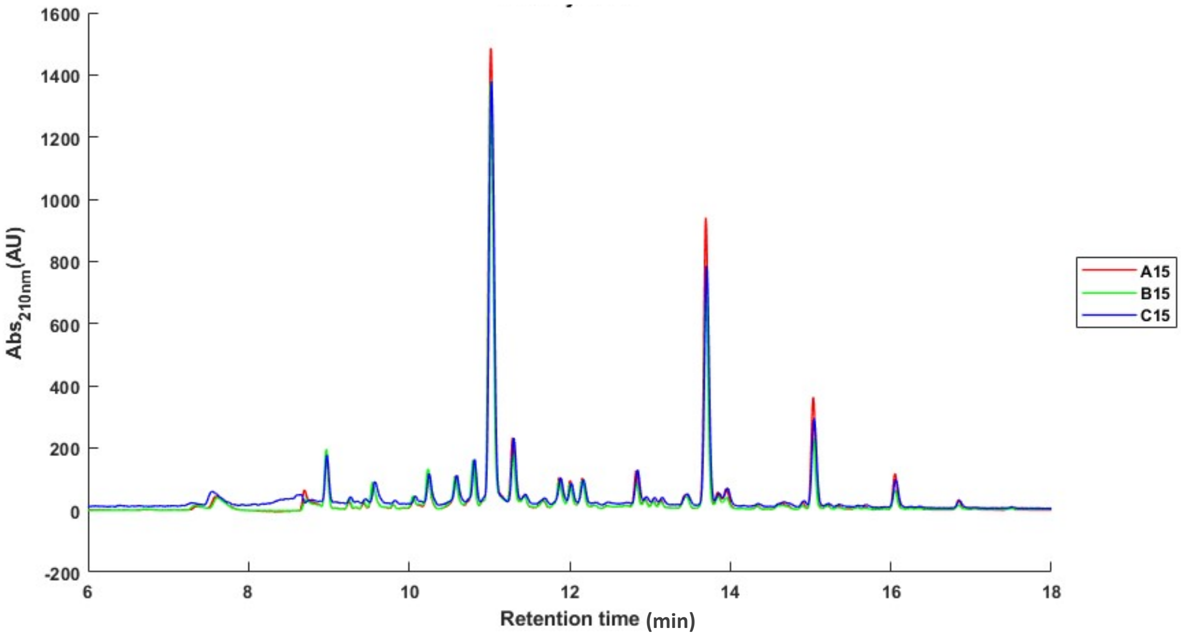

Supplementary Figure S75. HPLC overlay chromatogram of 3 repeats of Hydroxybutyric acid and β-alanine reaction products. Hydroxybutyric acid (hba) and β-alanine (β-Ala) were dried for 7 days in 85 °C, at a 5:1 molar ratio, in favor of hba. Dry down products were then resuspended in an aqueous solution of 20% acetonitrile in water (v/v). The resulting products were seperated on C18 column.

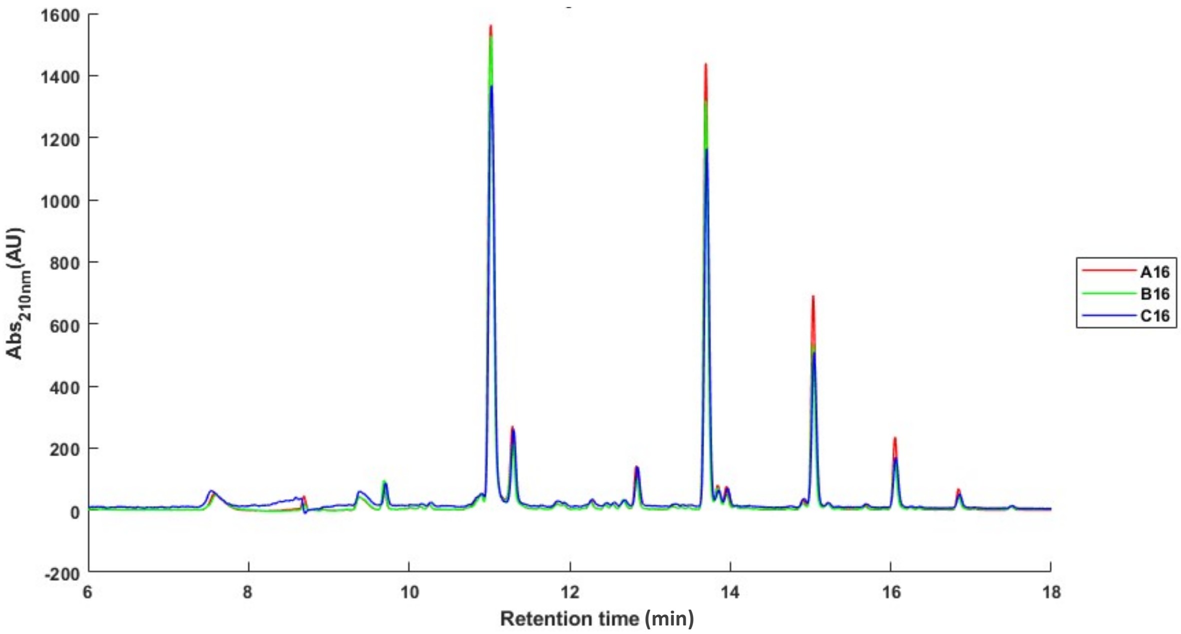

Supplementary Figure S76. HPLC overlay chromatogram of 3 repeats of Hydroxybutyric acid and β-aminobutyric acid reaction products. Hydroxybutyric acid (hba) and β-aminobutyric (β-Aba) acid were dried for 7 days in 85 °C, at a 5:1 molar ratio, in favor of hba. Dry down products were then resuspended in an aqueous solution of 20% acetonitrile in water (v/v). The resulting products were seperated on C18 column.

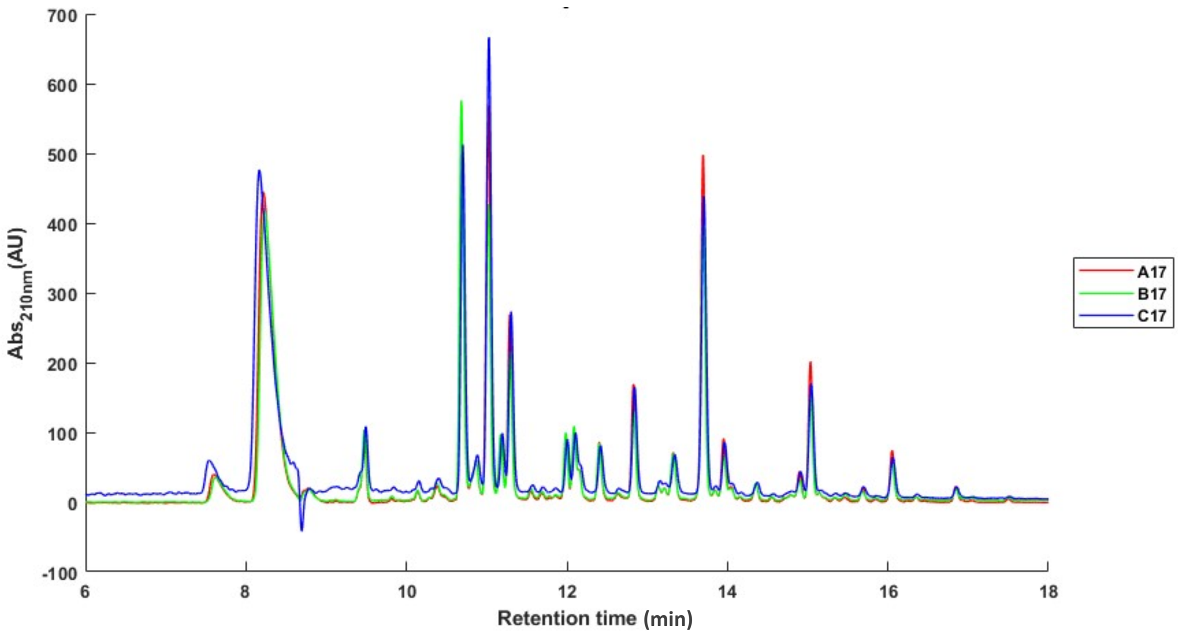

Supplementary Figure S77. HPLC overlay chromatogram of 3 repeats of Hydroxybutyric acid and γ-aminobutyric acid reaction products. Hydroxybutyric acid (hba) and γ-aminobutyric acid (γ-Aba) were dried for 7 days in 85 °C, at a 5:1 molar ratio, in favor of hba. Dry down products were then resuspended in an aqueous solution of 20% acetonitrile in water (v/v). The resulting products were seperated on C18 column.

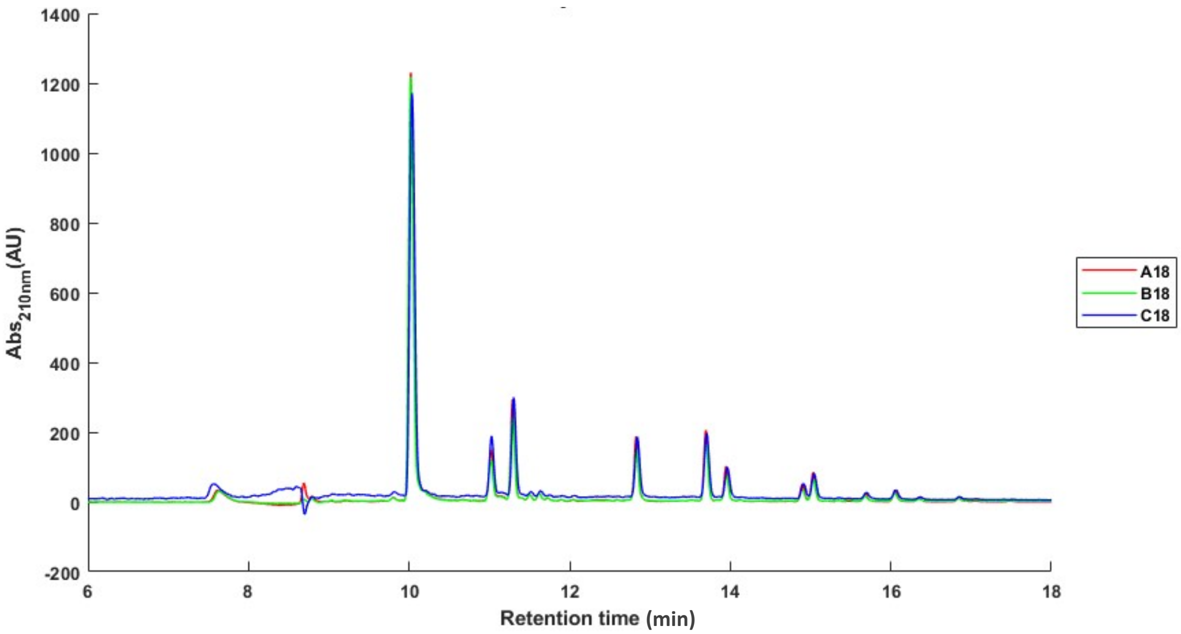

Supplementary Figure S78. HPLC overlay chromatogram of 3 repeats of Hydroxybutyric acid and 4-aminopentanoic acid reaction products. Hydroxybutyric acid (hba) and γ-aminopentanoic (γ-Apa) acid were dried for 7 days in 85 °C, at a 5:1 molar ratio, in favor of hba. Dry down products were then resuspended in an aqueous solution of 20% acetonitrile in water (v/v). The resulting products were seperated on C18 column.

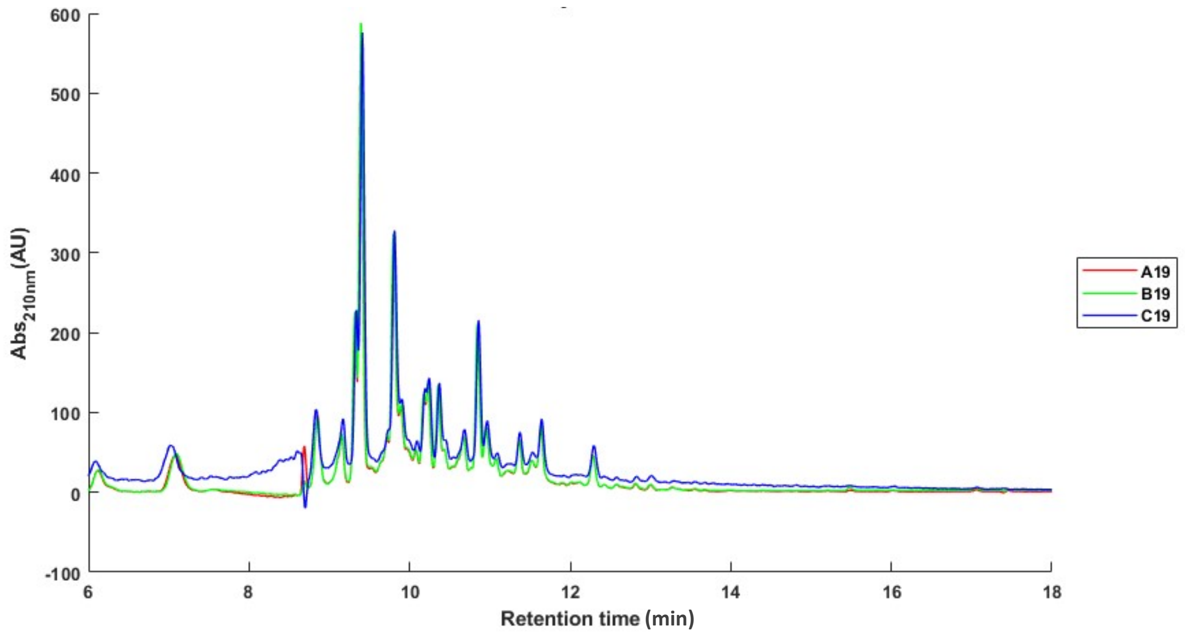

Supplementary Figure S79. HPLC overlay chromatogram of 3 repeats of Hydroxypropionic acid and glycine reaction products. Hydroxypropionic acid (hpa) and glycine (Gly) were dried for 7 days in 85 °C, at a 5:1 molar ratio, in favor of hpa. Dry down products were then resuspended in an aqueous solution of 20% acetonitrile in water (v/v). The resulting products were seperated on C18 column.

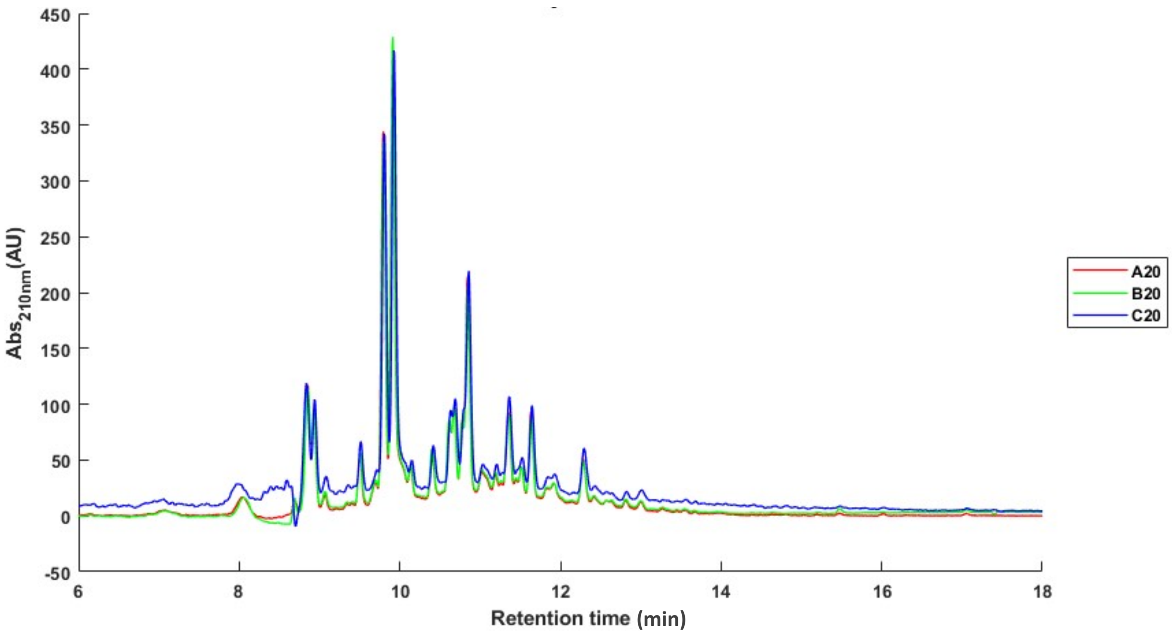

Supplementary Figure S80. HPLC overlay chromatogram of 3 repeats of Hydroxypropionic acid and L-alanine reaction products. Hydroxypropionic acid (hpa and L-alanine (Ala) were dried for 7 days in 85 °C, at a 5:1 molar ratio, in favor of hpa. Dry down products were then resuspended in an aqueous solution of 20% acetonitrile in water (v/v). The resulting products were seperated on C18 column.

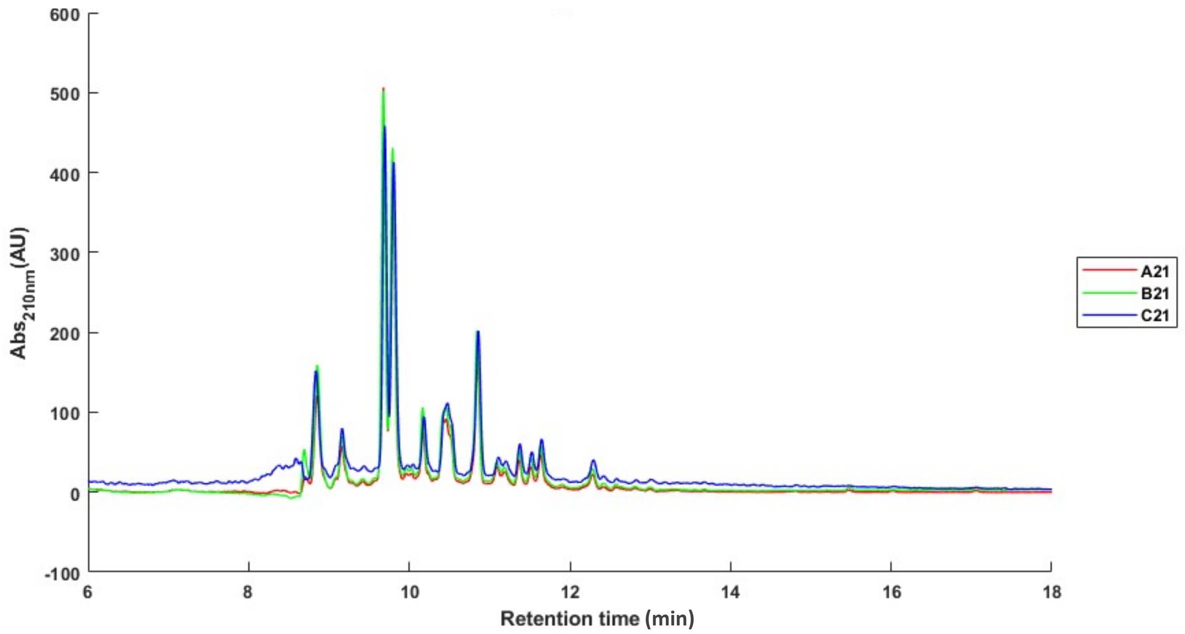

Supplementary Figure S81. HPLC overlay chromatogram of 3 repeats of Hydroxypropionic acid and β-alanine reaction products. Hydroxypropionic acid (hpa) and β-alanine (β-Ala) were dried for 7 days in 85 °C, at a 5:1 molar ratio, in favor of hpa. Dry down products were then resuspended in an aqueous solution of 20% acetonitrile in water (v/v). The resulting products were seperated on C18 column.

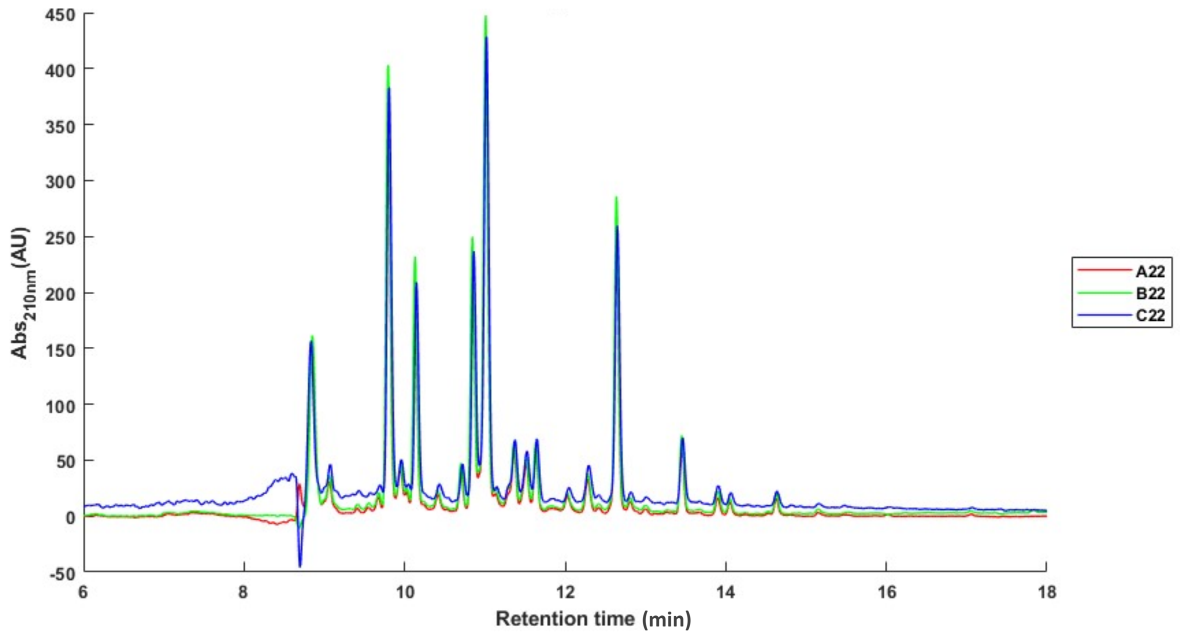

Supplementary Figure S82. HPLC overlay chromatogram of 3 repeats of Hydroxypropionic acid and β-aminobutyric acid reaction products. Hydroxypropionic acid (hpa) and β-aminobutyric acid (β-Aba) were dried for 7 days in 85 °C, at a 5:1 molar ratio, in favor of hpa. Dry down products were then resuspended in an aqueous solution of 20% acetonitrile in water (v/v). The resulting products were seperated on C18 column.

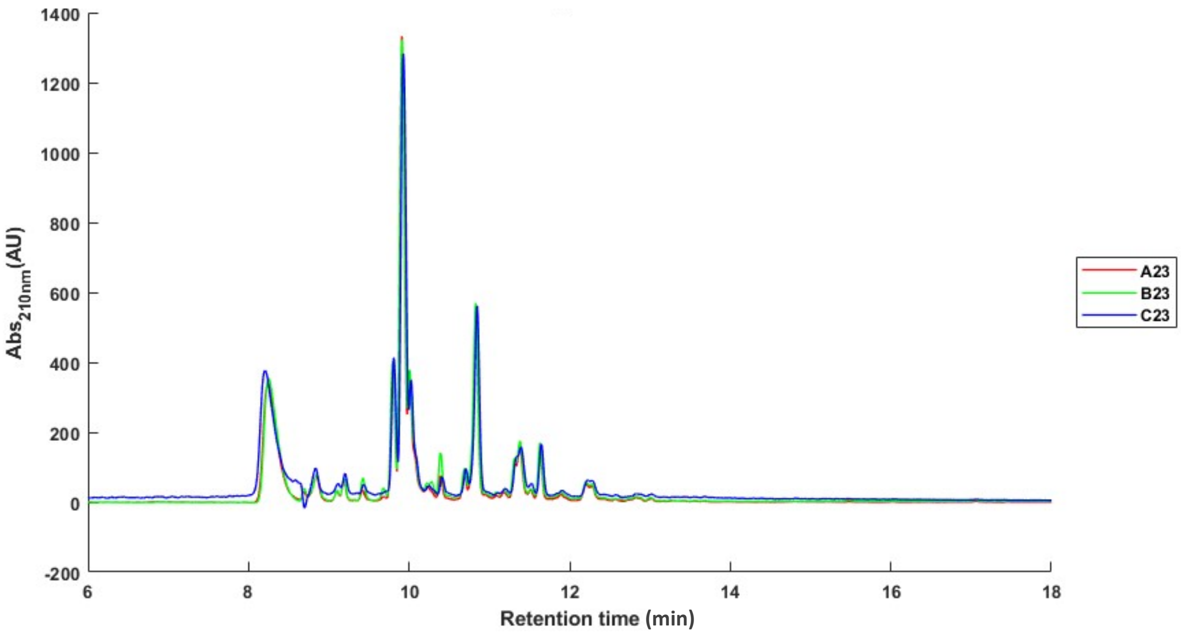

Supplementary Figure S83. HPLC overlay chromatogram of 3 repeats of Hydroxypropionic acid and γ-aminobutyric acid reaction products. Hydroxypropionic acid (hpa) and γ-aminobutyric acid (γ-Aba) were dried for 7 days in 85 °C, at a 5:1 molar ratio, in favor of hpa. Dry down products were then resuspended in an aqueous solution of 20% acetonitrile in water (v/v). The resulting products were seperated on C18 column.

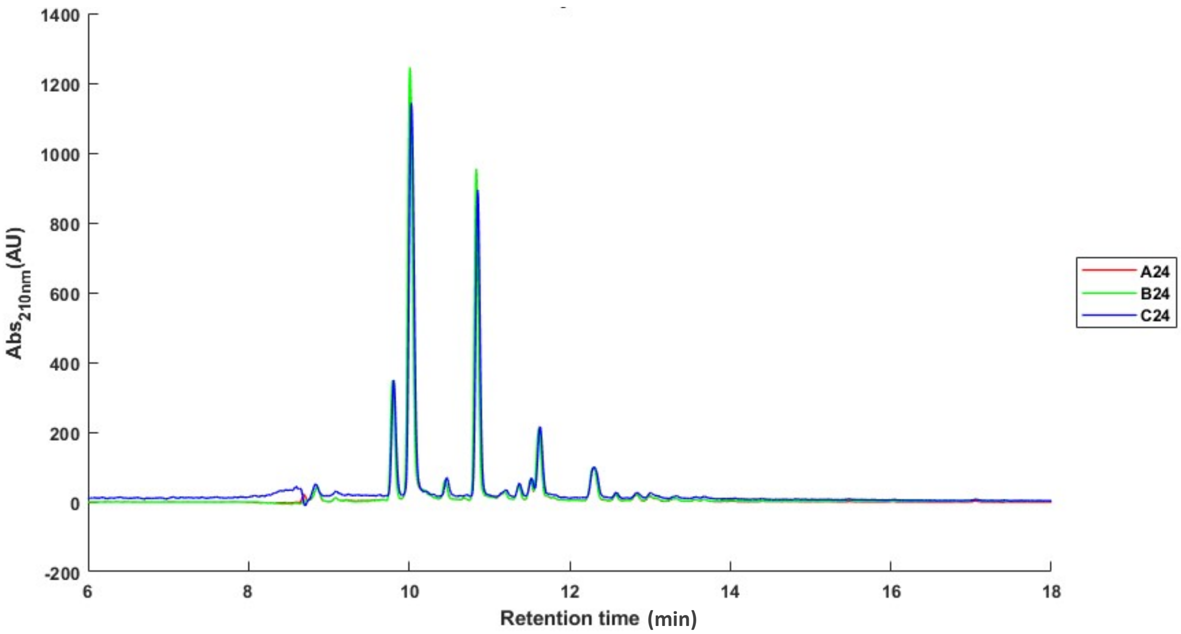

Supplementary Figure S84. HPLC overlay chromatogram of 3 repeats of Hydroxypropionic acid and 4-aminopentanoic acid reaction products. Hydroxypropionic acid (hpa) and 4-aminopentanoic (γ-Apa) acid were dried for 7 days in 85 °C, at a 5:1 molar ratio, in favor of hpa. Dry down products were then resuspended in an aqueous solution of 20% acetonitrile in water (v/v). The resulting products were seperated on C18 column.

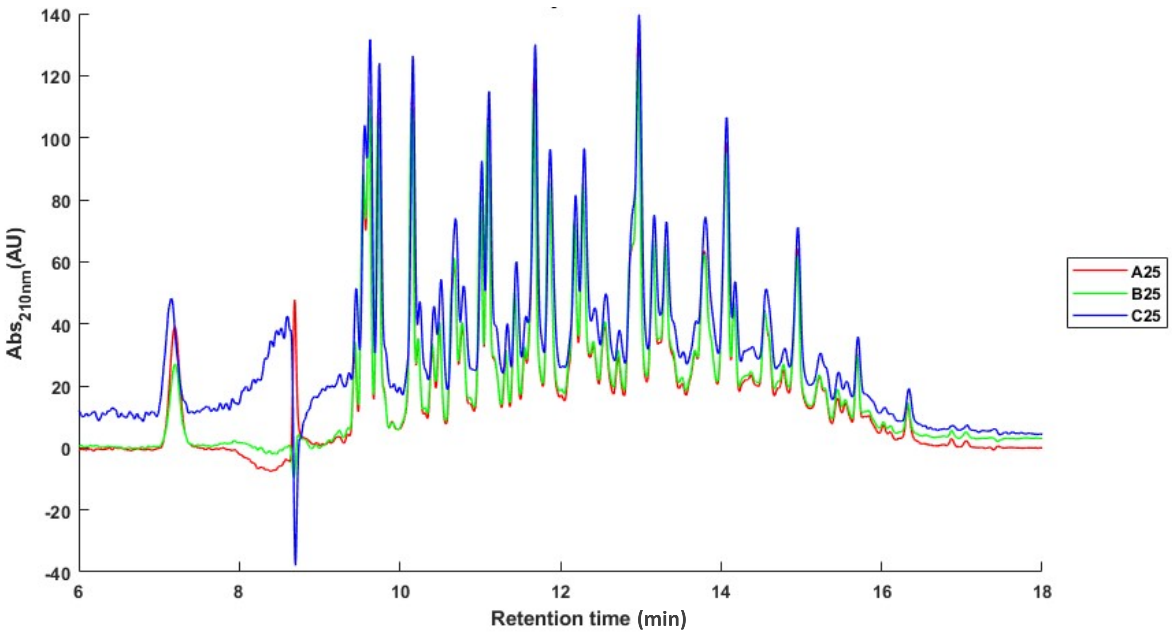

Supplementary Figure S85. HPLC overlay chromatogram of 3 repeats of DL-lactic acid and glycine reaction products. DL-lactic acid (DL-lac) and glycine (Gly) were dried for 7 days in 85 °C, at a 5:1 molar ratio, in favor of DL-lac. Dry down products were then resuspended in an aqueous solution of 20% acetonitrile in water (v/v). The resulting products were seperated on C18 column.

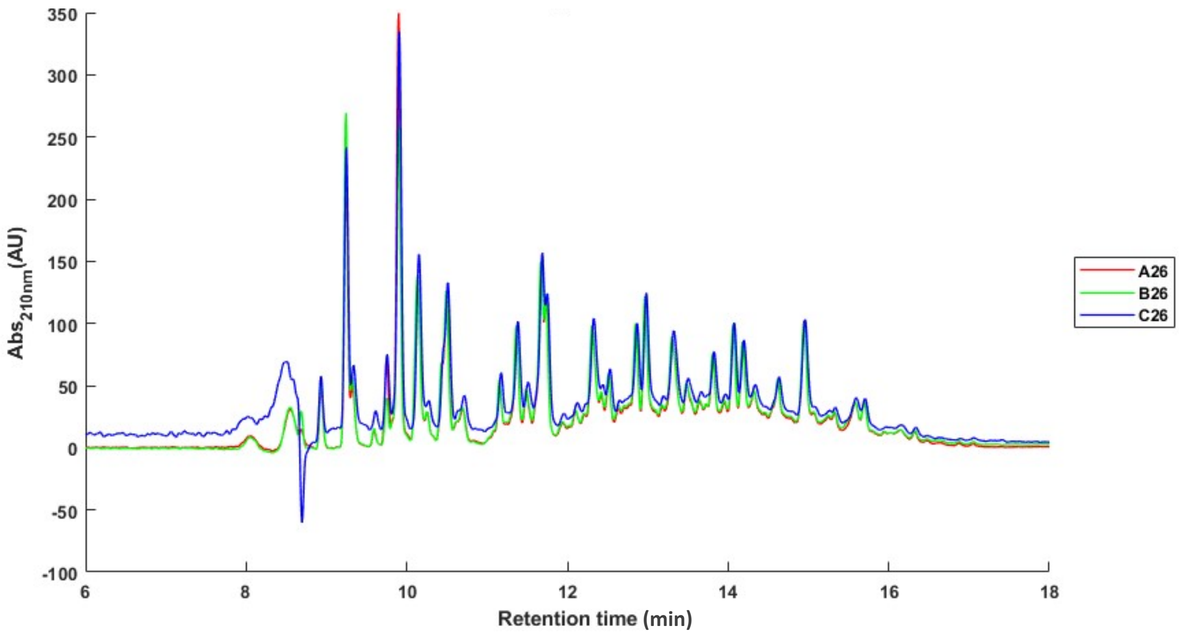

Supplementary Figure S86. HPLC overlay chromatogram of 3 repeats of DL-lactic acid and L-alanine reaction products. DL-lactic acid (DL-lac) and L-alanine (Ala) were dried for 7 days in 85 °C, at a 5:1 molar ratio, in favor of DL-lac. Dry down products were then resuspended in an aqueous solution of 20% acetonitrile in water (v/v). The resulting products were seperated on C18 column.

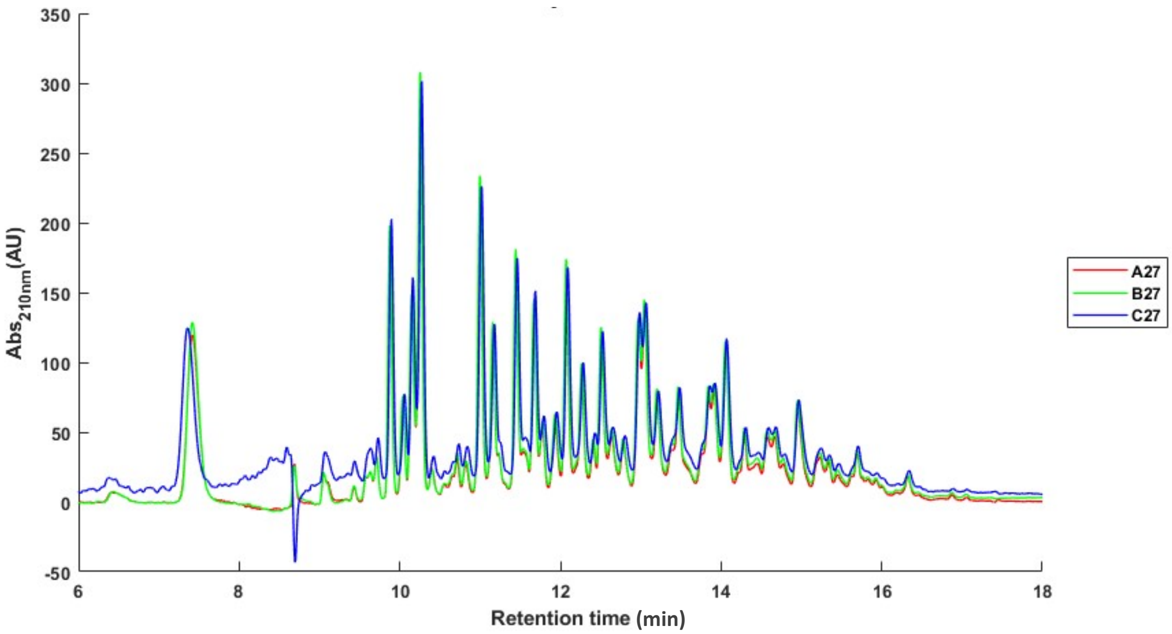

Supplementary Figure S87. HPLC overlay chromatogram of 3 repeats of DL-lactic acid and β-alanine reaction products. DL-lactic acid (DL-lac) and β-alanine (β-Ala) were dried for 7 days in 85 °C, at a 5:1 molar ratio, in favor of DL-lac. Dry down products were then resuspended in an aqueous solution of 20% acetonitrile in water (v/v). The resulting products were seperated on C18 column.

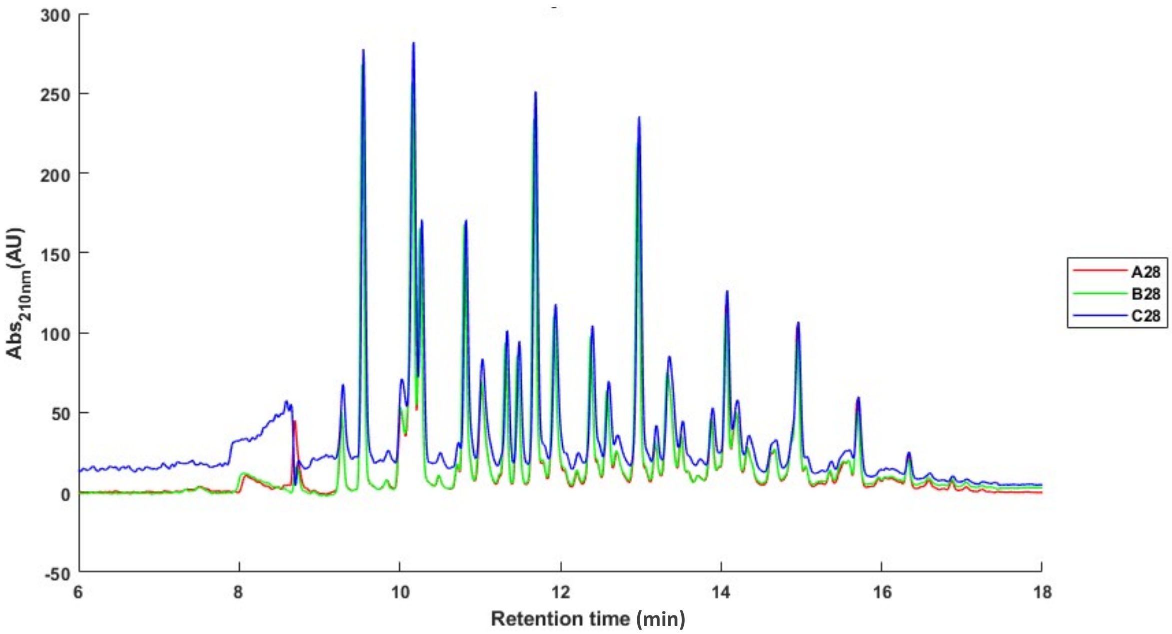

Supplementary Figure S88. HPLC overlay chromatogram of 3 repeats of DL-lactic acid and β-aminobutyric acid reaction products. DL-lactic acid (DL-lac) and β-aminobutyric acid (β-Aba) were dried for 7 days in 85 °C, at a 5:1 molar ratio, in favor of DL-lac. Dry down products were then resuspended in an aqueous solution of 20% acetonitrile in water (v/v). The resulting products were seperated on C18 column.

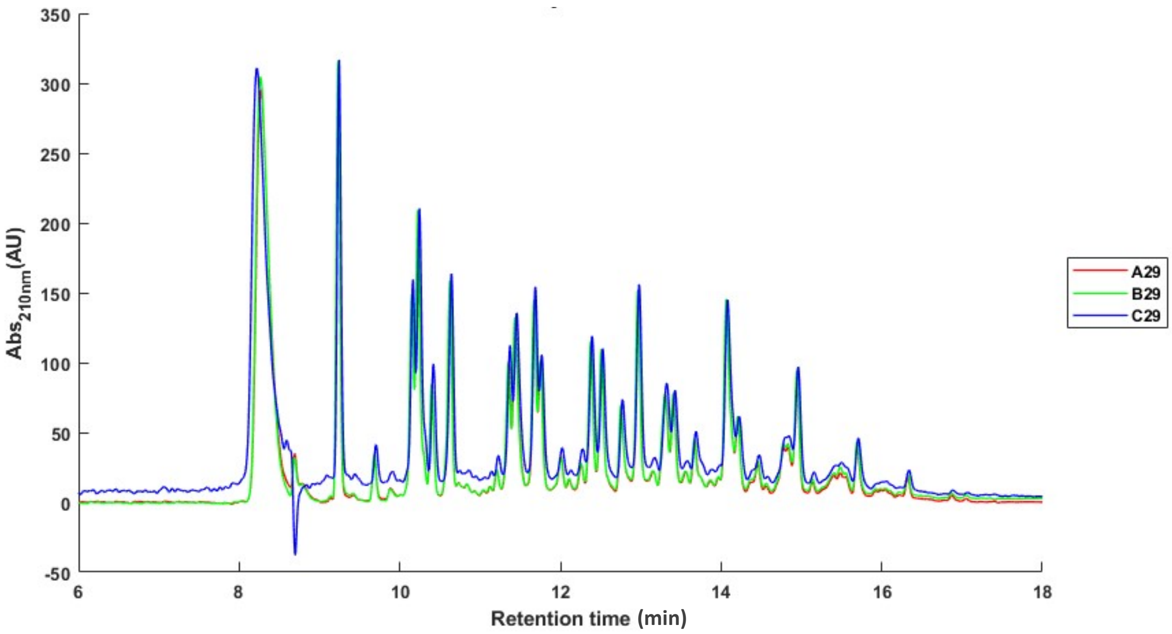

Supplementary Figure S89. HPLC overlay chromatogram of 3 repeats of DL-lactic acid and γ-aminobutyric acid reaction products. DL-lactic acid (DL-lac) and γ-aminobutyric acid (γ-Apa) were dried for 7 days in 85 °C, at a 5:1 molar ratio, in favor of DL-lac. Dry down products were then resuspended in an aqueous solution of 20% acetonitrile in water (v/v). The resulting products were seperated on C18 column.

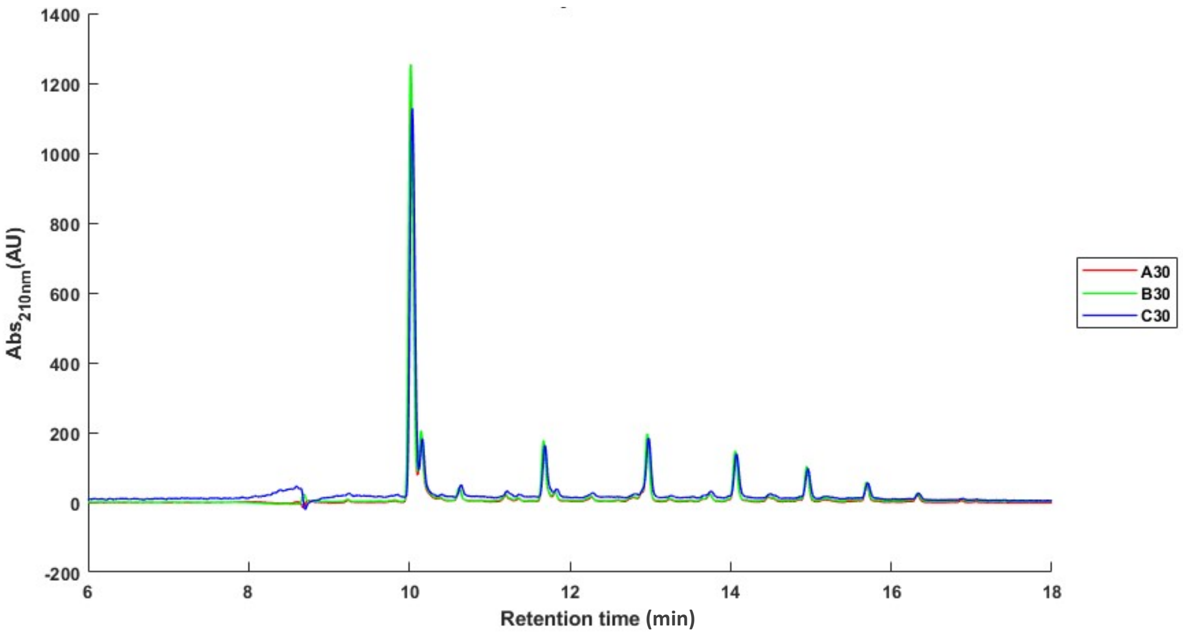

Supplementary Figure S90. HPLC overlay chromatogram of 3 repeats of DL-lactic acid and 4-aminopentanoic acid reaction products. DL-lactic acid (DL-lac) and 4-aminopentanoic (γ-Apa) acid were dried for 7 days in 85 °C, at a 5:1 molar ratio, in favor of DL-lac. Dry down products were then resuspended in an aqueous solution of 20% acetonitrile in water (v/v). The resulting products were seperated on C18 column.

AA /HA pair	Volume(µl)	Solvent	HA/AA ratio (µmole/µmole)	Sample internal code
glycolic Acid+glycine	500	80% H2O 20%ACN	250/50	A1
glycolic Acid+L-alanine	500	80% H2O 20%ACN	250/50	A2
glycolic Acid+beta alanine	500	80% H2O 20%ACN	250/50	A3
glycolic Acid+beta ABA	500	80% H2O 20%ACN	250/50	A4
glycolic Acid+gamma ABA	500	80% H2O 20%ACN	250/50	A5
glycolic Acid+ gamma APA	500	80% H2O 20%ACN	250/50	A6
L-lactic acid+glycine	500	80% H2O 20%ACN	250/50	A7
L-lactic acid+L-alanine	500	80% H2O 20%ACN	250/50	A8
L-lactic acid+beta alanine	500	80% H2O 20%ACN	250/50	A9
L-lactic acid+beta ABA	500	80% H2O 20%ACN	250/50	A10
L-lactic acid+gamma ABA	500	80% H2O 20%ACN	250/50	A11
L-lactic acid+ gamma APA	500	80% H2O 20%ACN	250/50	A12
HBA+glycine	500	80% H2O 20%ACN	250/50	A13
HBA+L-alanine	500	80% H2O 20%ACN	250/50	A14
HBA+beta alanine	500	80% H2O 20%ACN	250/50	A15
HBA+beta ABA	500	80% H2O 20%ACN	250/50	A16
HBA+gamma ABA	500	80% H2O 20%ACN	250/50	A17
HBA+ gamma APA	500	80% H2O 20%ACN	250/50	A18
HPA+glycine	500	80% H2O 20%ACN	250/50	A19
HPA+L-alanine	500	80% H2O 20%ACN	250/50	A20
HPA+beta alanine	500	80% H2O 20%ACN	250/50	A21
HPA+beta ABA	500	80% H2O 20%ACN	250/50	A22
HPA+gamma ABA	500	80% H2O 20%ACN	250/50	A23
HPA+ gamma APA	500	80% H2O 20%ACN	250/50	A24
DL - lactic acid - glycine	500	80% H2O 20%ACN	250/50	A25
DL - lactic acid - L-alanine	500	80% H2O 20%ACN	250/50	A26
DL - lactic acid - beta alanine	500	80% H2O 20%ACN	250/50	A27
DL - lactic acid - beta ABA	500	80% H2O 20%ACN	250/50	A28
DL - lactic acid - gamma ABA	500	80% H2O 20%ACN	250/50	A29
DL - lactic acid - gamma APA	500	80% H2O 20%ACN	250/50	A30

Supplementary Table S1. List of samples. Batch A sample table coded from A1-A30, each sample code corrseponds to a mixture of amino acid and hydroxy acid, at a ratio of 5:1 in favor of the hydroxy acid. All samples were resuspended in 80% deionized water and 20% aectonitrile (ACN) to a final concentration of 100mM amino acid (referring to initial amount prior to drying).

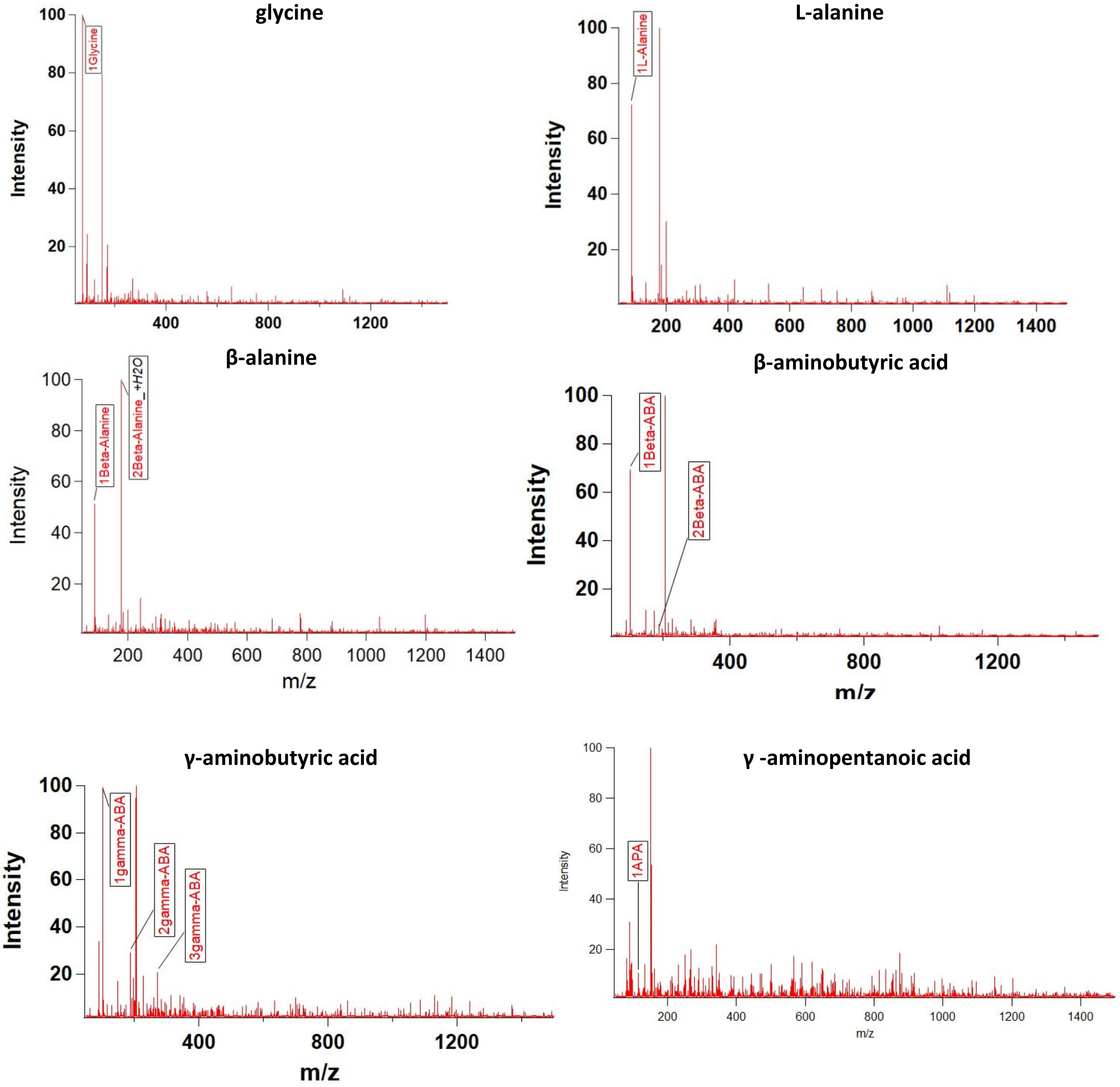

Supplementary Figure S91. ESI-MS of dry down of amino acid control samples – Glycine, Alanine, β-Alanine, β-Aminobutyric acid, γ-Aminobutyric acid, γ-Aminopentanoic acid. Amino acid control dry downs at 50 umole each, for 7 days at 85 °C. Dry down products were then resuspended in an aqueous solution of 20% acetonitrile in water (v/v). The resulting products were analyzed by negative-mode ESI-MS.

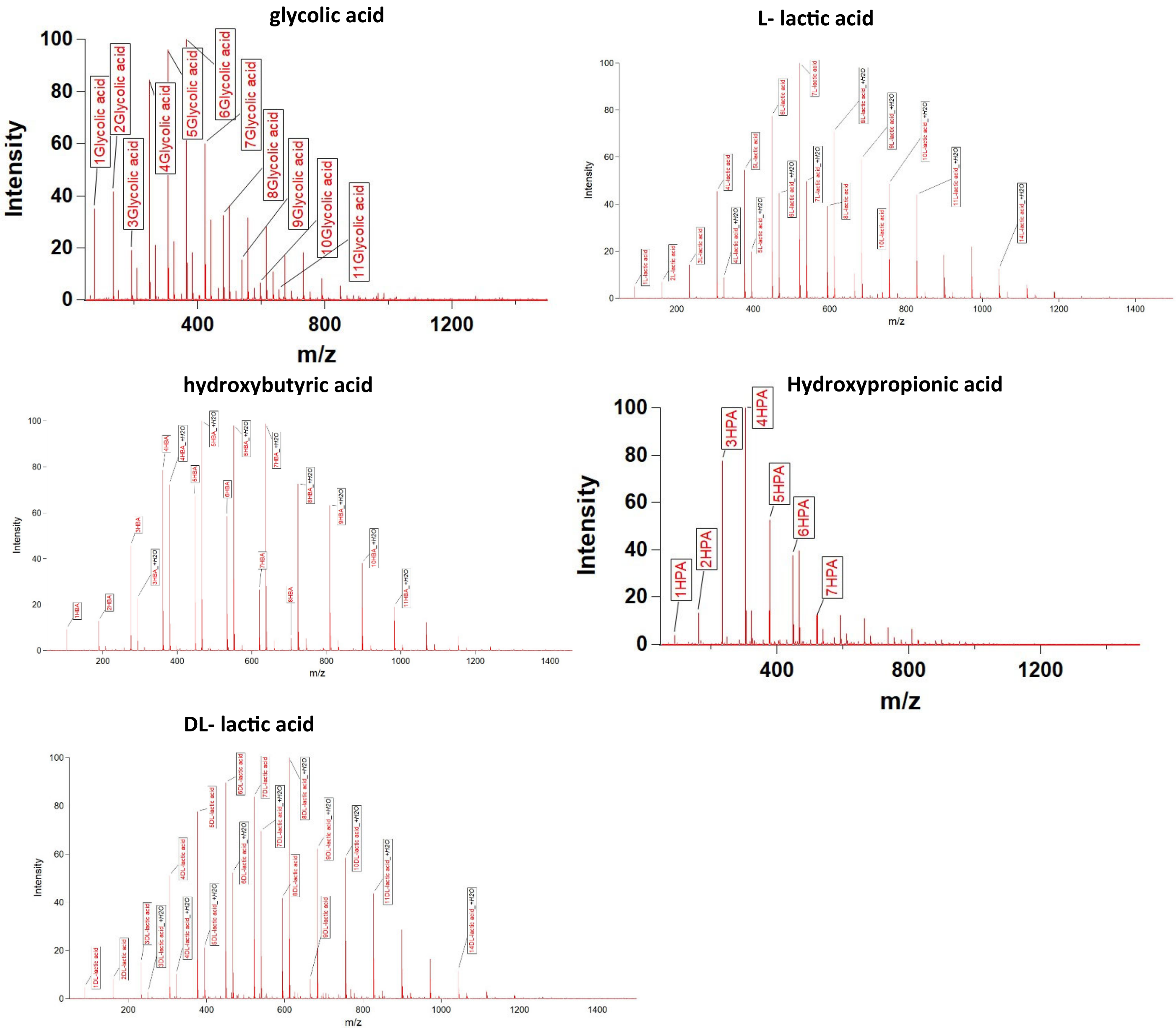

Supplementary Figure S92. ESI-MS of dry down of Hydroxy acid control samples – Glycolic acid, L-lactic acid, Hydroxybutyric acid, Hydroxypropionic acid, DL-lactic acid. Hydroxy acid control dry downs at 250 umole each, for 7 days at 85 °C. Dry down products were then resuspended in an aqueous solution of 20% acetonitrile in water (v/v). The resulting products were analyzed by negative-mode ESI-MS.

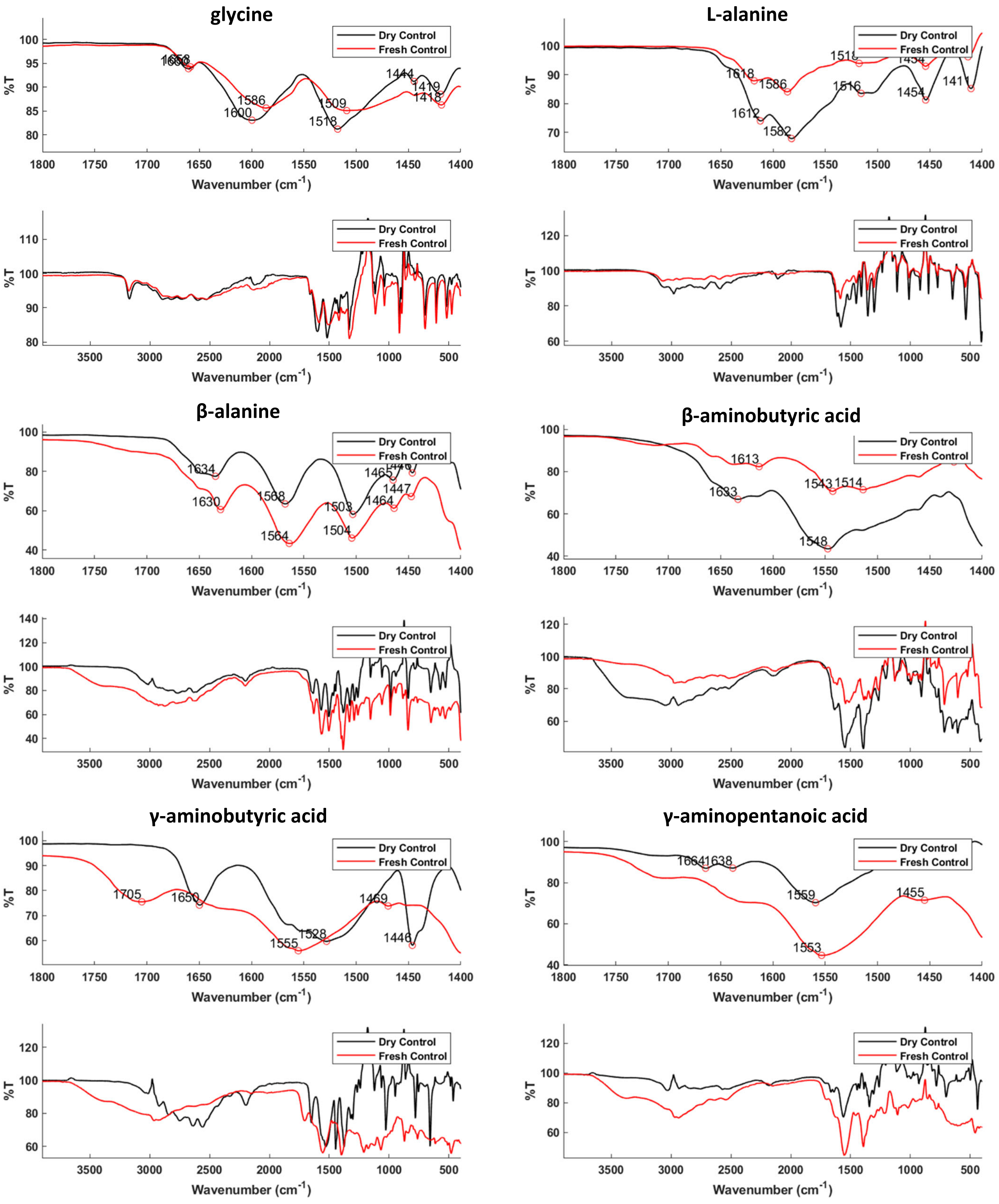

Supplementary Figure S93. FTIR spectrum of amino acid control samples.

In black samples that were dried for 7 days in 85 °C, in red fresh stock solution samples. Dry-down products were then resuspended in an aqueous solution of 20% acetonitrile in water (v/v).

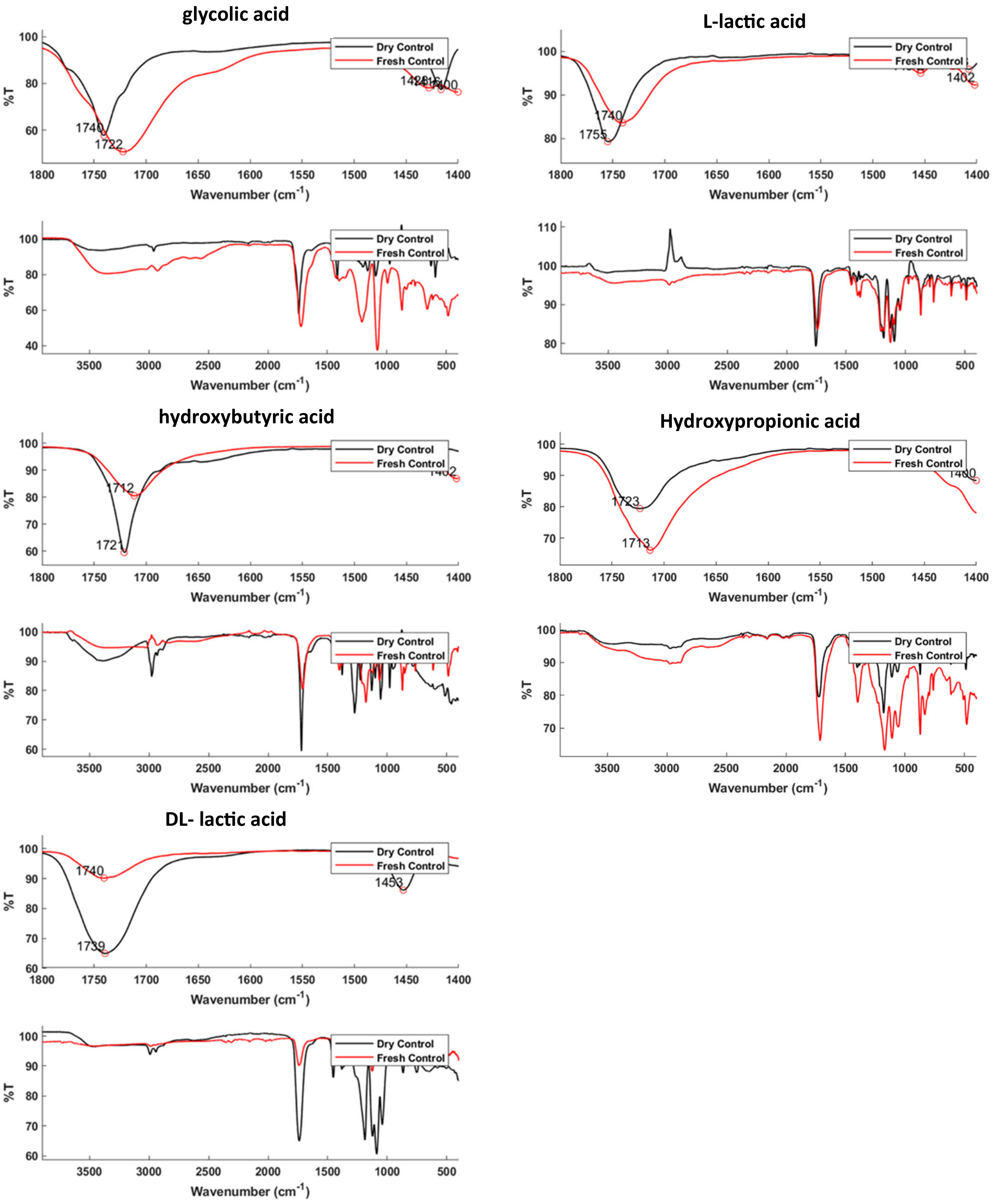

Supplementary Figure S94. FTIR spectrum of Hydroxy acid control samples. In black samples that were dried for 7 days in 85 °C, in red fresh stock solution samples. Dry down products were then resuspended in an aqueous solution of 20% acetonitrile in water (v/v).

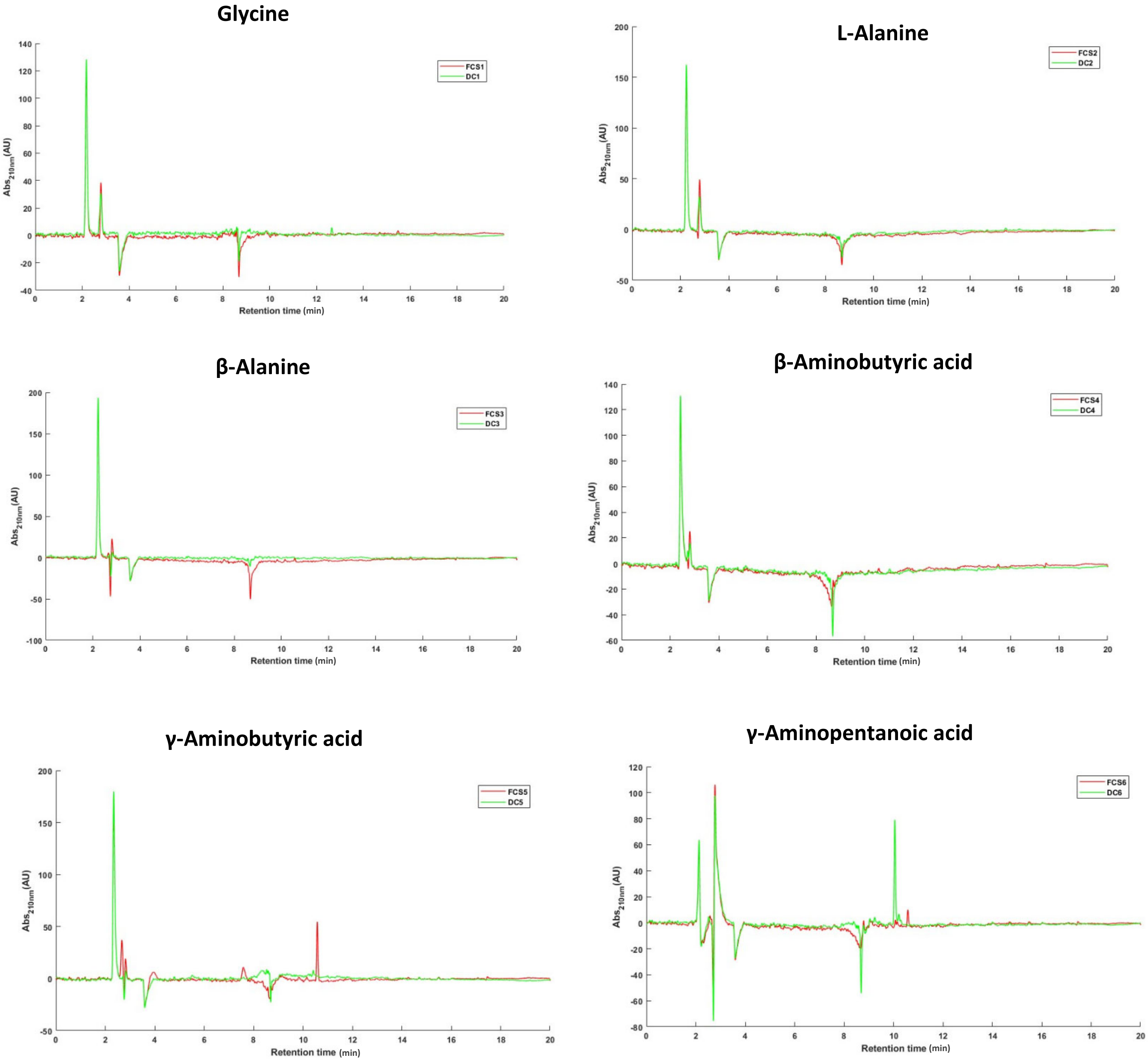

Supplementary Figure S95. HPLC of dry down and fresh amino acid control samples – Glycine, Alanine, β-Alanine, β-Aminobutyric acid, γ-Aminobutyric acid, γ-Aminopentanoic acid. Amino acid control dry downs at 50 umole each, for 7 days at 85 °C. Dry down products were then resuspended in an aqueous solution of 20% acetonitrile in water (v/v). The resulting products were analyzed by HPLC.

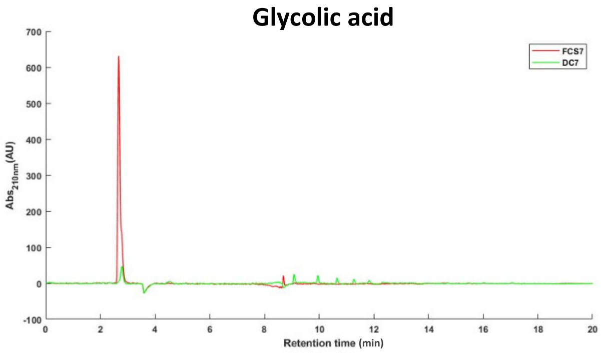

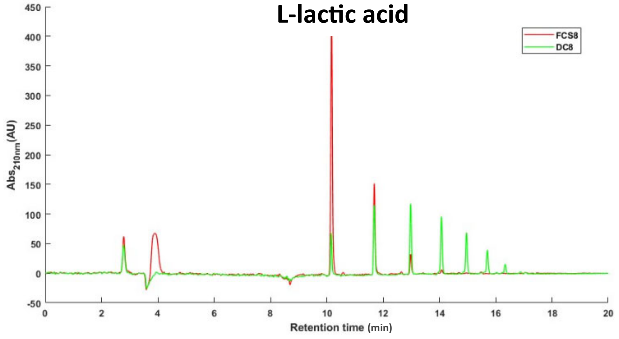

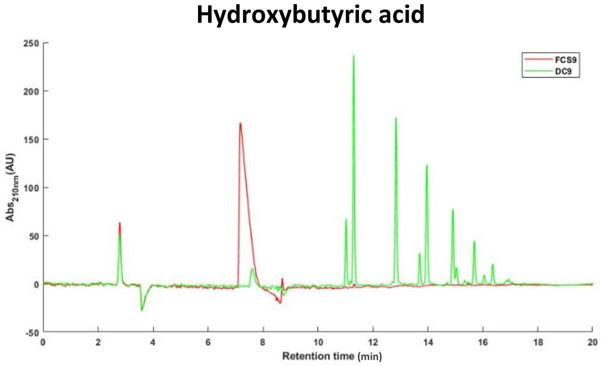

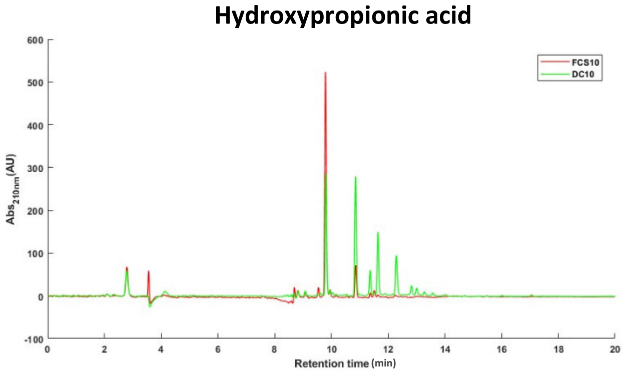

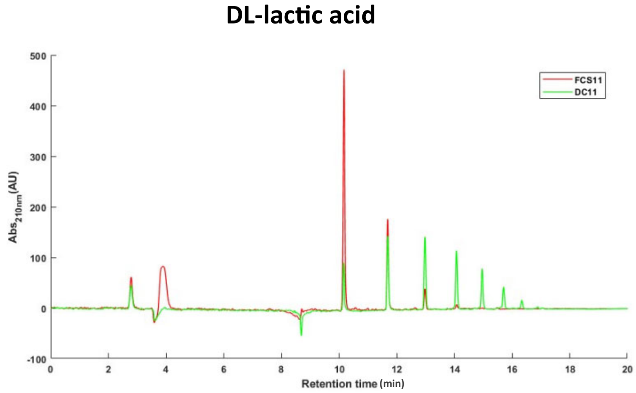

Supplementary Figure S96. HPLC of dry down and fresh Hydroxy acid control samples – Glycolic acid, L-lactic acid, Hydroxybutyric acid, Hydroxypropionic acid, DL-lactic acid. In green - dried down hydroxy acid controls 250 umole each, for 7 days at 85 °C. Dried products were then resuspended in an aqueous solution of 20% acetonitrile in water (v/v). In red – fresh monomers control samples. The resulting products were analyzed by HPLC.

A1→3

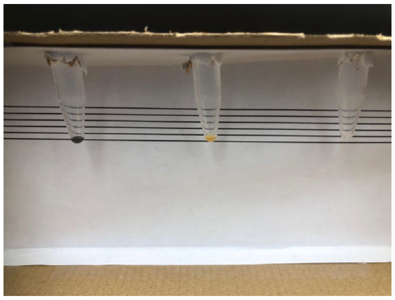

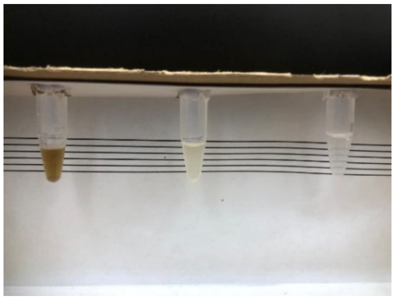

A4→6

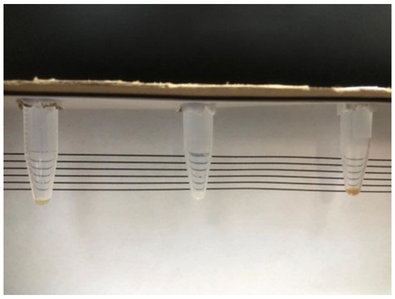

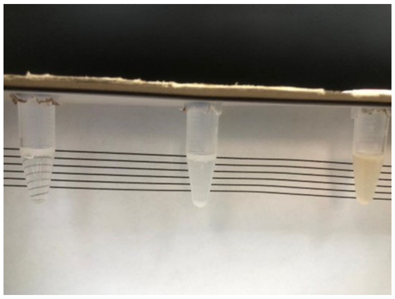

A7→9

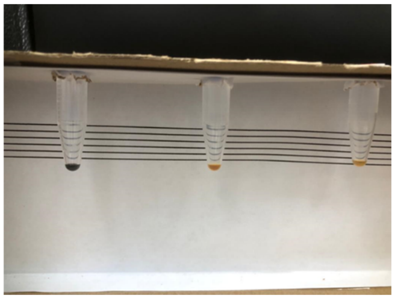

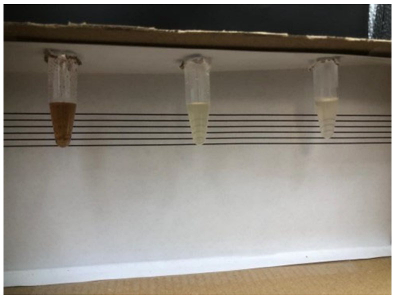

A10→12

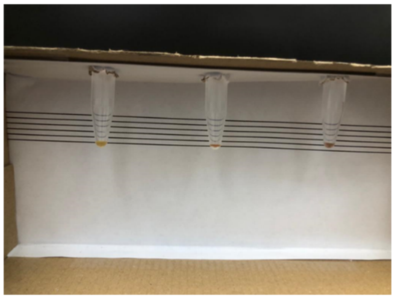

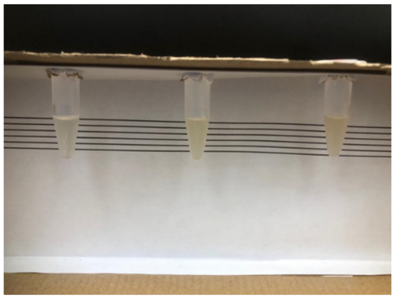

A13→15

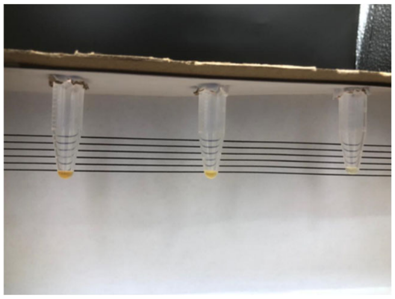

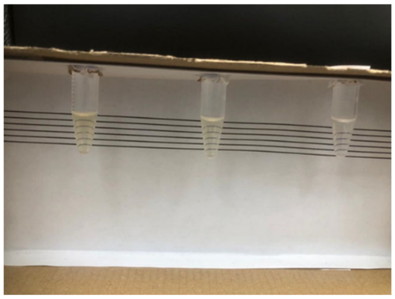

A16→18

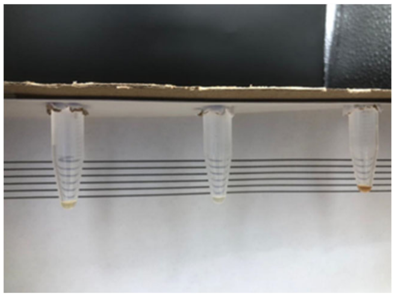

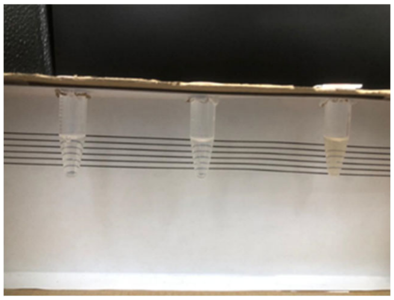

A19→21

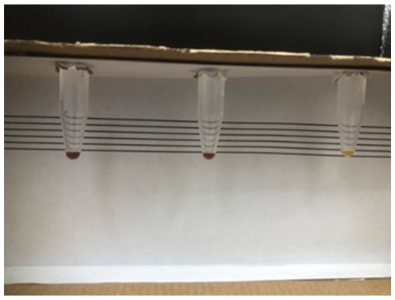

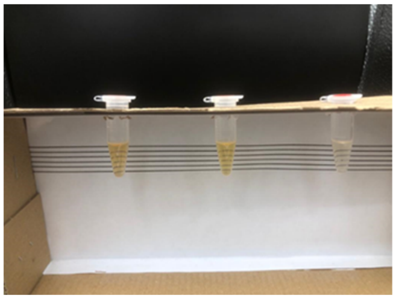

A22→24

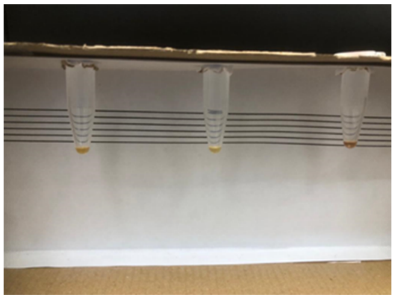

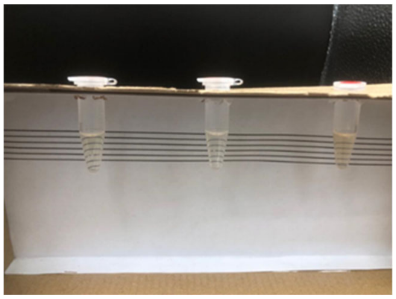

A25→27

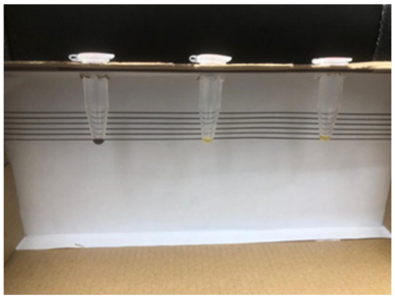

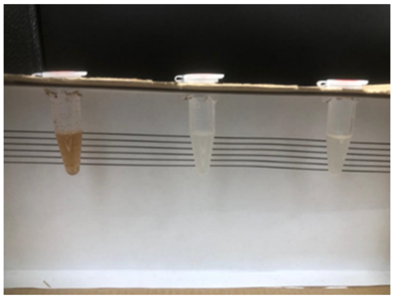

A28→30

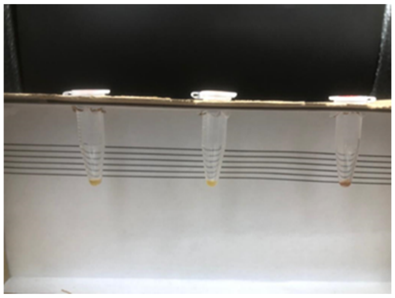

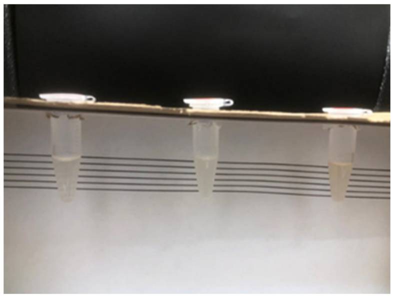

Supplementary Figure S97. – Visual appearance. Batch A sample images, coded A1-A30acorrding to the Table S1, after dried down for 7 days in 85 °C, before and after suspention, in a solution of 80% deionized water and 20% acetonitrile. (ACN), to 100mM concentration of amino acid (referring to initial amount prior to drying).

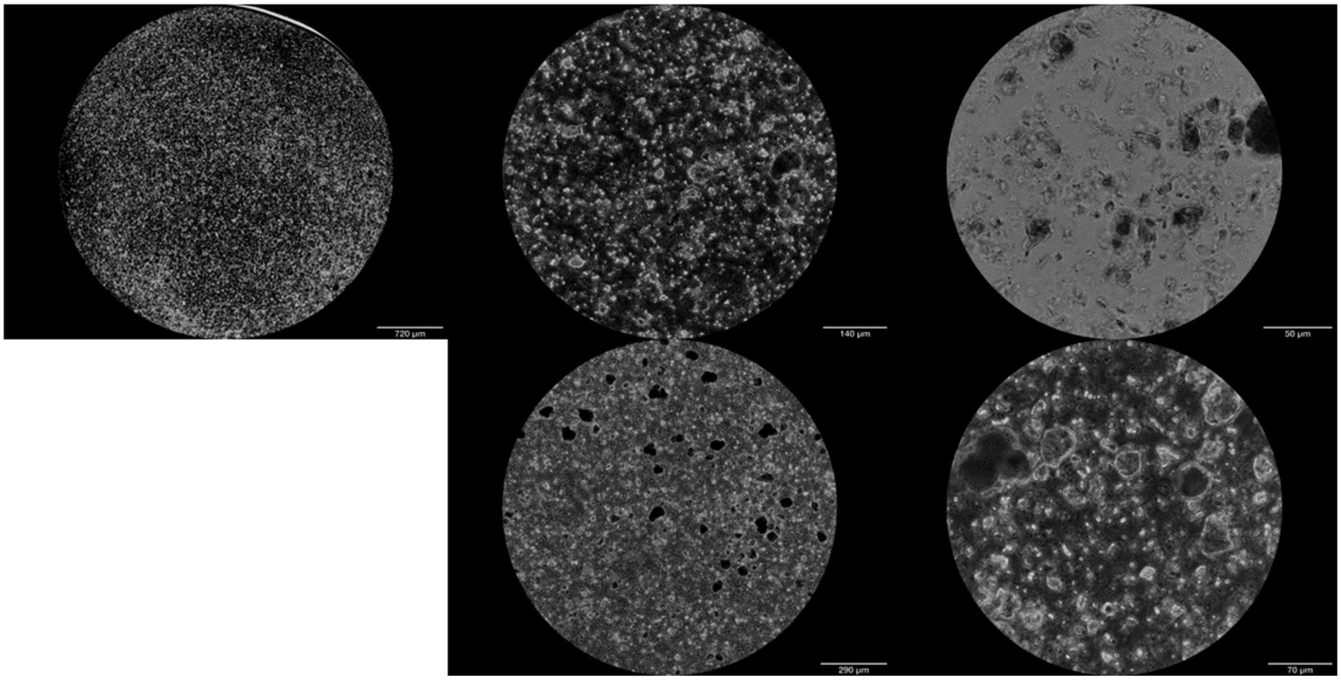

Supplementary Figure S98. Brightfield microscopy imaging of depsipeptides resulting from dry-down reactions between glycolic acid and Glycine. Microscopy of sample A1-glycolic acid + Glycine, resuspended in 80% deionized water and 20% acetonitrile (v/v), at five different magnifications- x4, x10, x20, x40 and x60.

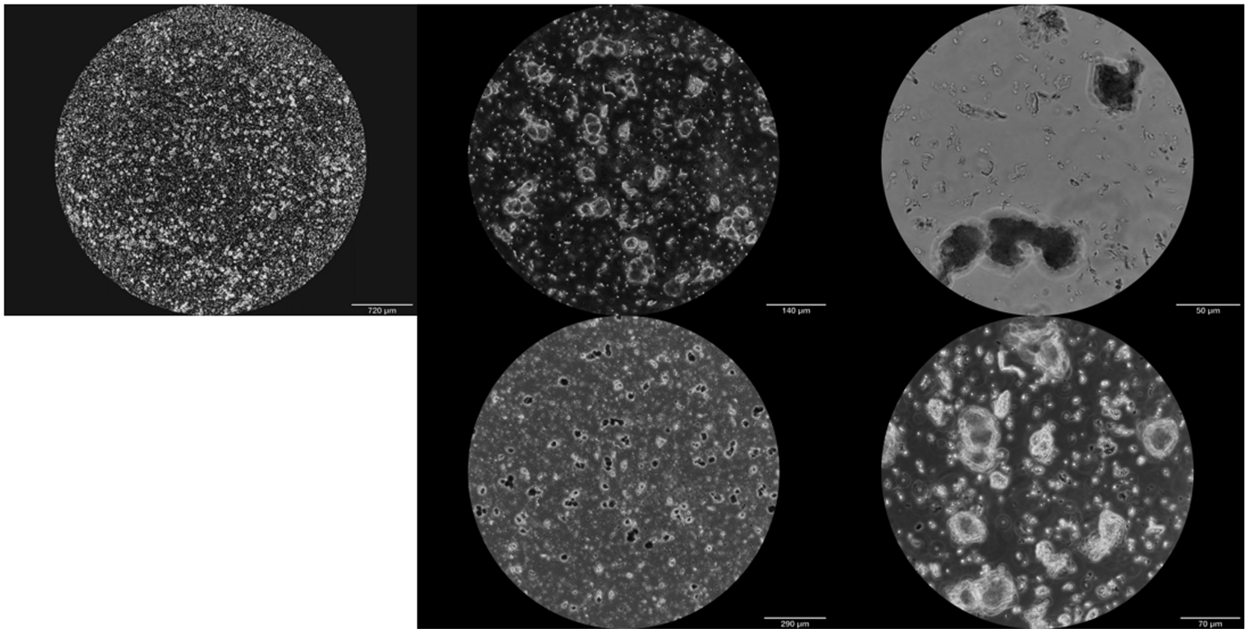

Supplementary Figure S99. Brightfield microscopy imaging of depsipeptides resulting from dry-down reactions between glycolic acid and L-alanine. Microscopy of sample A2-glycolic acid + alanine, resuspended in 80% deionized water and 20% acetonitrile (v/v), at five different magnifications- x4, x10, x20, x40 and x60.

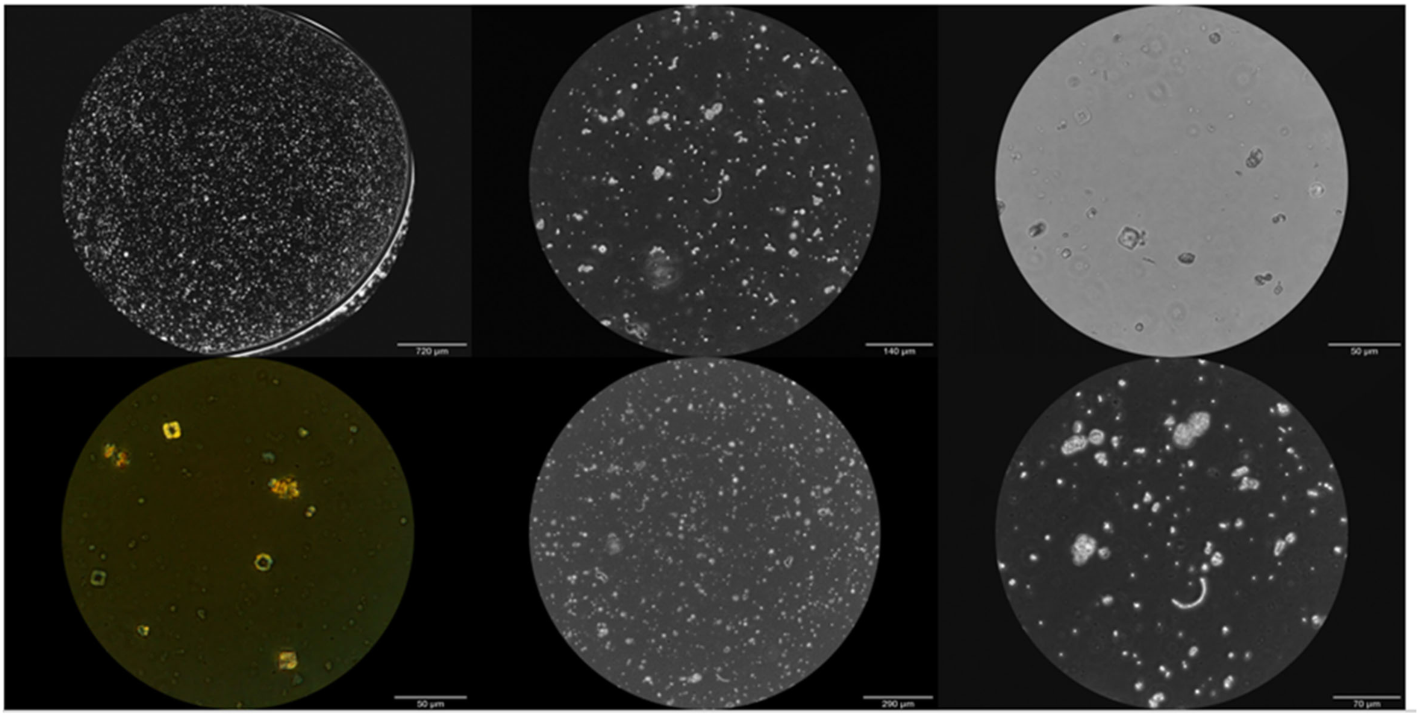

Supplementary Figure S100. Brightfield microscopy imaging of depsipeptides resulting from dry-down reactions between glycolic acid and β-alanine. Microscopy of sample A3-glycolic acid + β-alanine, resuspended in 80% deionized water and 20% acetonitrile (v/v), at five different magnifications - x4, x10, x20, x40 and x60.

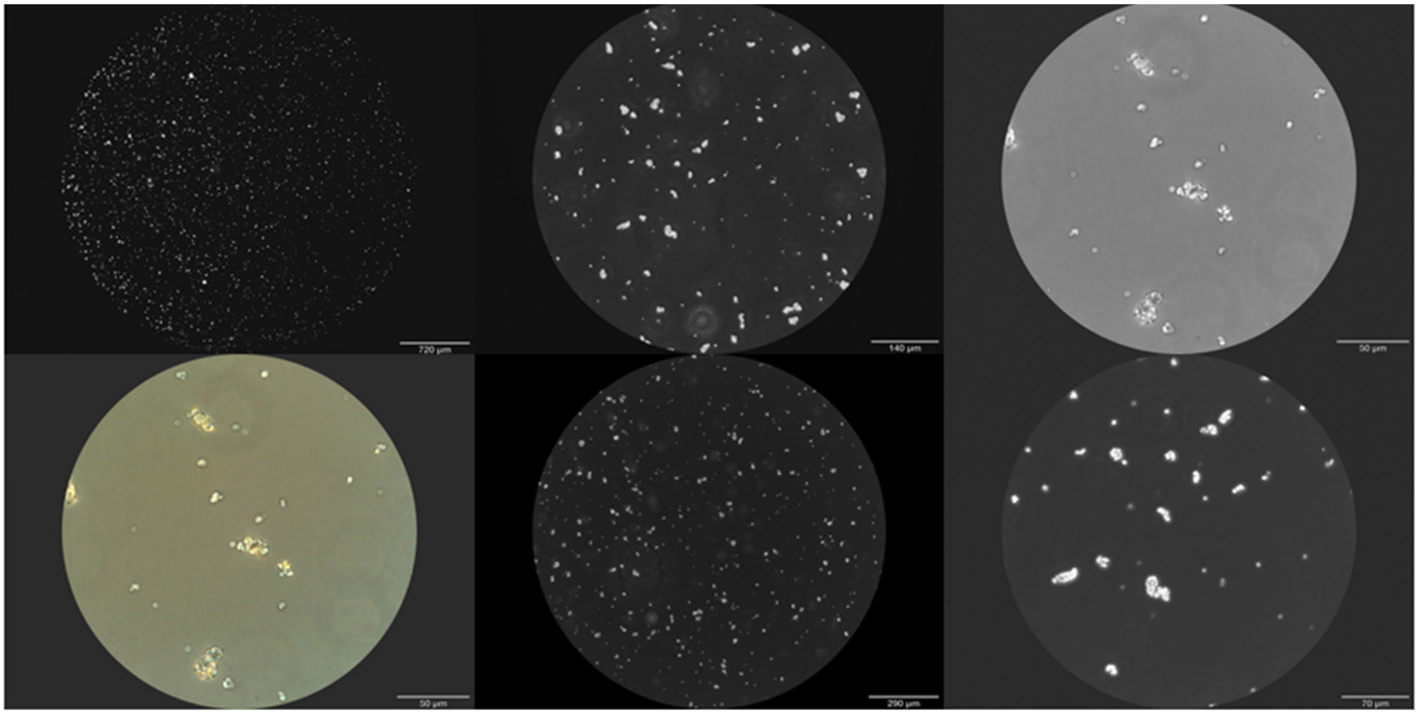

Supplementary Figure S101. Brightfield microscopy imaging of depsipeptides resulting from dry-down reactions between glycolic acid and β-aminobutyric acid. Microscopy of sample A4-glycolic acid + β-aminobutyric acid, resuspended in 80% deionized water and 20% acetonitrile (v/v), at five different magnifications- x4, x10, x20, x40 and x60.

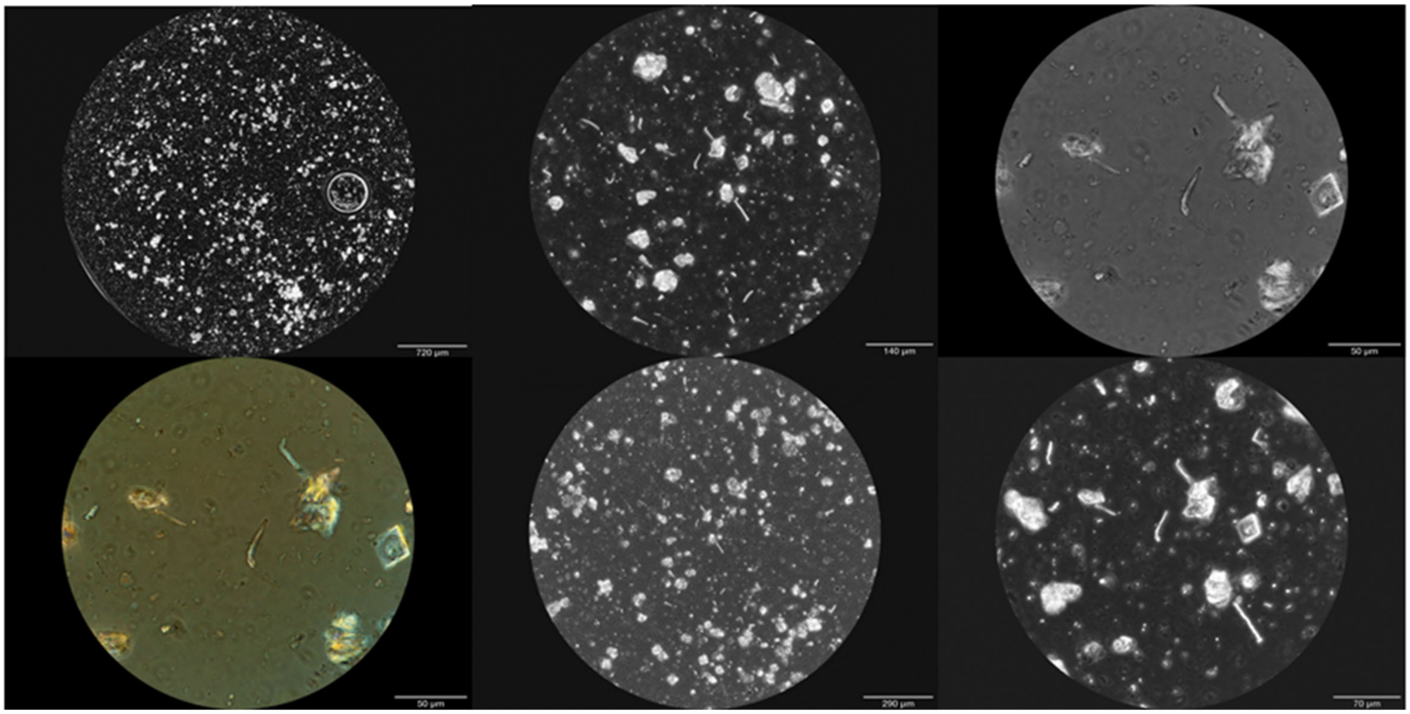

Supplementary Figure S102. Brightfield microscopy imaging of depsipeptides resulting from dry-down reactions between glycolic acid and γ-aminobutyric acid. Microscopy of sample A5-glycolic acid + γ-aminobutyric acid, resuspended in 80% deionized water and 20% acetonitrile (v/v), at five different magnifications- x4, x10, x20, x40 and x60.

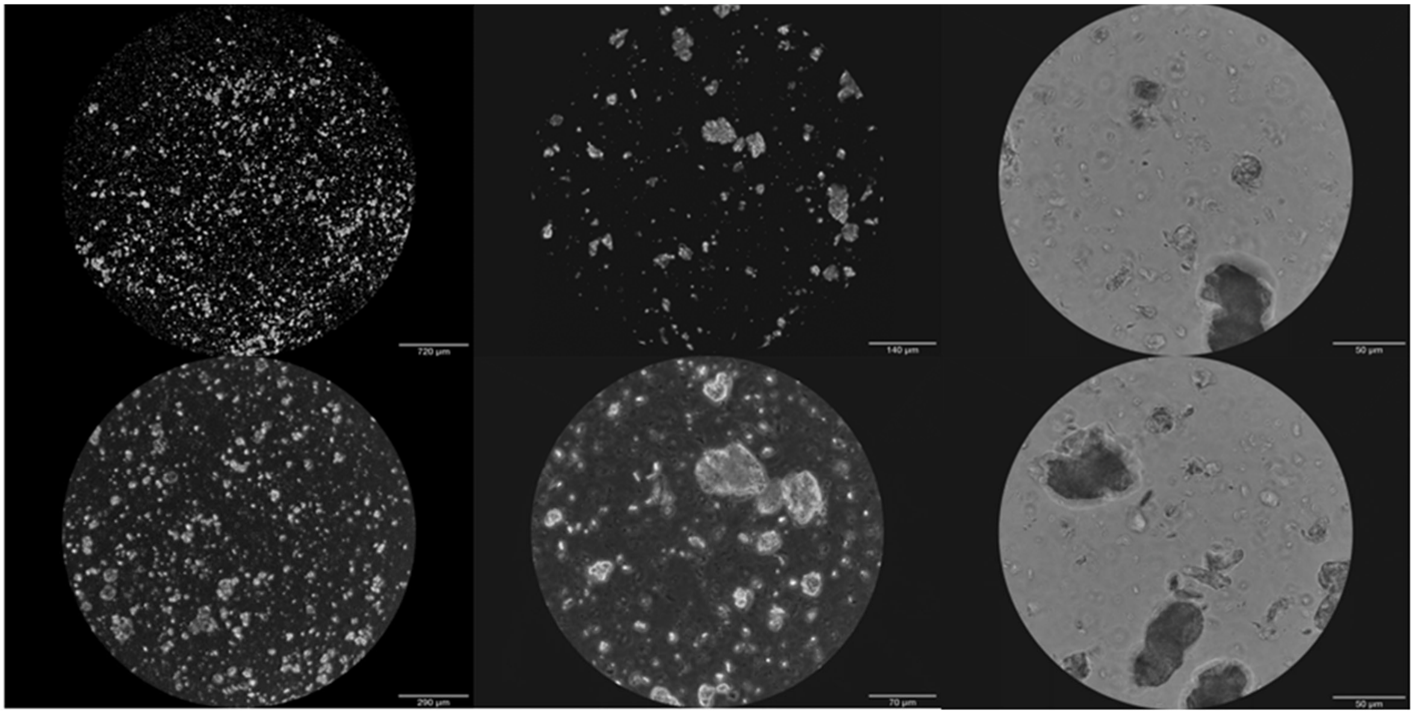

Supplementary Figure S103. Brightfield microscopy imaging of depsipeptides resulting from dry-down reactions between glycolic acid and γ-aminopentanoic acid. Microscopy of sample A6-glycolic acid + 4-pentanoic acid, resuspended in 80% deionized water and 20% acetonitrile (v/v), at five different magnifications- x4, x10, x20, x40 and x60.

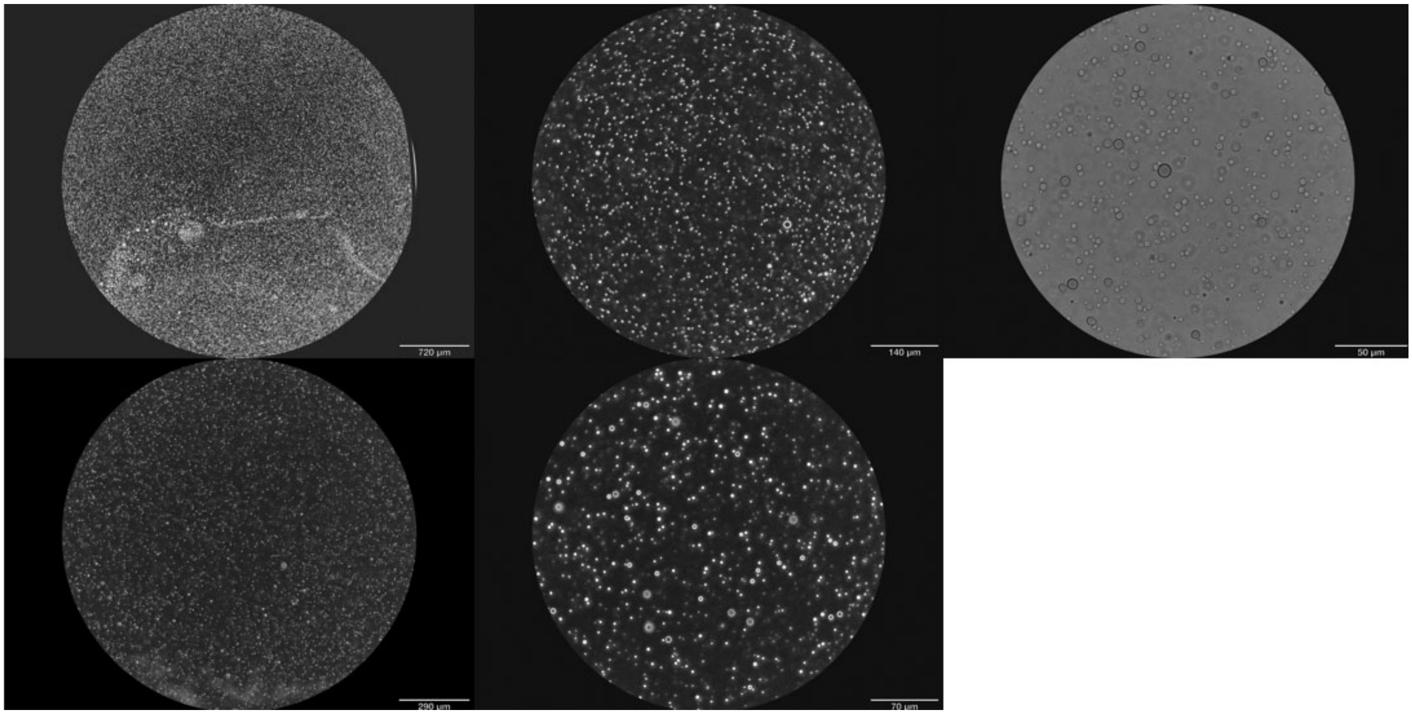

Supplementary Figure S104. Brightfield microscopy imaging of depsipeptides resulting from dry-down reactions between L-lactic acid and Glycine. Microscopy of sample A7-L-lactic acid + glycine, resuspended in 80% deionized water and 20% acetonitrile (v/v), at five different magnifications- x4, x10, x20, x40 and x60.

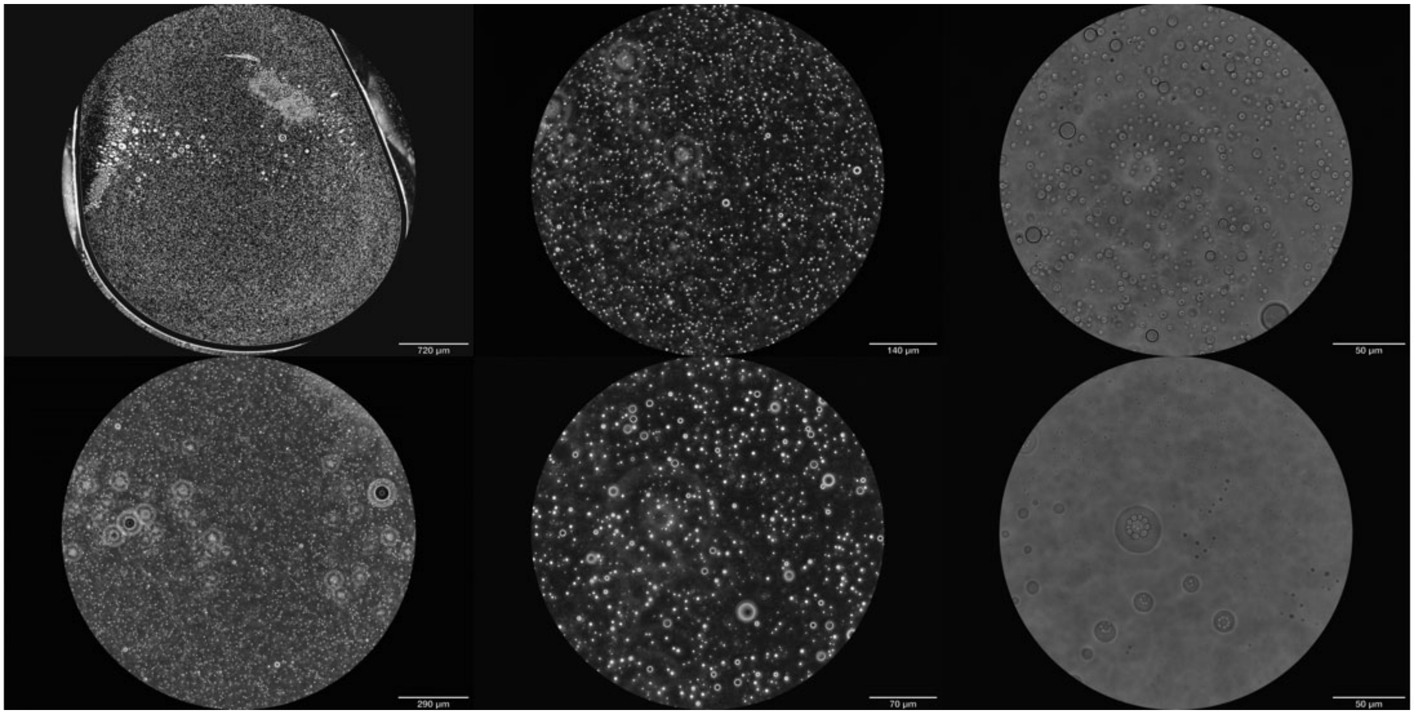

Supplementary Figure S105. Brightfield microscopy imaging of depsipeptides resulting from dry-down reactions between L-lactic acid and L-alanine. Microscopy of sample A8-L-lactic acid + L-alanine, resuspended in 80% deionized water and 20% acetonitrile (v/v), at five different magnifications- x4, x10, x20, x40 and x60.

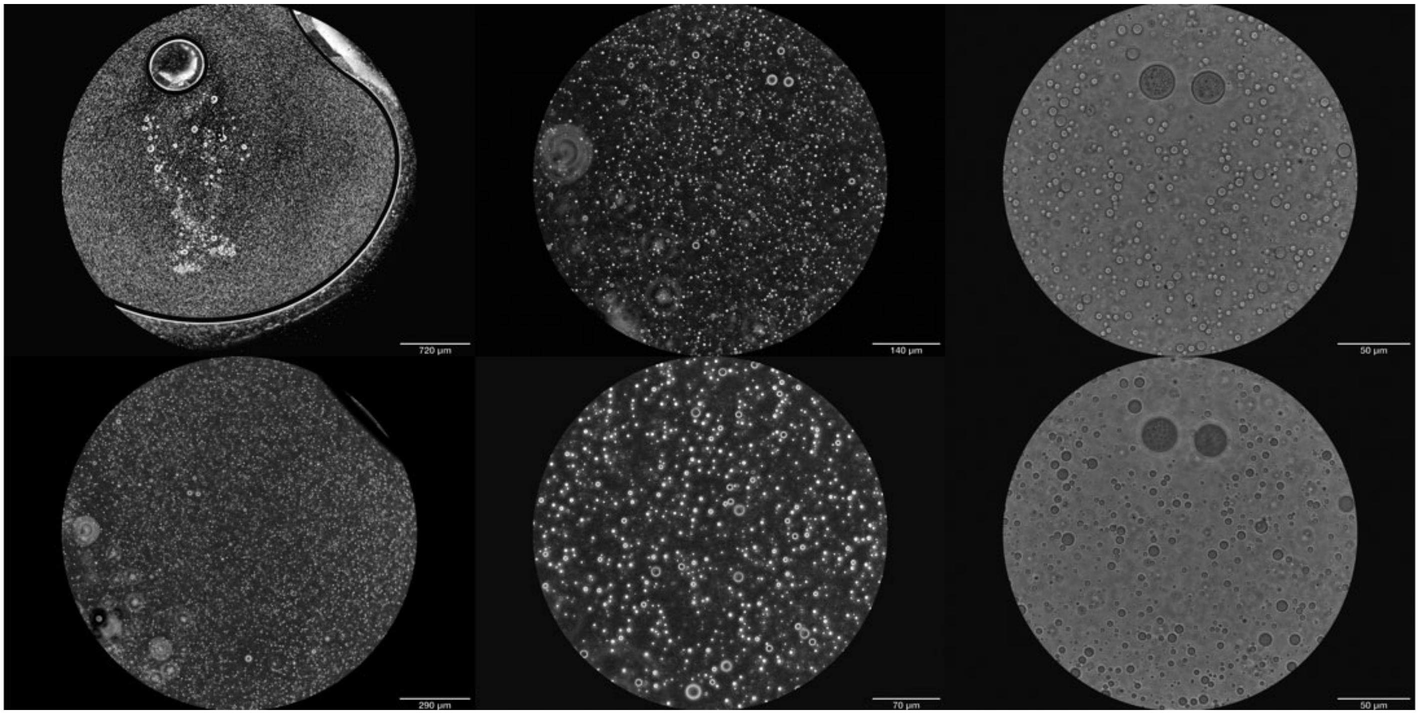

Supplementary Figure S106. Brightfield microscopy imaging of depsipeptides resulting from dry-down reactions between L-lactic acid and β-alanine. Microscopy of sample A9-L-lactic acid + β-alanine, resuspended in 80% deionized water and 20% acetonitrile (v/v), at five different magnifications- x4, x10, x20, x40 and x60.

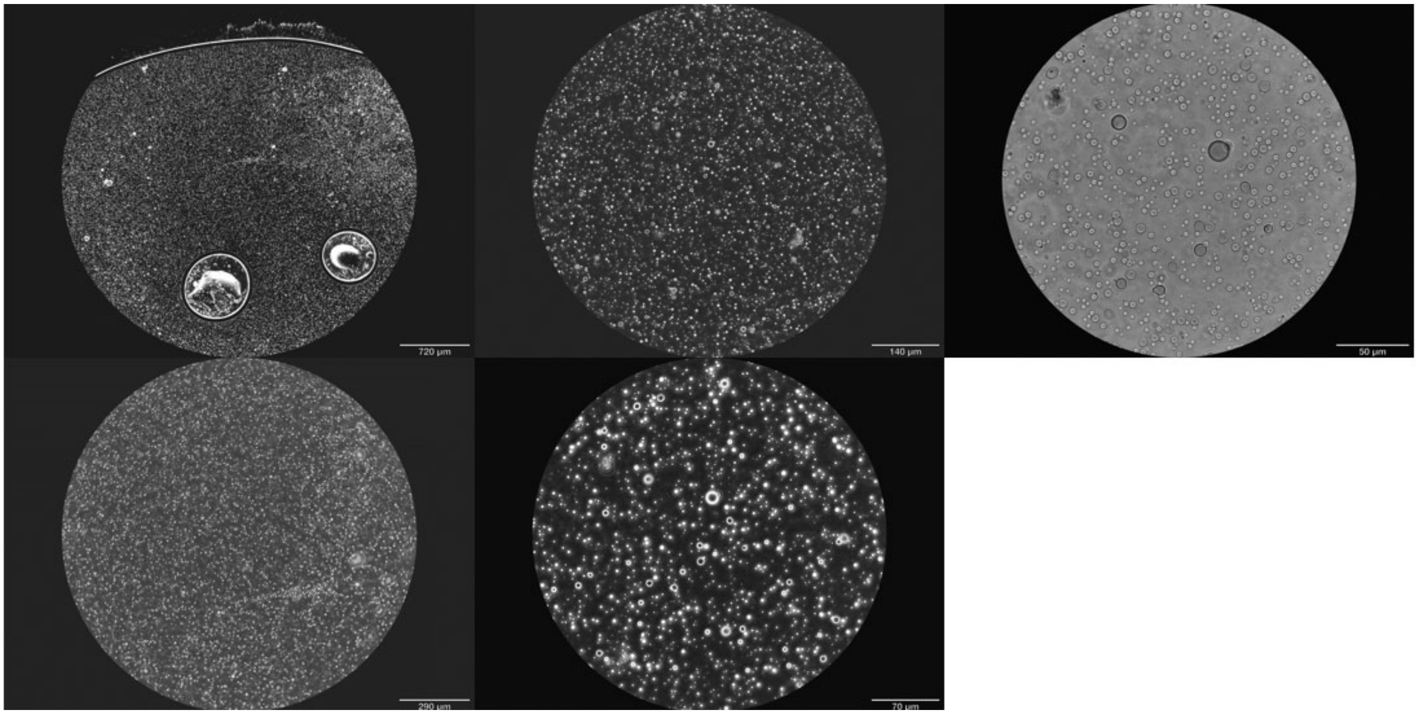

Supplementary Figure S107. Brightfield microscopy imaging of depsipeptides resulting from dry-down reactions between L-lactic acid and β-aminobutyric acid. Microscopy of sample A10-L-lactic acid + β-aminobutyric acid, resuspended in 80% deionized water and 20% acetonitrile (v/v), at five different magnifications- x4, x10, x20, x40 and x60.

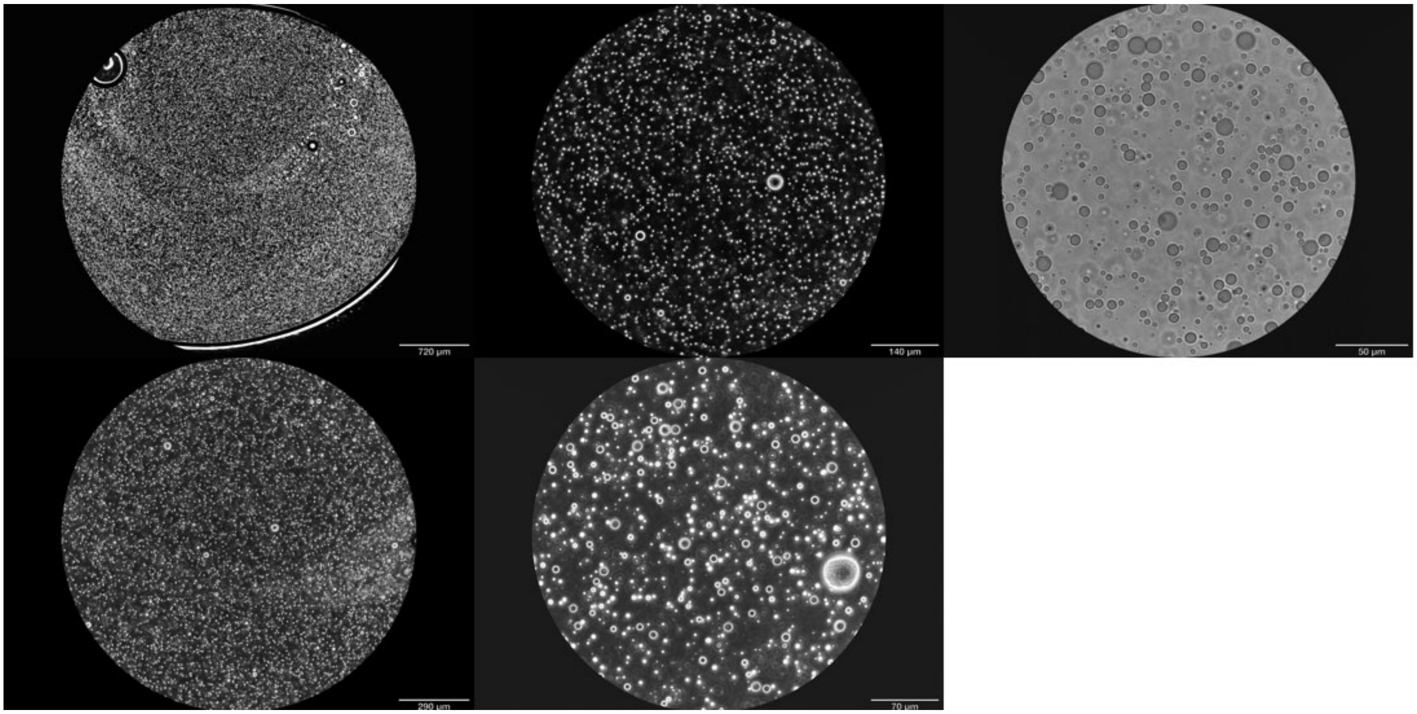

Supplementary Figure S108. Brightfield microscopy imaging of depsipeptides resulting from dry-down reactions between L-lactic acid and γ-aminobutyric acid. Microscopy of sample A11-L-lactic acid + γ-aminobutyric acid, resuspended in 80% deionized water and 20% acetonitrile (v/v), at five different magnifications- x4, x10, x20, x40 and x60.

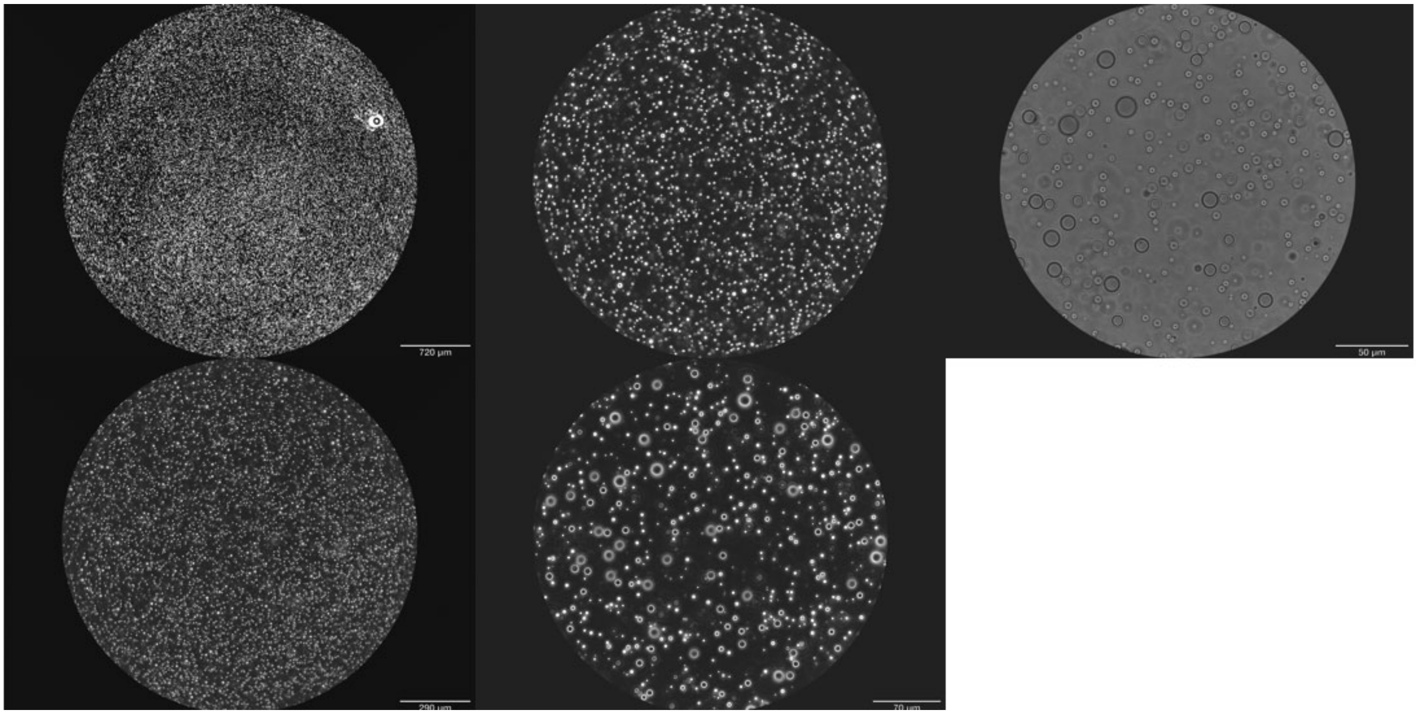

Supplementary Figure S109. Brightfield microscopy imaging of depsipeptides resulting from dry-down reactions between L-lactic acid and γ-aminopentanoic acid. Microscopy of sample A12-L-lactic acid + 4-aminopentanoic acid, resuspended in 80% deionized water and 20% acetonitrile (v/v), at five different magnifications- x4, x10, x20, x40 and x60.

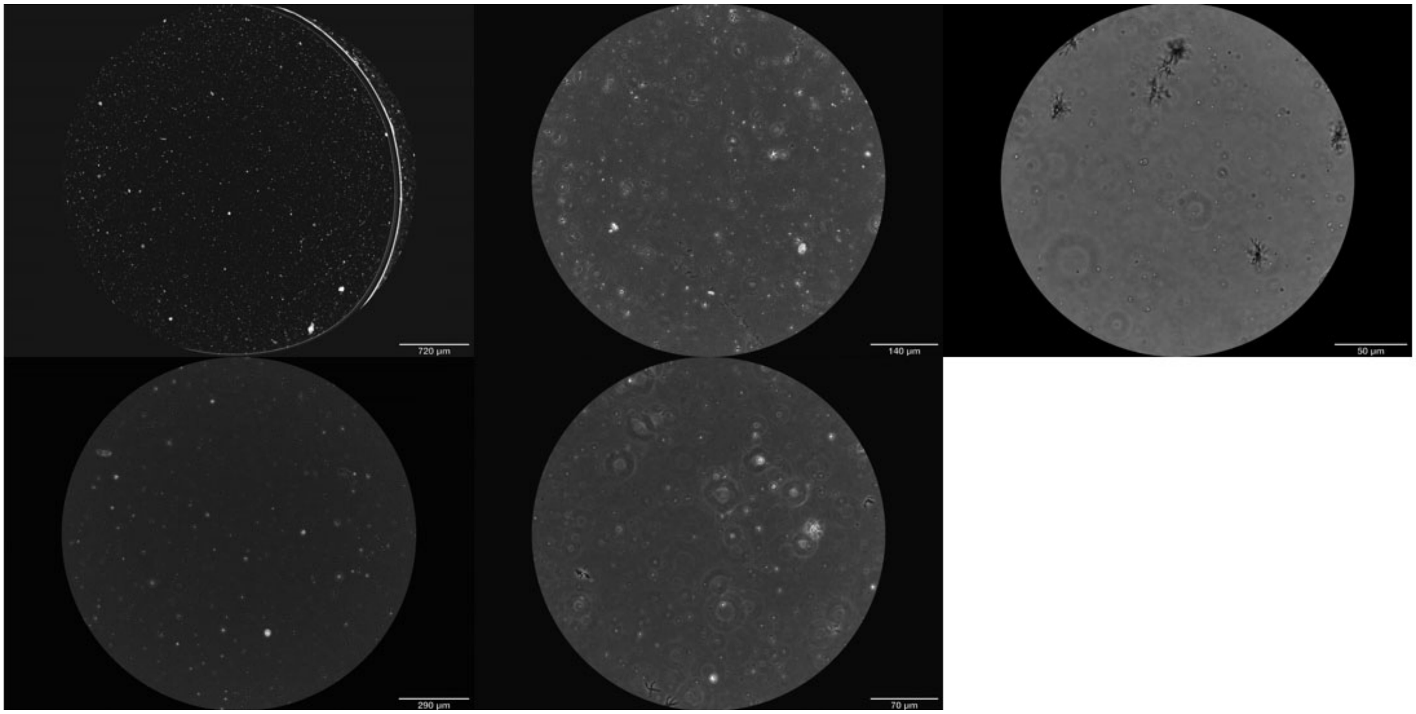

Supplementary Figure S110. Brightfield microscopy imaging of depsipeptides resulting from dry-down reactions between Hydroxybutyric acid and glycine. Microscopy of sample A13- Hydroxybutyric acid + glycine, resuspended in 80% deionized water and 20% acetonitrile (v/v), at five different magnifications- x4, x10, x20, x40 and x60.

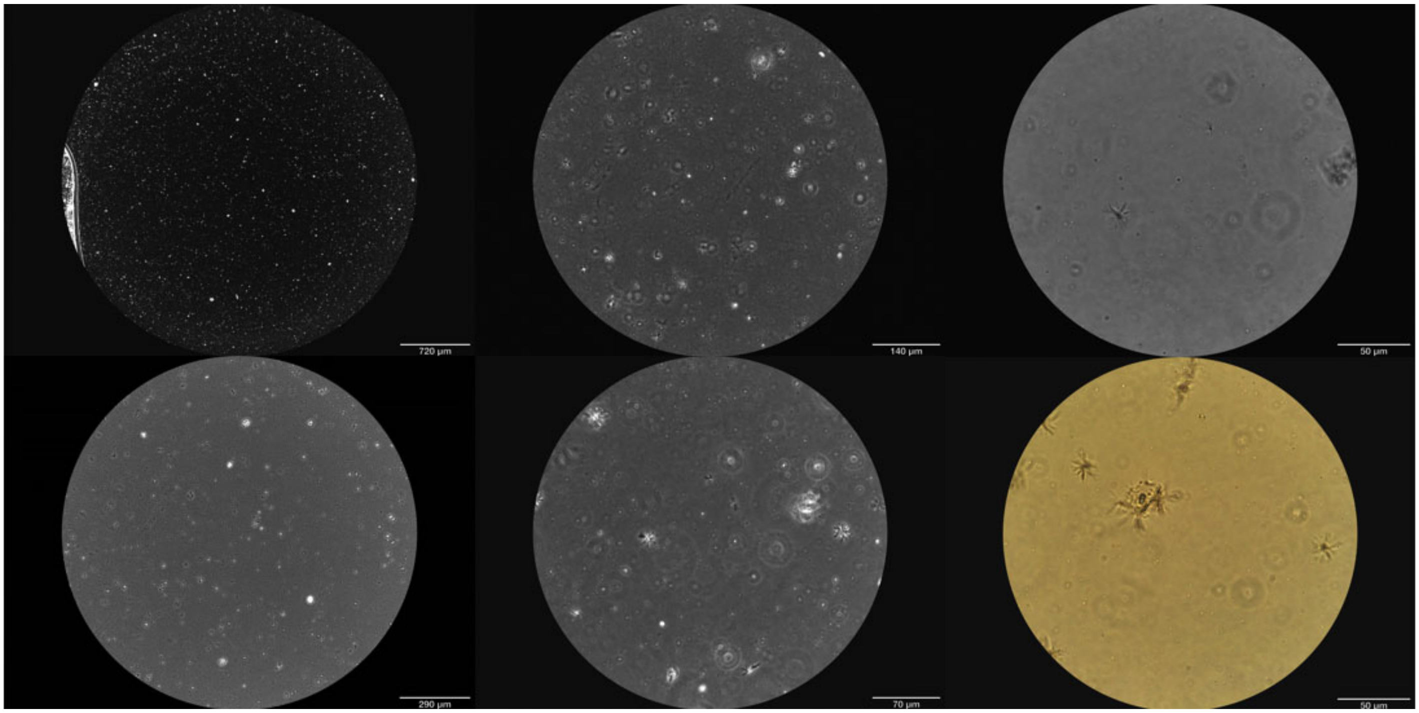

Supplementary Figure S111. Brightfield microscopy imaging of depsipeptides resulting from dry-down reactions between Hydroxybutyric acid and L-alanine. Microscopy of sample A14- Hydroxybutyric acid + L-alanine, resuspended in 80% deionized water and 20% acetonitrile (v/v), at five different magnifications- x4, x10, x20, x40 and x60.

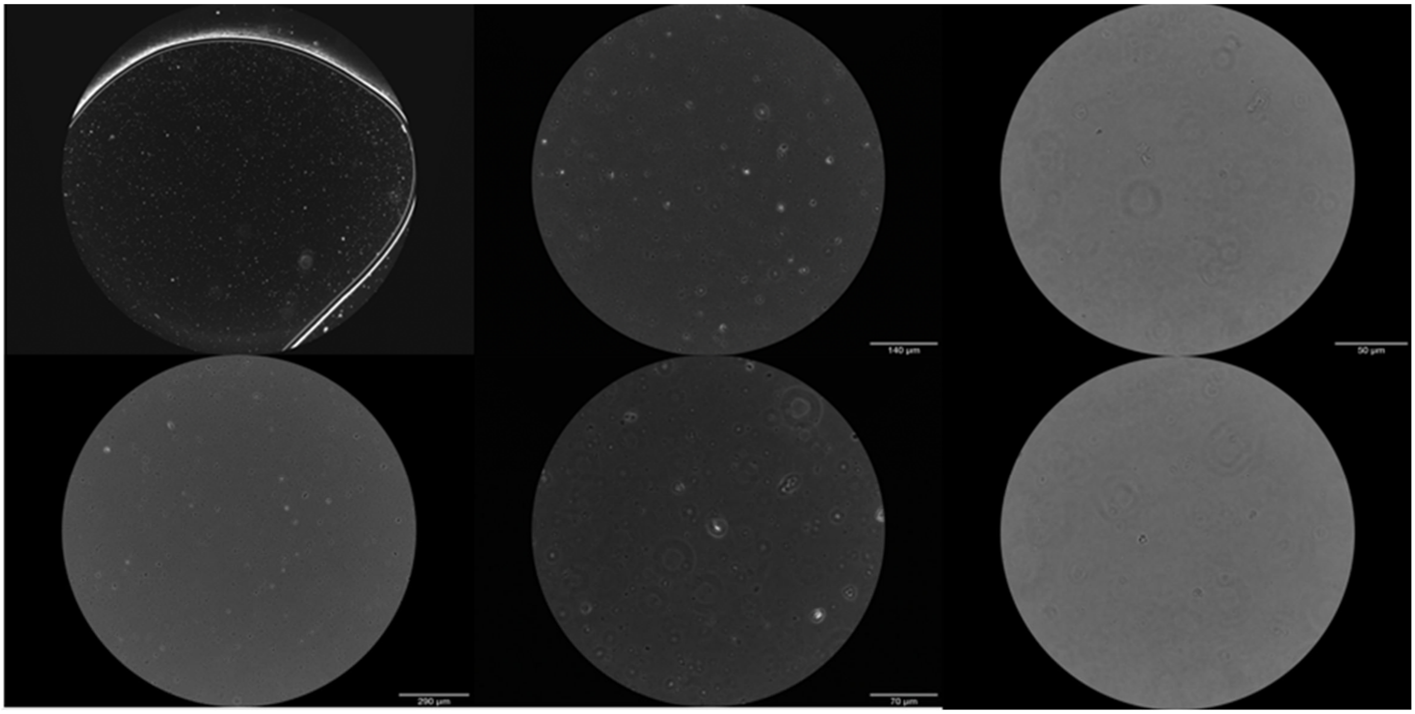

Supplementary Figure S112. Brightfield microscopy imaging of depsipeptides resulting from dry-down reactions between Hydroxybutyric acid and β-alanine. Microscopy of sample A15- Hydroxybutyric acid + β-alanine, resuspended in 80% deionized water and 20% acetonitrile (v/v), at five different magnifications- x4, x10, x20, x40 and x60.

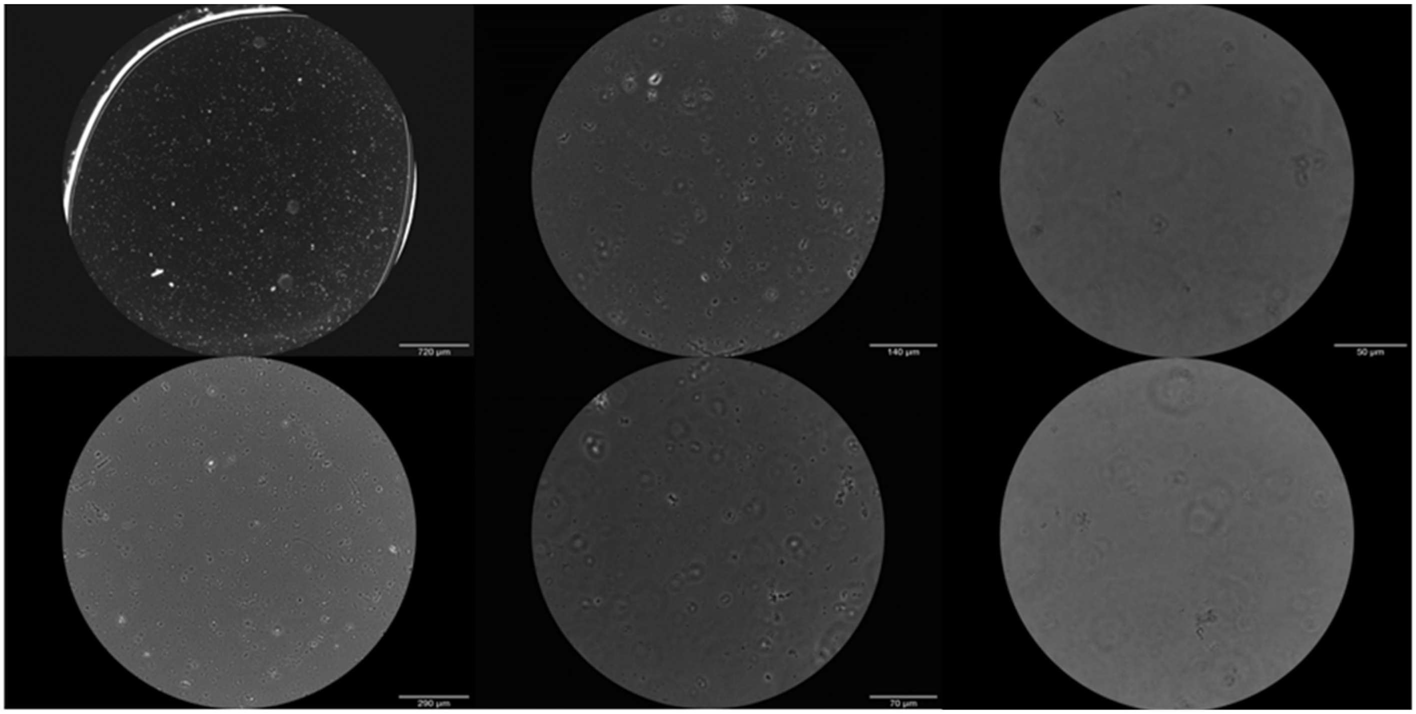

Supplementary Figure S113. Brightfield microscopy imaging of depsipeptides resulting from dry-down reactions between Hydroxybutyric acid and β-aminobutyric acid. Microscopy of sample A16- Hydroxybutyric acid + β-aminobutyric acid, resuspended in 80% deionized water and 20% acetonitrile (v/v), at five different magnifications- x4, x10, x20, x40 and x60.

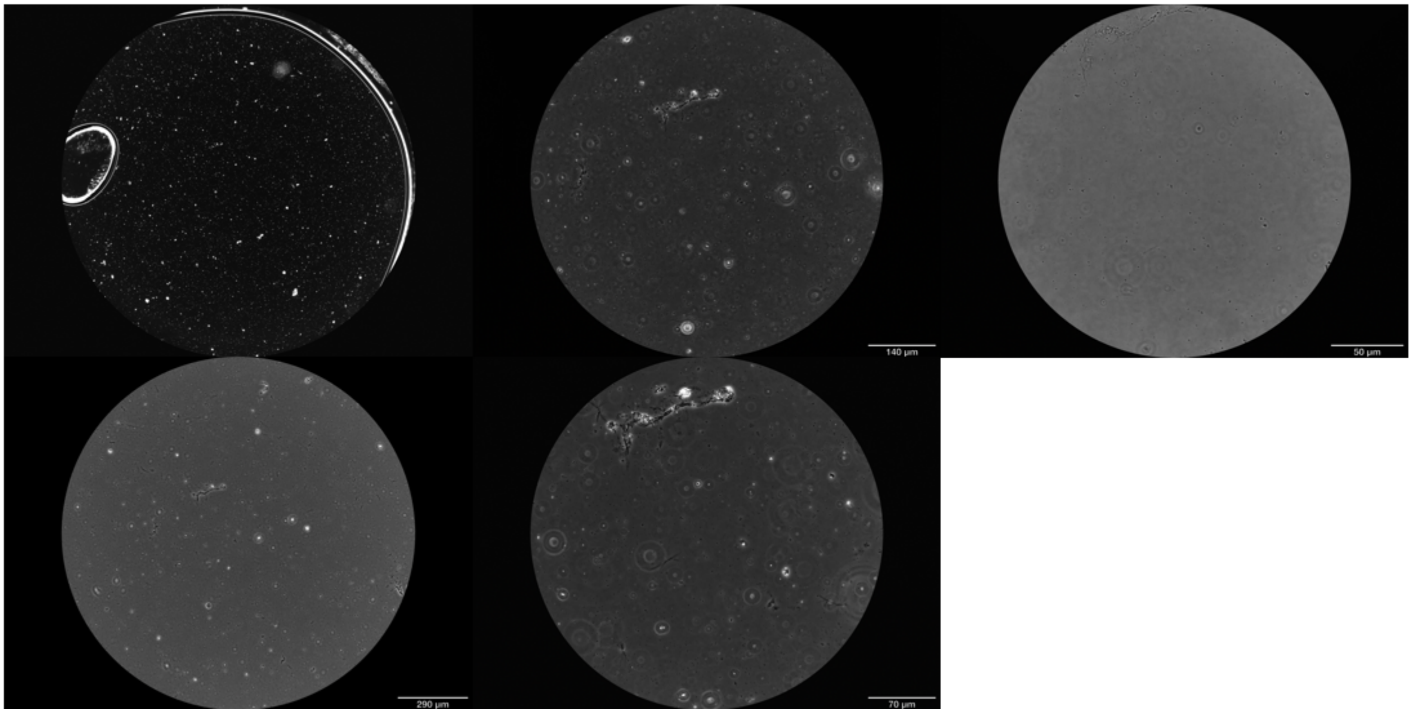

Supplementary Figure S114. Brightfield microscopy imaging of depsipeptides resulting from dry-down reactions between Hydroxybutyric acid and γ-aminobutyric acid. Microscopy of sample A17- Hydroxybutyric acid + γ-aminobutyric acid, resuspended in 80% deionized water and 20% acetonitrile (v/v), at five different magnifications- x4, x10, x20, x40 and x60.

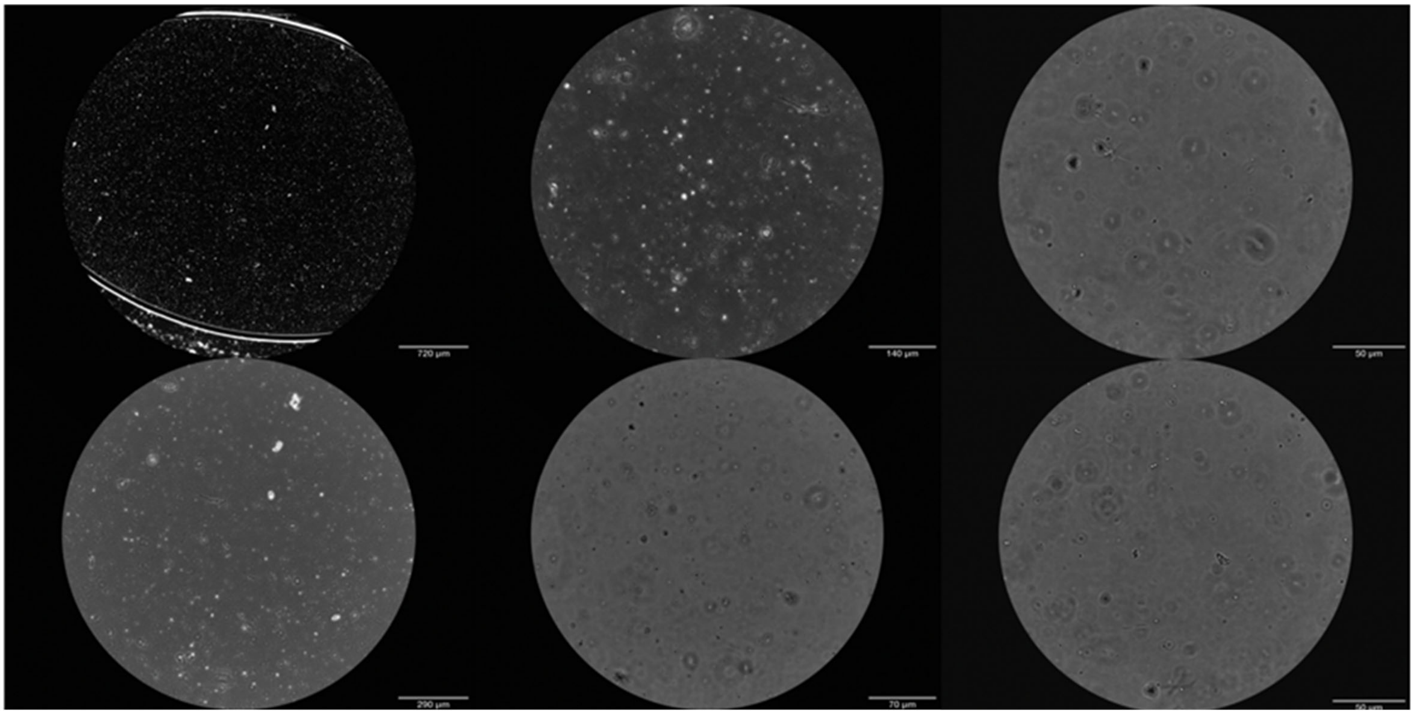

Supplementary Figure S115. Brightfield microscopy imaging of depsipeptides resulting from dry-down reactions between Hydroxybutyric acid and γ-aminopentanoic acid. Microscopy of sample A18- Hydroxybutyric acid + γ-aminopentanoic acid, resuspended in 80% deionized water and 20% acetonitrile (v/v), at five different magnifications- x4, x10, x20, x40 and x60.

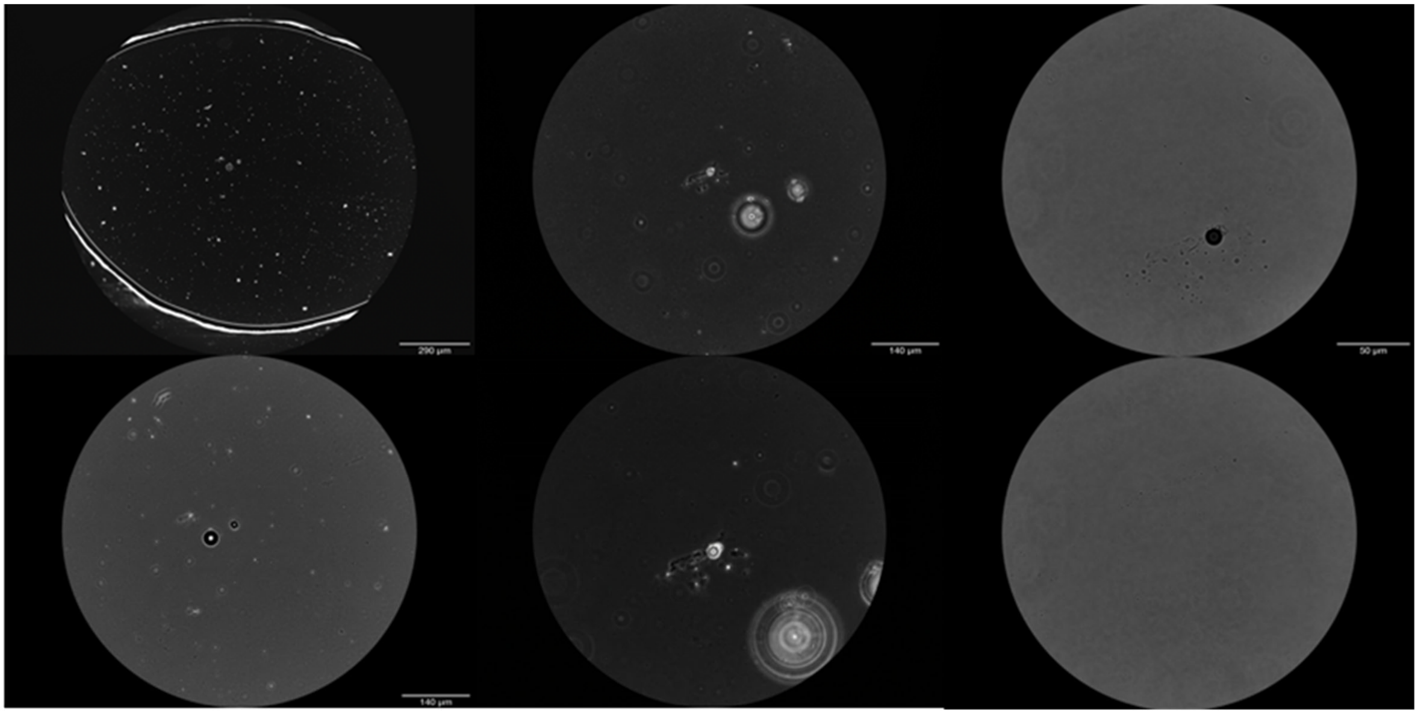

Supplementary Figure S116. Brightfield microscopy imaging of depsipeptides resulting from dry-down reactions between Hydroxypropionic acid and glycine. Microscopy of sample A19- Hydroxypropionic acid + glycine, resuspended in 80% deionized water and 20% acetonitrile (v/v), at five different magnifications- x4, x10, x20, x40 and x60.

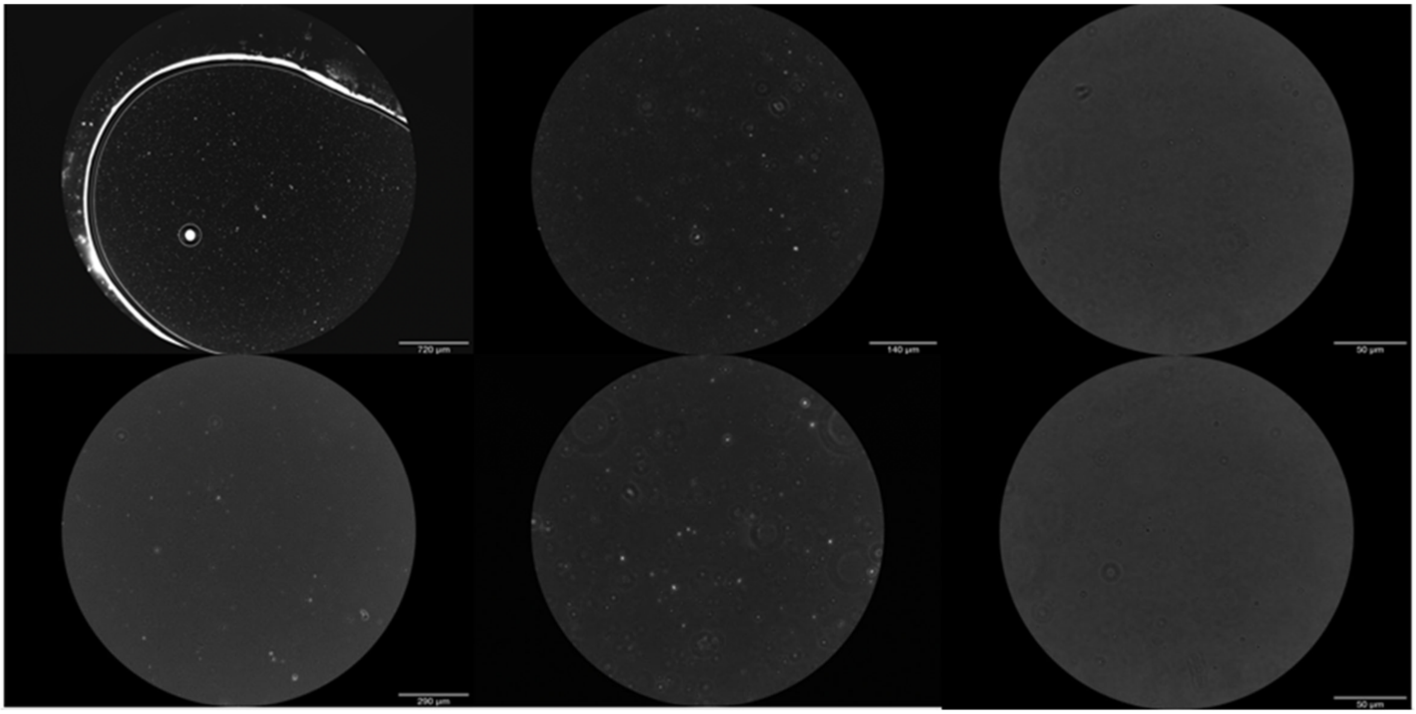

Supplementary Figure S117. Brightfield microscopy imaging of depsipeptides resulting from dry-down reactions between Hydroxypropionic acid and L-alanine. Microscopy of sample A20- Hydroxypropionic acid + L-alanine, resuspended in 80% deionized water and 20% acetonitrile (v/v), at five different magnifications- x4, x10, x20, x40 and x60.

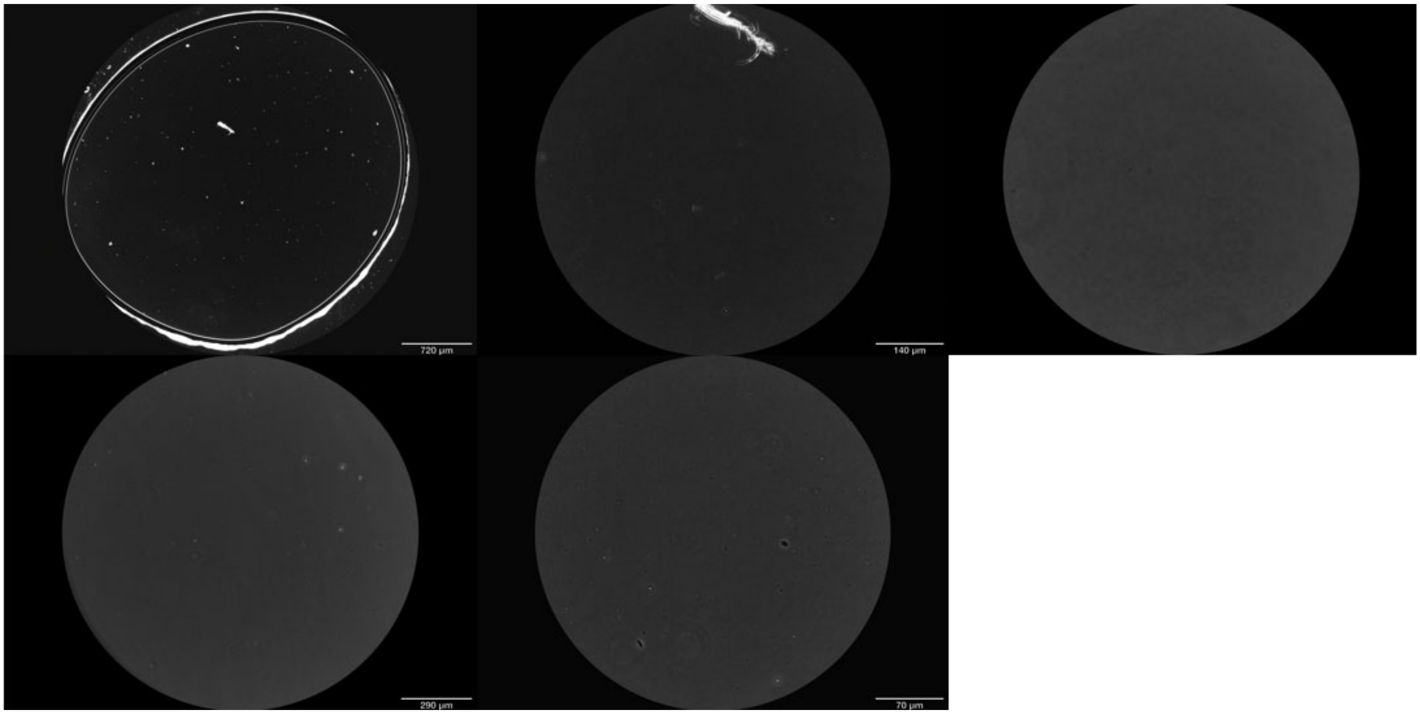

Supplementary Figure S118. Brightfield microscopy imaging of depsipeptides resulting from dry-down reactions between Hydroxypropionic acid and β-alanine. Microscopy of sample A21- Hydroxypropionic acid + β-alanine, resuspended in 80% deionized water and 20% acetonitrile (v/v), at five different magnifications- x4, x10, x20, x40 and x60.

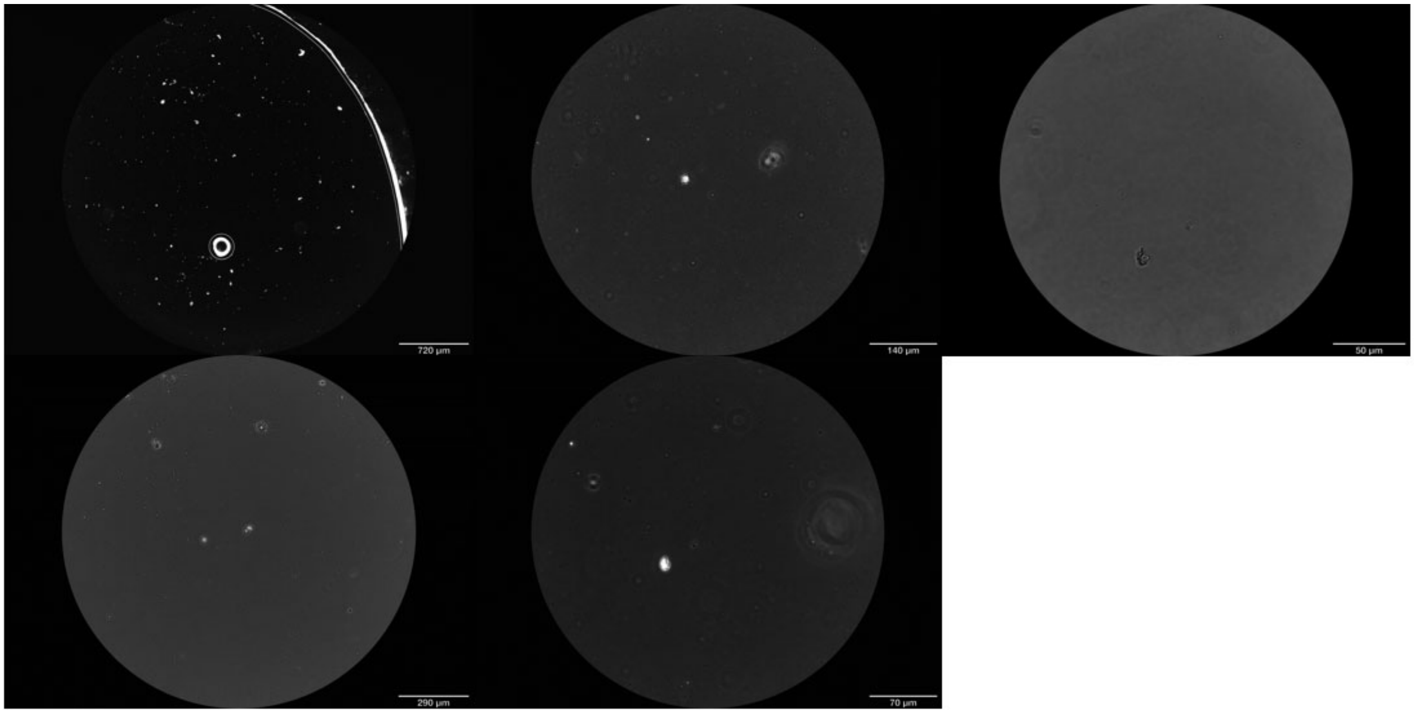

Supplementary Figure S119. Brightfield microscopy imaging of depsipeptides resulting from dry-down reactions between Hydroxypropionic acid and β-aminobutyric acid. Microscopy of sample A22-Hydroxypropionic acid + β-aminobutyric acid, resuspended in 80% deionized water and 20% acetonitrile (v/v), at five different magnifications- x4, x10, x20, x40 and x60.

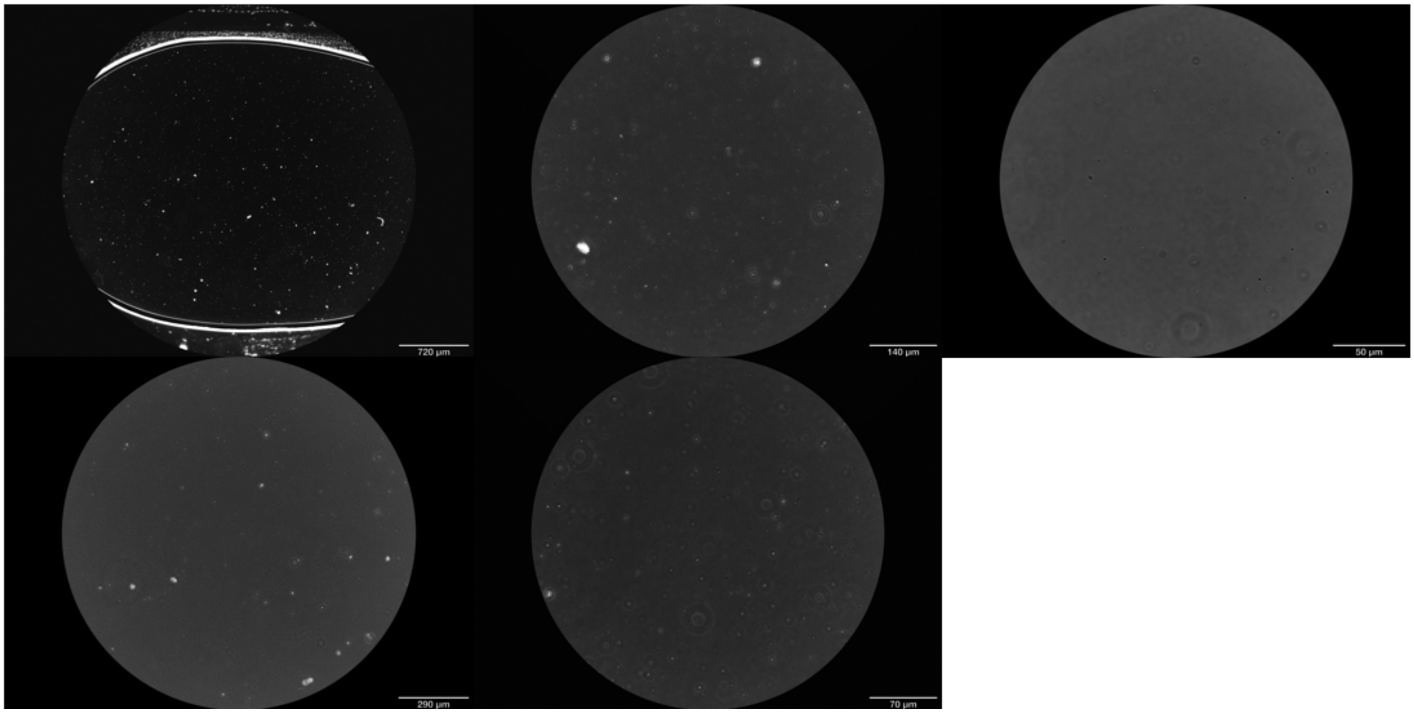

Supplementary Figure S120. Brightfield microscopy imaging of depsipeptides resulting from dry-down reactions between Hydroxypropionic acid and γ-aminobutyric acid. Microscopy of sample A23-Hydroxypropionic acid + γ-aminobutyric acid, resuspended in 80% deionized water and 20% acetonitrile (v/v), at five different magnifications- x4, x10, x20, x40 and x60.

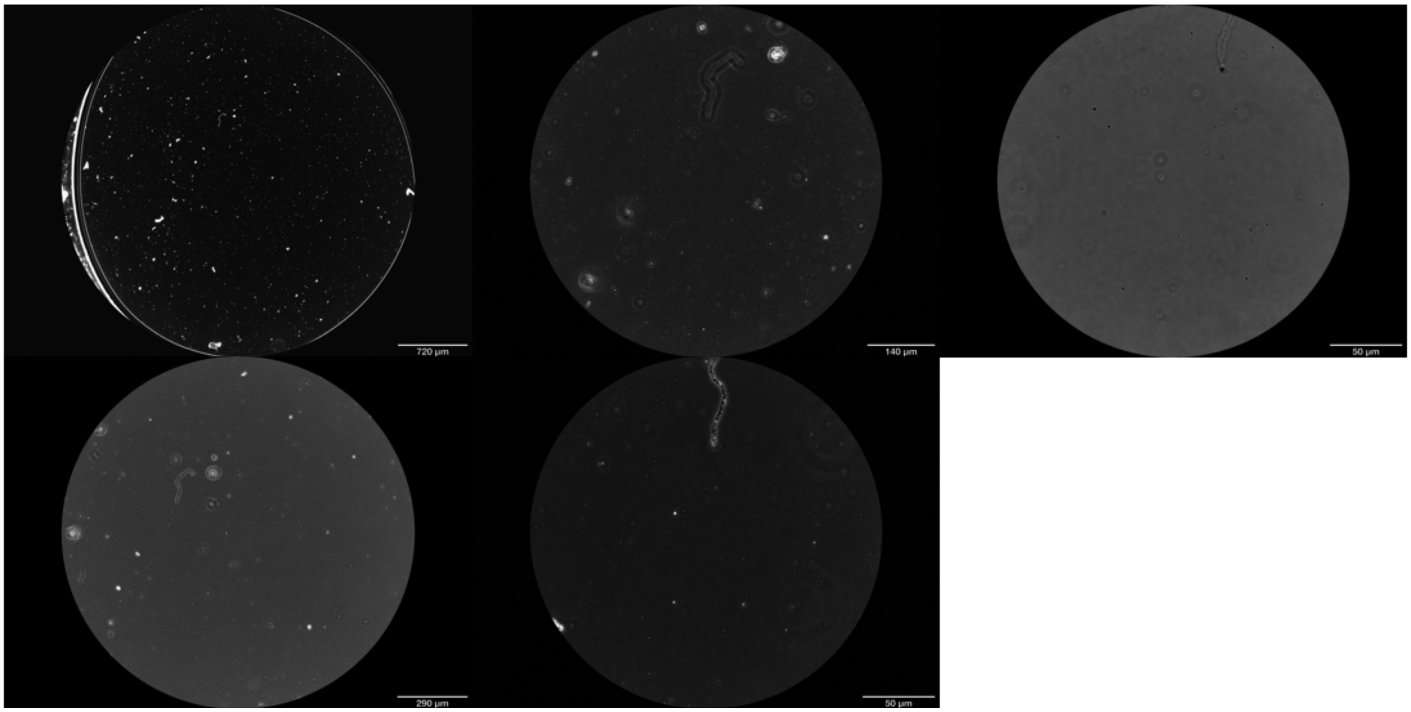

Supplementary Figure S121. Brightfield microscopy imaging of depsipeptides resulting from dry-down reactions between Hydroxypropionic acid and γ-aminopentanoic acid. Microscopy of sample A24-Hydroxypropionic acid + γ-aminopentanoic acid, resuspended in 80% deionized water and 20% acetonitrile (v/v), at five different magnifications- x4, x10, x20, x40 and x60.

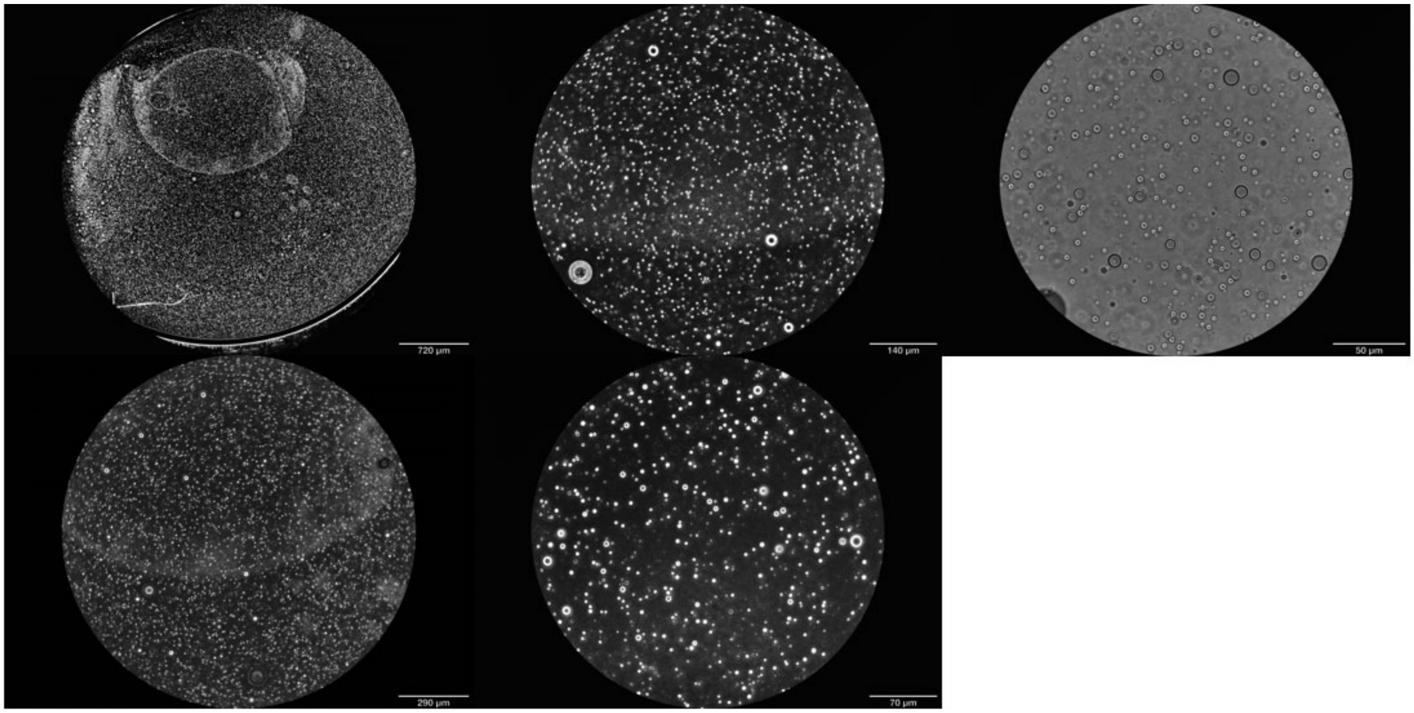

Supplementary Figure S122. Brightfield microscopy imaging of depsipeptides resulting from dry-down reactions between DL-lactic acid and glycine. Microscopy of sample A25- DL-lactic acid + glycine, resuspended in 80% deionized water and 20% acetonitrile (v/v), at five different magnifications- x4, x10, x20, x40 and x60.

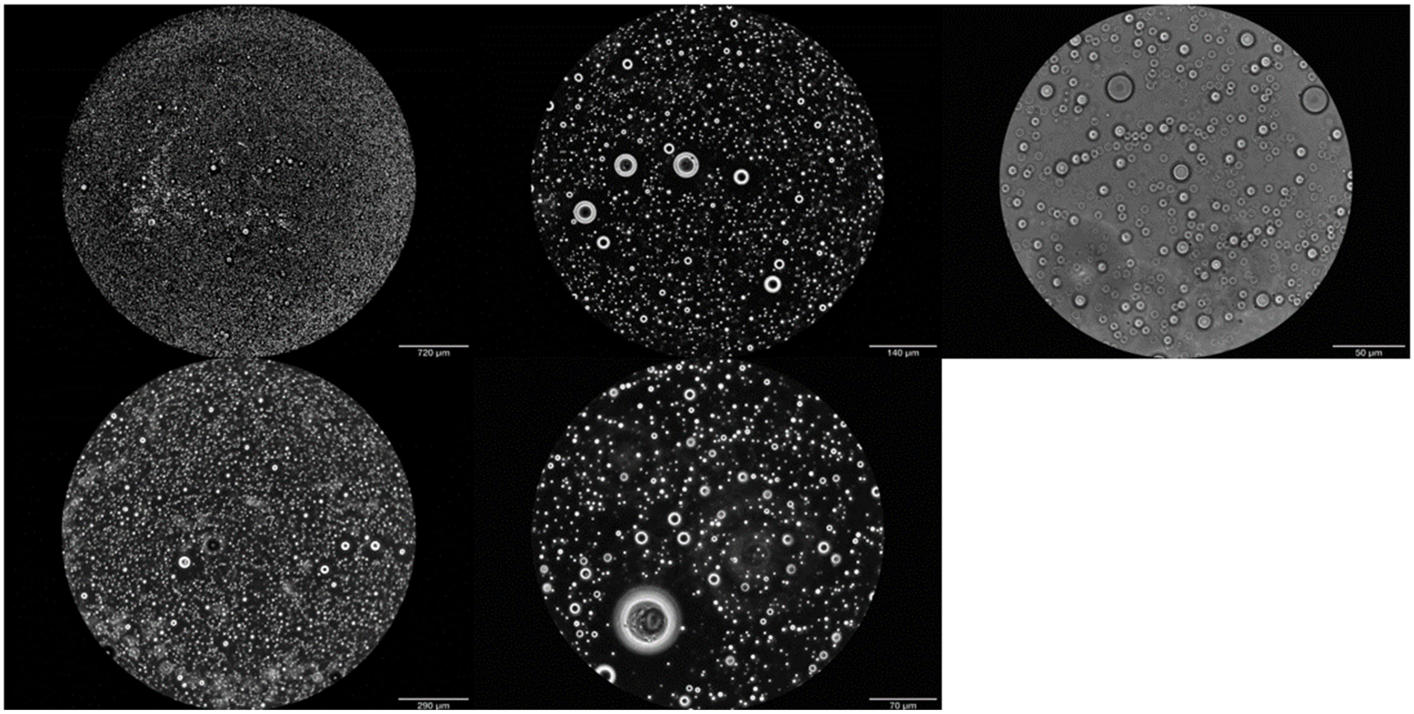

Supplementary Figure S123. Brightfield microscopy imaging of depsipeptides resulting from dry-down reactions between DL-lactic acid and L-alanine. Microscopy of sample A26- DL-lactic acid + L-alanine, resuspended in 80% deionized water and 20% acetonitrile (v/v), at five different magnifications- x4, x10, x20, x40 and x60.

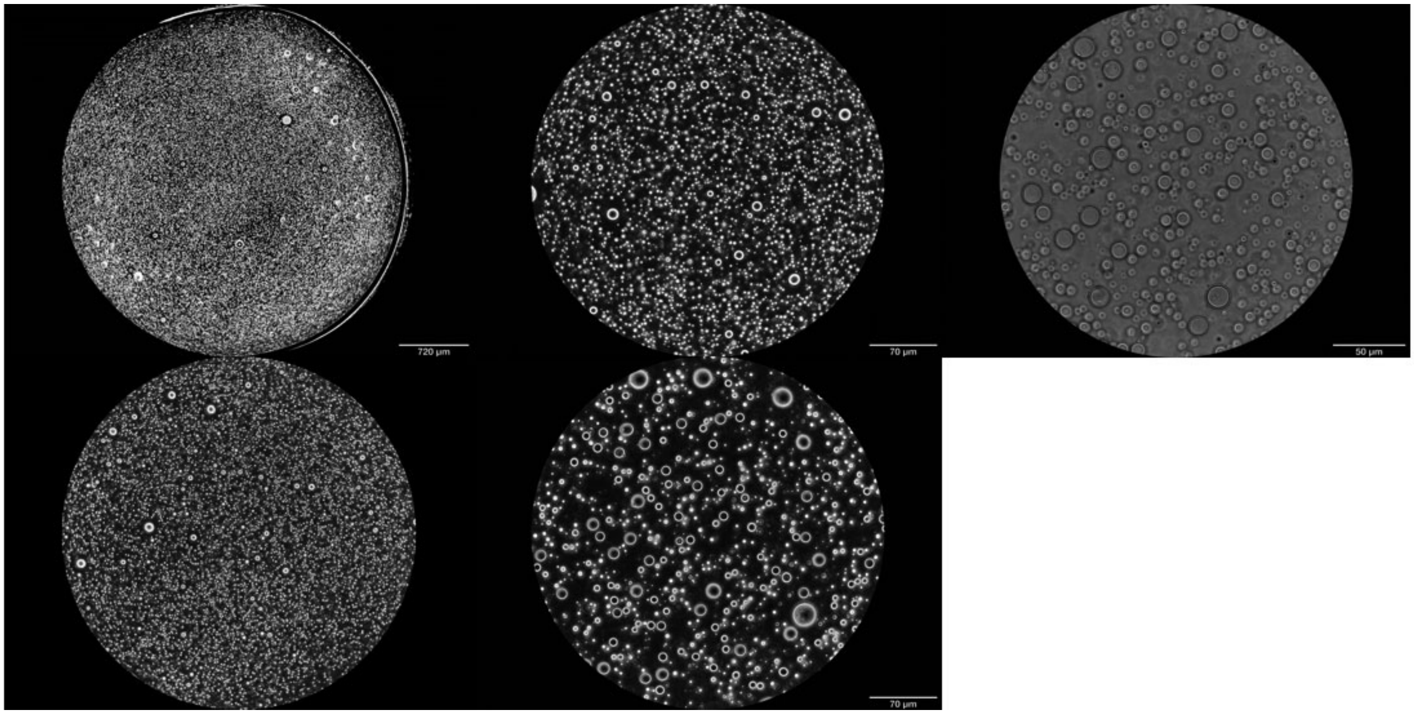

Supplementary Figure S124. Brightfield microscopy imaging of depsipeptides resulting from dry-down reactions between DL-lactic acid and β-alanine. Microscopy of sample A27- DL-lactic acid + β-alanine, resuspended in 80% deionized water and 20% acetonitrile (v/v), at five different magnifications- x4, x10, x20, x40 and x60.

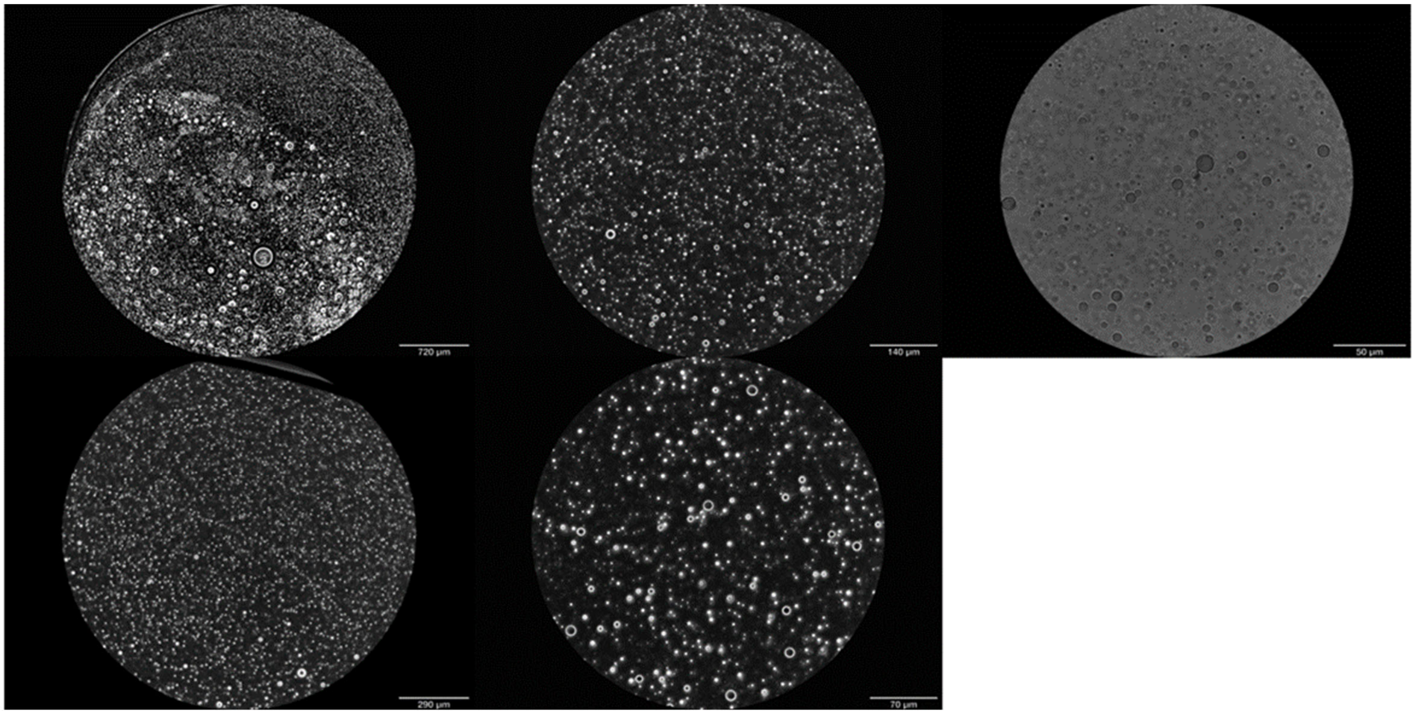

Supplementary Figure S125. Brightfield microscopy imaging of depsipeptides resulting from dry-down reactions between DL-lactic acid and β-aminobutyric acid. Microscopy of sample A28- DL-lactic acid + β-aminobutyric acid, resuspended in 80% deionized water and 20% acetonitrile (v/v), at five different magnifications- x4, x10, x20, x40 and x60.

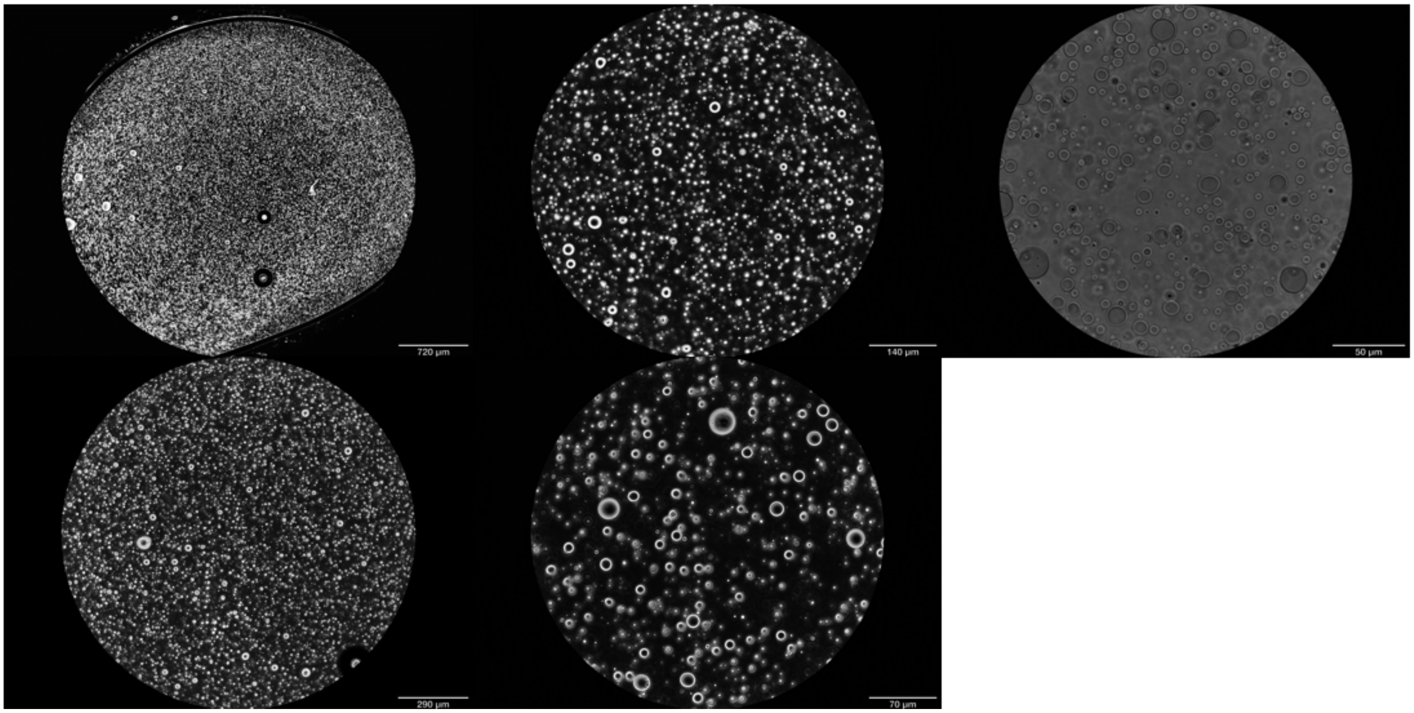

Supplementary Figure S126. Brightfield microscopy imaging of depsipeptides resulting from dry-down reactions between DL-lactic acid and γ-aminobutyric acid. Microscopy of sample A29- DL-lactic acid + γ-aminobutyric acid, resuspended in 80% deionized water and 20% acetonitrile (v/v), at five different magnifications- x4, x10, x20, x40 and x60.

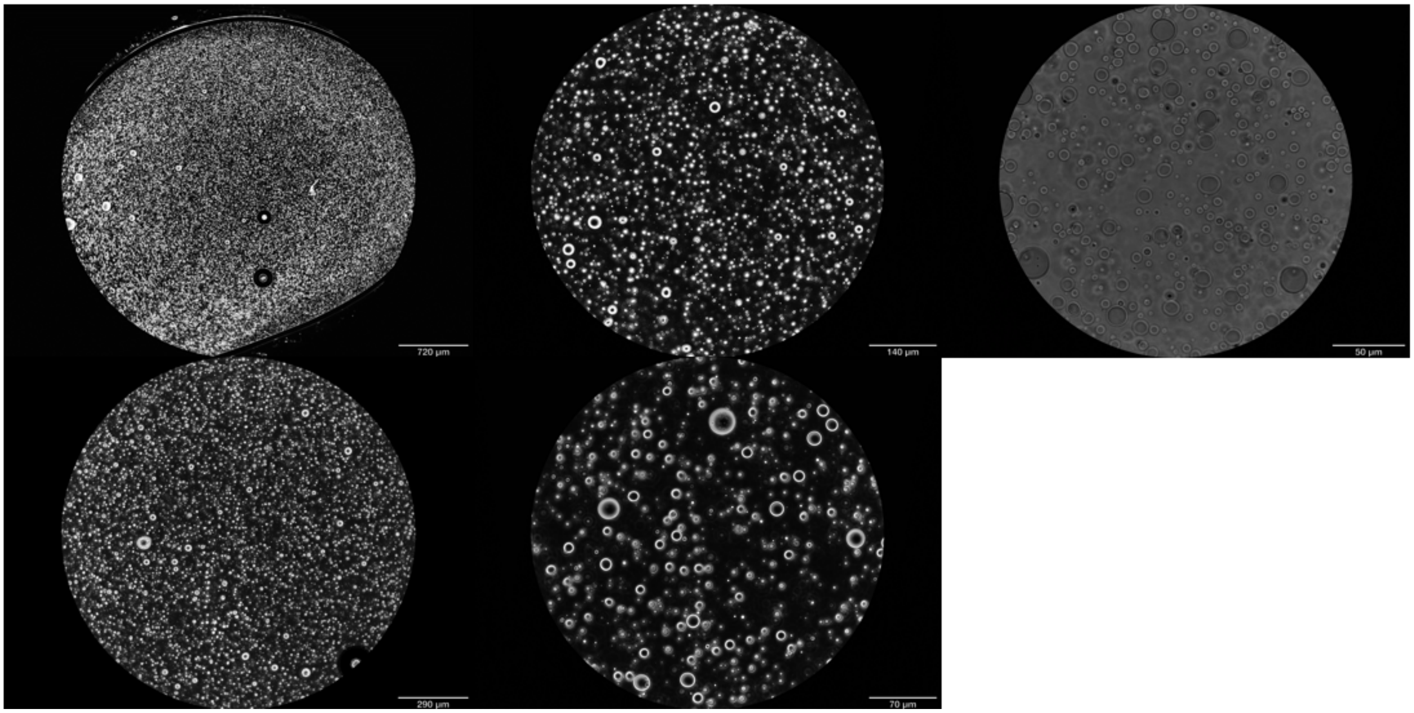

Supplementary Figure S127. Brightfield microscopy imaging of depsipeptides resulting from dry-down reactions between DL-lactic acid and 4-aminopentanoic acid. Microscopy of sample A30- DL-lactic acid + 4-aminopentanoic acid, resuspended in 80% deionized water and 20% acetonitrile (v/v), at five different magnifications- x4, x10, x20, x40 and x60.

glycine

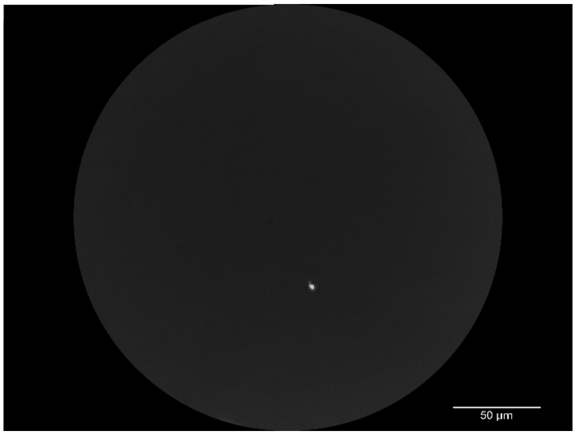

L-alanine

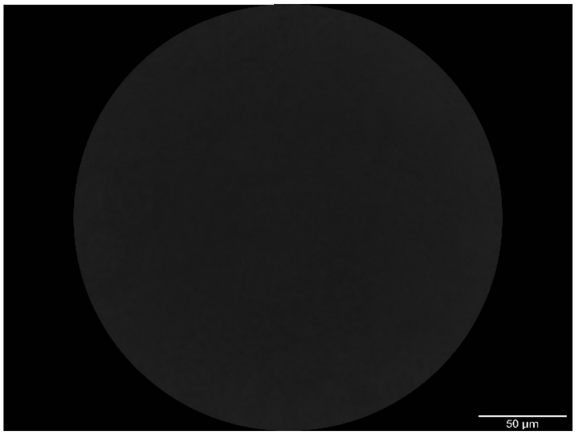

β-alanine

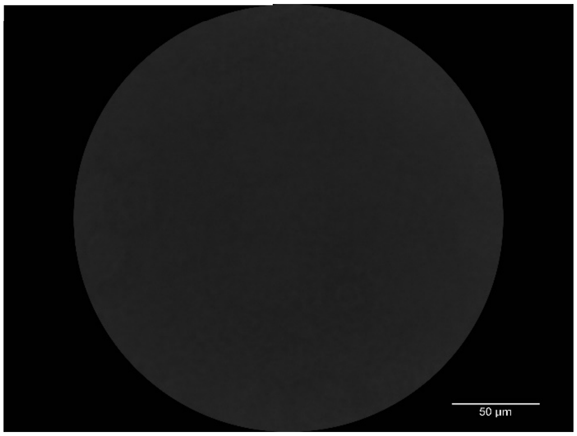

β-aminobutyric acid

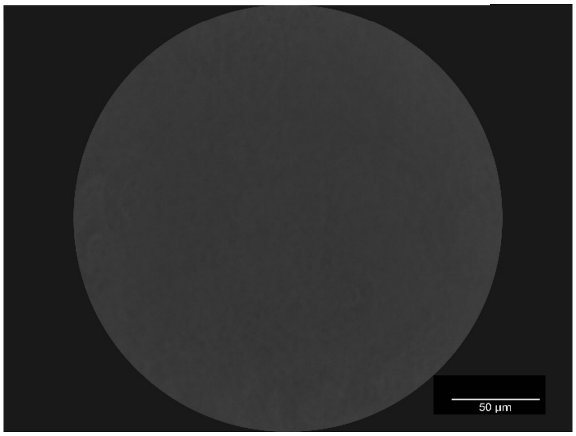

γ-aminobutyric acid

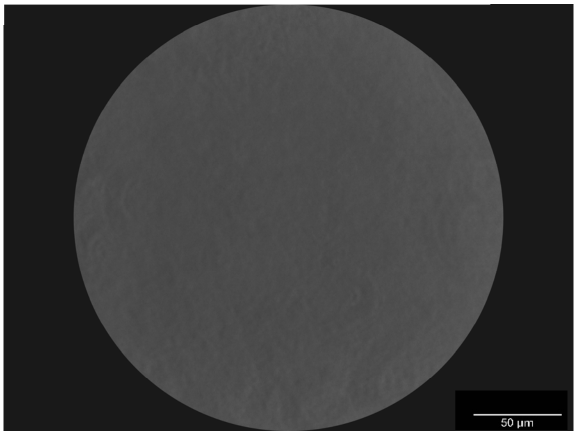

γ-aminopentanoic acid

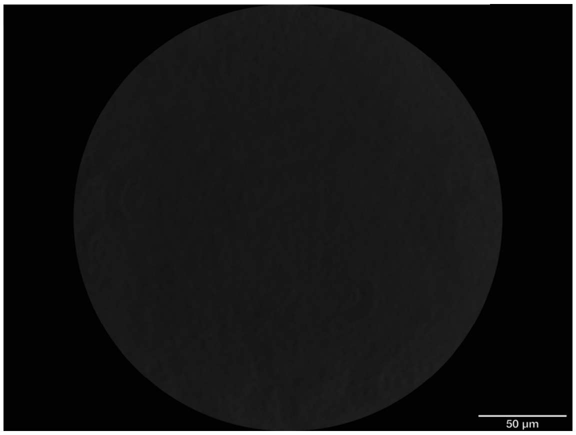

Supplementary Figure S128. Brightfield microscopy imaging of fresh control samples of L-lactic acid and all amino acids. Microscopy of fresh mixtures of L-lactic acid and all amino acid, resuspended in 80% deionized water and 20% acetonitrile (v/v), x60 magnification.

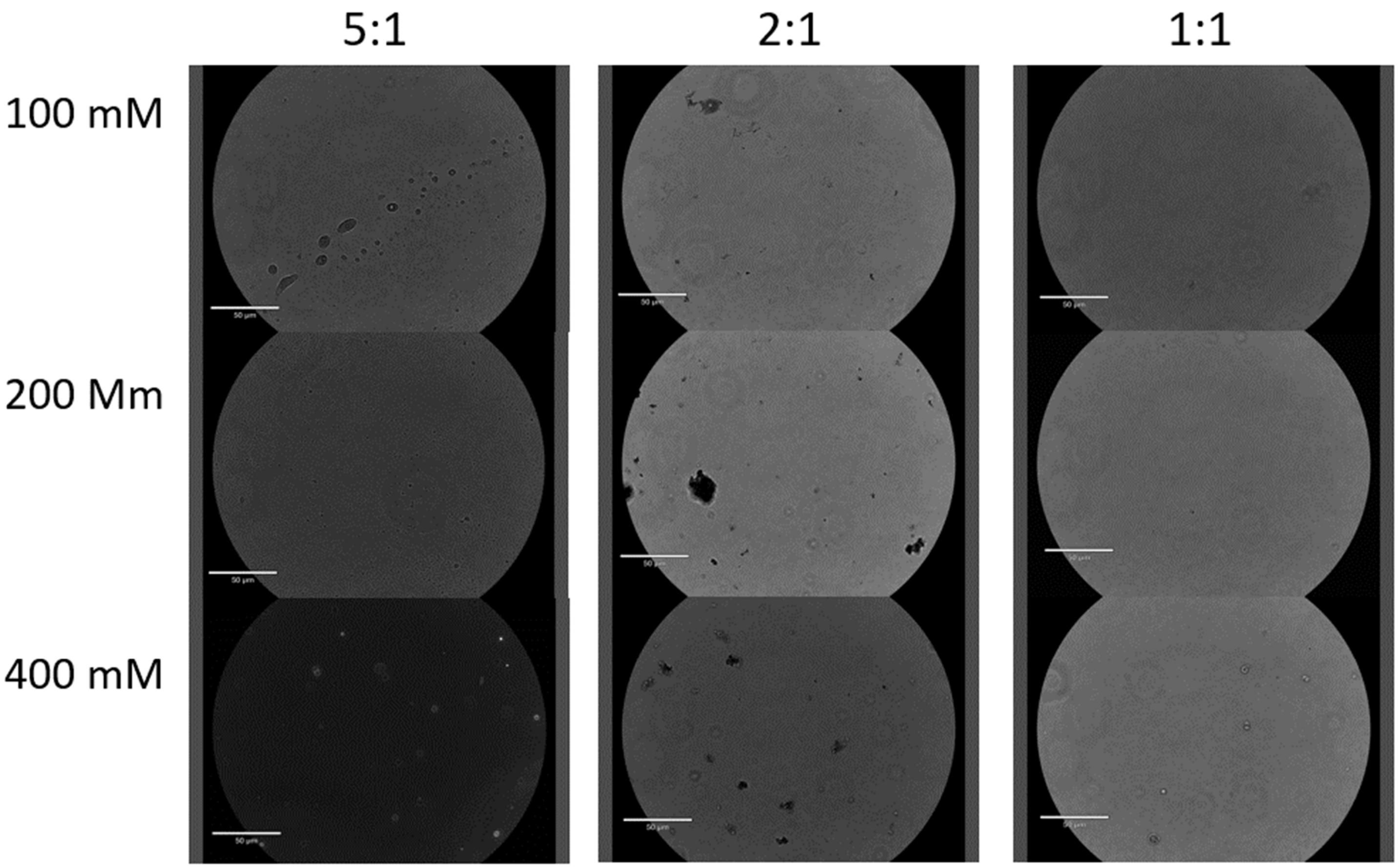

Supplementary Figure S129. Brightfield microscopy imaging of depsipeptides in 100% water at various concentrations. L-Lactic acid (lac) and Glycine were dried down at a 5:1, 2:1, or 1:1 molar ratio (lac:Glycine), for 7 days at 85 °C. Dry-down products were then resuspended in three different concentrations (referring to the initial amount of Glycine monomer) in 100% water. Scale bar is 50 µm.

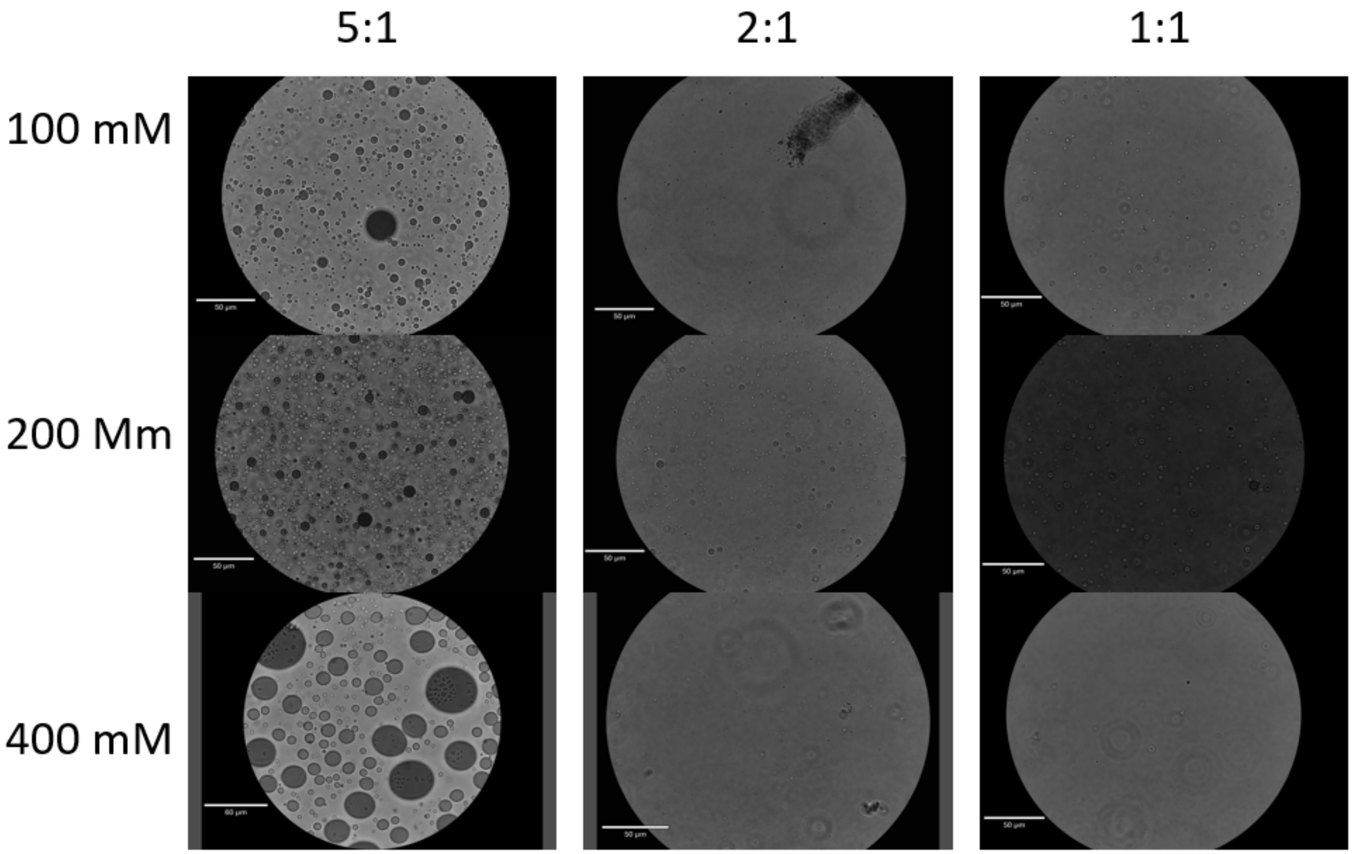

Supplementary Figure S130. Brightfield microscopy imaging of depsipeptides in 80% aqueous 20% organic solution at various concentrations. L-Lactic acid (lac) and Glycine were dried down at a 5:1, 2:1, or 1:1 molar ratio (lac:Glycine), for 7 days at 85 °C. Dry down products were then resuspended in three different concentrations (referring to the initial amount of Glycine monomer) in a mixture of 20% acetonitrile in water. Scale bar is 50 µm.

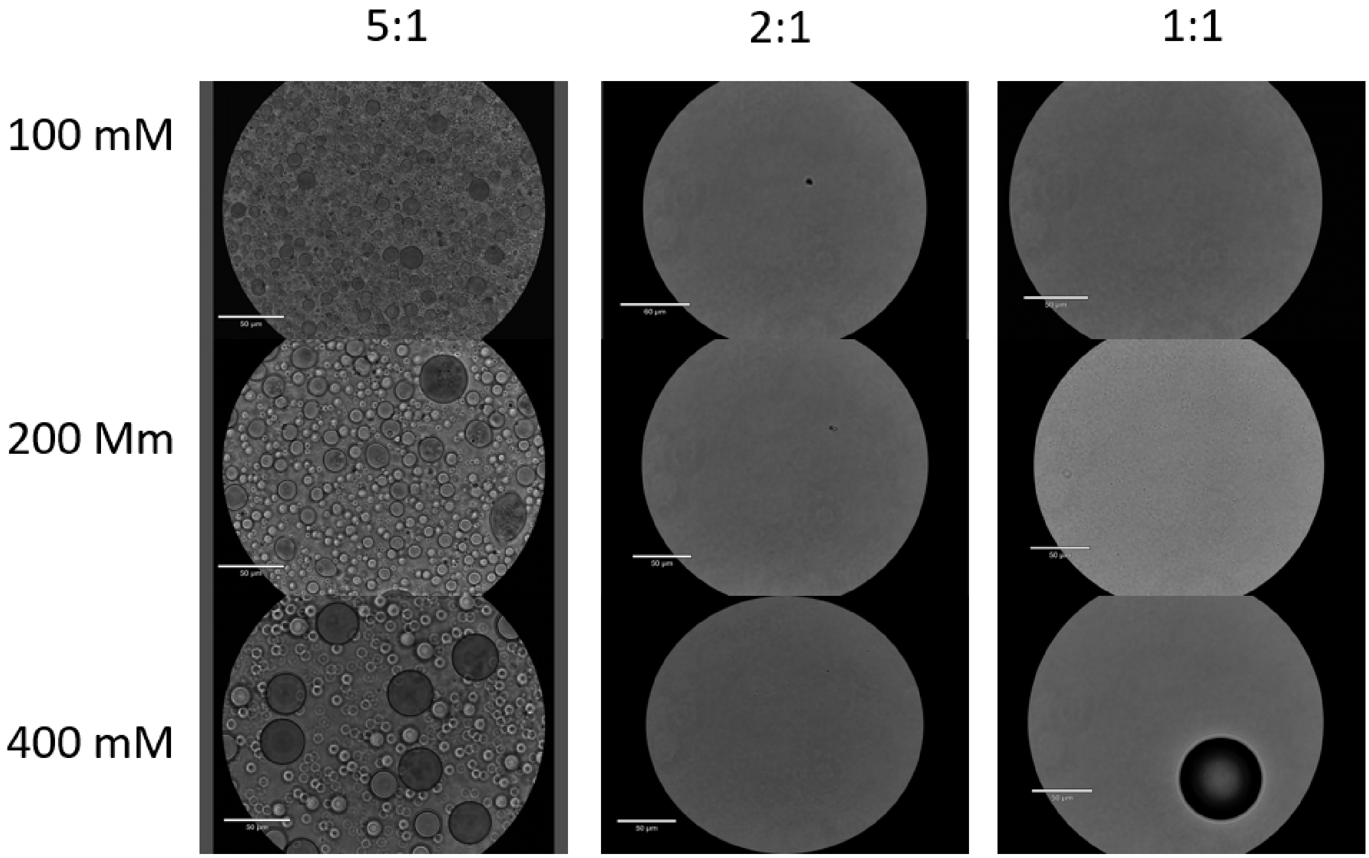

Supplementary Figure S131. Brightfield microscopy imaging of depsipeptides in 60% aqueous 40% organic solution at various concentrations. L-Lactic acid (lac) and Glycine were dried down at a 5:1, 2:1, or 1:1 molar ratio (lac:Glycine), for 7 days at 85 °C. Dry down products were then resuspended in three different concentrations (referring to the initial amount of Glycine monomer) in a mixture of 40% acetonitrile in water. Scale bar is 50 µm.

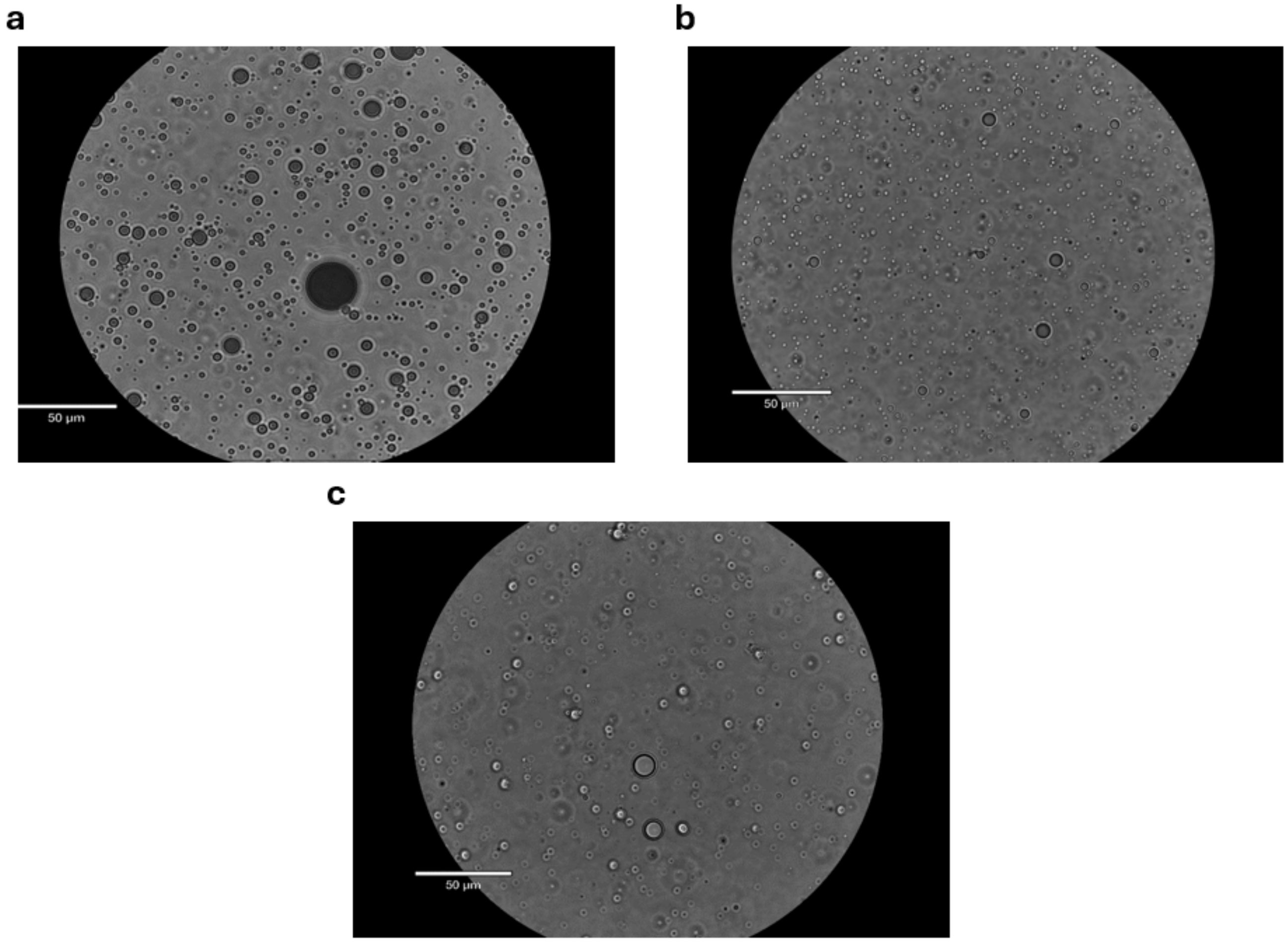

Supplementary Figure S132. Brightfield microscopy imaging of depsipeptides demonstrates stability of microdroplet structures. L-Lactic acid (lac) and Glycine were dried down at a 5:1 molar ratio, in favor of lac, for 7 days at 85 °C. Dry down products were then resuspended in an aqueous solution of 20% acetonitrile in water (v/v). Scale bar in each picture is 50 µm.**a)** microscopy imaging taken immediately upon resuspension of the dry-down product. **b)** the sample after incubation for 7 weeks at RT and freezing at -80°C for 24 hours. **c)** the sample after incubation for 7 weeks at RT.

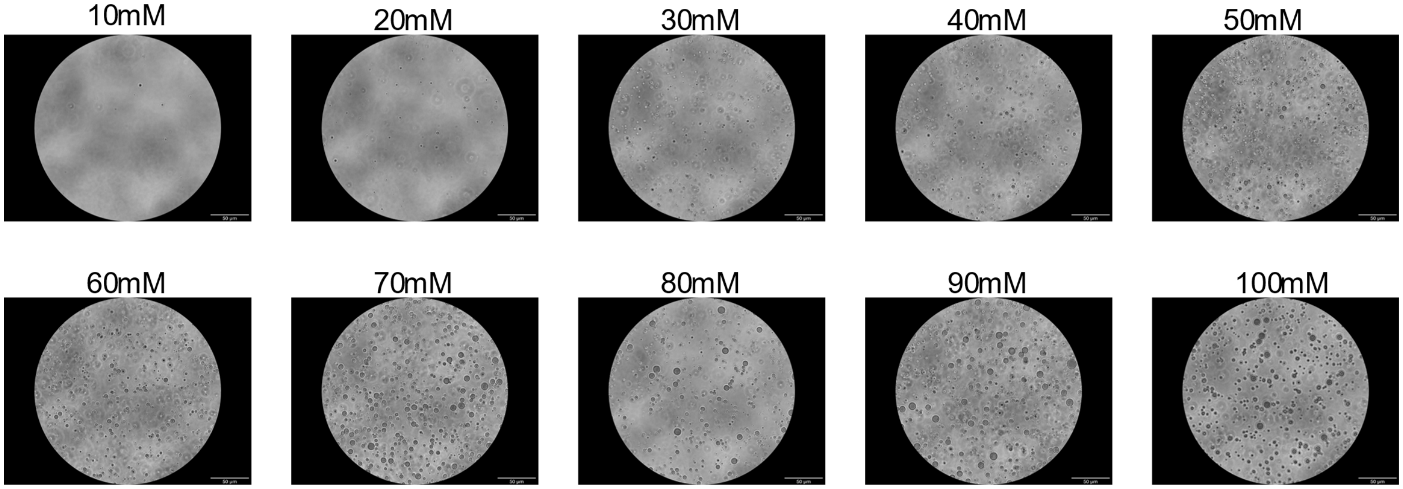

Supplementary Figure S133. Concentration-dependent assembly formation of L-Lactic acid & Glycine. L-Lactic acid (lac) and Glycine were dried down at a 5:1 molar ratio, in favor of lac, for 7 days at 85 °C. Dry down products were then resuspended in an aqueous solution of 20% acetonitrile in water (v/v) to give a concentration of 100 mM amino acid (referring to the starting amino acid concentration). We then tested assembly at different concentrations following dilution, from 10 mM to 100 mM.

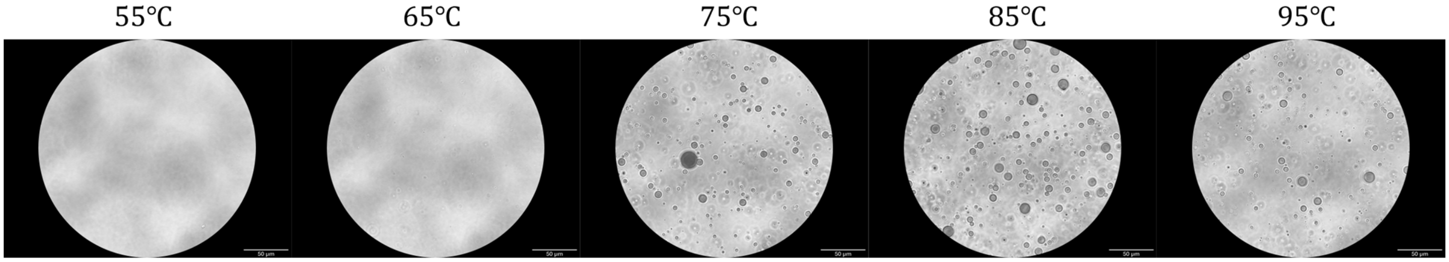

Supplementary Figure S134. Temperature-dependent assembly formation of L-Lactic acid & Glycine. L-Lactic acid (lac) and Glycine were dried down at a 5:1 molar ratio, in favor of lac, for 7 days at five different temperatures: 95 °C, 85 °C, 75 °C, 65 °C, and 55 °C. Dry down products were then resuspended in an aqueous solution of 20% acetonitrile in water (v/v) and observed under the microscope.

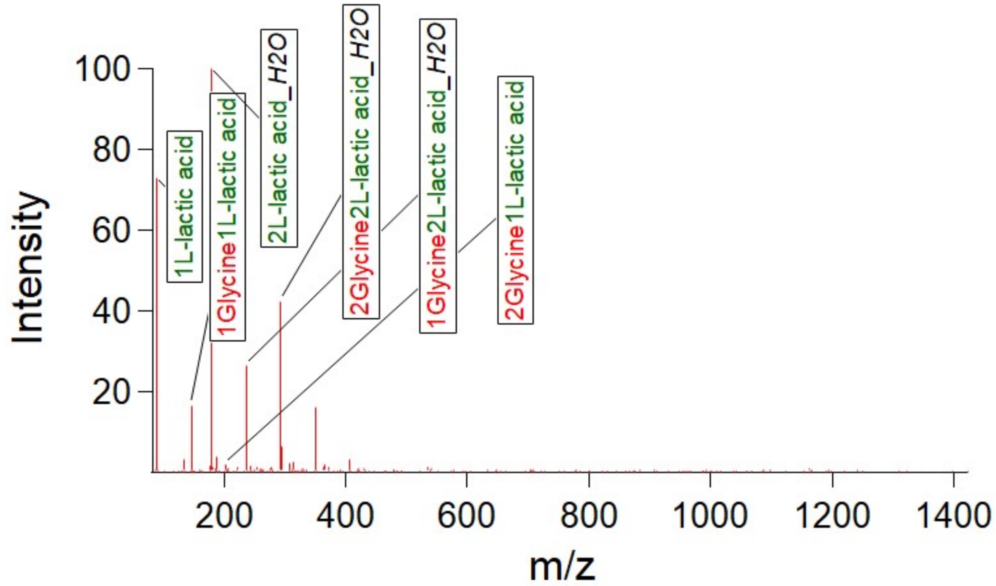

Supplementary Figure S135. Hydrolysis of L-lactic acid and Glycine negative MS spectrum. L-Lactic acid (lac) and Glycine (Gly) were dried down at a 5:1 molar ratio (lac:Glycine), for 7 days at 85 °C. We rehydrated the dry sample of lac and Gly in 500 ul of water and incubated it for 1 week at 85 ºC with a closed cap to prevent evaporation of water. The resulting products were analyzed by negative-mode ESI-MS, indicating hydrolysis of all ester bonds.

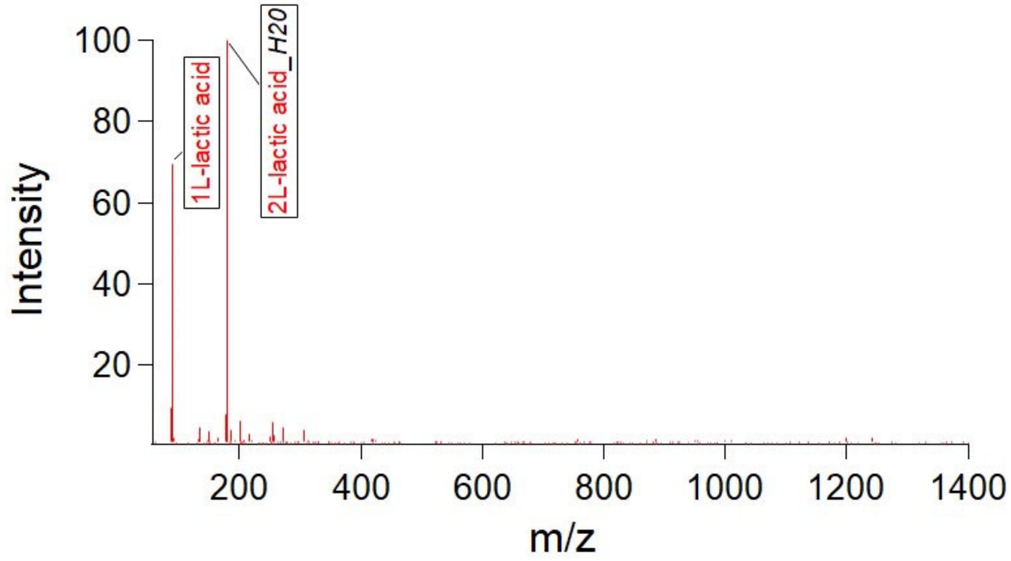

Supplementary Figure S136. Hydrolysis of L-lactic acid negative MS spectrum. L-Lactic acid (lac) was dried down for 7 days at 85 °C.We rehydrated the dry sample in 500 ul of water and incubated it for 1 week at 85 ºC with a closed cap to prevent evaporation of water. The resulting products were analyzed by negative-mode ESI-MS, indicating hydrolysis of all ester bonds.

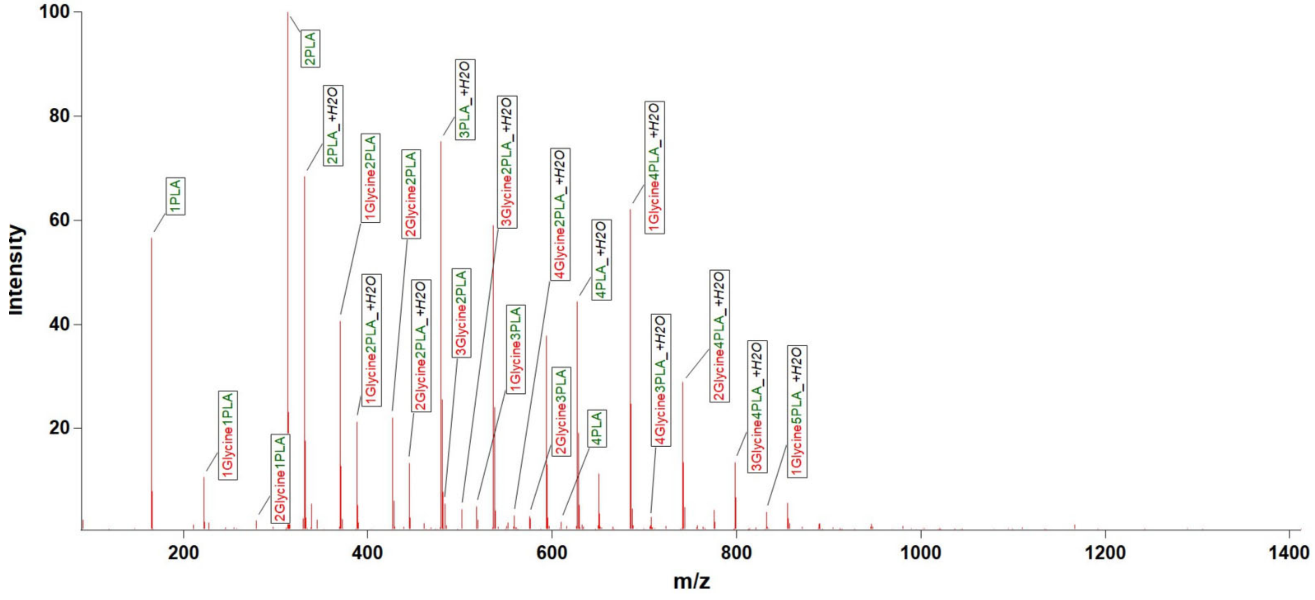

Supplementary Figure S137. ESI-MS spectrum of a dry-down reaction of sample NA1 – Phenyllactic acid and glycine. Phenyllactic acid (pla) and Glycine (gly) were dried down at a 5:1 molar ratio, in favor of PLA, for 7 days at 85 °C. Dry down products were then resuspended in an aqueous solution of 20% acetonitrile in water (v/v). The resulting products were analyzed by negative-mode ESI-MS, indicating a variety of depsipeptides. PLA is labeled in green, gly is labeled in red.

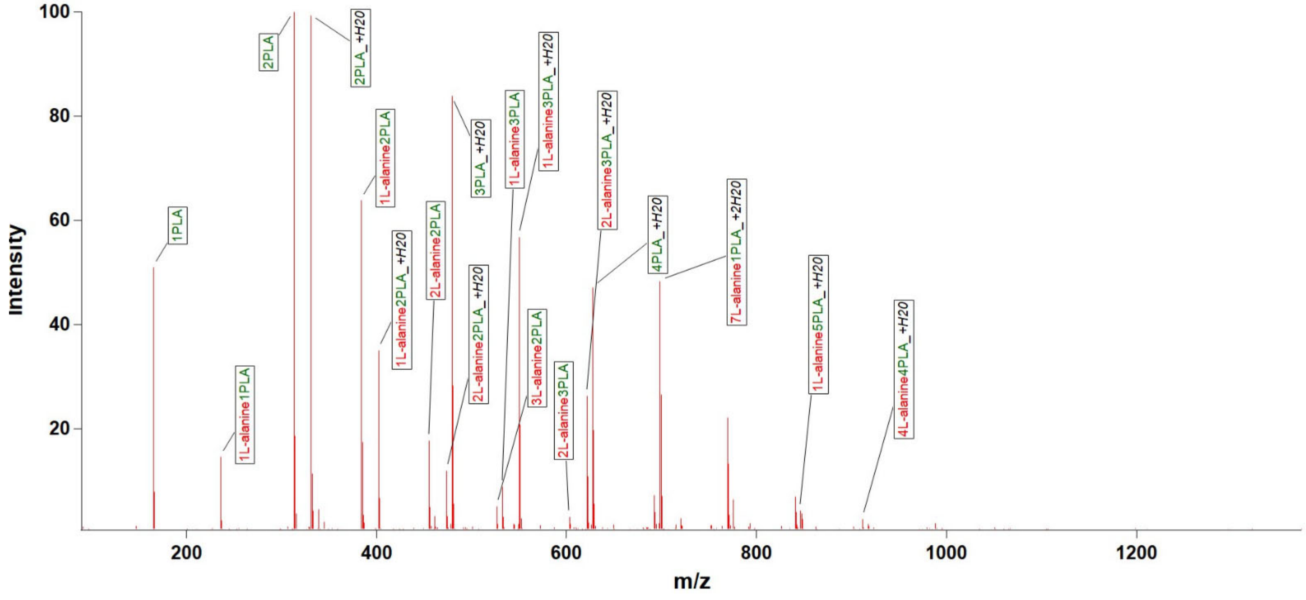

Supplementary Figure S138. ESI-MS spectrum of a dry-down reaction of sample NA2 – Phenyllactic acid and L-alanine. Phenyllactic acid (PLA) and L-alanine (ala) were dried down at a 5:1 molar ratio, in favor of PLA, for 7 days at 85 °C. Dry down products were then resuspended in an aqueous solution of 20% acetonitrile in water (v/v). The resulting products were analyzed by negative-mode ESI-MS, indicating a variety of depsipeptides. PLA is labeled in green, ala is labeled in red.

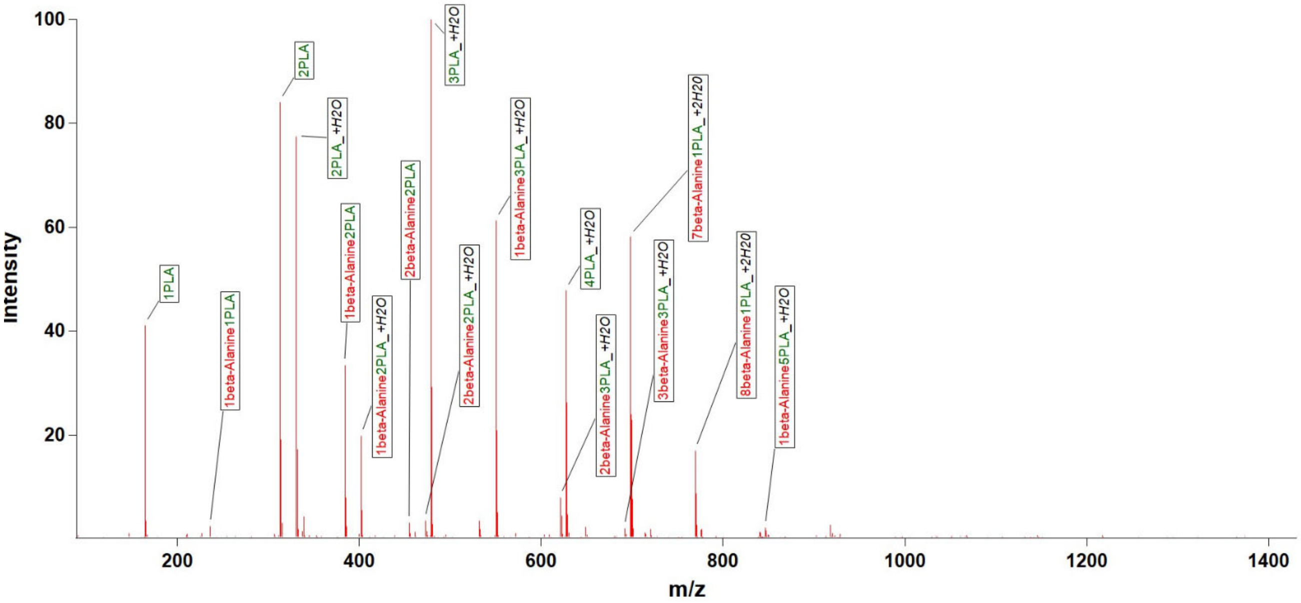

Supplementary Figure S139. ESI-MS spectrum of a dry-down reaction of sample NA3 – Phenyllactic acid and β-alanine. Phenyllactic acid (PLA) and β-alanine (β-ala) were dried down at a 5:1 molar ratio, in favor of PLA, for 7 days at 85 °C. Dry down products were then resuspended in an aqueous solution of 20% acetonitrile in water (v/v). The resulting products were analyzed by negative-mode ESI-MS, indicating a variety of depsipeptides. PLA is labeled in green, β-ala is labeled in red.

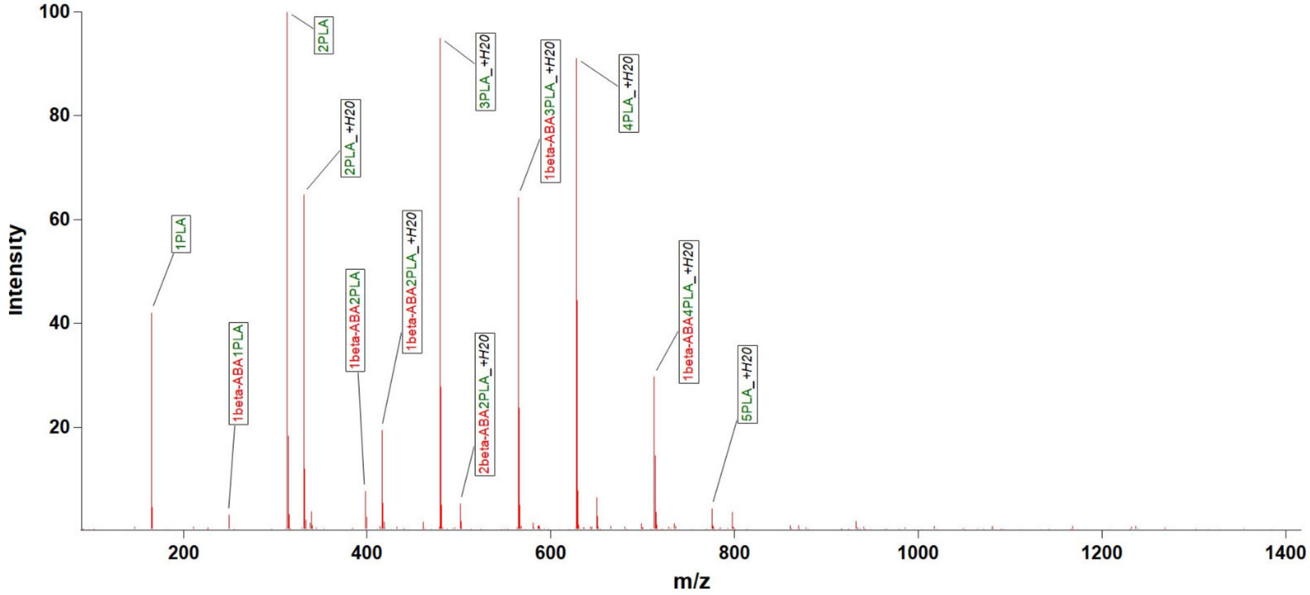

Supplementary Figure S140. ESI-MS spectrum of a dry-down reaction of sample NA4 – Phenyllactic acid and β-aminobutyric acid. Phenyllactic acid (PLA) and β-aminobutyric acid (β-ABA) were dried down at a 5:1 molar ratio, in favor of PLA, for 7 days at 85 °C. Dry down products were then resuspended in an aqueous solution of 20% acetonitrile in water (v/v). The resulting products were analyzed by negative-mode ESI-MS, indicating a variety of depsipeptides. PLA is labeled in green, β-ABA is labeled in red.

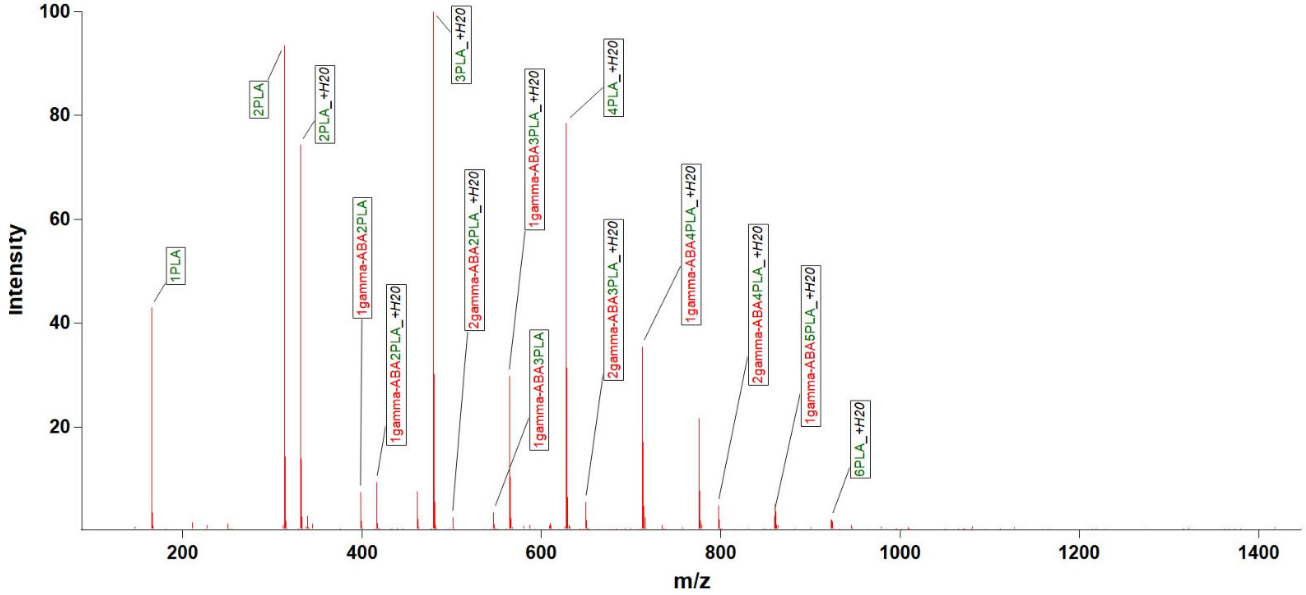

Supplementary Figure S141. ESI-MS spectrum of a dry-down reaction of sample NA5 – Phenyllactic acid and γ-aminobutyric acid. Phenyllactic acid (PLA) and γ-aminobutyric acid (γ-ABA) were dried down at a 5:1 molar ratio, in favor of PLA, for 7 days at 85 °C. Dry down products were then resuspended in an aqueous solution of 20% acetonitrile in water (v/v). The resulting products were analyzed by negative-mode ESI-MS, indicating a variety of depsipeptides. PLA is labeled in green, γ-ABA is labeled in red.

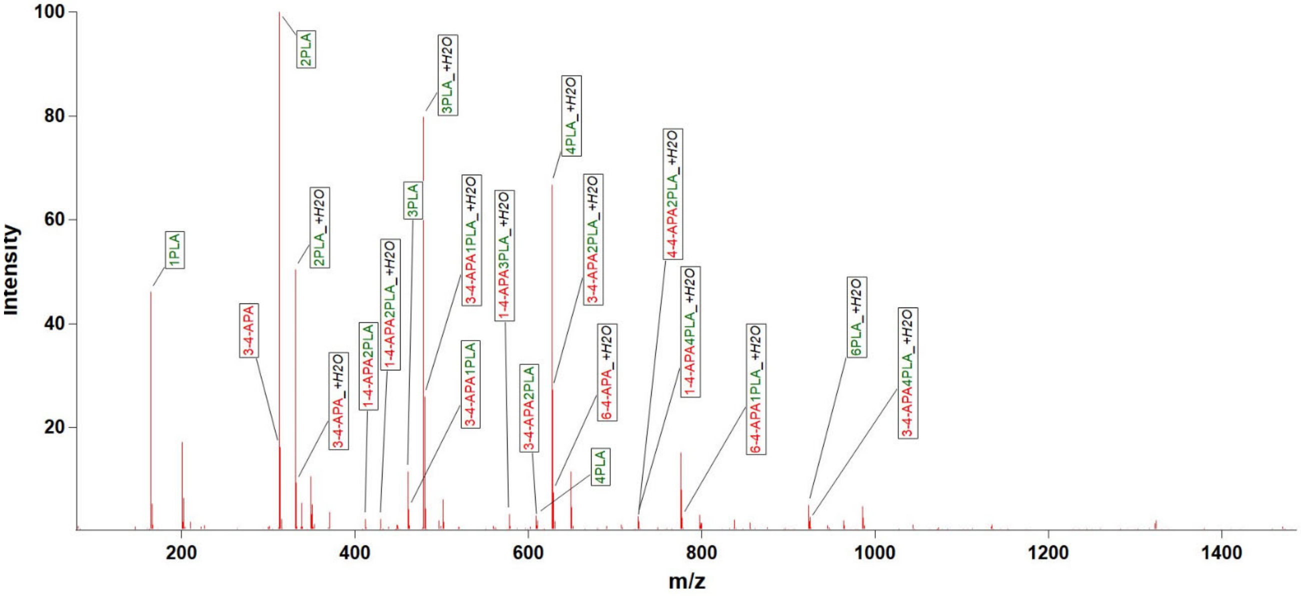

Supplementary Figure S142. ESI-MS spectrum of a dry-down reaction of sample NA6 – Phenyllactic acid and γ-aminopentanoic acid. Phenyllactic acid (PLA) and 4-aminopentanoic acid (γ-APA) were dried down at a 5:1 molar ratio, in favor of PLA, for 7 days at 85 °C. Dry down products were then resuspended in an aqueous solution of 20% acetonitrile in water (v/v). The resulting products were analyzed by negative-mode ESI-MS, indicating a variety of depsipeptides. PLA is labeled in green, γ-APA is labeled in red.

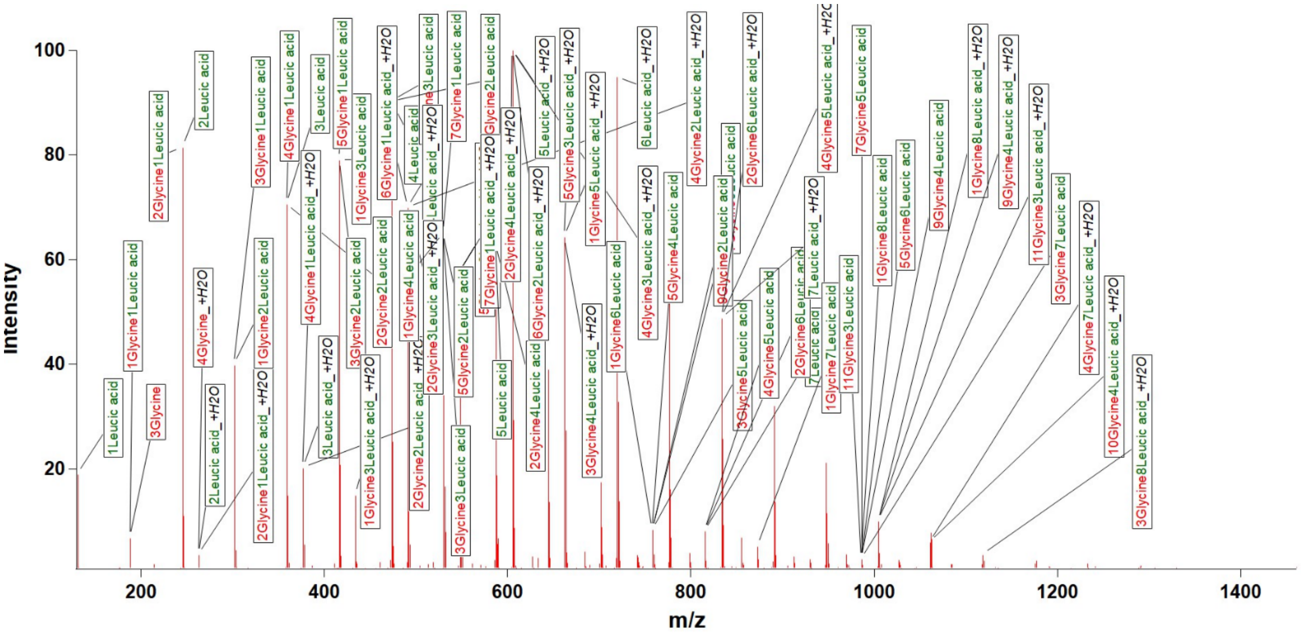

Supplementary Figure S143. ESI-MS spectrum of a dry-down reaction of sample NA7 – Leucic acid and Glycine. Leucic acid (LA) and Glycine (gly) were dried down at a 5:1 molar ratio, in favor of LA, for 7 days at 85 °C. Dry down products were then resuspended in an aqueous solution of 20% acetonitrile in water (v/v). The resulting products were analyzed by negative-mode ESI-MS, indicating a variety of depsipeptides. LA is labeled in green, gly is labeled in red. leucic acid is the same mass as a dimer of glycine so some masses might not reflect the real amount of glycines/leucic acid present in the small depsipeptide.

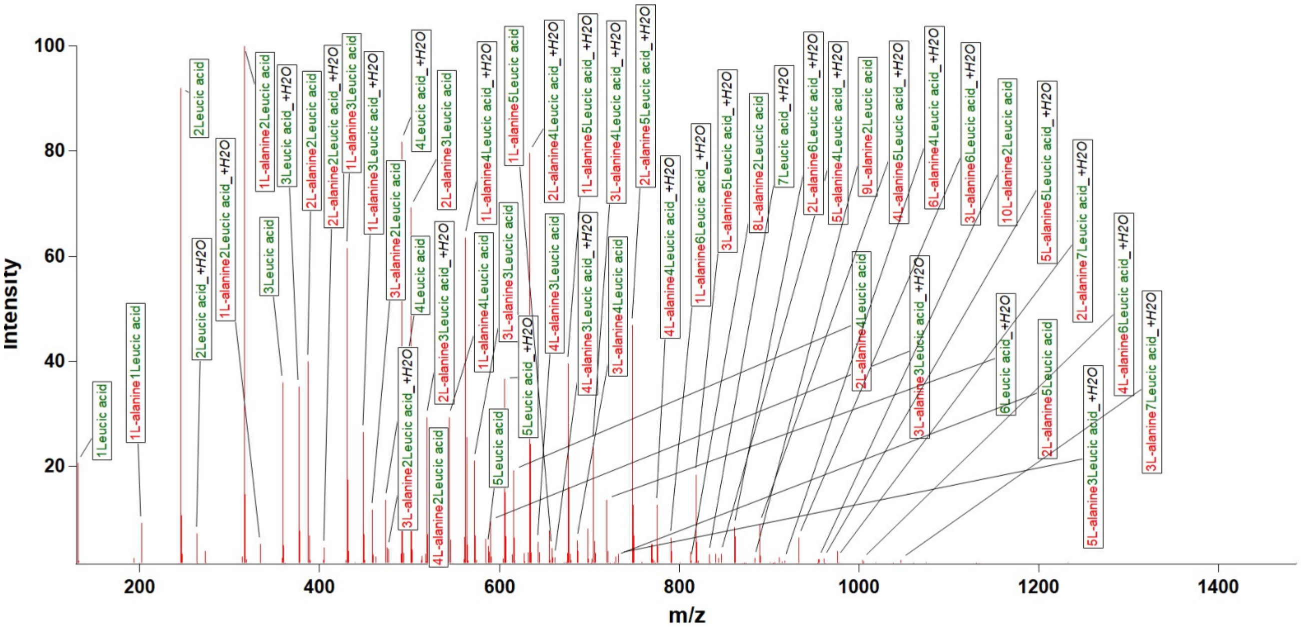

Supplementary Figure S144. ESI-MS spectrum of a dry-down reaction of sample NA8 – Leucic acid and L-alanine. Leucic acid (LA) and L-alanine (ala) were dried down at a 5:1 molar ratio, in favor of LA, for 7 days at 85 °C. Dry down products were then resuspended in an aqueous solution of 20% acetonitrile in water (v/v). The resulting products were analyzed by negative-mode ESI-MS, indicating a variety of depsipeptides. LA is labeled in green, ala is labeled in red.

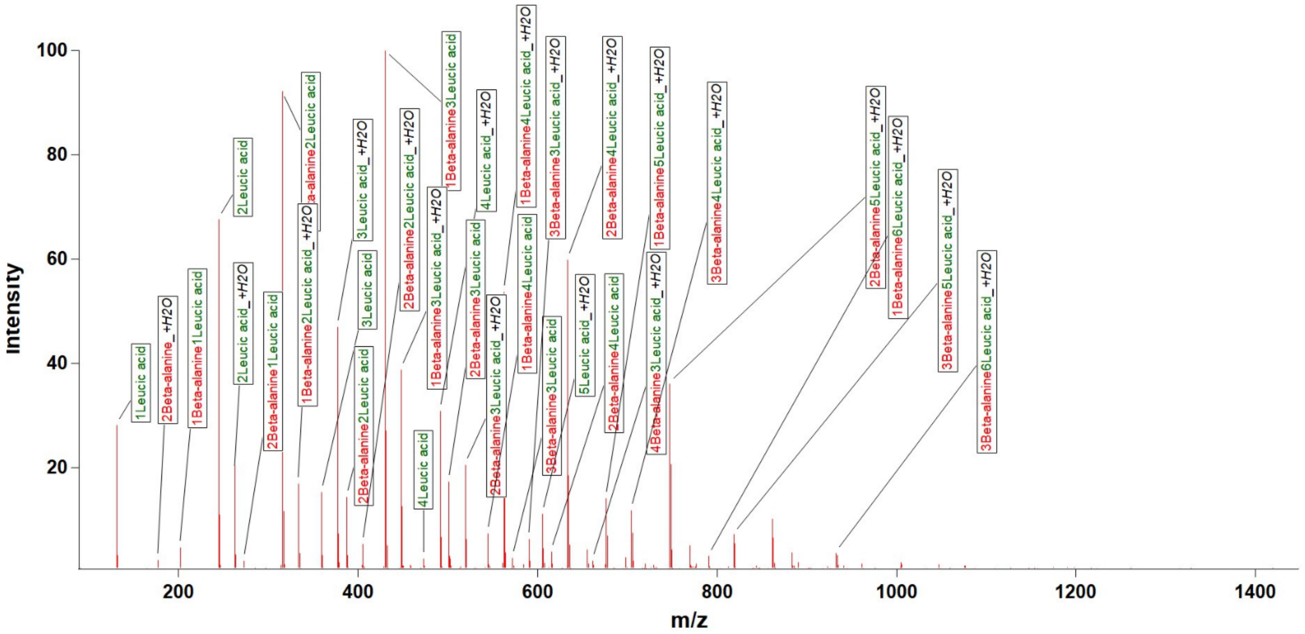

Supplementary Figure S145. ESI-MS spectrum of a dry-down reaction of sample NA9 – Leucic acid and β-alanine. Leucic acid (LA) and β-alanine (β-ala) were dried down at a 5:1 molar ratio, in favor of LA, for 7 days at 85 °C. Dry down products were then resuspended in an aqueous solution of 20% acetonitrile in water (v/v). The resulting products were analyzed by negative-mode ESI-MS, indicating a variety of depsipeptides. LA is labeled in green, β-ala is labeled in red.

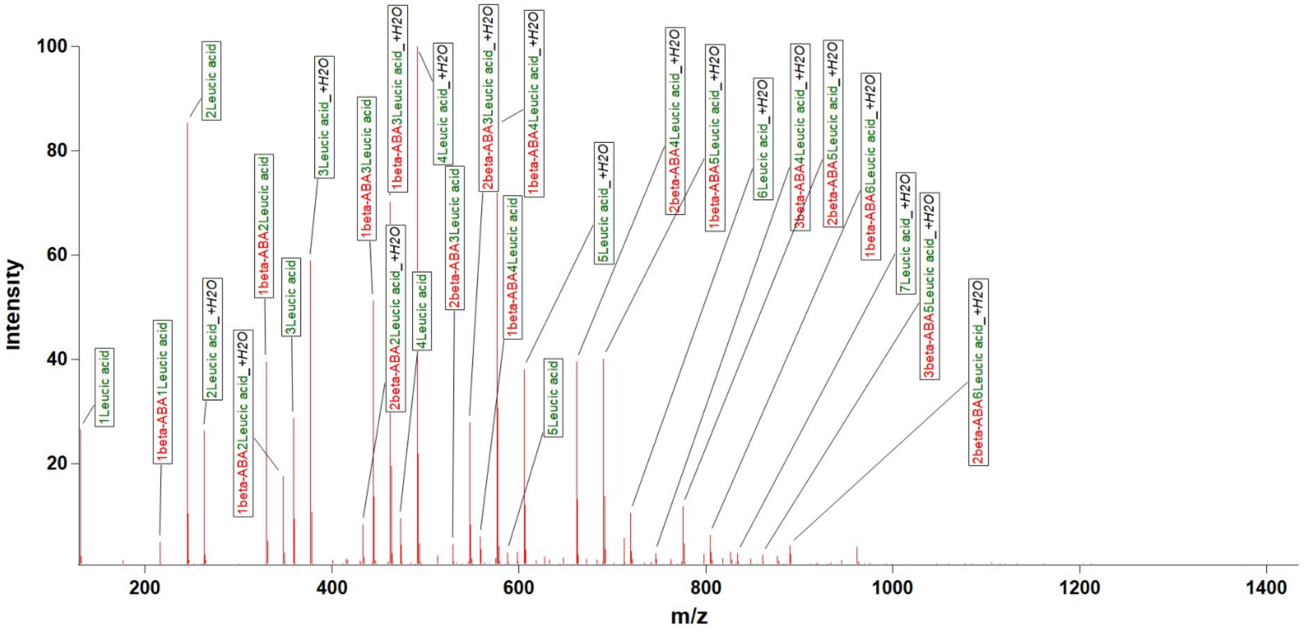

Supplementary Figure S146. ESI-MS spectrum of a dry-down reaction of sample NA10 – Leucic acid and β-aminobutyric acid. Leucic acid (LA) and β-aminobutyric acid (β-ABA) were dried down at a 5:1 molar ratio, in favor of LA, for 7 days at 85 °C. Dry down products were then resuspended in an aqueous solution of 20% acetonitrile in water (v/v). The resulting products were analyzed by negative-mode ESI-MS, indicating a variety of depsipeptides. LA is labeled in green, β-ABA is labeled in red.

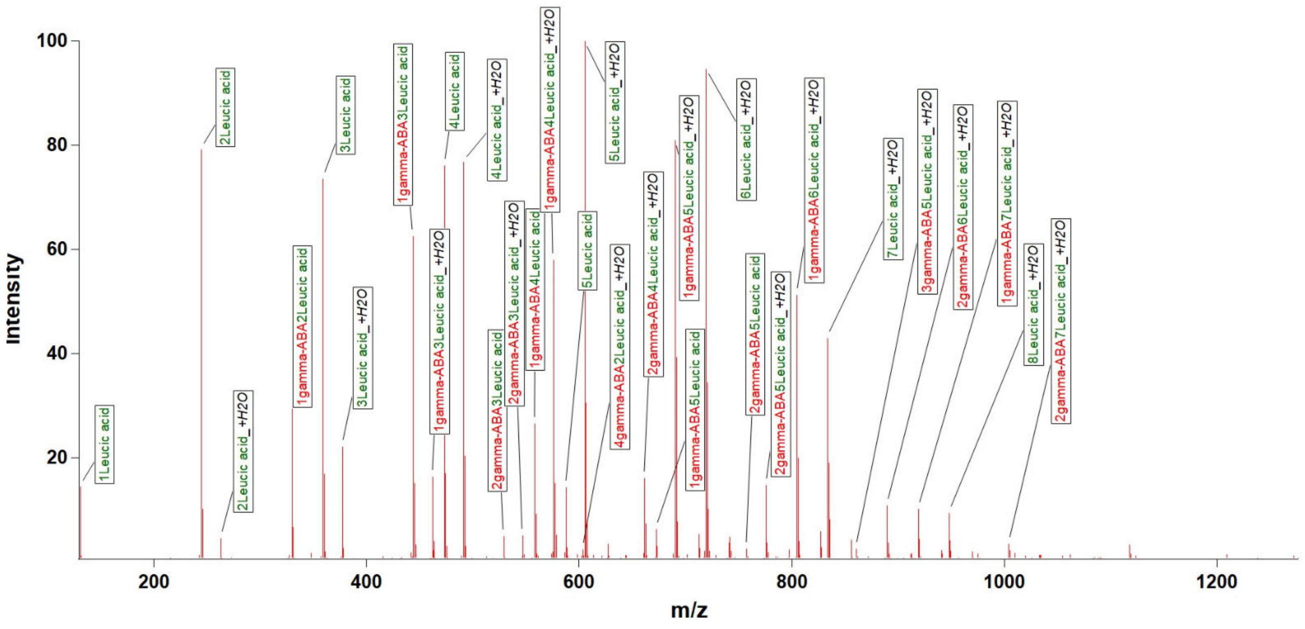

Supplementary Figure S147. ESI-MS spectrum of a dry-down reaction of sample NA11 – Leucic acid and γ-aminobutyric acid. Leucic acid (LA) and γ-aminobutyric acid (γ-ABA) were dried down at a 5:1 molar ratio, in favor of LA, for 7 days at 85 °C. Dry down products were then resuspended in an aqueous solution of 20% acetonitrile in water (v/v). The resulting products were analyzed by negative-mode ESI-MS, indicating a variety of depsipeptides. LA is labeled in green, γ-ABA is labeled in red.

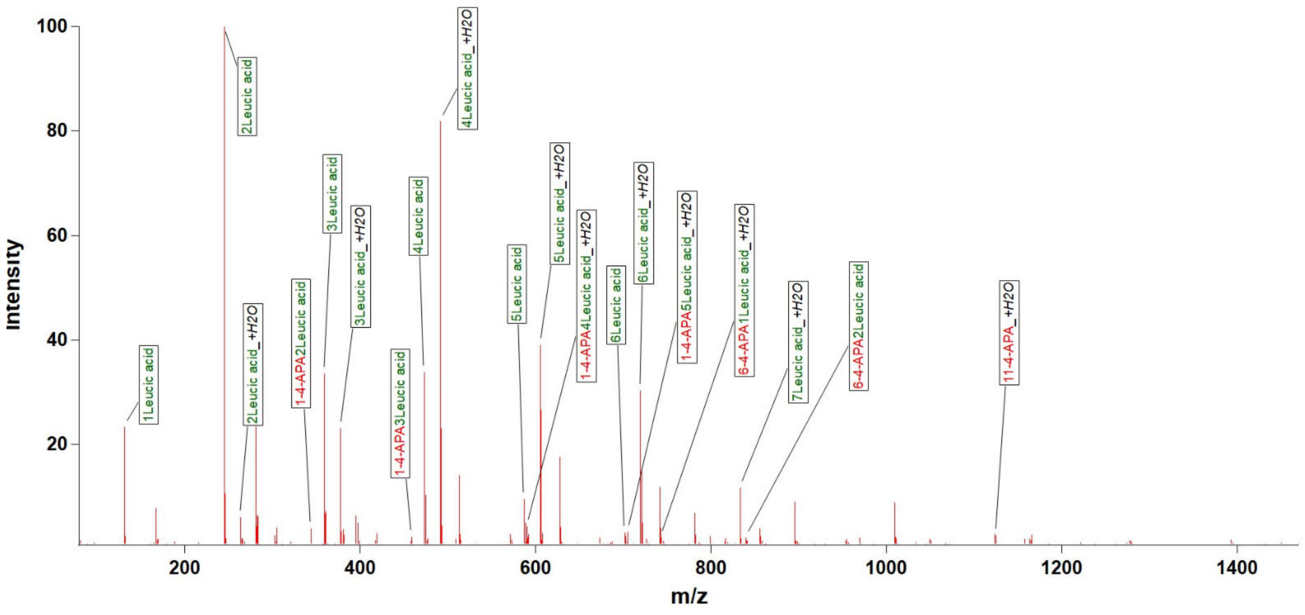

Supplementary Figure S148. ESI-MS spectrum of a dry-down reaction of sample NA12 – Leucic acid and γ-aminopentanoic acid. Leucic acid (LA) and 4-aminopentanoic acid (γ-APA) were dried down at a 5:1 molar ratio, in favor of LA, for 7 days at 85 °C. Dry down products were then resuspended in an aqueous solution of 20% acetonitrile in water (v/v). The resulting products were analyzed by negative-mode ESI-MS, indicating a variety of depsipeptides. LA is labeled in green, γ-APA is labeled in red.

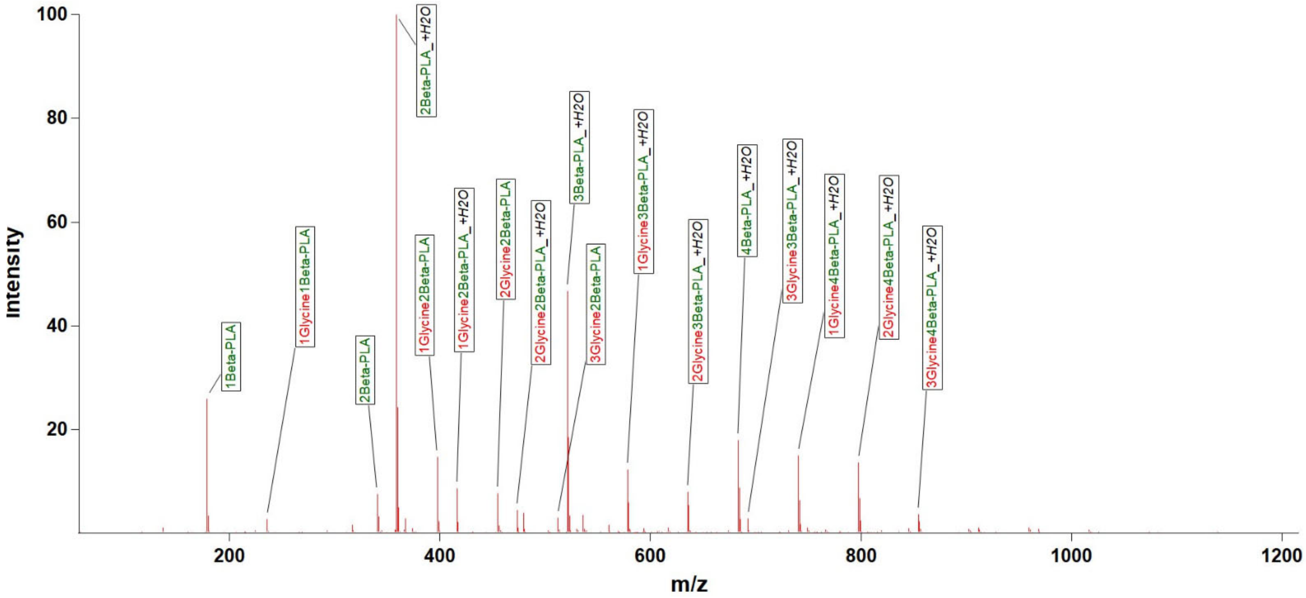

Supplementary Figure S149. ESI-MS spectrum of a dry-down reaction of sample NA13 – β-Phenyllactic acid and Glycine. β-Phenyllactic acid (β-PLA) and Glycine (gly) were dried down at a 5:1 molar ratio, in favor of β-PLA, for 7 days at 85 °C. Dry down products were then resuspended in an aqueous solution of 20% acetonitrile in water (v/v). The resulting products were analyzed by negative-mode ESI-MS, indicating a variety of depsipeptides. β-PLA is labeled in green, gly is labeled in red.

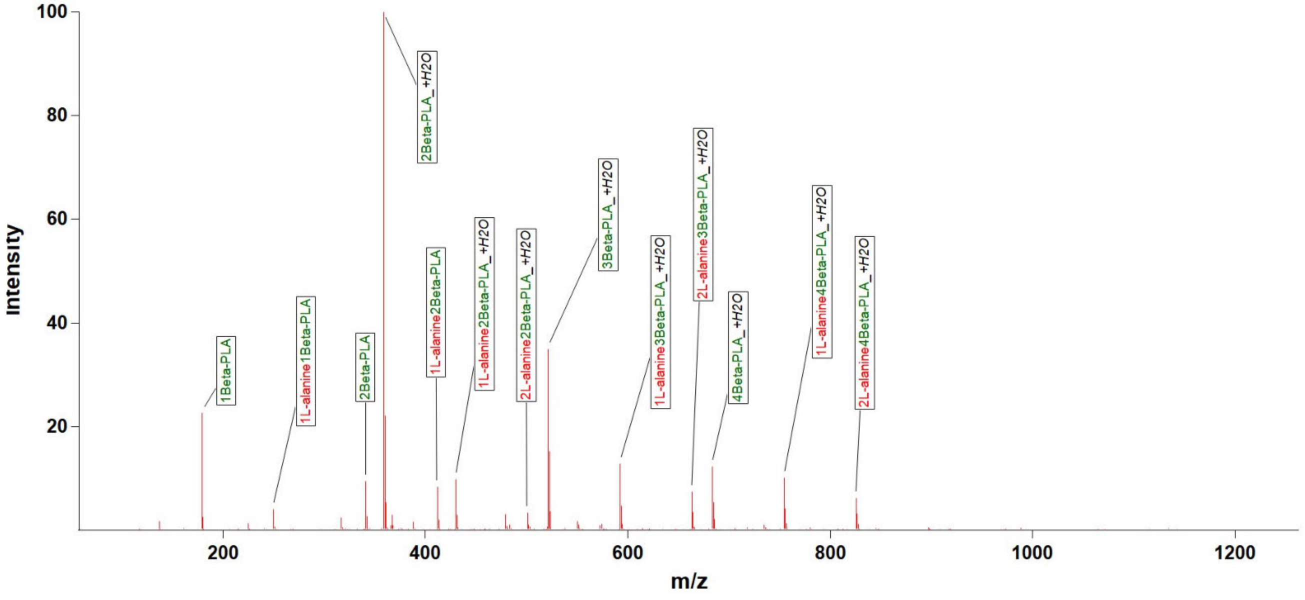

Supplementary Figure S150. ESI-MS spectrum of a dry-down reaction of sample NA14 – β-Phenyllactic acid and L-alanine. β-Phenyllactic acid (β-PLA) and L-alanine (ala) were dried down at a 5:1 molar ratio, in favor of β-PLA, for 7 days at 85 °C. Dry down products were then resuspended in an aqueous solution of 20% acetonitrile in water (v/v). The resulting products were analyzed by negative-mode ESI-MS, indicating a variety of depsipeptides. β-PLA is labeled in green, ala is labeled in red.

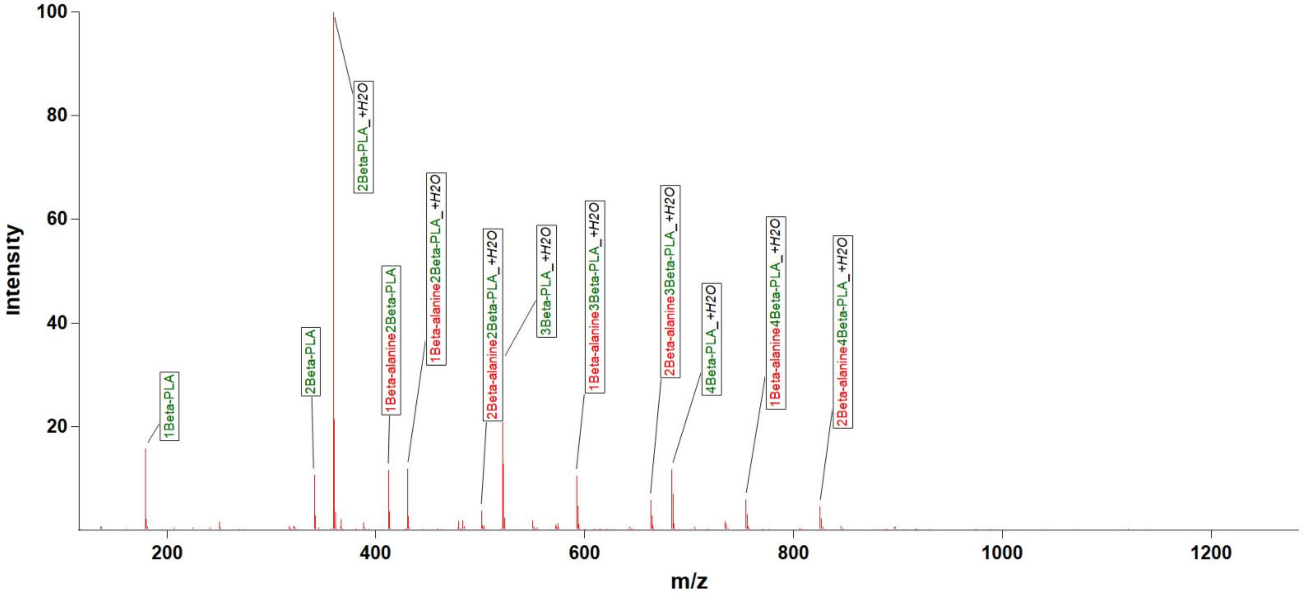

Supplementary Figure S151. ESI-MS spectrum of a dry-down reaction of sample NA15 – β-Phenyllactic acid and β-alanine. β-Phenyllactic acid (β-PLA) and β-alanine (β-ala) were dried down at a 5:1 molar ratio, in favor of β-PLA, for 7 days at 85 °C. Dry down products were then resuspended in an aqueous solution of 20% acetonitrile in water (v/v). The resulting products were analyzed by negative-mode ESI-MS, indicating a variety of depsipeptides. β-PLA is labeled in green, β-ala is labeled in red.

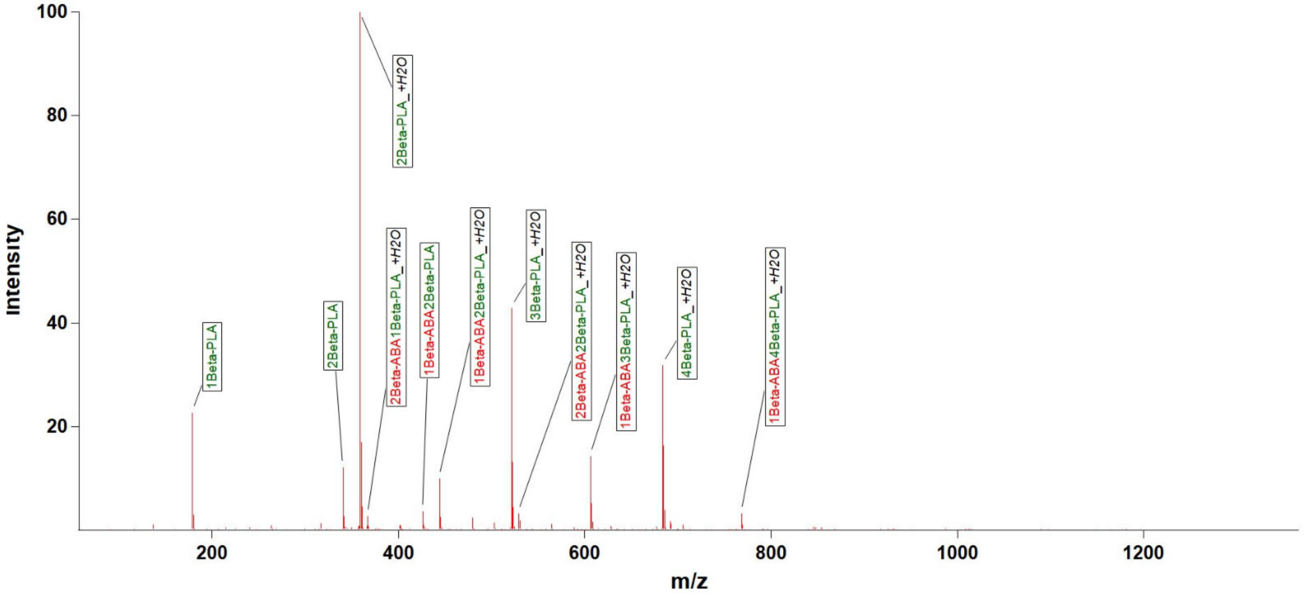

Supplementary Figure S152. ESI-MS spectrum of a dry-down reaction of sample NA16 – β-Phenyllactic acid and β-aminobutyric acid. β-Phenyllactic acid (β-PLA) and β-aminobutyric acid (β-ABA) were dried down at a 5:1 molar ratio, in favor of β-PLA, for 7 days at 85 °C. Dry down products were then resuspended in an aqueous solution of 20% acetonitrile in water (v/v). The resulting products were analyzed by negative-mode ESI-MS, indicating a variety of depsipeptides. β-PLA is labeled in green, β-ABA is labeled in red.

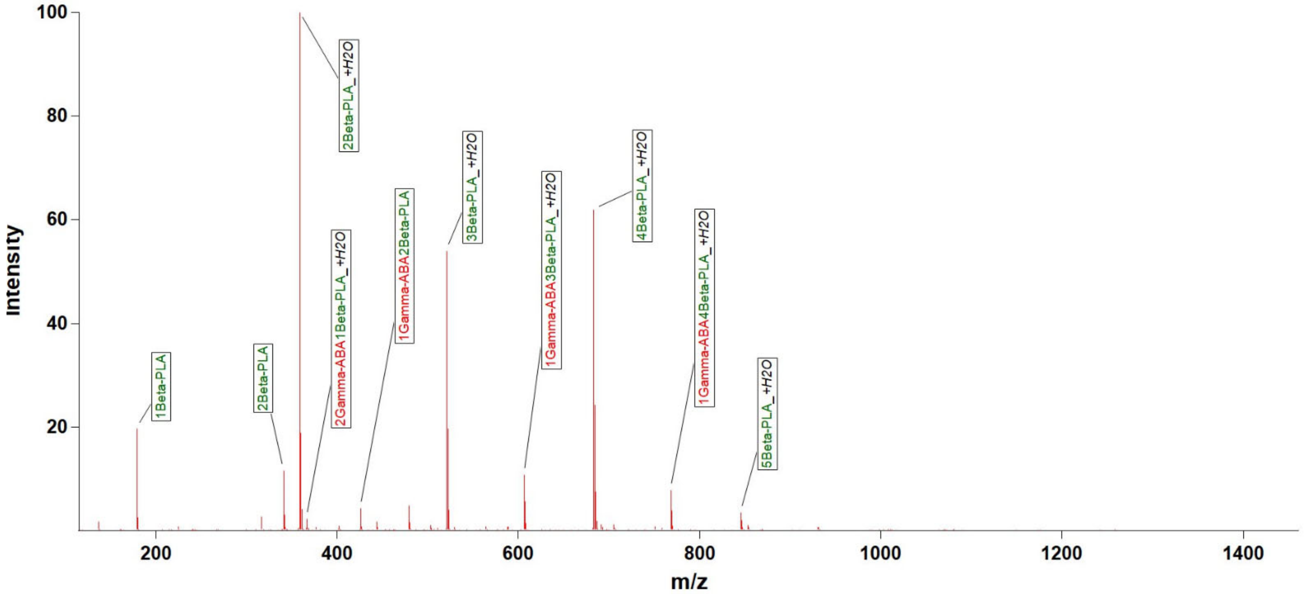

Supplementary Figure S153. ESI-MS spectrum of a dry-down reaction of sample NA17 – β-Phenyllactic acid and γ-aminobutyric acid. β-Phenyllactic acid (β-PLA) and γ-aminobutyric acid (γ-ABA) were dried down at a 5:1 molar ratio, in favor of β-PLA, for 7 days at 85 °C. Dry down products were then resuspended in an aqueous solution of 20% acetonitrile in water (v/v). The resulting products were analyzed by negative-mode ESI-MS, indicating a variety of depsipeptides. β-PLA is labeled in green, γ-ABA is labeled in red.

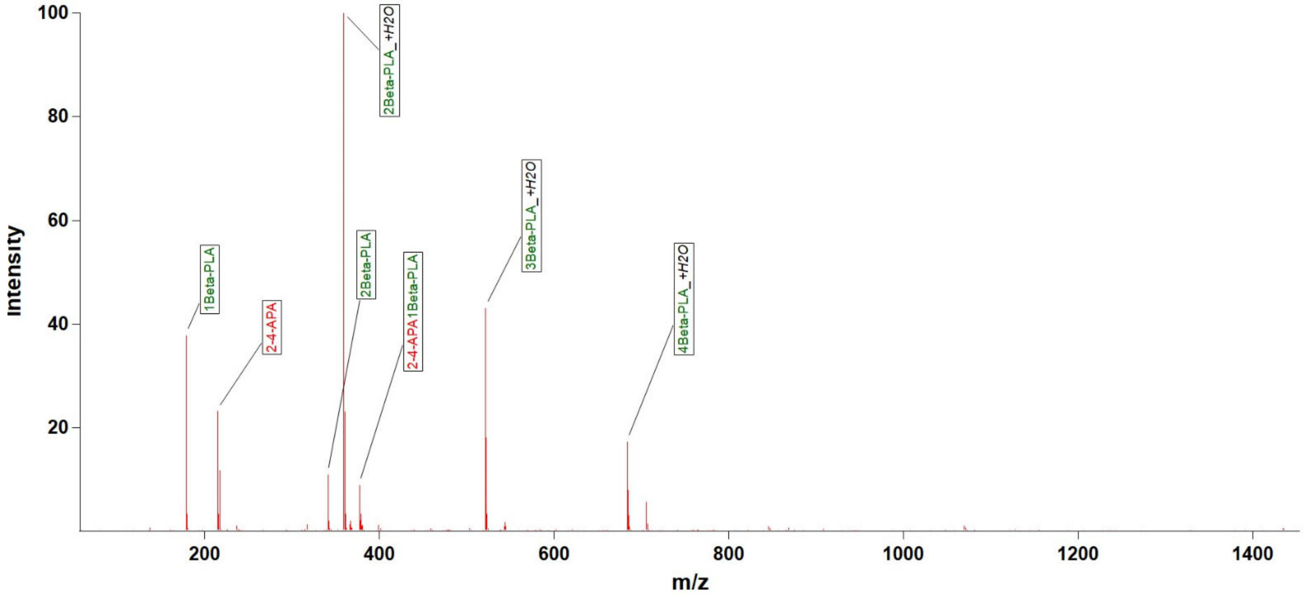

Supplementary Figure S154. ESI-MS spectrum of a dry-down reaction of sample NA18 – β-Phenyllactic acid and γ-aminopentanoic acid. β-Phenyllactic acid (β-PLA) and 4-aminopentanoic acid (γ-APA) were dried down at a 5:1 molar ratio, in favor of β-PLA, for 7 days at 85 °C. Dry down products were then resuspended in an aqueous solution of 20% acetonitrile in water (v/v). The resulting products were analyzed by negative-mode ESI-MS, indicating a variety of depsipeptides. β-PLA is labeled in green, γ-APA is labeled in red.

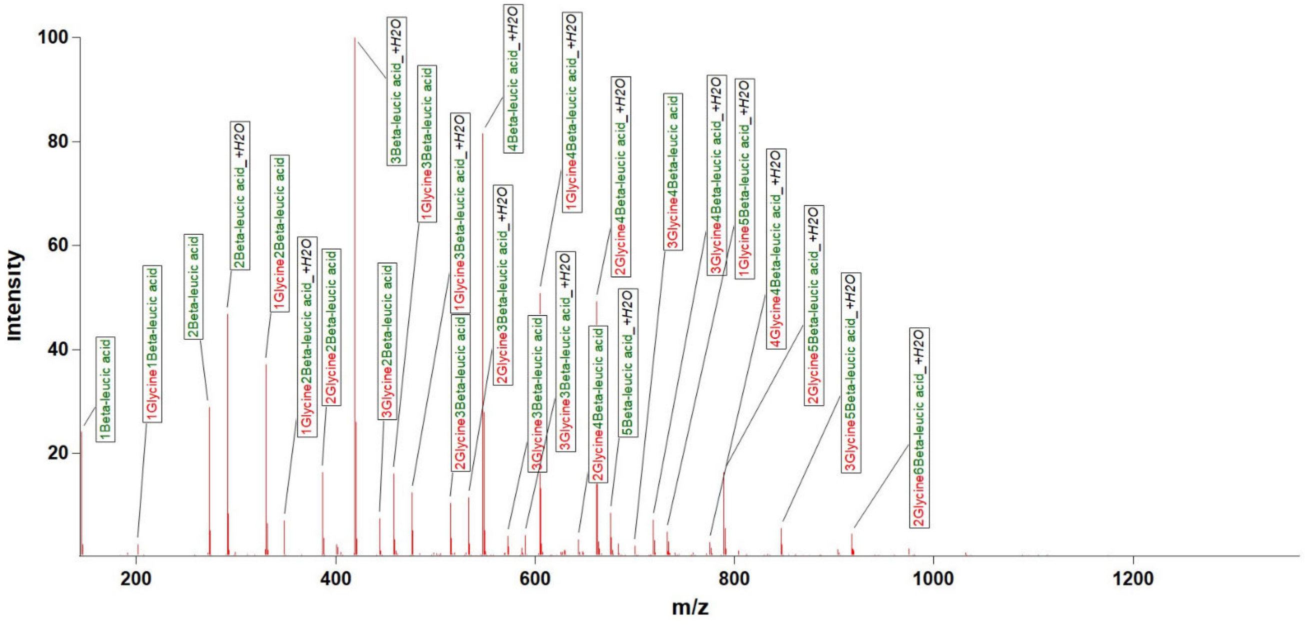

Supplementary Figure S155. ESI-MS spectrum of a dry-down reaction of sample NA19 – β-Leucic acid and Glycine. β-Leucic acid (β-LA) and Glycine (gly) were dried down at a 5:1 molar ratio, in favor of β-LA, for 7 days at 85 °C. Dry down products were then resuspended in an aqueous solution of 20% acetonitrile in water (v/v). The resulting products were analyzed by negative-mode ESI-MS, indicating a variety of depsipeptides. β-LA is labeled in green, gly is labeled in red.

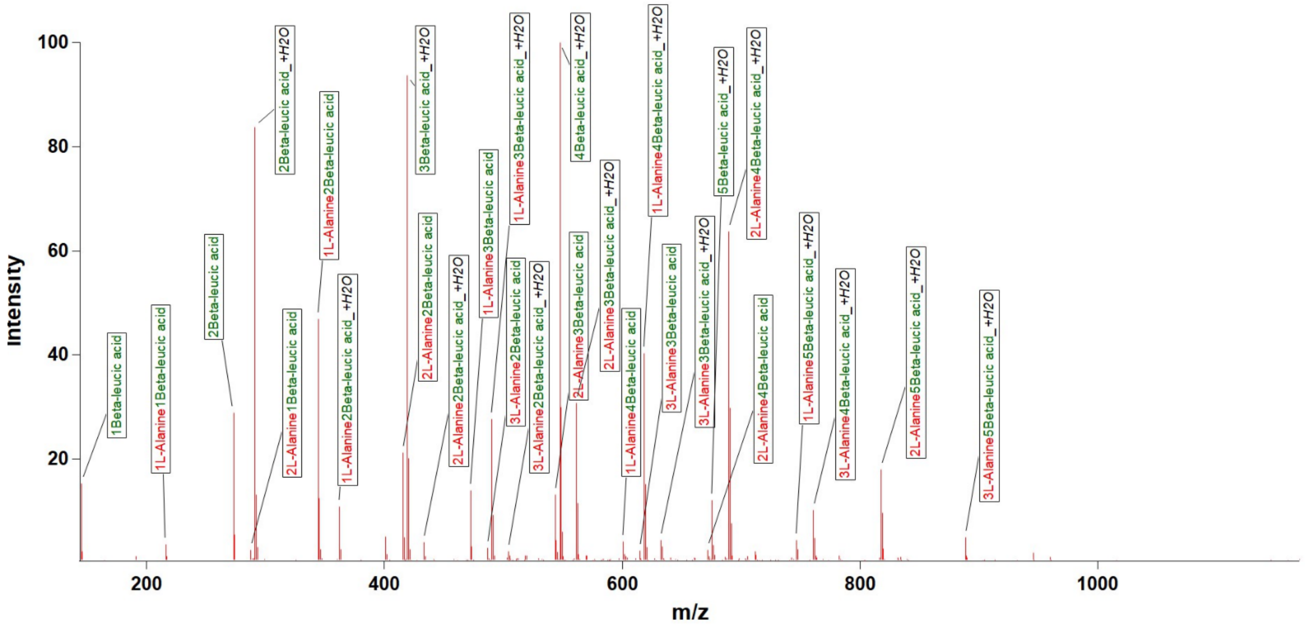

Supplementary Figure S156. ESI-MS spectrum of a dry-down reaction of sample NA20 – β-Leucic acid and L-alanine. β-Leucic acid (β-LA) and L-alanine (ala) were dried down at a 5:1 molar ratio, in favor of β-LA, for 7 days at 85 °C. Dry down products were then resuspended in an aqueous solution of 20% acetonitrile in water (v/v). The resulting products were analyzed by negative-mode ESI-MS, indicating a variety of depsipeptides. β-LA is labeled in green, ala is labeled in red.

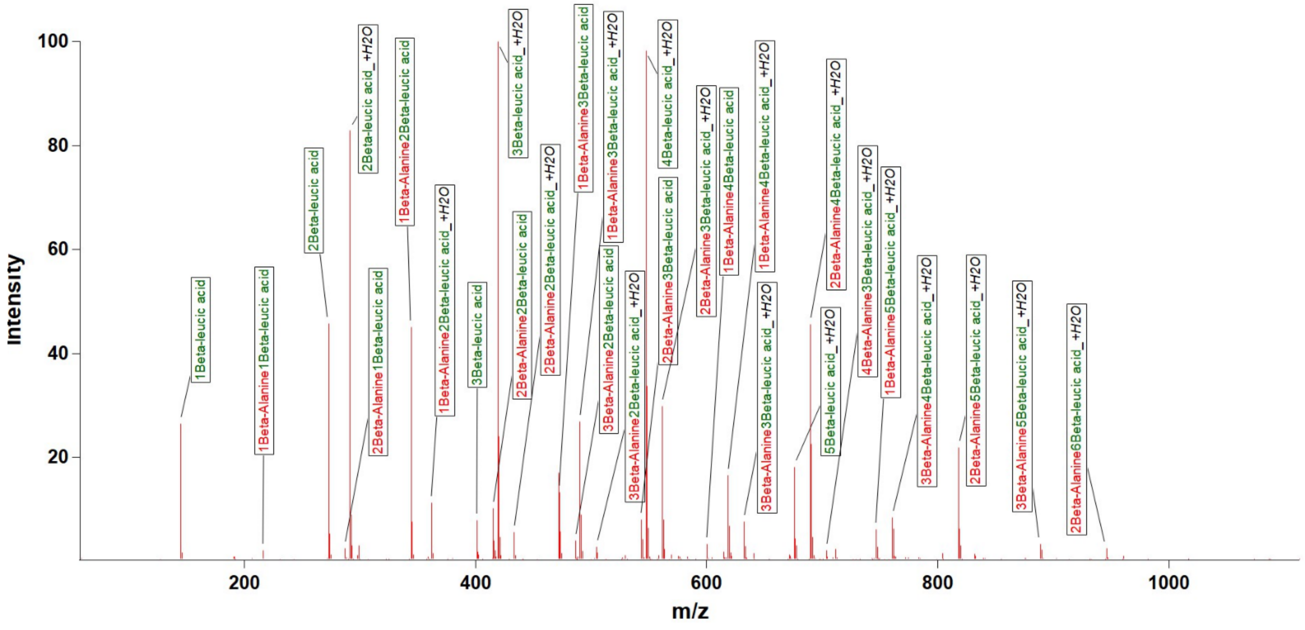

Supplementary Figure S157. ESI-MS spectrum of a dry-down reaction of sample NA21 – β-Leucic acid and β-alanine. β-Leucic acid (β-LA) and β-alanine (β-ala) were dried down at a 5:1 molar ratio, in favor of β-LA, for 7 days at 85 °C. Dry down products were then resuspended in an aqueous solution of 20% acetonitrile in water (v/v). The resulting products were analyzed by negative-mode ESI-MS, indicating a variety of depsipeptides. β-LA is labeled in green, β-ala is labeled in red.

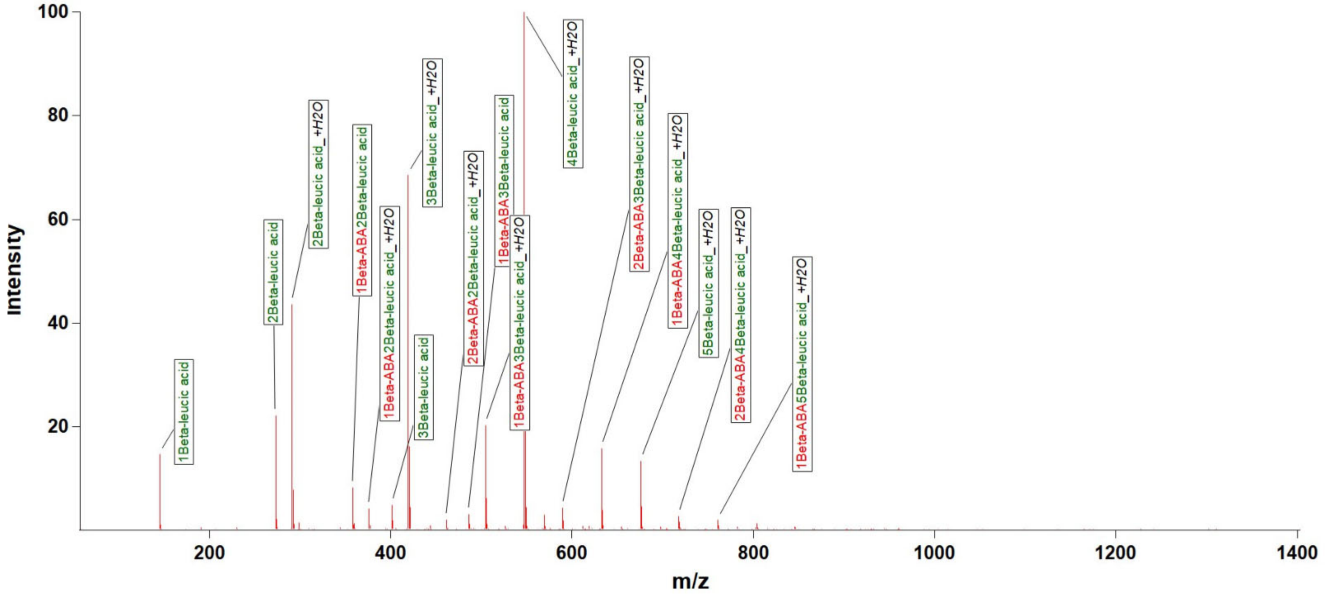

Supplementary Figure S158. ESI-MS spectrum of a dry-down reaction of sample NA22 – β-Leucic acid and β-aminobutyric acid. β-Leucic acid (β-LA) and β-aminobutyric acid (β-ABA) were dried down at a 5:1 molar ratio, in favor of β-LA, for 7 days at 85 °C. Dry down products were then resuspended in an aqueous solution of 20% acetonitrile in water (v/v). The resulting products were analyzed by negative-mode ESI-MS, indicating a variety of depsipeptides. β-LA is labeled in green, β-ABA is labeled in red.

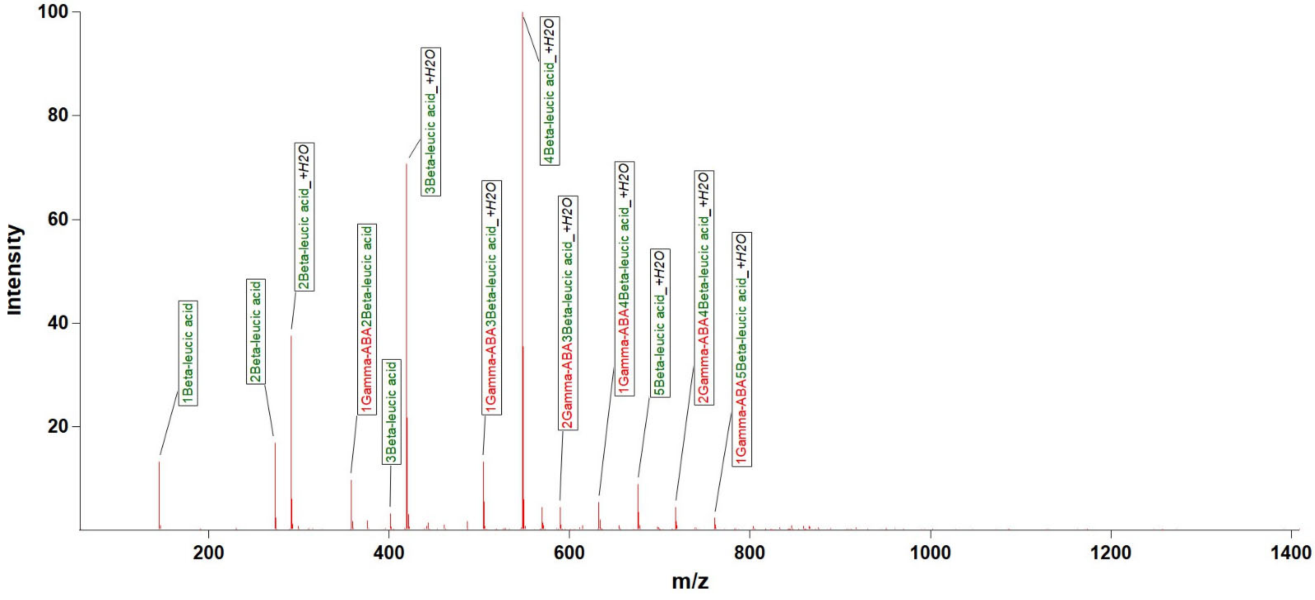

Supplementary Figure S159. ESI-MS spectrum of a dry-down reaction of sample NA23 – β-Leucic acid and γ-aminobutyric acid. β-Leucic acid (β-LA) and γ-aminobutyric acid (γ-ABA) were dried down at a 5:1 molar ratio, in favor of β-LA, for 7 days at 85 °C. Dry down products were then resuspended in an aqueous solution of 20% acetonitrile in water (v/v). The resulting products were analyzed by negative-mode ESI-MS, indicating a variety of depsipeptides. β-LA is labeled in green, γ-ABA is labeled in red.

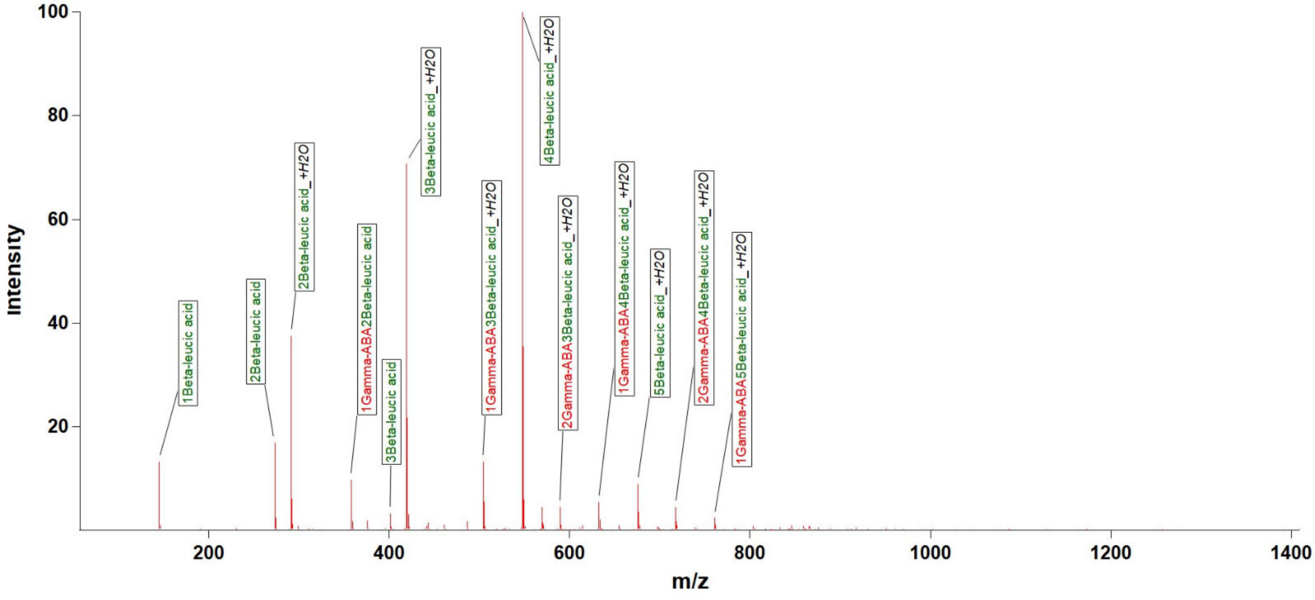

Supplementary Figure S160. ESI-MS spectrum of a dry-down reaction of sample NA24 – β-Leucic acid and γ-aminopentanoic acid. β-Leucic acid (β-LA) and 4-aminopentanoic acid (γ-APA) were dried down at a 5:1 molar ratio, in favor of β-LA, for 7 days at 85 °C. Dry down products were then resuspended in an aqueous solution of 20% acetonitrile in water (v/v). The resulting products were analyzed by negative-mode ESI-MS, indicating a variety of depsipeptides. β-LA is labeled in green, γ-APA is labeled in red.

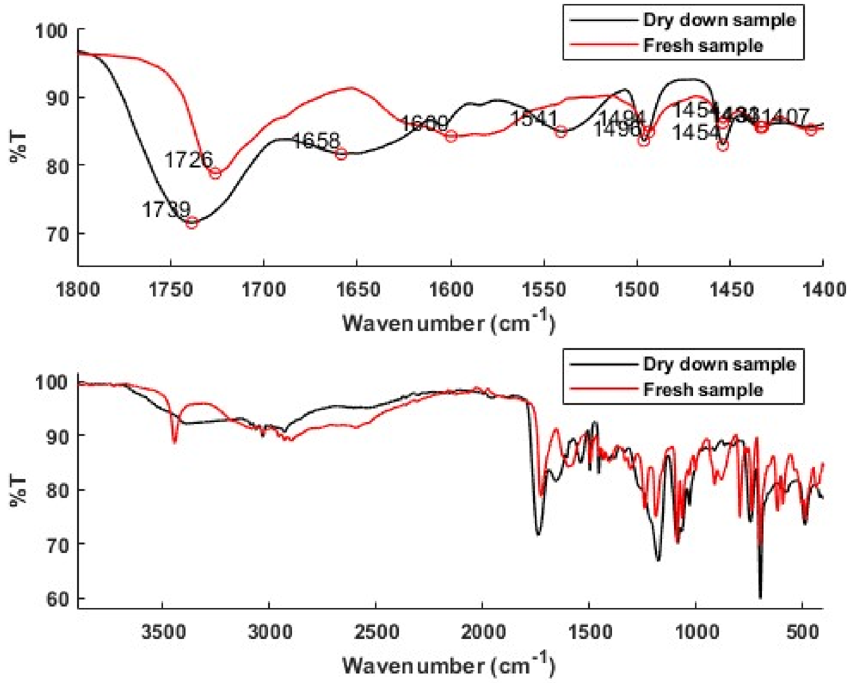

Supplementary Figure S161. FTIR spectrum of sample NA1- Phenyllactic acid and Glycine. in black sample was dried for 7 days in 85 °C, in red fresh stock solution samples. Phenyllactic acid (PLA) and Glycine were dried down at a 5:1 molar ratio, in favor of PLA. Dry down products were then resuspended in an aqueous solution of 20% acetonitrile in water (v/v). The resulting products were analyzed by ATR-FTIR.

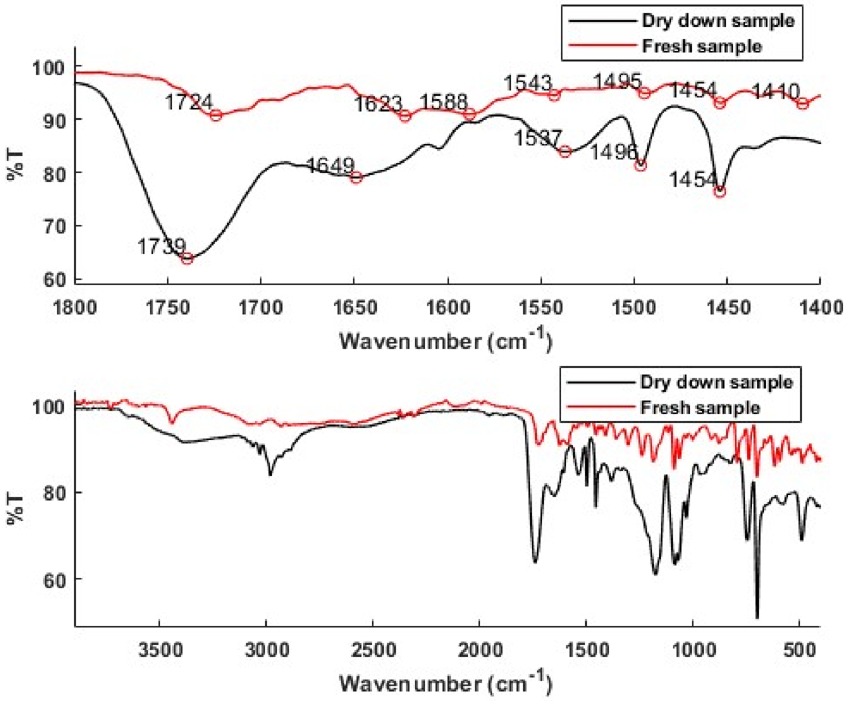

Supplementary Figure S162. FTIR spectrum of sample NA2- Phenyllactic acid and L-alanine. in black sample was dried for 7 days in 85 °C, in red fresh stock solution samples. Phenyllactic acid (PLA) and L-alanine were dried down at a 5:1 molar ratio, in favor of PLA. Dry down products were then resuspended in an aqueous solution of 20% acetonitrile in water (v/v). The resulting products were analyzed by ATR-FTIR.

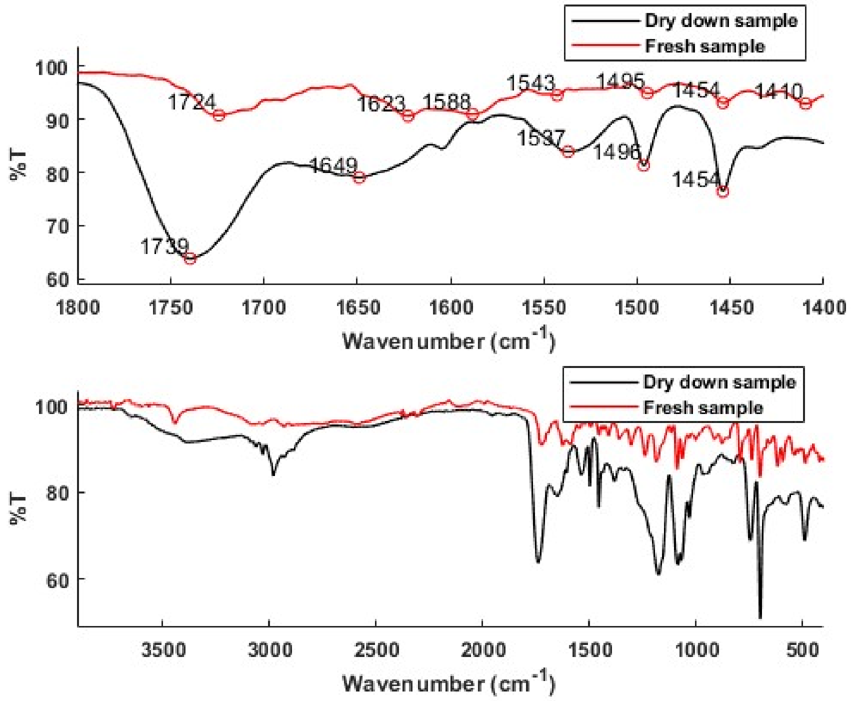

Supplementary Figure S163. FTIR spectrum of sample NA3- Phenyllactic acid and β-alanine. in black sample was dried for 7 days in 85 °C, in red fresh stock solution samples. Phenyllactic acid (PLA) and β-alanine were dried down at a 5:1 molar ratio, in favor of PLA. Dry down products were then resuspended in an aqueous solution of 20% acetonitrile in water (v/v). The resulting products were analyzed by ATR-FTIR.

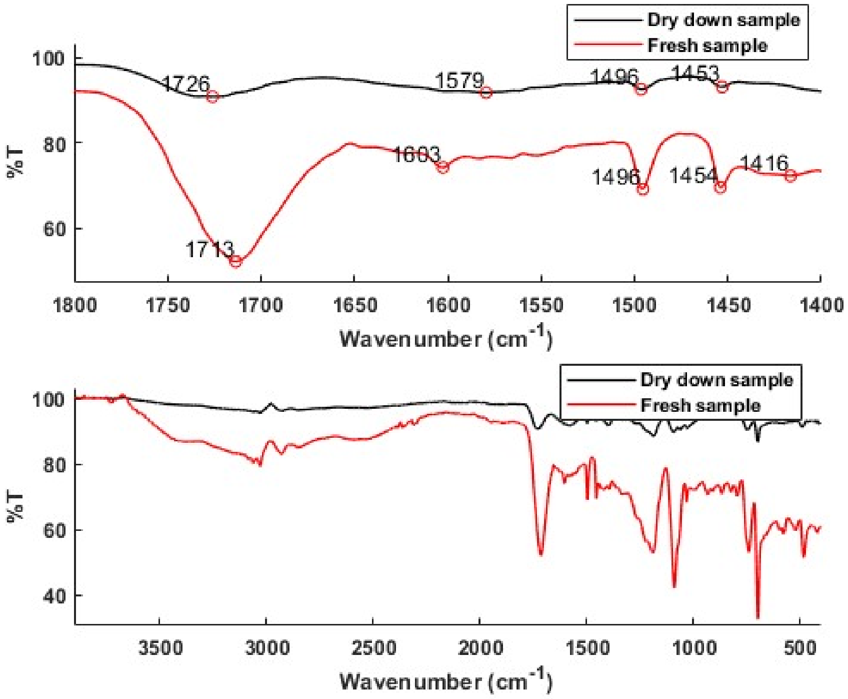

Supplementary Figure S164. FTIR spectrum of sample NA4- Phenyllactic acid and β-aminobutyric acid. in black sample was dried for 7 days in 85 °C, in red fresh stock solution samples. Phenyllactic acid (PLA) and β-aminobutyric acid were dried down at a 5:1 molar ratio, in favor of PLA. Dry down products were then resuspended in an aqueous solution of 20% acetonitrile in water (v/v). The resulting products were analyzed by ATR-FTIR.

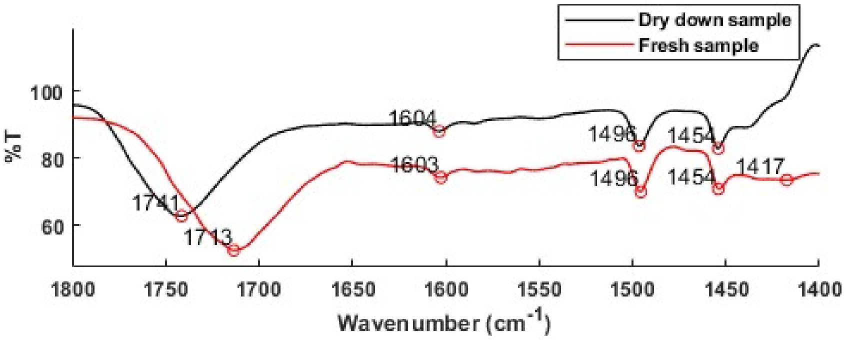

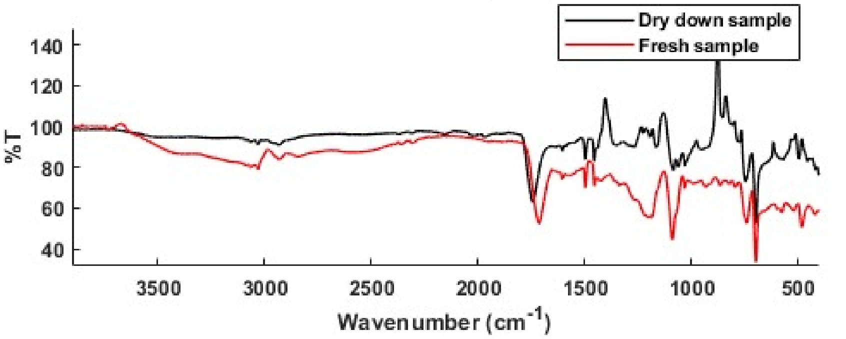

Supplementary Figure S165. FTIR spectrum of sample NA5- Phenyllactic acid and γ-aminobutyric acid. in black sample was dried for 7 days in 85 °C, in red fresh stock solution samples. Phenyllactic acid (PLA) and γ-aminobutyric acid were dried down at a 5:1 molar ratio, in favor of PLA. Dry down products were then resuspended in an aqueous solution of 20% acetonitrile in water (v/v). The resulting products were analyzed by ATR-FTIR.

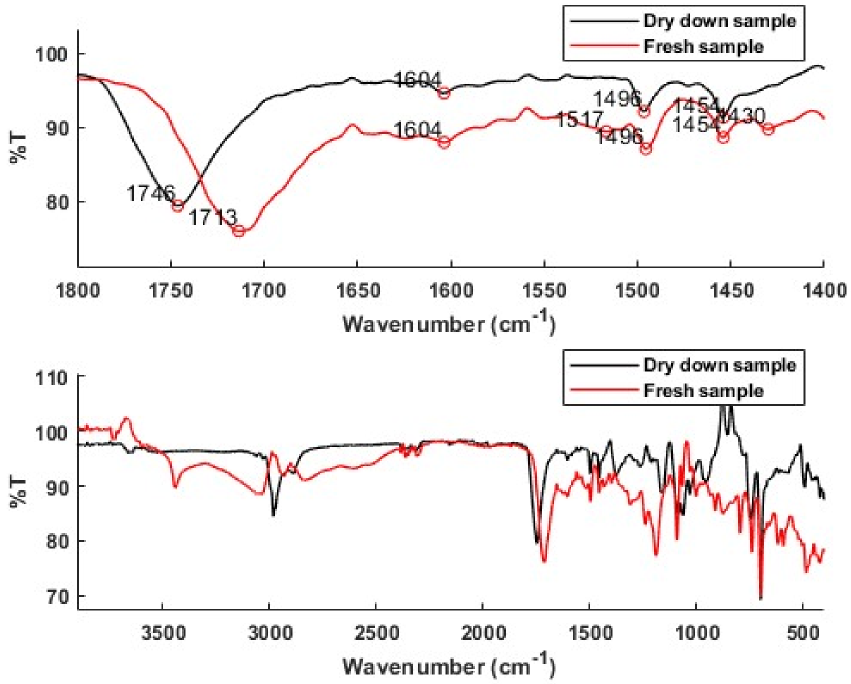

Supplementary Figure S166. FTIR spectrum of sample NA6- Phenyllactic acid and γ-aminopentanoic acid. in black sample was dried for 7 days in 85 °C, in red fresh stock solution samples. Phenyllactic acid (PLA) and γ-aminopentanoic acid were dried down at a 5:1 molar ratio, in favor of PLA. Dry down products were then resuspended in an aqueous solution of 20% acetonitrile in water (v/v). The resulting products were analyzed by ATR-FTIR.

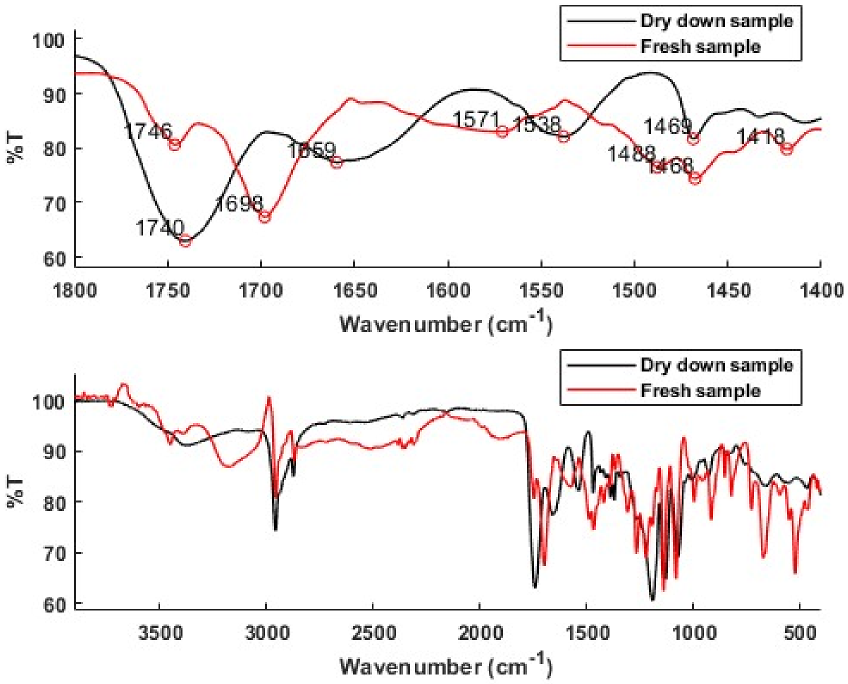

Supplementary Figure S167. FTIR spectrum of sample NA7- Leucic acid and Glycine. in black sample was dried for 7 days in 85 °C, in red fresh stock solution samples. Leucic acid (LA) and Glycine were dried down at a 5:1 molar ratio, in favor of LA. Dry down products were then resuspended in an aqueous solution of 20% acetonitrile in water (v/v). The resulting products were analyzed by ATR-FTIR.

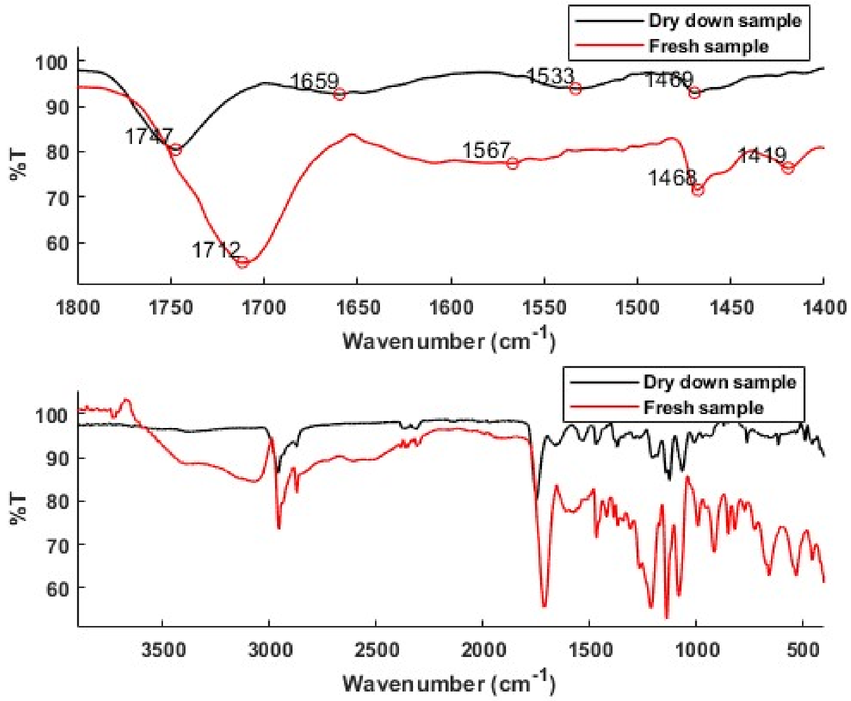

Supplementary Figure S168. FTIR spectrum of sample NA8- Leucic acid and L-alanine. in black sample was dried for 7 days in 85 °C, in red fresh stock solution samples. Leucic acid (LA) and L-alanine were dried down at a 5:1 molar ratio, in favor of LA. Dry down products were then resuspended in an aqueous solution of 20% acetonitrile in water (v/v). The resulting products were analyzed by ATR-FTIR.

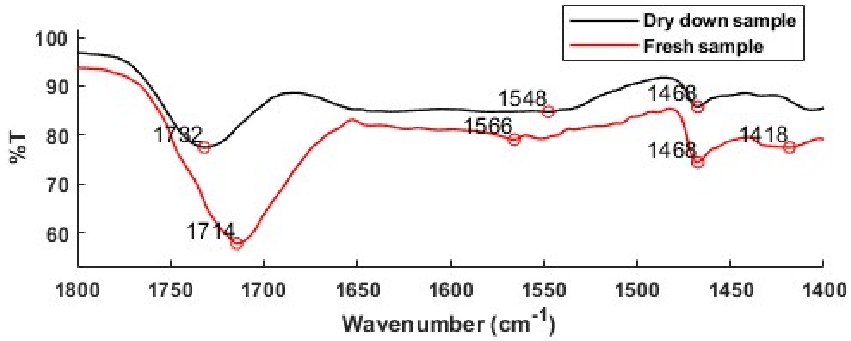

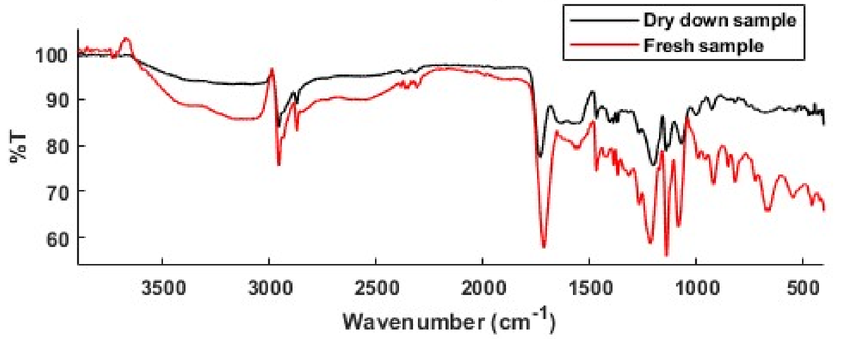

Supplementary Figure S169. FTIR spectrum of sample NA9- Leucic acid and β-alanine. in black sample was dried for 7 days in 85 °C, in red fresh stock solution samples. Leucic acid (LA) and β-alanine were dried down at a 5:1 molar ratio, in favor of LA. Dry down products were then resuspended in an aqueous solution of 20% acetonitrile in water (v/v). The resulting products were analyzed by ATR-FTIR.

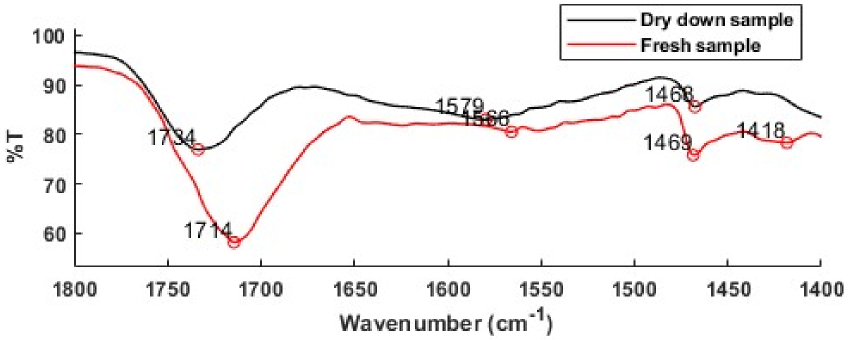

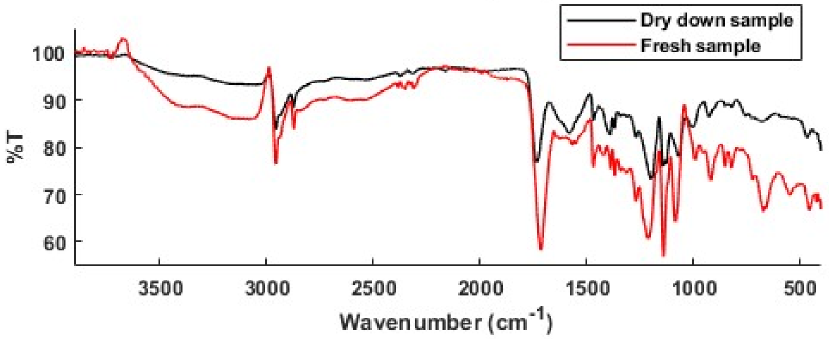

Supplementary Figure S170. FTIR spectrum of sample NA10- Leucic acid and β-aminobutyric acid. in black sample was dried for 7 days in 85 °C, in red fresh stock solution samples. Leucic acid (LA) and β-aminobutyric acid were dried down at a 5:1 molar ratio, in favor of LA. Dry down products were then resuspended in an aqueous solution of 20% acetonitrile in water (v/v). The resulting products were analyzed by ATR-FTIR.

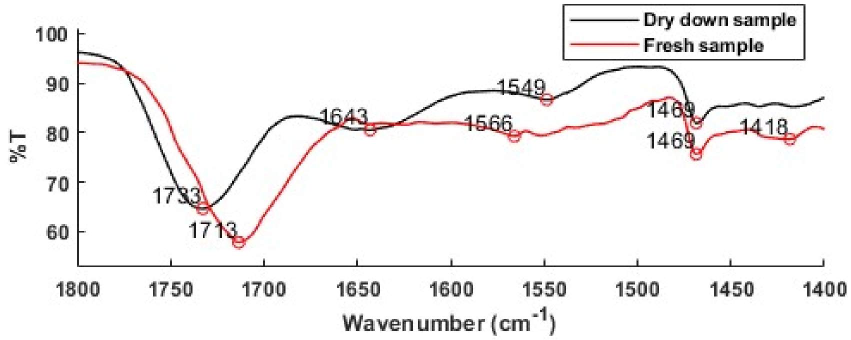

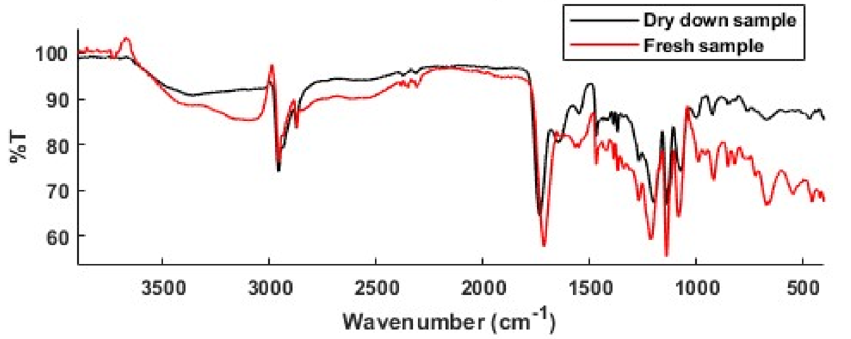

Supplementary Figure S171. FTIR spectrum of sample NA11- Leucic acid and γ-aminobutyric acid. in black sample was dried for 7 days in 85 °C, in red fresh stock solution samples. Leucic acid (LA) and γ-aminobutyric acid were dried down at a 5:1 molar ratio, in favor of LA. Dry down products were then resuspended in an aqueous solution of 20% acetonitrile in water (v/v). The resulting products were analyzed by ATR-FTIR.

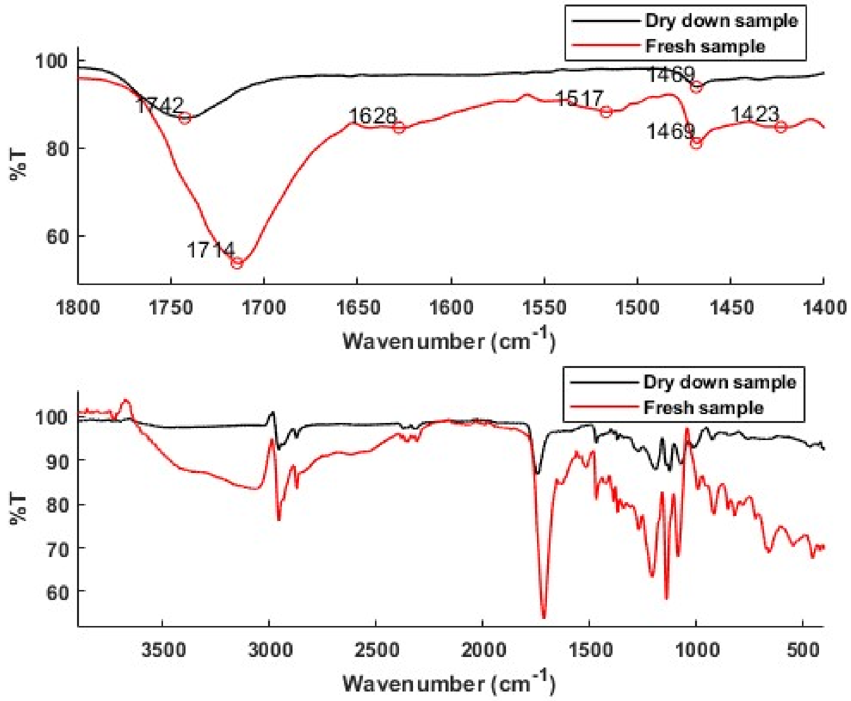

Supplementary Figure S172. FTIR spectrum of sample NA12- Leucic acid and γ-aminopentanoic acid. in black sample was dried for 7 days in 85 °C, in red fresh stock solution samples. Leucic acid (LA) and γ-aminopentanoic acid were dried down at a 5:1 molar ratio, in favor of LA. Dry down products were then resuspended in an aqueous solution of 20% acetonitrile in water (v/v). The resulting products were analyzed by ATR-FTIR.

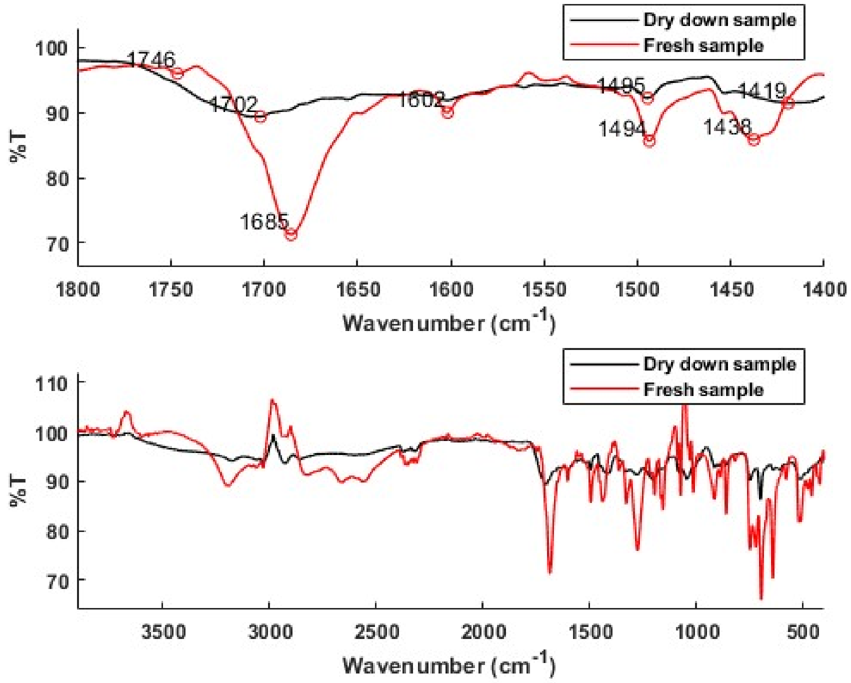

Supplementary Figure S173. FTIR spectrum of sample NA13- β-Phenyllactic acid and Glycine. in black sample was dried for 7 days in 85 °C, in red fresh stock solution samples. β-Phenyllactic acid (β-PLA) and Glycine were dried down at a 5:1 molar ratio, in favor of β-PLA. Dry down products were then resuspended in an aqueous solution of 20% acetonitrile in water (v/v). The resulting products were analyzed by ATR-FTIR.

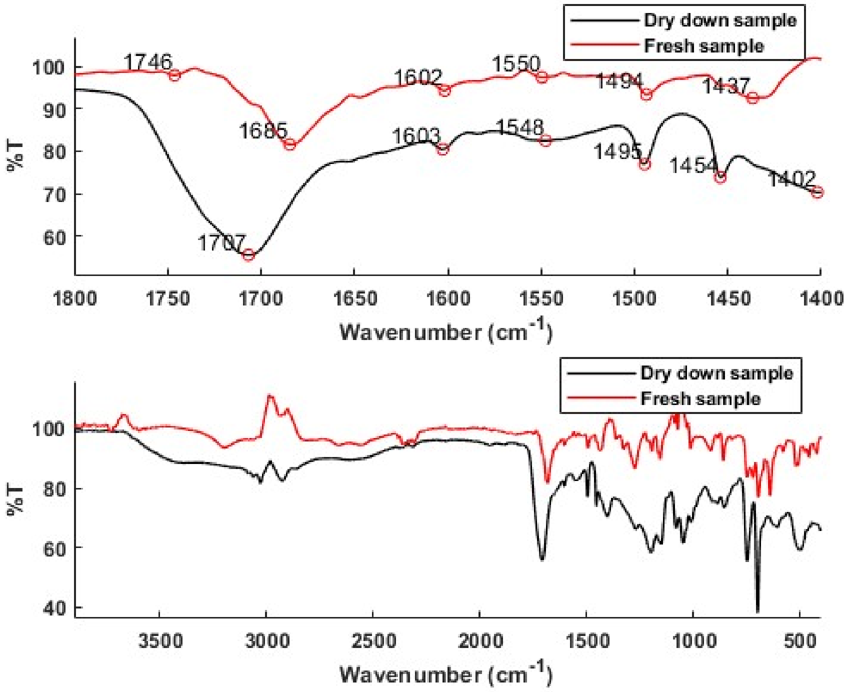

Supplementary Figure S174. FTIR spectrum of sample NA14- β-Phenyllactic acid and L-alanine. in black sample was dried for 7 days in 85 °C, in red fresh stock solution samples. β-Phenyllactic acid (β-PLA) and L-alanine were dried down at a 5:1 molar ratio, in favor of β-PLA. Dry down products were then resuspended in an aqueous solution of 20% acetonitrile in water (v/v). The resulting products were analyzed by ATR-FTIR.

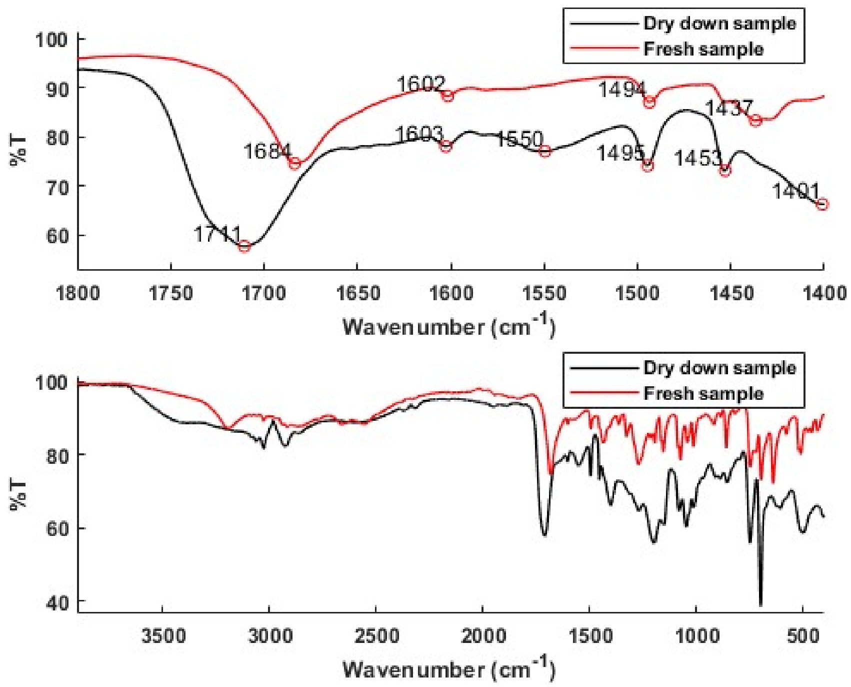

Supplementary Figure S175. FTIR spectrum of sample NA15- β-Phenyllactic acid and β-alanine. in black sample was dried for 7 days in 85 °C, in red fresh stock solution samples. β-Phenyllactic acid (β-PLA) and β-alanine were dried down at a 5:1 molar ratio, in favor of β-PLA. Dry down products were then resuspended in an aqueous solution of 20% acetonitrile in water (v/v). The resulting products were analyzed by ATR-FTIR.

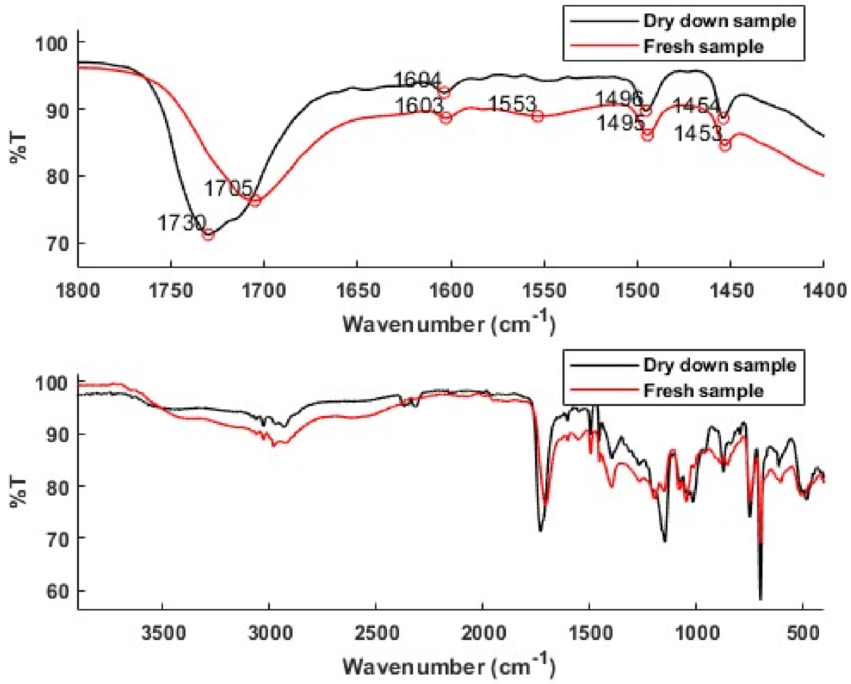

Supplementary Figure S176. FTIR spectrum of sample NA16- β-Phenyllactic acid and β-aminobutyric acid. in black sample was dried for 7 days in 85 °C, in red fresh stock solution samples. β-Phenyllactic acid (β-PLA) and β-aminobutyric acid were dried down at a 5:1 molar ratio, in favor of β-PLA. Dry down products were then resuspended in an aqueous solution of 20% acetonitrile in water (v/v). The resulting products were analyzed by ATR-FTIR.

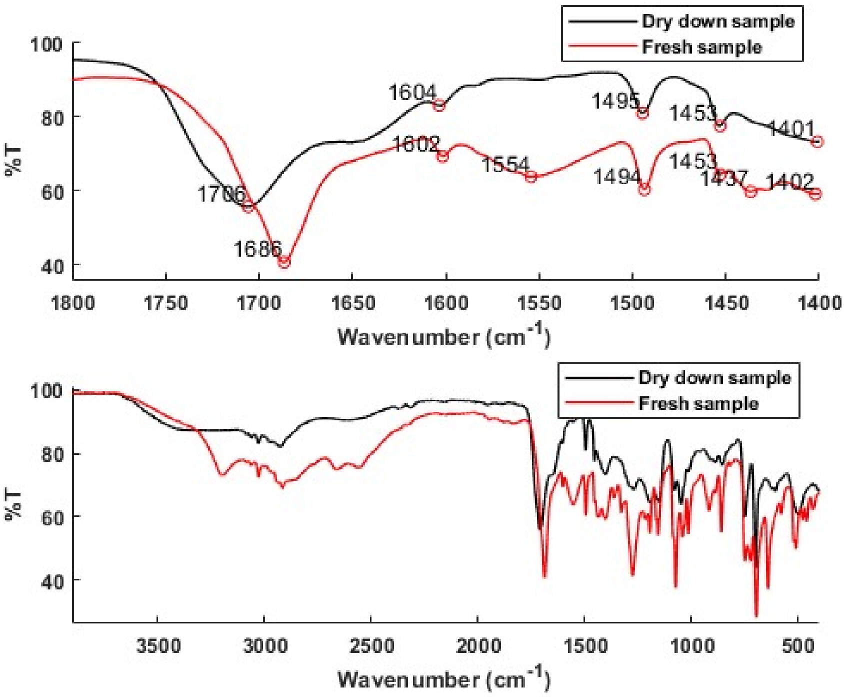

Supplementary Figure S177. FTIR spectrum of sample NA17- β-Phenyllactic acid and γ-aminobutyric acid. in black sample was dried for 7 days in 85 °C, in red fresh stock solution samples. β-Phenyllactic acid (β-PLA) and γ-aminobutyric acid were dried down at a 5:1 molar ratio, in favor of β-PLA. Dry down products were then resuspended in an aqueous solution of 20% acetonitrile in water (v/v). The resulting products were analyzed by ATR-FTIR.

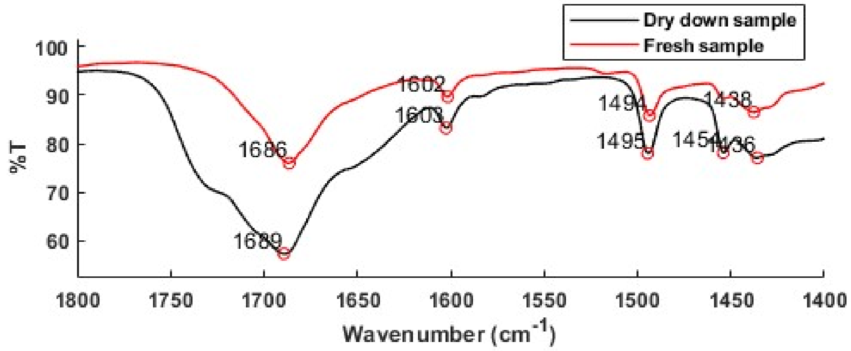

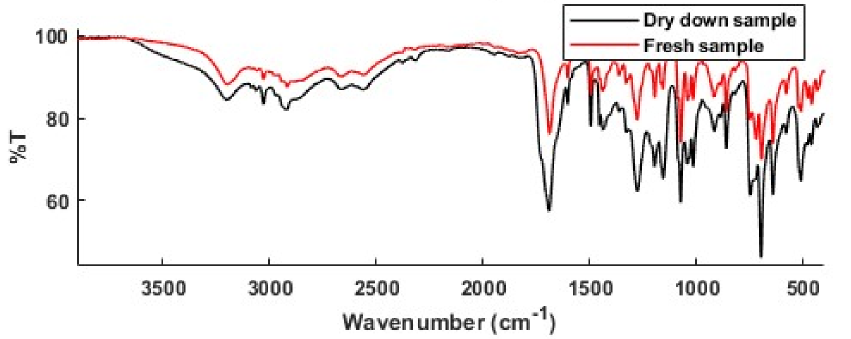

Supplementary Figure S178. FTIR spectrum of sample NA18- β-Phenyllactic acid and γ-aminopentanoic acid. in black sample was dried for 7 days in 85 °C, in red fresh stock solution samples. β-Phenyllactic acid (β-PLA) and γ-aminopentanoic acid were dried down at a 5:1 molar ratio, in favor of β-PLA. Dry down products were then resuspended in an aqueous solution of 20% acetonitrile in water (v/v). The resulting products were analyzed by ATR-FTIR.

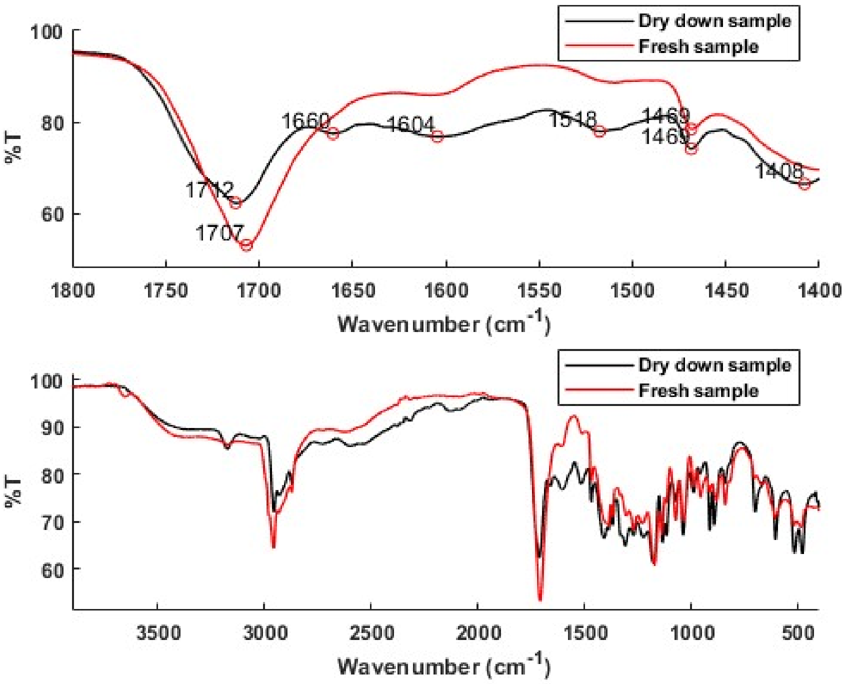

Supplementary Figure S179. FTIR spectrum of sample NA19- β-Leucic acid and Glycine. in black sample was dried for 7 days in 85 °C, in red fresh stock solution samples. β-Leucic acid (β-LA) and Glycine were dried down at a 5:1 molar ratio, in favor of β-LA. Dry down products were then resuspended in an aqueous solution of 20% acetonitrile in water (v/v). The resulting products were analyzed by ATR-FTIR.

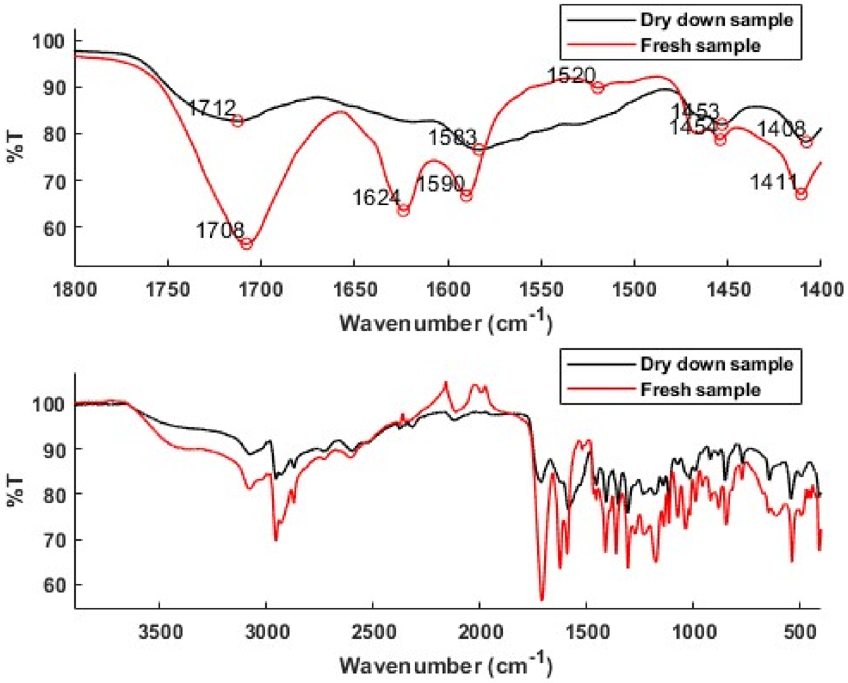

Supplementary Figure S180. FTIR spectrum of sample NA20- β-Leucic acid and L-alanine. in black sample was dried for 7 days in 85 °C, in red fresh stock solution samples. β-Leucic acid (β-LA) and L-alanine were dried down at a 5:1 molar ratio, in favor of β-LA. Dry down products were then resuspended in an aqueous solution of 20% acetonitrile in water (v/v). The resulting products were analyzed by ATR-FTIR.

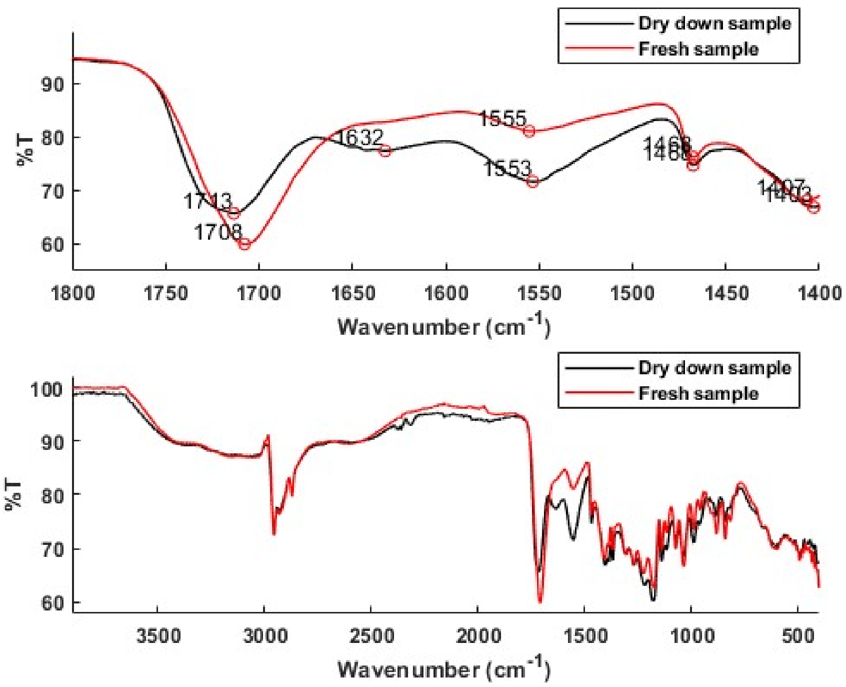

Supplementary Figure S181. FTIR spectrum of sample NA21- β-Leucic acid and β-alanine. in black sample was dried for 7 days in 85 °C, in red fresh stock solution samples. β-Leucic acid (β-LA) and β-alanine were dried down at a 5:1 molar ratio, in favor of β-LA. Dry down products were then resuspended in an aqueous solution of 20% acetonitrile in water (v/v). The resulting products were analyzed by ATR-FTIR.

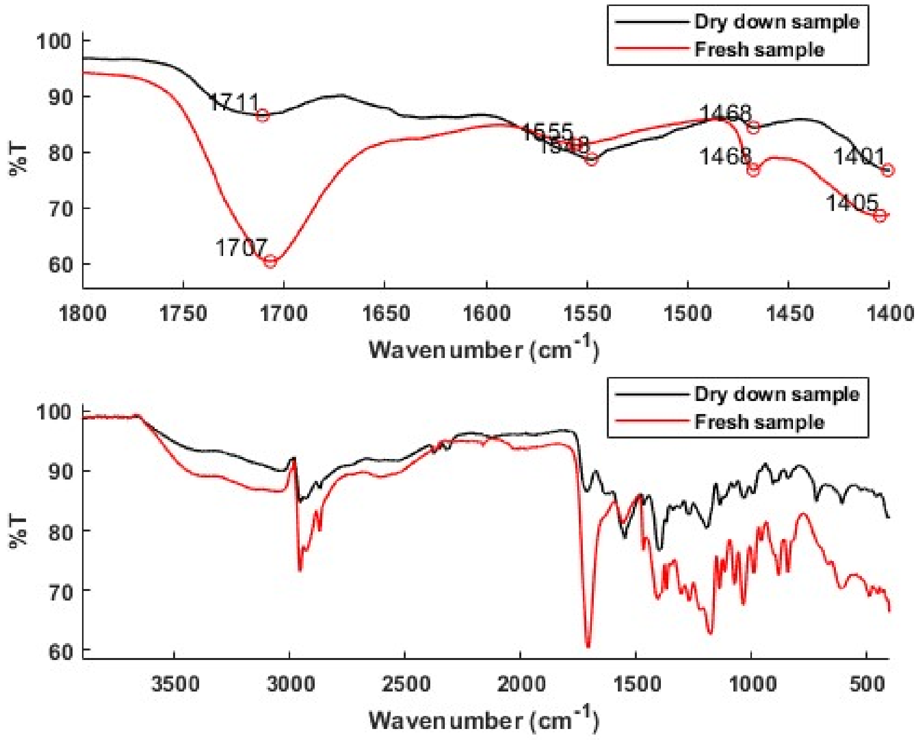

Supplementary Figure S182. FTIR spectrum of sample NA22- β-Leucic acid and β-aminobutyric acid. in black sample was dried for 7 days in 85 °C, in red fresh stock solution samples. β-Leucic acid (β-LA) and β-aminobutyric acid were dried down at a 5:1 molar ratio, in favor of β-LA. Dry down products were then resuspended in an aqueous solution of 20% acetonitrile in water (v/v). The resulting products were analyzed by ATR-FTIR.

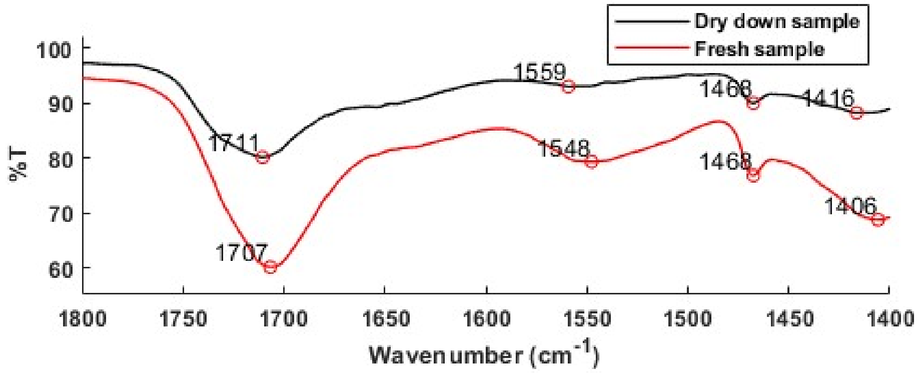

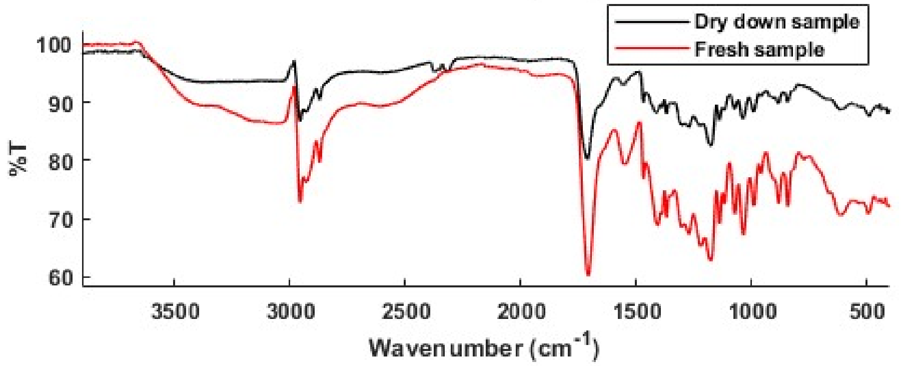

Supplementary Figure S183. FTIR spectrum of sample NA23- β-Leucic acid and γ-aminobutyric acid. in black sample was dried for 7 days in 85 °C, in red fresh stock solution samples. β-Leucic acid (β-LA) and γ-aminobutyric acid were dried down at a 5:1 molar ratio, in favor of β-LA. Dry down products were then resuspended in an aqueous solution of 20% acetonitrile in water (v/v). The resulting products were analyzed by ATR-FTIR.

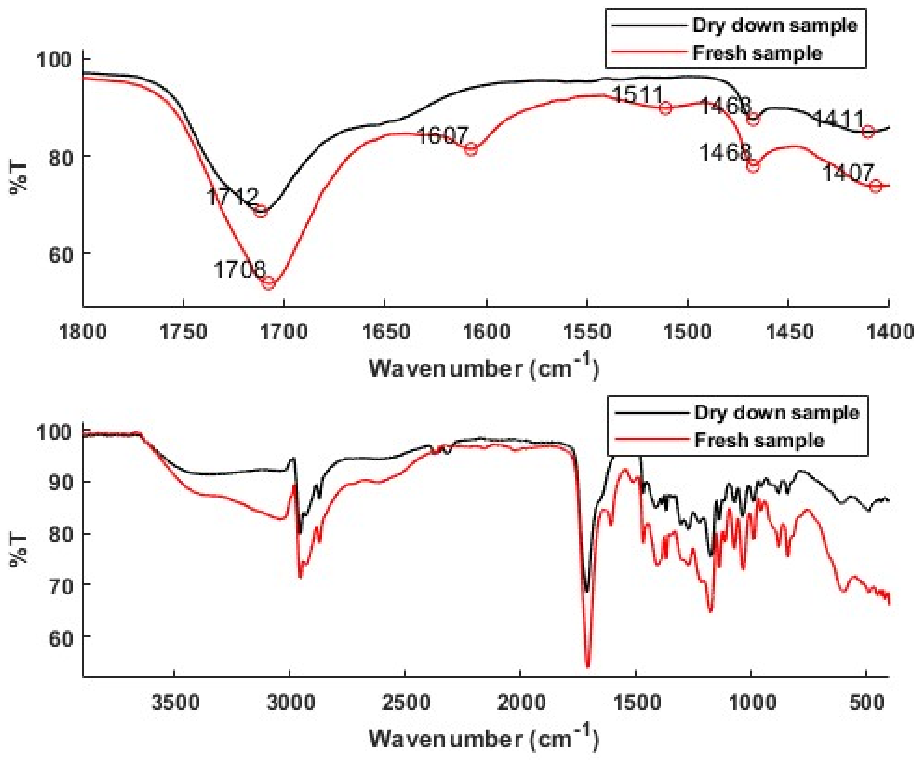

Supplementary Figure S184. FTIR spectrum of sample NA24- β-Leucic acid and 4-aminopentanoic acid. in black sample was dried for 7 days in 85 °C, in red fresh stock solution samples. β-Leucic acid (β-LA) and 4-aminopentanoic acid were dried down at a 5:1 molar ratio, in favor of β-LA. Dry down products were then resuspended in an aqueous solution of 20% acetonitrile in water (v/v). The resulting products were analyzed by ATR-FTIR.

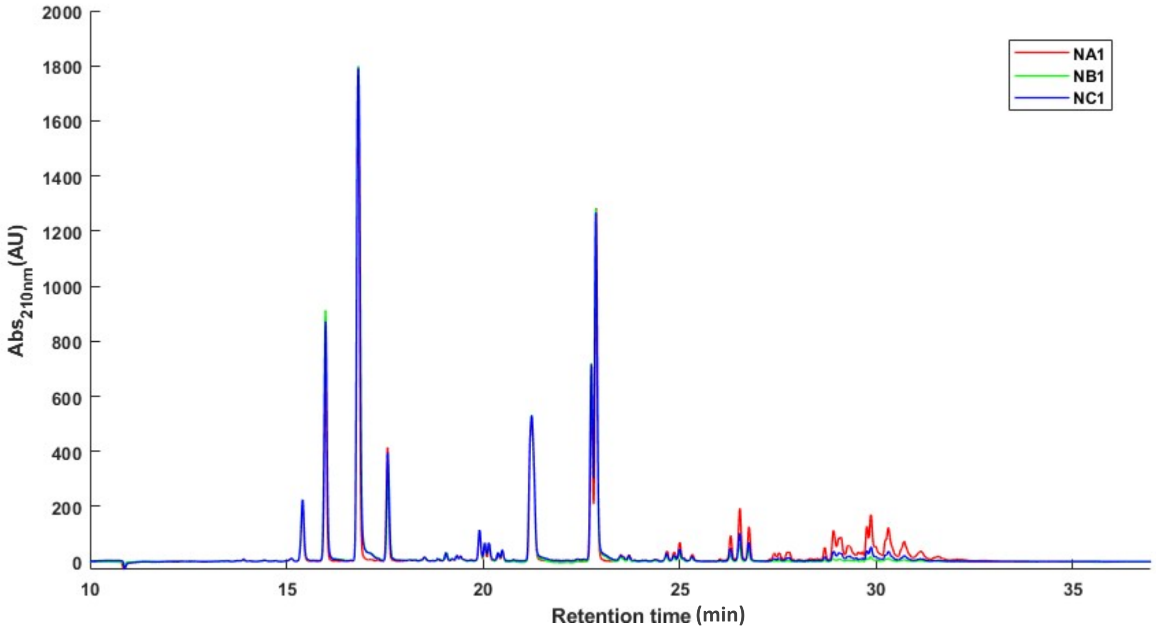

Supplementary Figure S185. HPLC overlay chromatogram of 3 repeats of Phenyllactic acid and Glycine. Phenyllactic acid (PLA) and Glycine were dried for 7 days in 85 °C, at a 5:1 molar ratio, in favor of PLA. Dry down products were then resuspended in an aqueous solution of 20% acetonitrile in water (v/v). The resulting products were analyzed by C18-HPLC.

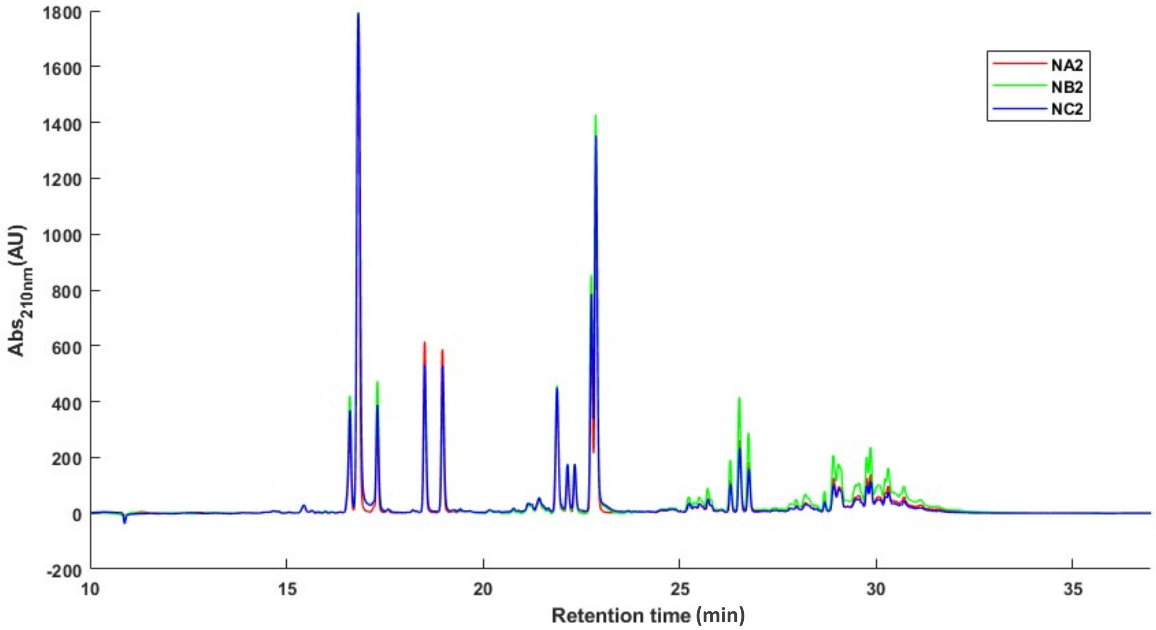

Supplementary Figure S186. HPLC overlay chromatogram of 3 repeats of Phenyllactic acid and L-alanine. Phenyllactic acid (PLA) and L-alanine were dried for 7 days in 85 °C, at a 5:1 molar ratio, in favor of PLA. Dry down products were then resuspended in an aqueous solution of 20% acetonitrile in water (v/v). The resulting products were analyzed by C18-HPLC.

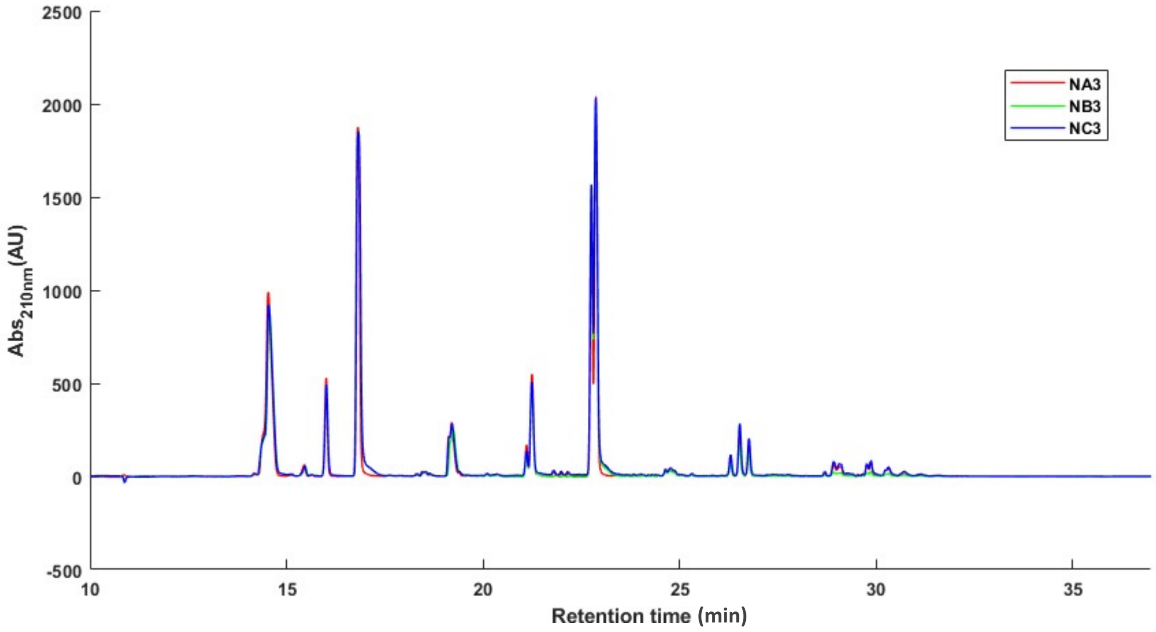

Supplementary Figure S187. HPLC overlay chromatogram of 3 repeats of Phenyllactic acid and β-alanine. Phenyllactic acid (PLA) and β-alanine were dried for 7 days in 85 °C, at a 5:1 molar ratio, in favor of PLA. Dry down products were then resuspended in an aqueous solution of 20% acetonitrile in water (v/v). The resulting products were analyzed by C18-HPLC.

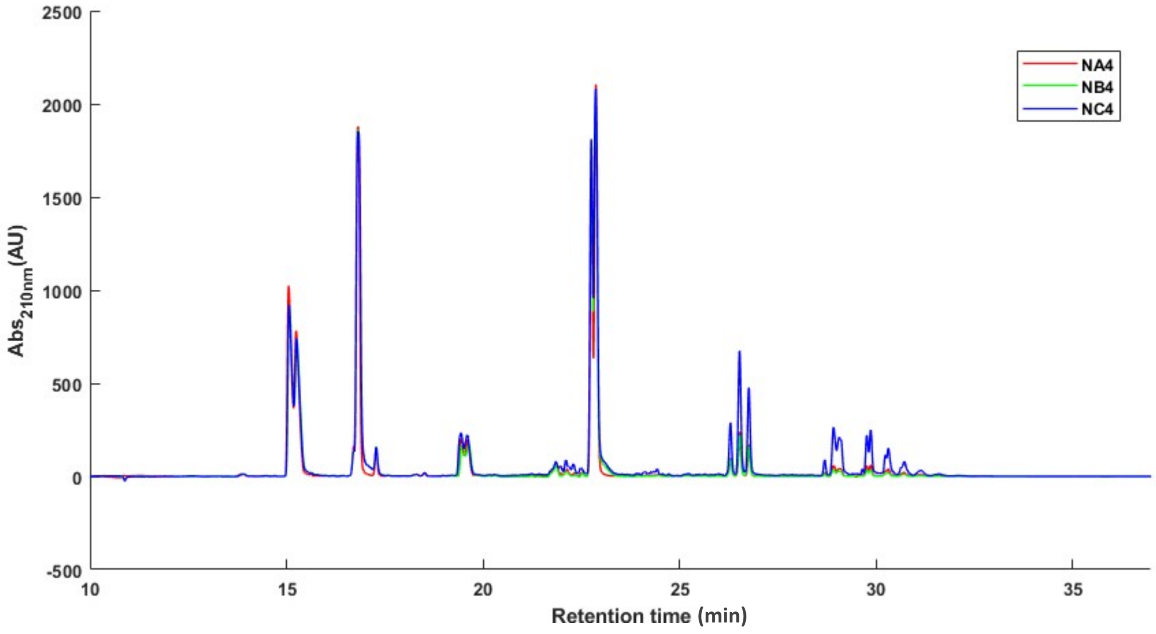

Supplementary Figure S188. HPLC overlay chromatogram of 3 repeats of Phenyllactic acid and β-aminobutyric acid. Phenyllactic acid (PLA) and β-aminobutyric acid were dried for 7 days in 85 °C, at a 5:1 molar ratio, in favor of PLA. Dry down products were then resuspended in an aqueous solution of 20% acetonitrile in water (v/v). The resulting products were analyzed by C18-HPLC.

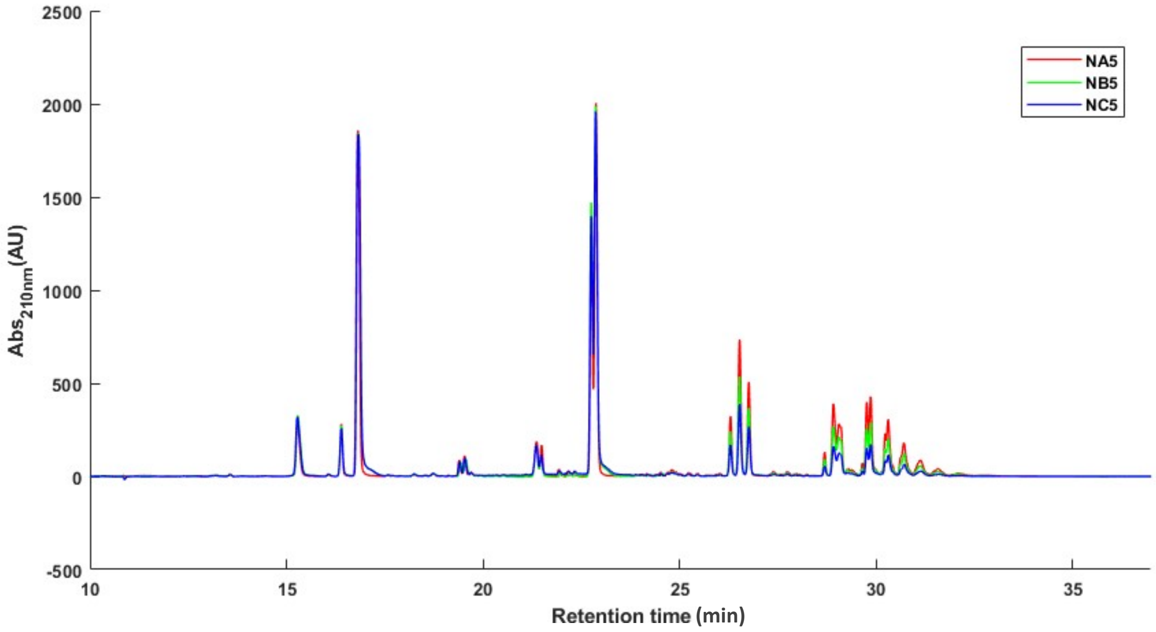

Supplementary Figure S189. HPLC overlay chromatogram of 3 repeats of Phenyllactic acid and γ-aminobutyric acid. Phenyllactic acid (PLA) and γ-aminobutyric acid were dried for 7 days in 85 °C, at a 5:1 molar ratio, in favor of PLA. Dry down products were then resuspended in an aqueous solution of 20% acetonitrile in water (v/v). The resulting products were analyzed by C18-HPLC.

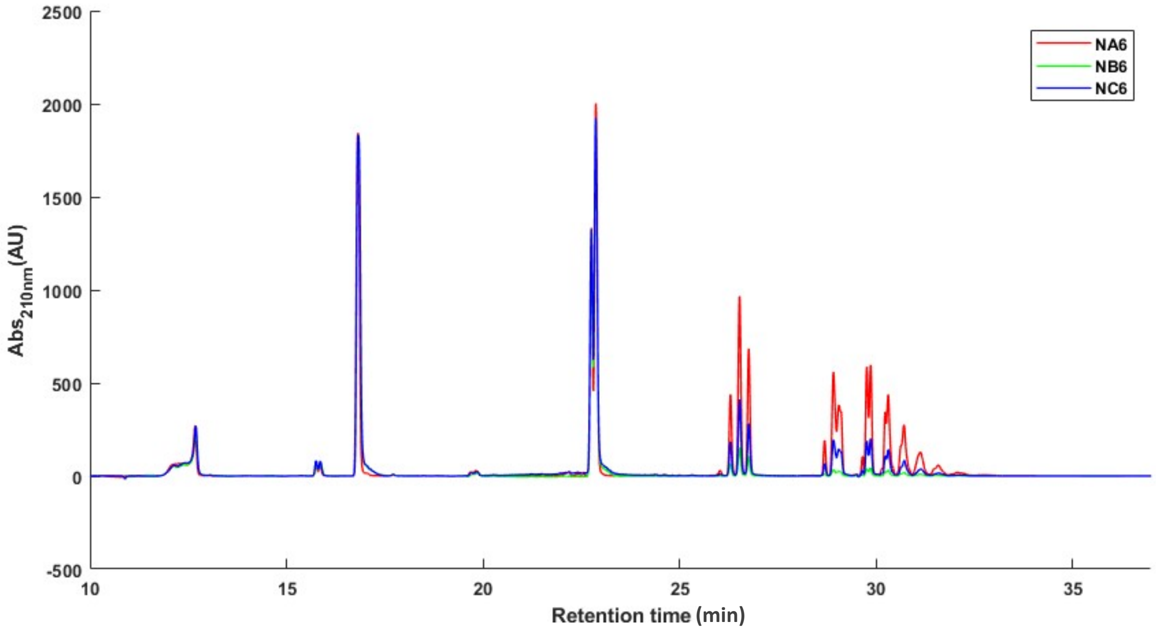

Supplementary Figure S190. HPLC overlay chromatogram of 3 repeats of Phenyllactic acid and γ-aminopentanoic acid. Phenyllactic acid (PLA) and γ-aminopentanoic acid were dried for 7 days in 85 °C, at a 5:1 molar ratio, in favor of PLA. Dry down products were then resuspended in an aqueous solution of 20% acetonitrile in water (v/v). The resulting products were analyzed by C18-HPLC.

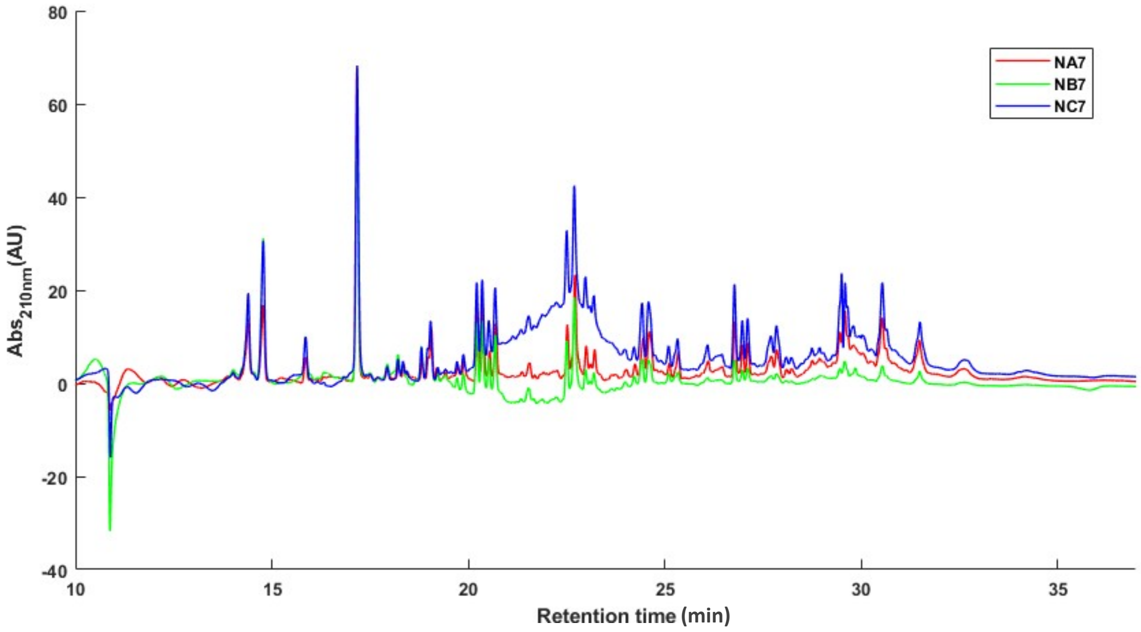

Supplementary Figure S191. HPLC overlay chromatogram of 3 repeats of Leucic acid and Glycine. Leucic acid (LA) and Glycine were dried for 7 days in 85 °C, at a 5:1 molar ratio, in favor of LA. Dry down products were then resuspended in an aqueous solution of 20% acetonitrile in water (v/v). The resulting products were analyzed by C18-HPLC.

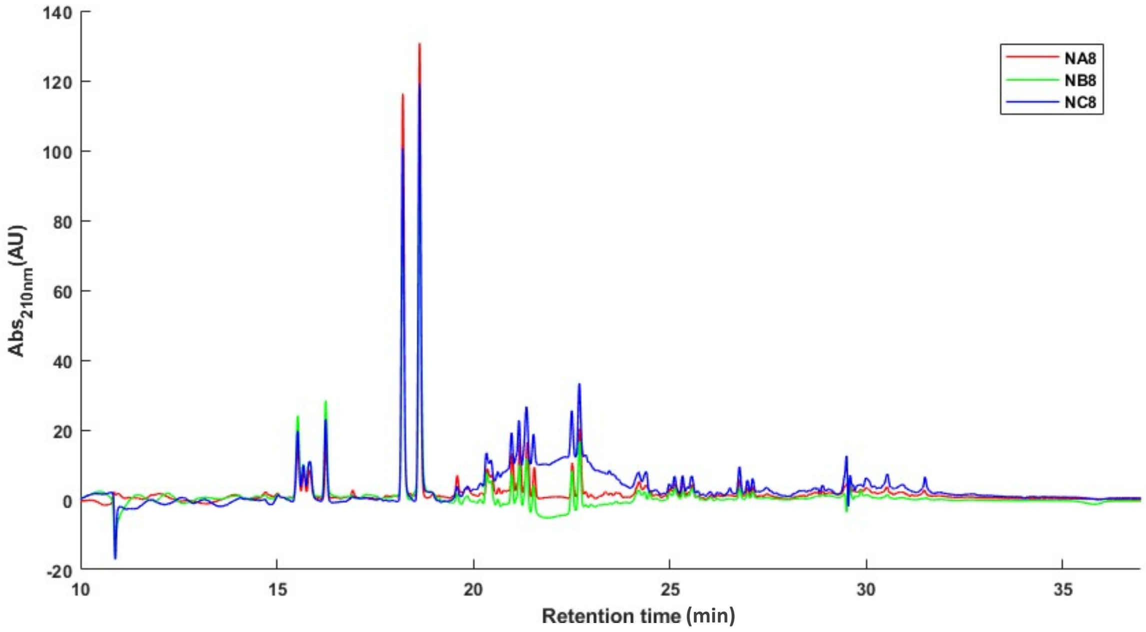

Supplementary Figure S192. HPLC overlay chromatogram of 3 repeats of Leucic acid and L-alanine. Leucic acid (LA) and L-alanine were dried for 7 days in 85 °C, at a 5:1 molar ratio, in favor of LA. Dry down products were then resuspended in an aqueous solution of 20% acetonitrile in water (v/v). The resulting products were analyzed by C18-HPLC.

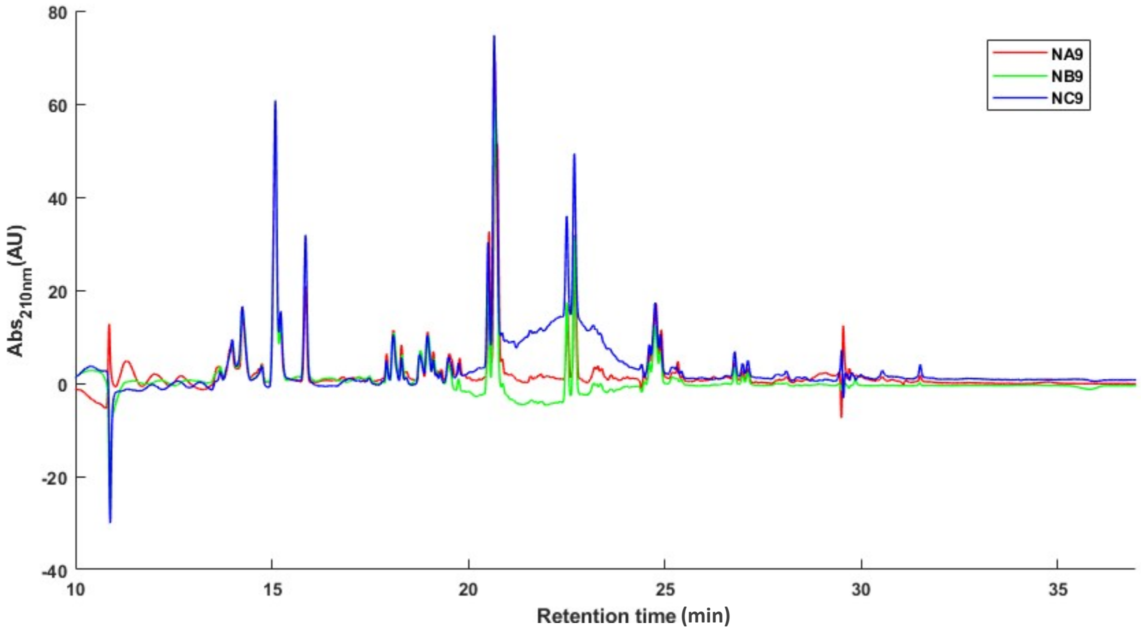

Supplementary Figure S193. HPLC overlay chromatogram of 3 repeats of Leucic acid and β-alanine. Leucic acid (LA) and β-alanine were dried for 7 days in 85 °C, at a 5:1 molar ratio, in favor of LA. Dry down products were then resuspended in an aqueous solution of 20% acetonitrile in water (v/v). The resulting products were analyzed by C18-HPLC.

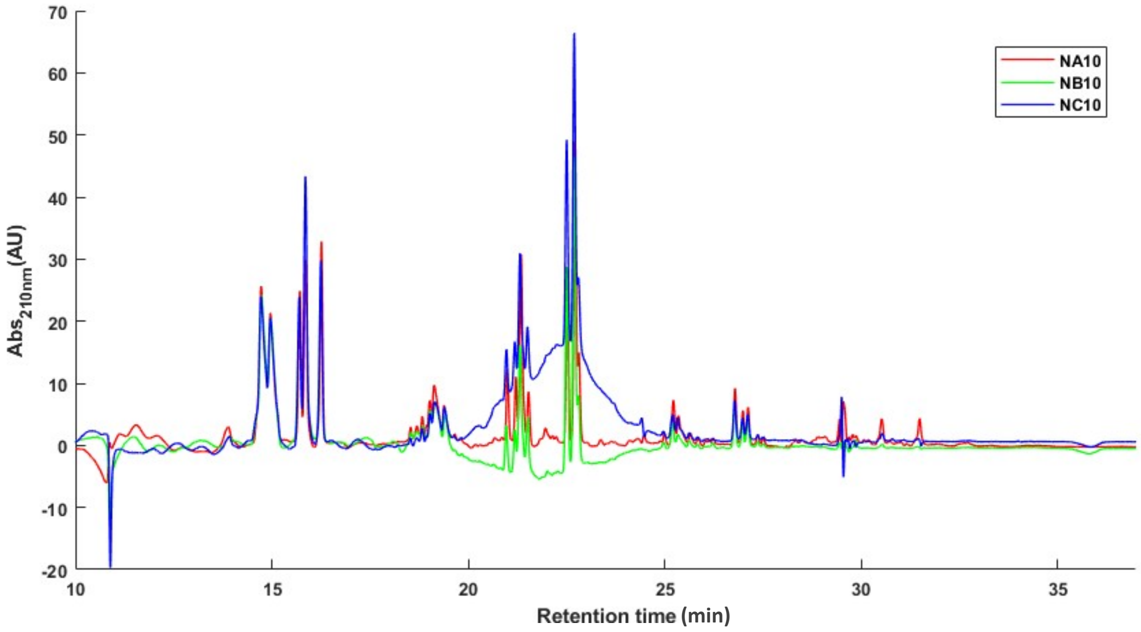

Supplementary Figure S194. HPLC overlay chromatogram of 3 repeats of Leucic acid and β-aminobutyric acid. Leucic acid (LA) and β-aminobutyric acid were dried for 7 days in 85 °C, at a 5:1 molar ratio, in favor of LA. Dry down products were then resuspended in an aqueous solution of 20% acetonitrile in water (v/v). The resulting products were analyzed by C18-HPLC.

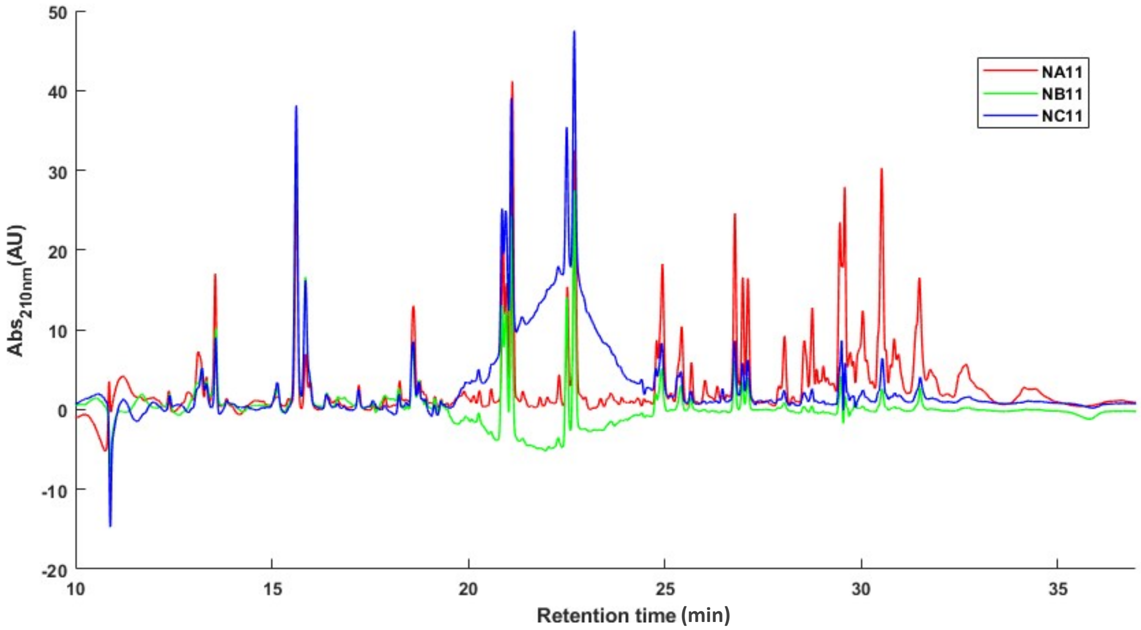

Supplementary Figure S195. HPLC overlay chromatogram of 3 repeats of Leucic acid and γ-aminobutyric acid. Leucic acid (LA) and γ-aminobutyric acid were dried for 7 days in 85 °C, at a 5:1 molar ratio, in favor of LA. Dry down products were then resuspended in an aqueous solution of 20% acetonitrile in water (v/v). The resulting products were analyzed by C18-HPLC.

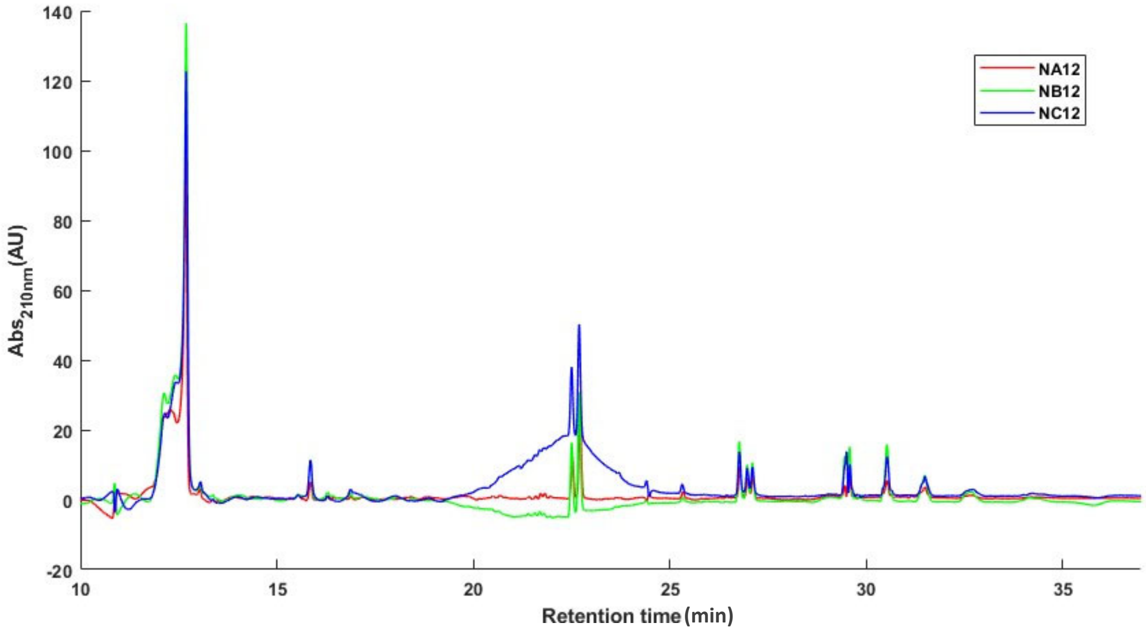

Supplementary Figure S196. HPLC overlay chromatogram of 3 repeats of Leucic acid and γ-aminopentanoic acid. Leucic acid (LA) and γ-aminopentanoic acid were dried for 7 days in 85 °C, at a 5:1 molar ratio, in favor of LA. Dry down products were then resuspended in an aqueous solution of 20% acetonitrile in water (v/v). The resulting products were analyzed by C18-HPLC.

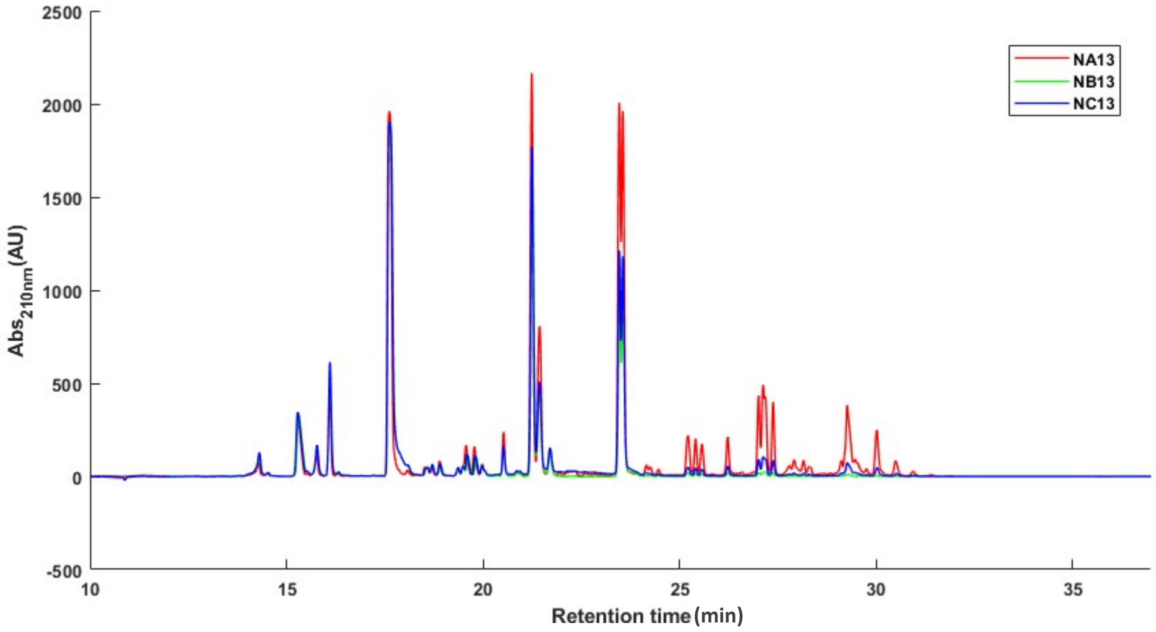

Supplementary Figure S197. HPLC overlay chromatogram of 3 repeats of β-Phenyllactic acid and Glycine. β-Phenyllactic acid (β-PLA) and Glycine acid were dried for 7 days in 85 °C, at a 5:1 molar ratio, in favor of β-PLA. Dry down products were then resuspended in an aqueous solution of 20% acetonitrile in water (v/v). The resulting products were analyzed by C18-HPLC.

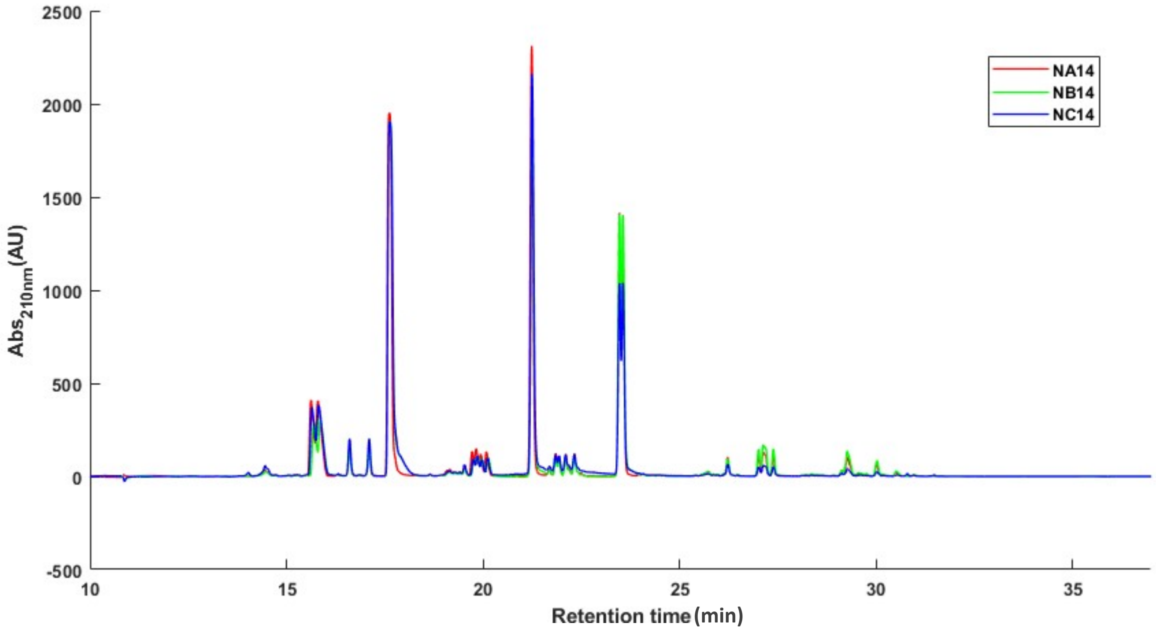

Supplementary Figure S198. HPLC overlay chromatogram of 3 repeats of β-Phenyllactic acid and L-alanine. β-Phenyllactic acid (β-PLA) and L-alanine were dried for 7 days in 85 °C, at a 5:1 molar ratio, in favor of β-PLA. Dry down products were then resuspended in an aqueous solution of 20% acetonitrile in water (v/v). The resulting products were analyzed by C18-HPLC.

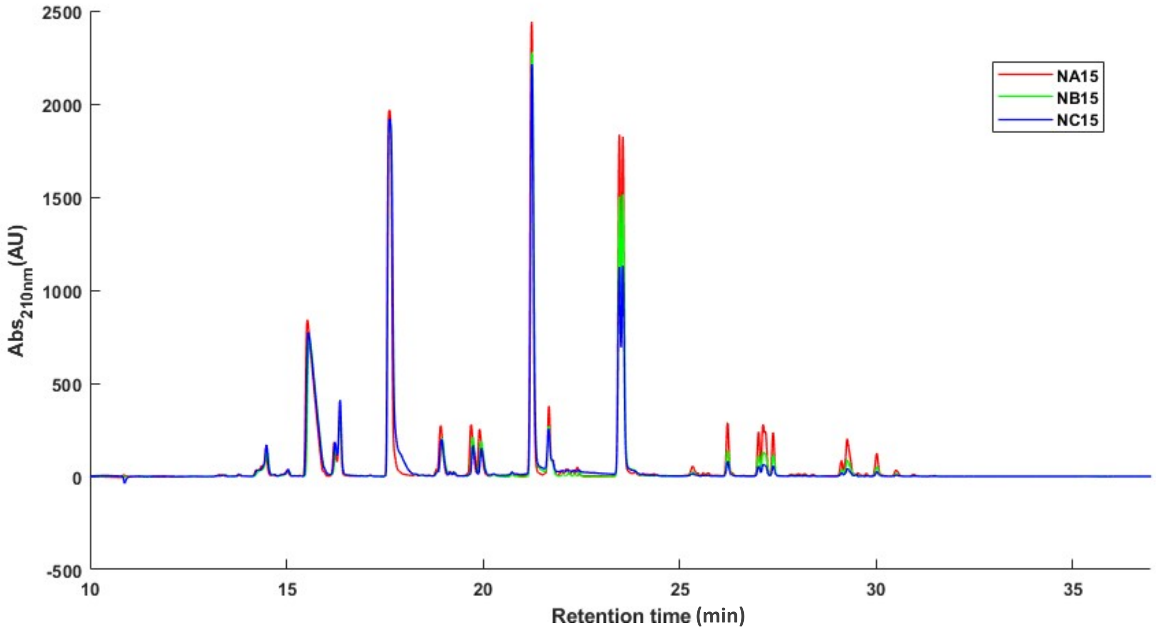

Supplementary Figure S199. HPLC overlay chromatogram of 3 repeats of β-Phenyllactic acid and β-alanine. β-Phenyllactic acid (β-PLA) and β-alanine were dried for 7 days in 85 °C, at a 5:1 molar ratio, in favor of β-PLA. Dry down products were then resuspended in an aqueous solution of 20% acetonitrile in water (v/v). The resulting products were analyzed by C18-HPLC.

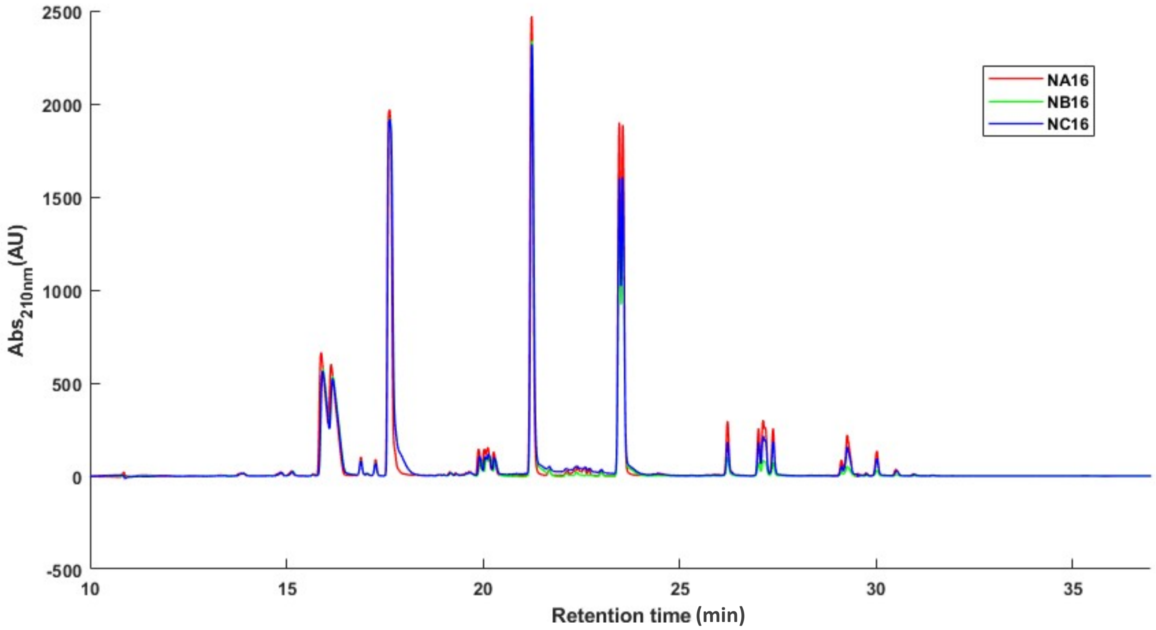

Supplementary Figure S200. HPLC overlay chromatogram of 3 repeats of β-Phenyllactic acid and β-aminobutyric acid. β-Phenyllactic acid (β-PLA) and β-aminobutyric acid were dried for 7 days in 85 °C, at a 5:1 molar ratio, in favor of β-PLA. Dry down products were then resuspended in an aqueous solution of 20% acetonitrile in water (v/v). The resulting products were analyzed by C18-HPLC.

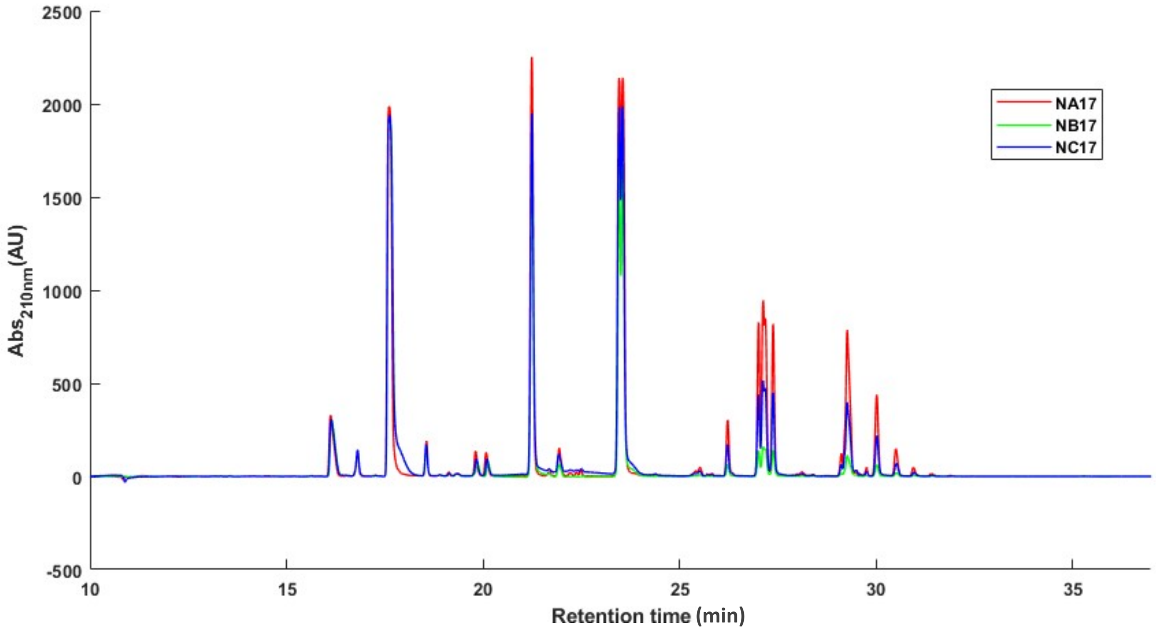

Supplementary Figure S201. HPLC overlay chromatogram of 3 repeats of β-Phenyllactic acid and γ-aminobutyric acid. β-Phenyllactic acid (β-PLA) and γ-aminobutyric acid were dried for 7 days in 85 °C, at a 5:1 molar ratio, in favor of β-PLA. Dry down products were then resuspended in an aqueous solution of 20% acetonitrile in water (v/v). The resulting products were analyzed by C18-HPLC.

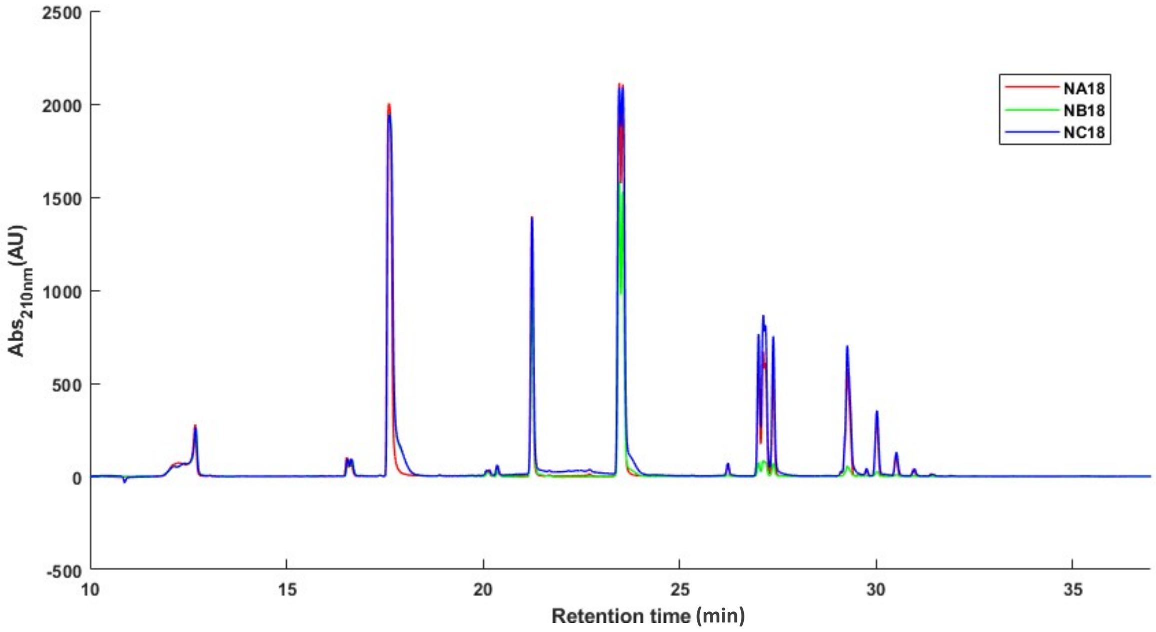

Supplementary Figure S202. HPLC overlay chromatogram of 3 repeats of β-Phenyllactic acid and γ-aminopentanoic acid. β-Phenyllactic acid (β-PLA) and γ-aminopentanoic acid were dried for 7 days in 85 °C, at a 5:1 molar ratio, in favor of β-PLA. Dry down products were then resuspended in an aqueous solution of 20% acetonitrile in water (v/v). The resulting products were analyzed by C18-HPLC.

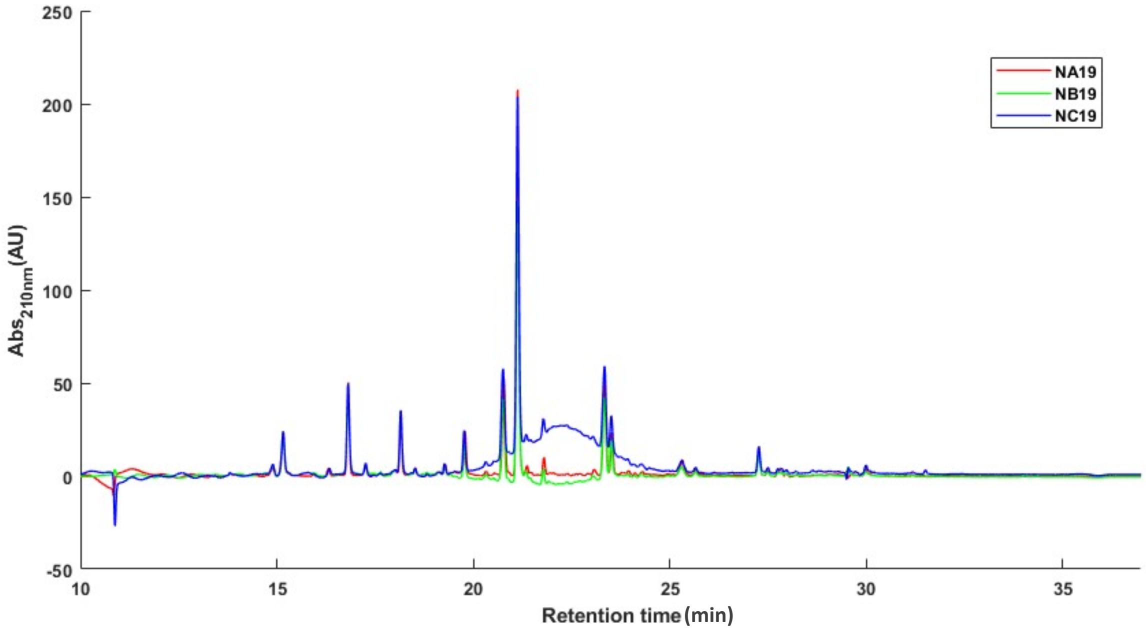

Supplementary Figure S203. HPLC overlay chromatogram of 3 repeats of β-Leucic acid and Glycine. β-Leucic acid (β-LA) and Glycine were dried for 7 days in 85 °C, at a 5:1 molar ratio, in favor of β-LA. Dry down products were then resuspended in an aqueous solution of 20% acetonitrile in water (v/v). The resulting products were analyzed by C18-HPLC.

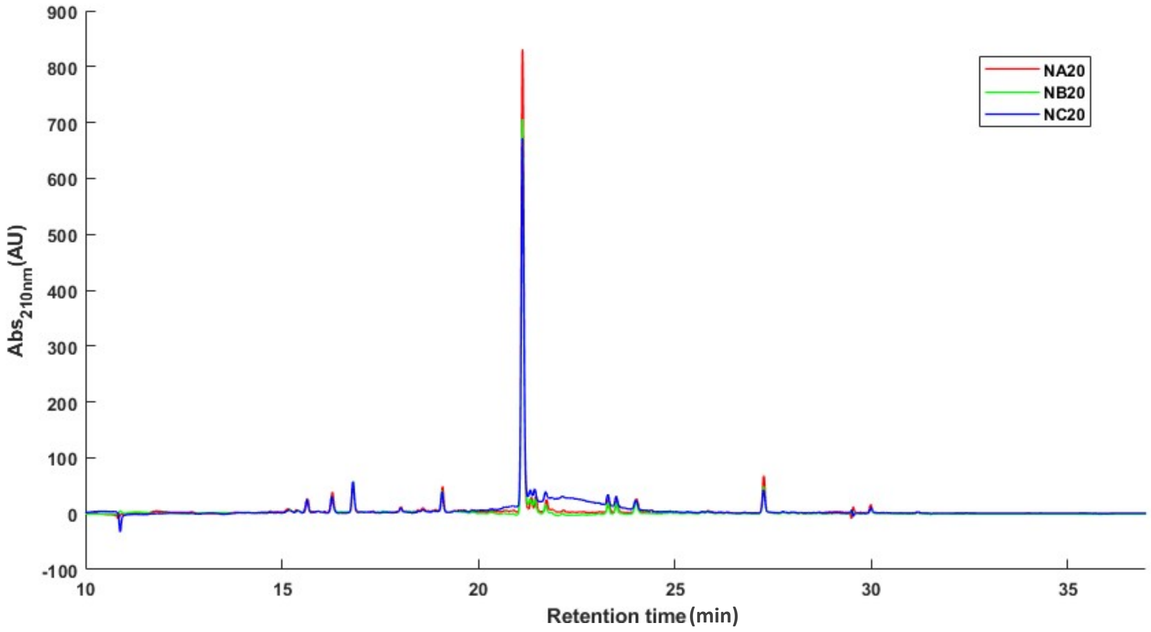

Supplementary Figure S204. HPLC overlay chromatogram of 3 repeats of β-Leucic acid and L-alanine. β-Leucic acid (β-LA) and L-alanine were dried for 7 days in 85 °C, at a 5:1 molar ratio, in favor of β-LA. Dry down products were then resuspended in an aqueous solution of 20% acetonitrile in water (v/v). The resulting products were analyzed by C18-HPLC.

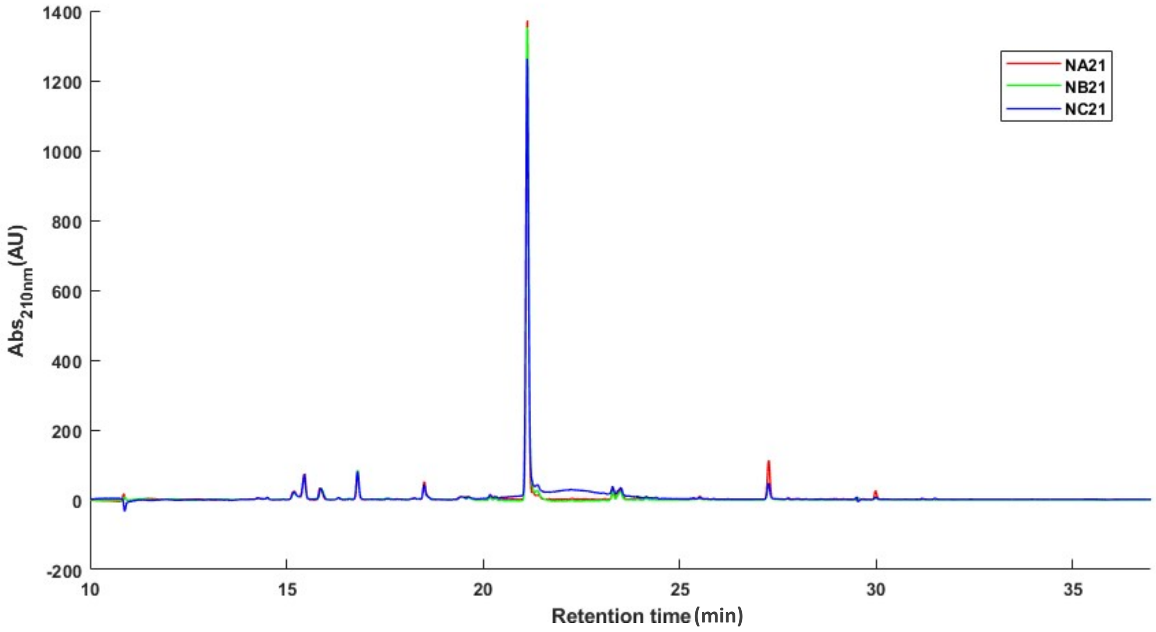

Supplementary Figure S205. HPLC overlay chromatogram of 3 repeats of β-Leucic acid and β-alanine. β-Leucic acid (β-LA) and β-alanine were dried for 7 days in 85 °C, at a 5:1 molar ratio, in favor of β-LA. Dry down products were then resuspended in an aqueous solution of 20% acetonitrile in water (v/v). The resulting products were analyzed by C18-HPLC.

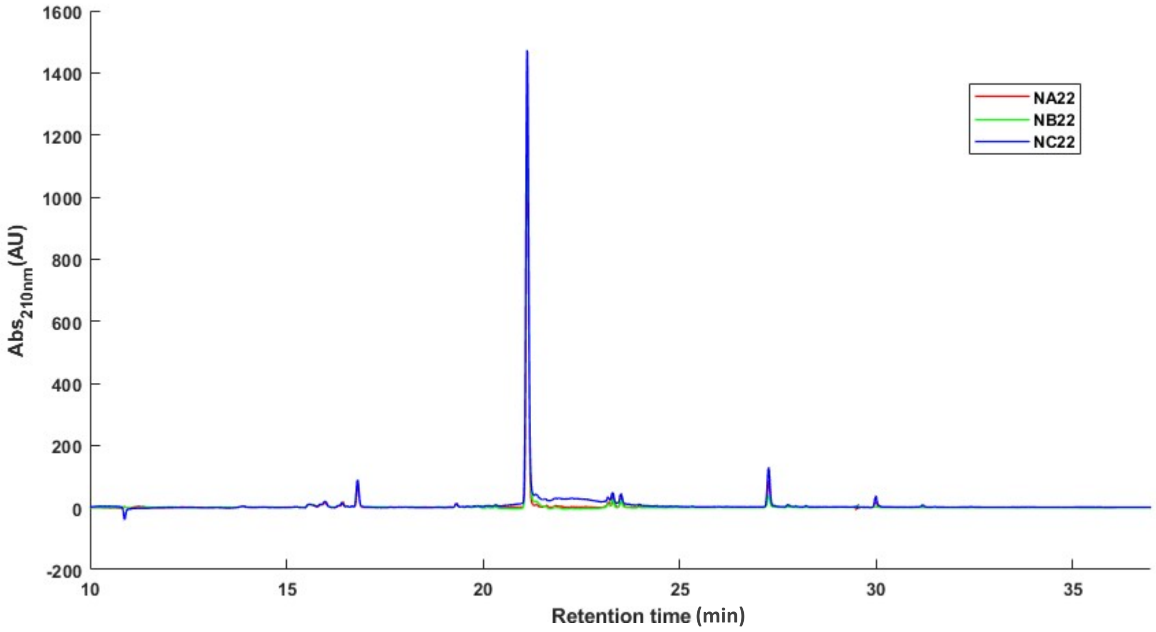

Supplementary Figure S206. HPLC overlay chromatogram of 3 repeats of β-Leucic acid and β-aminobutyric acid. β-Leucic acid (β-LA) and β-aminobutyric acid were dried for 7 days in 85 °C, at a 5:1 molar ratio, in favor of β-LA. Dry down products were then resuspended in an aqueous solution of 20% acetonitrile in water (v/v). The resulting products were analyzed by C18-HPLC.

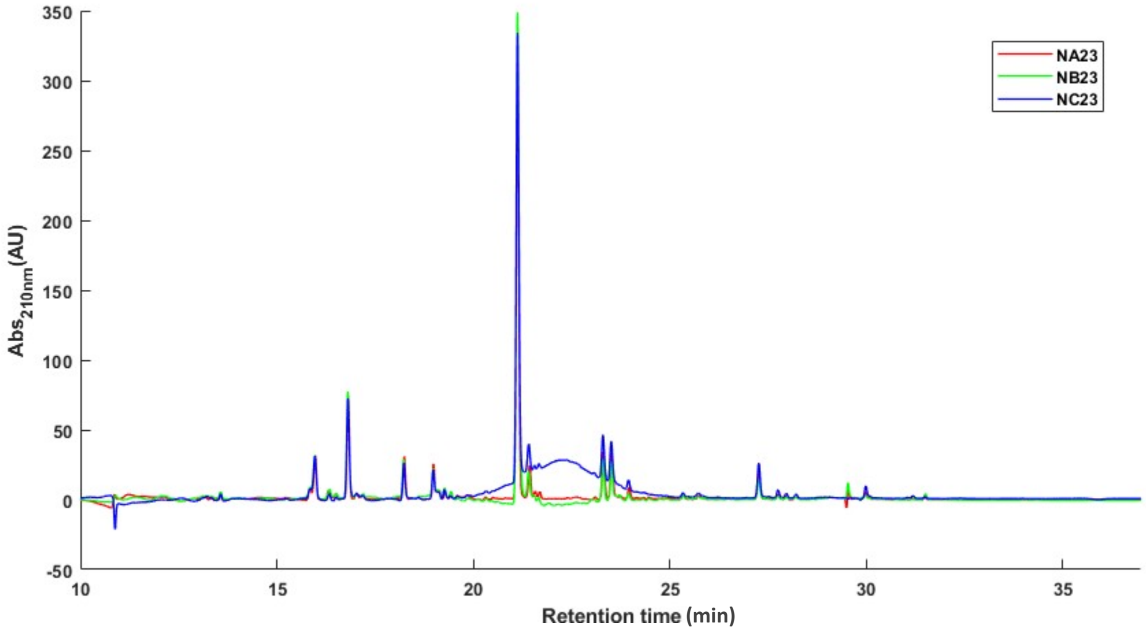

Supplementary Figure S207. HPLC overlay chromatogram of 3 repeats of β-Leucic acid and γ-aminobutyric acid. β-Leucic acid (β-LA) and γ-aminobutyric acid were dried for 7 days in 85 °C, at a 5:1 molar ratio, in favor of β-LA. Dry down products were then resuspended in an aqueous solution of 20% acetonitrile in water (v/v). The resulting products were analyzed by C18-HPLC.

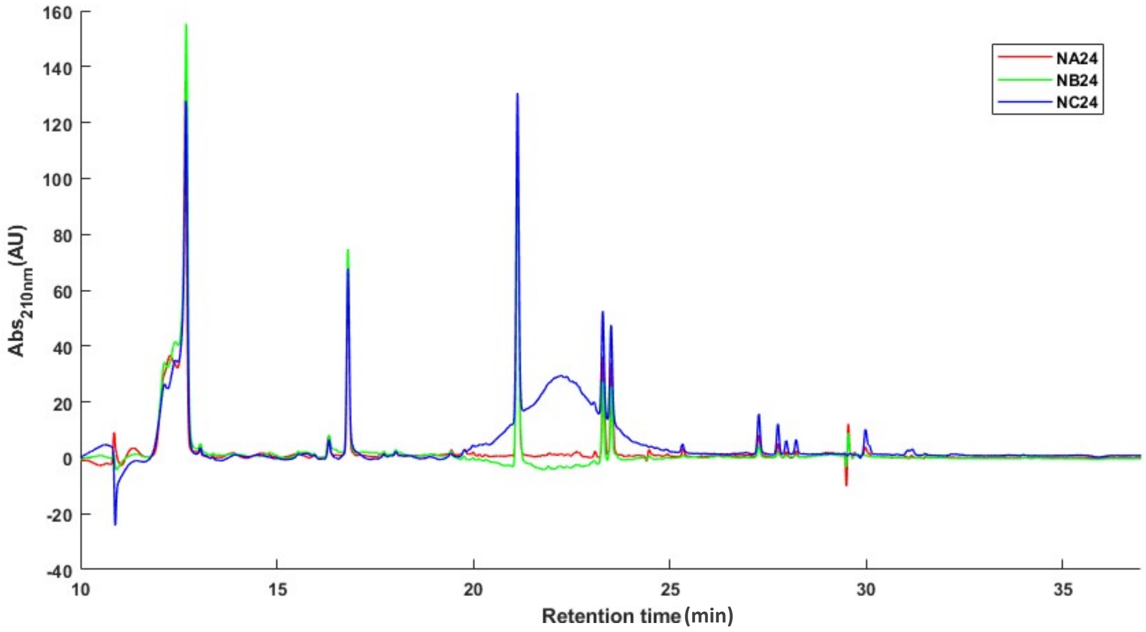

Supplementary Figure S208. HPLC overlay chromatogram of 3 repeats of β-Leucic acid and γ-aminopentanoic acid. β-Leucic acid (β-LA) and γ-aminopentanoic acid were dried for 7 days in 85 °C, at a 5:1 molar ratio, in favor of β-LA. Dry down products were then resuspended in an aqueous solution of 20% acetonitrile in water (v/v). The resulting products were analyzed by C18-HPLC.

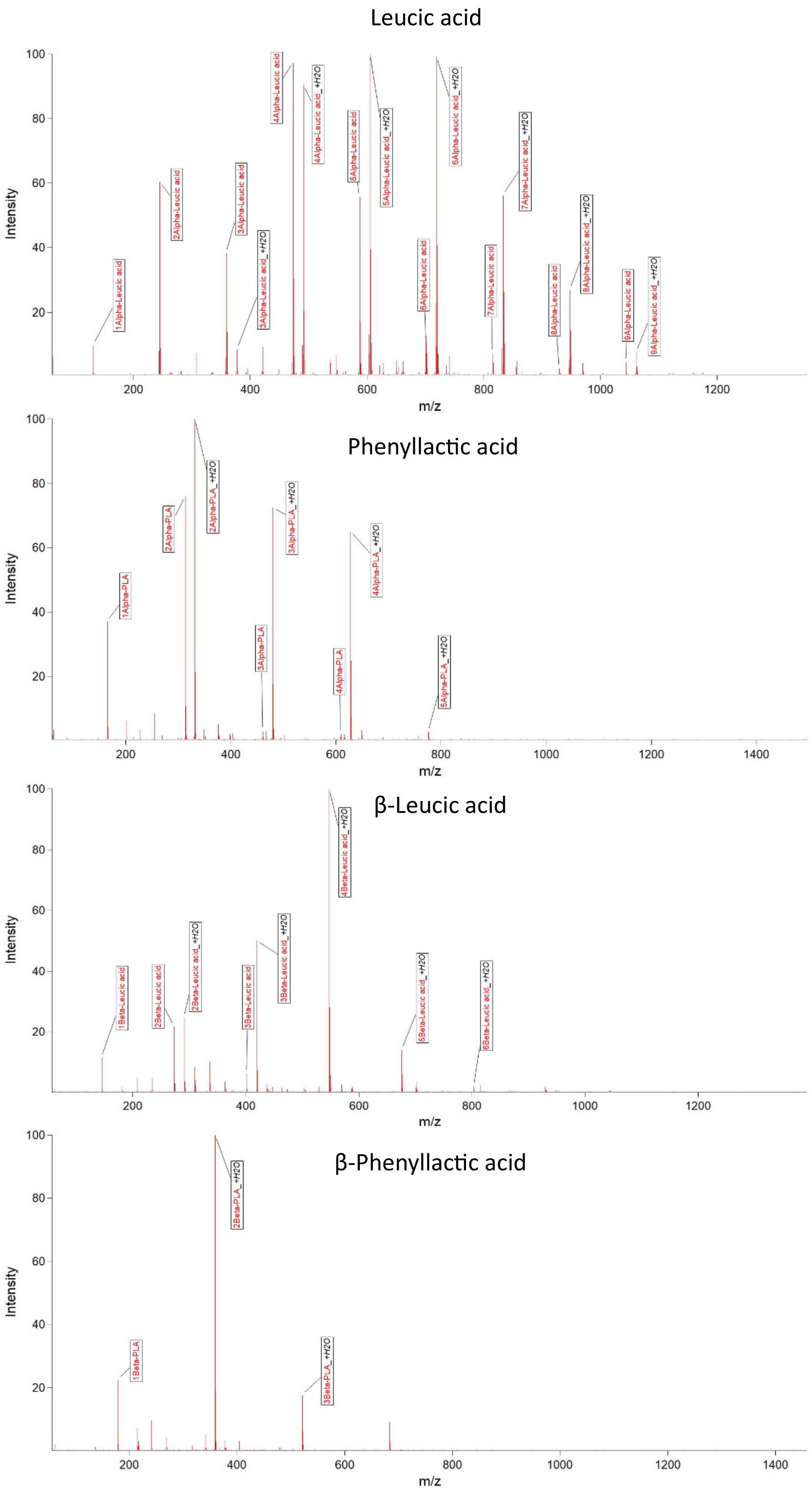

Supplementary Figure S209. ESI-MS spectra of dried down Hydroxy acid control samples – Phenyllactic acid, Leucic acid, β-Phenyllactic acid, β-Leucic acid. Hydroxy acid control dry downs at 100 umole each. Dry down products were then resuspended in an aqueous solution of 20% acetonitrile in water (v/v). The resulting products were analyzed by negative-mode ESI-MS.

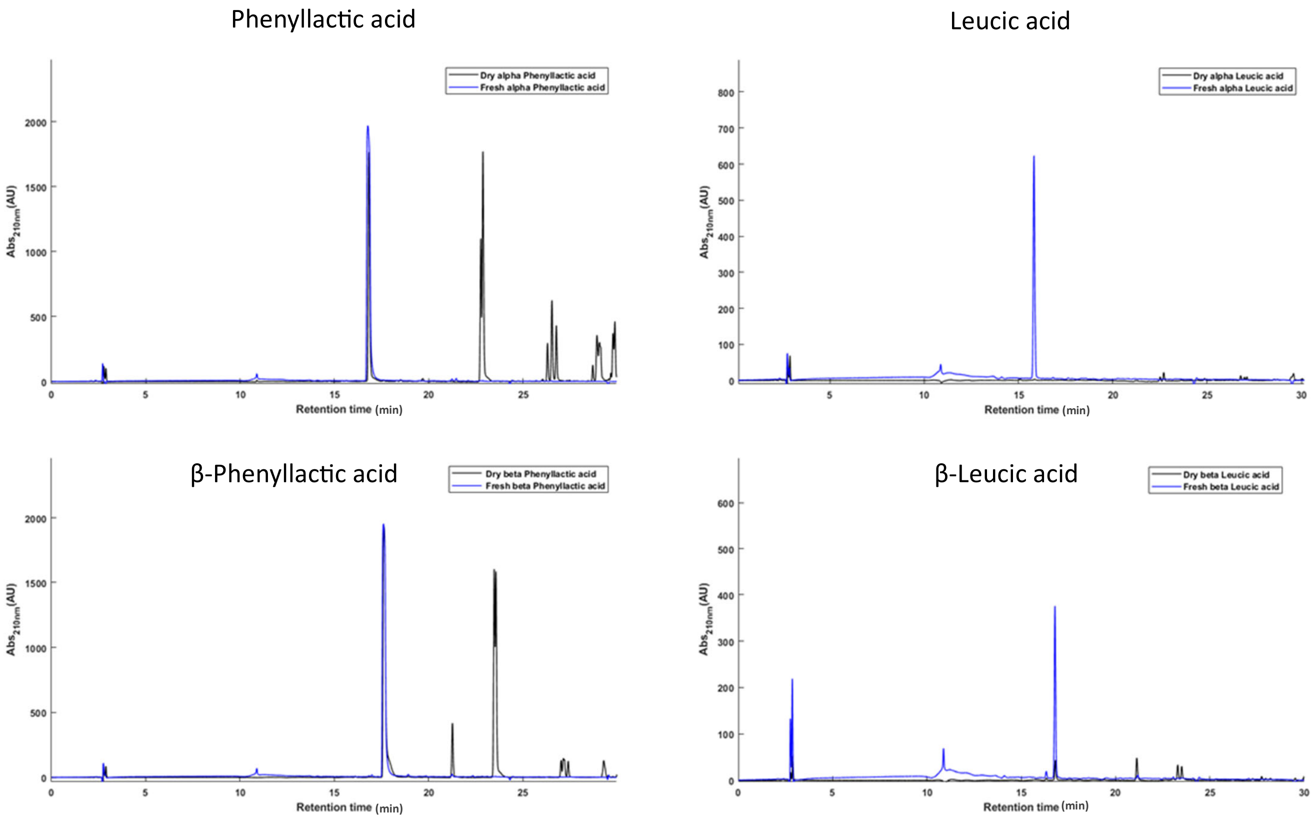

Supplementary Figure S210. HPLC chromatograms of dried down Hydroxy acid control samples – Phenyllactic acid, Leucic acid, β-Phenyllactic acid, β-Leucic acid. Hydroxy acid control dry downs at 100 umole each. Dry down products were then resuspended in an aqueous solution of 20% acetonitrile in water (v/v). The resulting products were then corrected to 50% acetonitrile for analysis by HPLC .

Phenyllactic acid

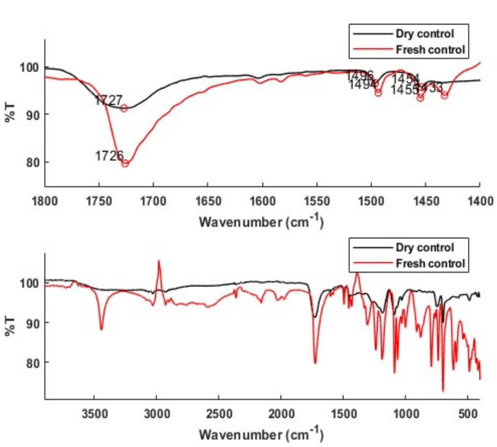

Leucic acid

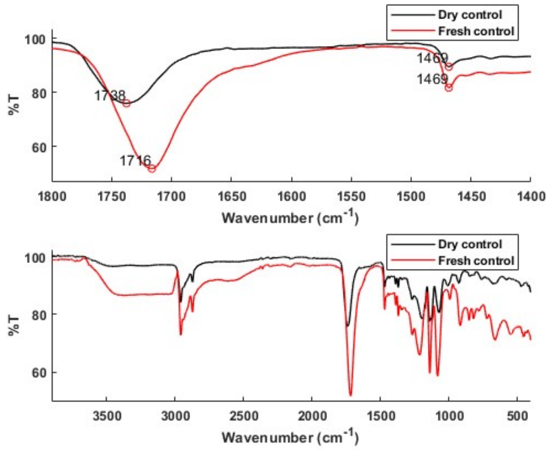

β-Phenyllactic acid

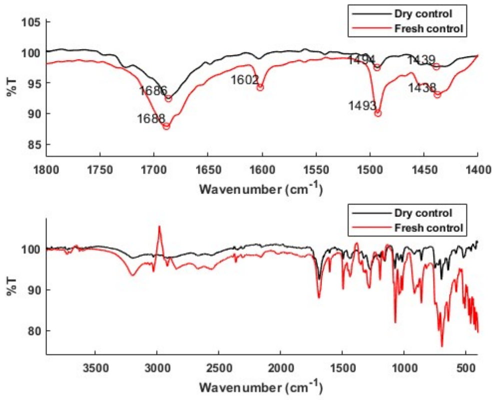

β-Leucic acid

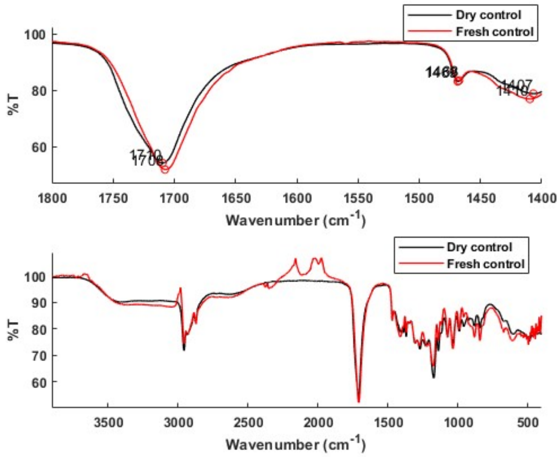

Supplementary Figure S211. FTIR overlay spectrum of dry down and fresh control samples of Hydroxy acids – Phenyllactic acid, Leucic acid, β-Phenyllactic acid, β-Leucic acid. Hydroxy acid control dry downs at 100 umole each. Dry down products were then resuspended in an aqueous solution of 20% acetonitrile in water (v/v). The resulting products were analyzed by FTIR

AA / pair	Volume(µl)	Solvent	HA/AA ratio (µmole/µmole)	Sample internal code
Phenyllactic acid+glycine	500	80% H2O 20%ACN	100/20	NA1
Phenyllactic acid +L-alanine	500	80% H2O 20%ACN	100/20	NA2
Phenyllactic acid +beta alanine	500	80% H2O 20%ACN	100/20	NA3
Phenyllactic acid +beta ABA	500	80% H2O 20%ACN	100/20	NA4
Phenyllactic acid +gamma ABA	500	80% H2O 20%ACN	100/20	NA5
Phenyllactic acid + gamma APA	500	80% H2O 20%ACN	100/20	NA6
Leucic acid+glycine	500	80% H2O 20%ACN	100/20	NA7
Leucic acid+L-alanine	500	80% H2O 20%ACN	100/20	NA8
Leucic acid+beta alanine	500	80% H2O 20%ACN	100/20	NA9
Leucic acid+beta ABA	500	80% H2O 20%ACN	100/20	NA10
Leucic acid+gamma ABA	500	80% H2O 20%ACN	100/20	NA11
Leucic acid+ gamma APA	500	80% H2O 20%ACN	100/20	NA12
beta- Phenyllactic acid +glycine	500	80% H2O 20%ACN	100/20	NA13
beta- Phenyllactic acid +L-alanine	500	80% H2O 20%ACN	100/20	NA14
beta- Phenyllactic acid +beta alanine	500	80% H2O 20%ACN	100/20	NA15
beta- Phenyllactic acid +beta ABA	500	80% H2O 20%ACN	100/20	NA16
beta- Phenyllactic acid +gamma ABA	500	80% H2O 20%ACN	100/20	NA17
beta- Phenyllactic acid + gamma APA	500	80% H2O 20%ACN	100/20	NA18
beta-Leucic acid+glycine	500	80% H2O 20%ACN	100/20	NA19
beta-Leucic acid+L-alanine	500	80% H2O 20%ACN	100/20	NA20
beta-Leucic acid+beta alanine	500	80% H2O 20%ACN	100/20	NA21
beta-Leucic acid+beta ABA	500	80% H2O 20%ACN	100/20	NA22
beta-Leucic acid+gamma ABA	500	80% H2O 20%ACN	100/20	NA23
beta-Leucic acid+ gamma APA	500	80% H2O 20%ACN	100/20	NA24

Supplementary Table S2. List of samples of the second matrix. Batch NA sample table coded from NA1-NA24, each sample code corrseponds to a mixture of amino acid and hydroxy acid. All samples were prepered at a ratio of 5:1 in favor of the hydroxy acid and resuspended with a solution of 80% deionized water and 20% aectonitrile (ACN), to a 40mM concentration of amino acid (referring to initial amount prior to drying).

NA1→3

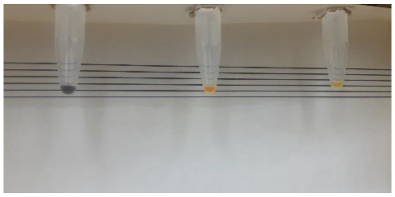
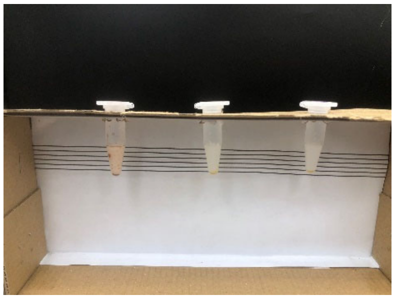

NA4→6

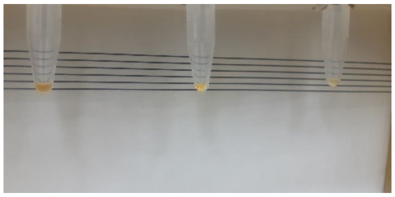
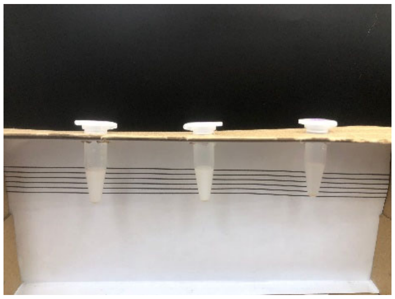

NA7→9

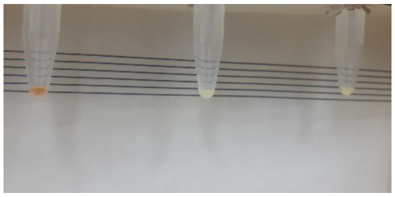
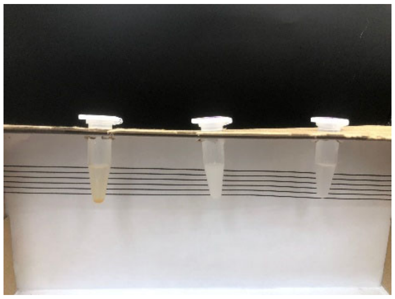

NA10→12

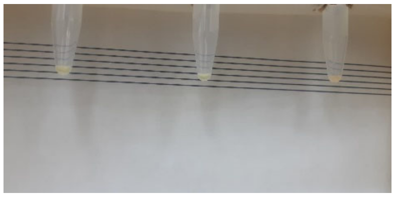
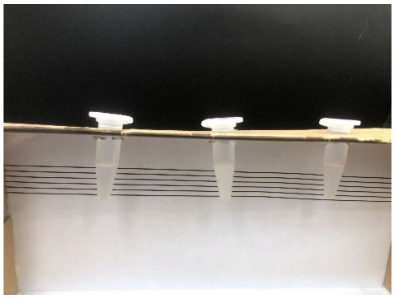

NA13→15

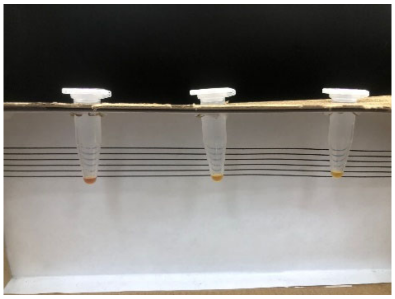
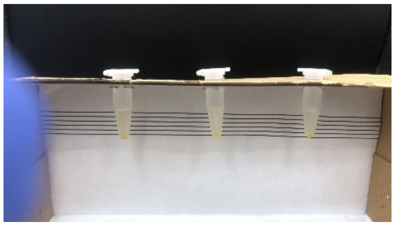

NA16→18

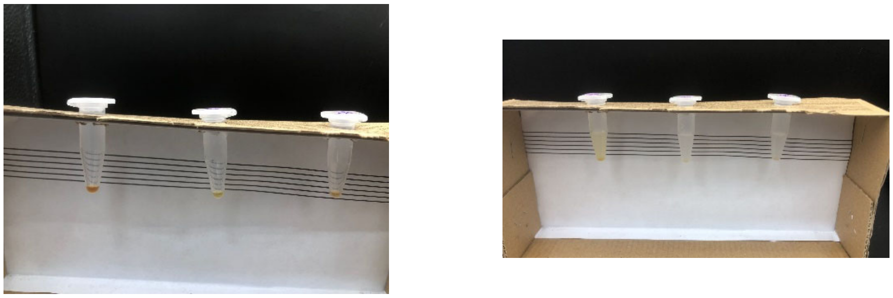

NA19→21

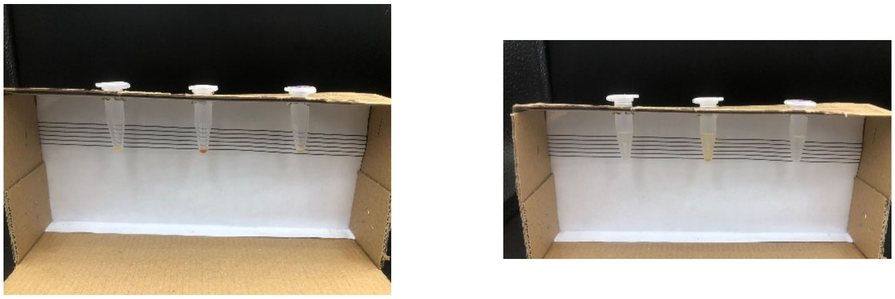

NA22→24

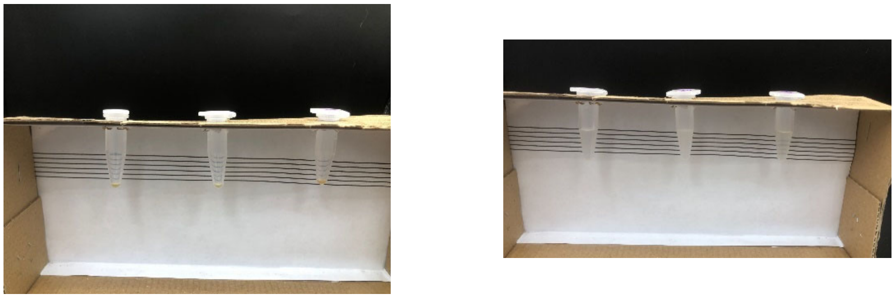

Supplementary Figure S212. – Visual appearance. Batch NA sample images after dry down for 7 days in 85 °C, before and after suspention in a solution of 80% deionized water and 20% acetonitrile (ACN) to final concentration of 40mM of amino acid (referring to initial amount prior to drying). Samples were coded NA1-NA24 acorrding to Table S2,

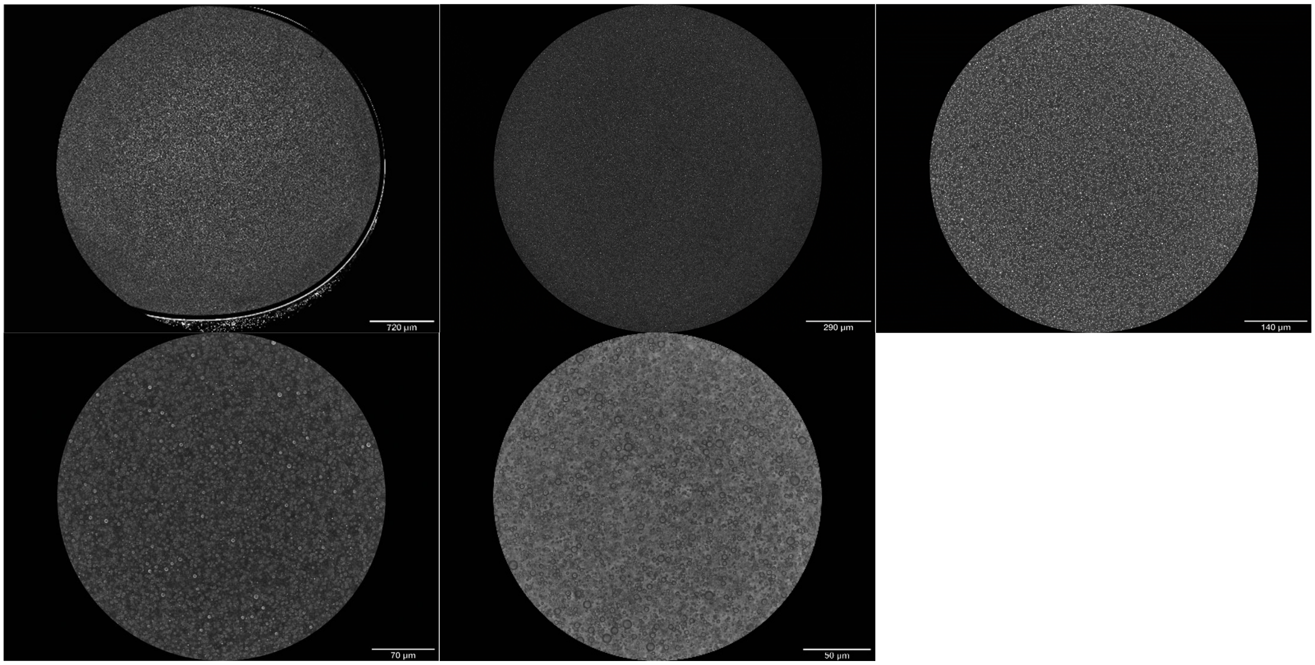

Supplementary Figure S213. Brightfield microscopy imaging of depsipeptides resulting from dry-down reactions of Phenyllactic acid and Glycine. Microscopy of sample NA1- Phenyllactic acid and Glycine, resuspended in 80% deionized water and 20% acetonitrile (v/v), at five different magnifications - x4, x10, x20, x40 and x60.

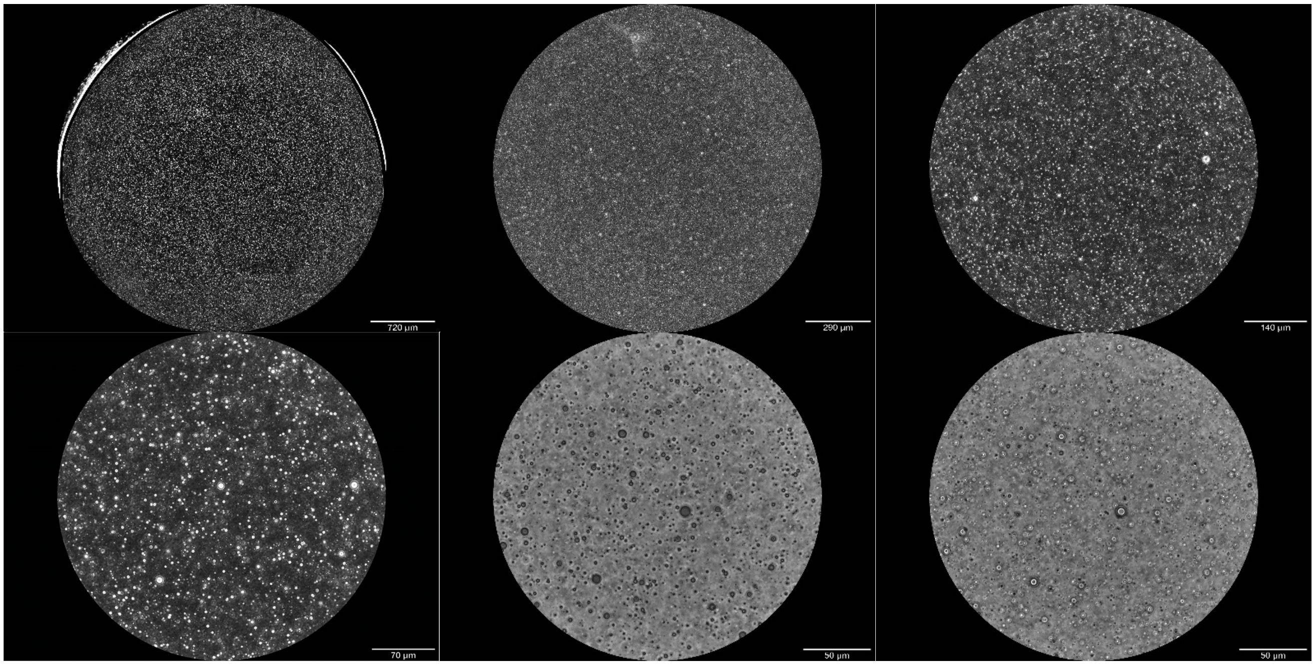

Supplementary Figure S214. Brightfield microscopy imaging of depsipeptides resulting from dry-down reactions of Phenyllactic acid and L-alanine. Microscopy of sample NA2- Phenyllactic acid and L-alanine, resuspended in 80% deionized water and 20% acetonitrile (v/v), at five different magnifications - x4, x10, x20, x40 and x60.

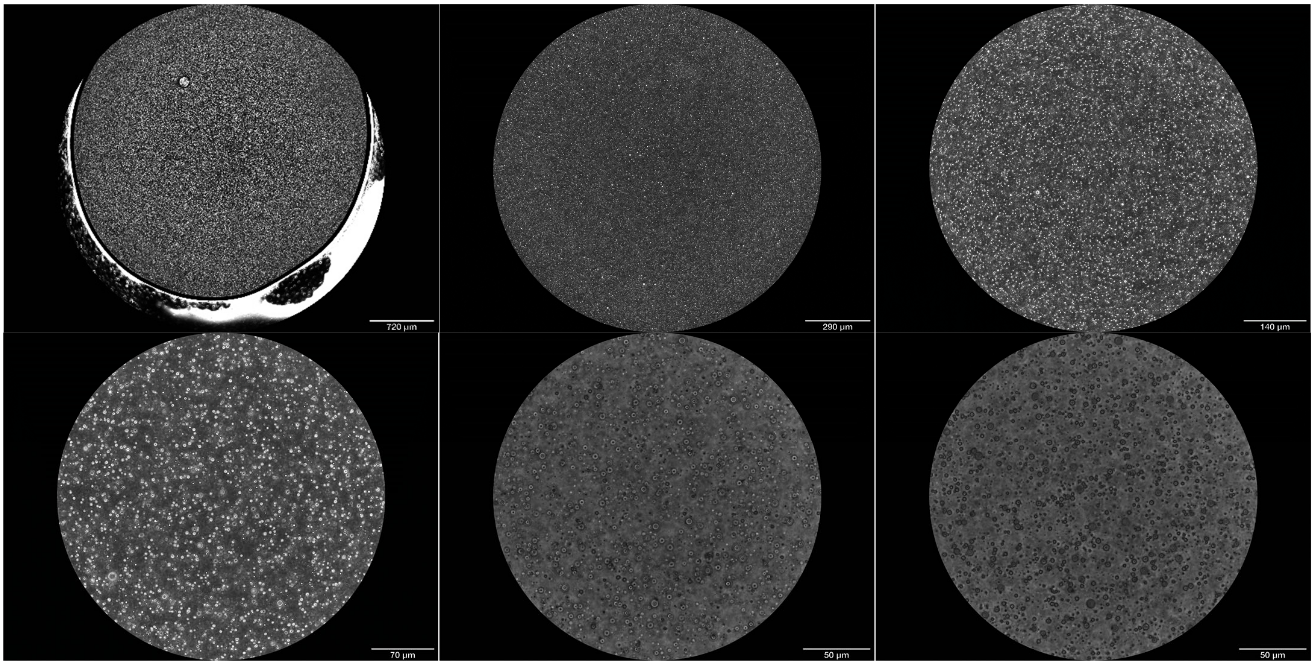

Supplementary Figure S215. Brightfield microscopy imaging of depsipeptides resulting from dry-down reactions of Phenyllactic acid and β-alanine. Microscopy of sample NA3- Phenyllactic acid and β-alanine, resuspended in 80% deionized water and 20% acetonitrile (v/v), at five different magnifications - x4, x10, x20, x40 and x60.

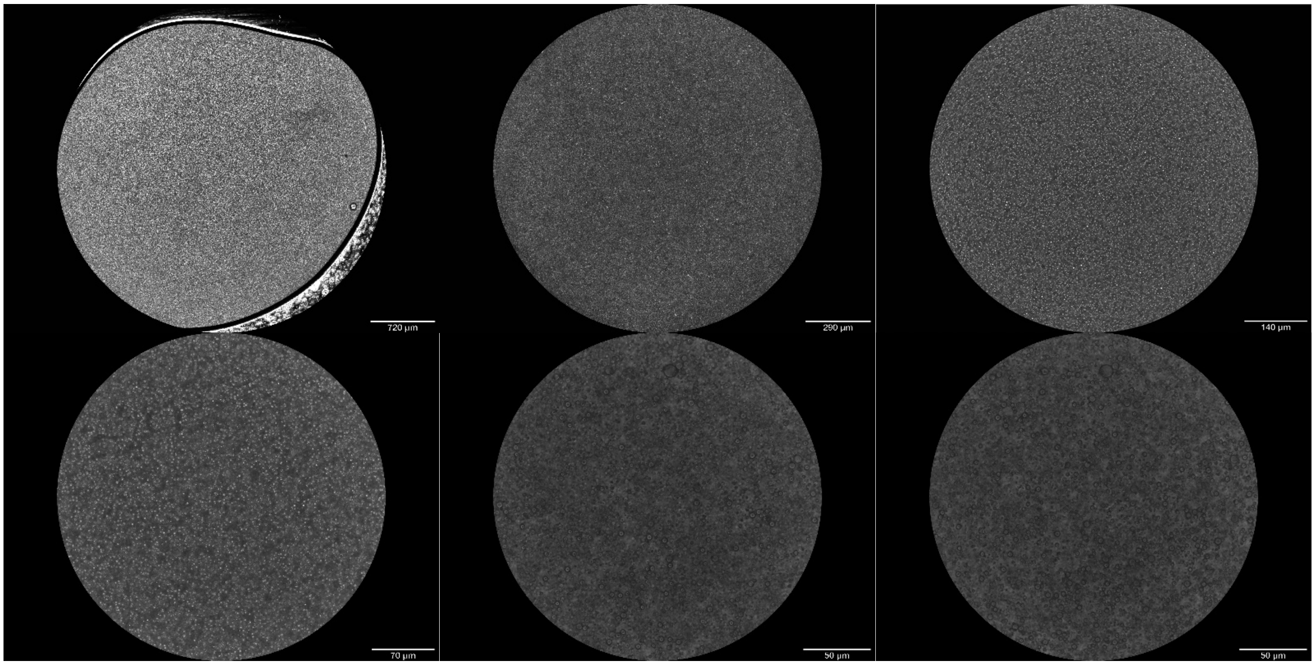

Supplementary Figure S216. Brightfield microscopy imaging of depsipeptides resulting from dry-down reactions of Phenyllactic acid and β-aminobutyric acid. Microscopy of sample NA4- Phenyllactic acid and β-aminobutyric acid, resuspended in 80% deionized water and 20% acetonitrile (v/v), at five different magnifications - x4, x10, x20, x40 and x60.

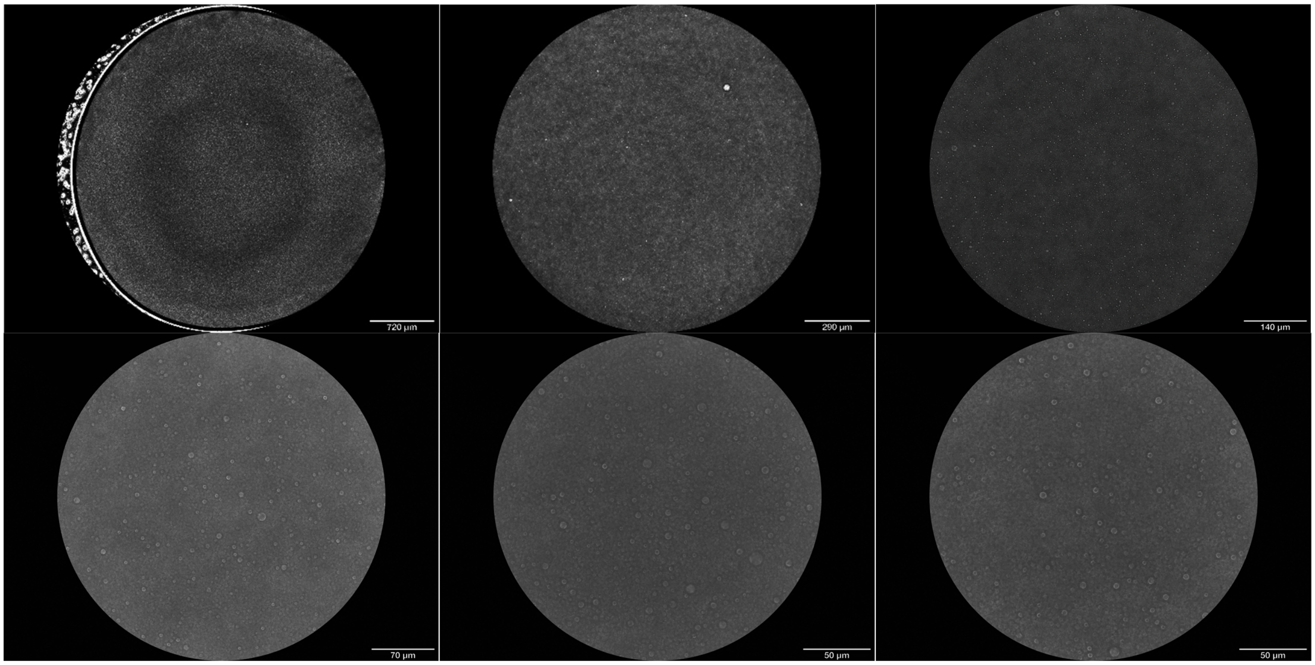

Supplementary Figure S217. Brightfield microscopy imaging of depsipeptides resulting from dry-down reactions of Phenyllactic acid and γ-aminobutyric acid. Microscopy of sample NA5- Phenyllactic acid and γ-aminobutyric acid, resuspended in 80% deionized water and 20% acetonitrile (v/v), at five different magnifications - x4, x10, x20, x40 and x60.

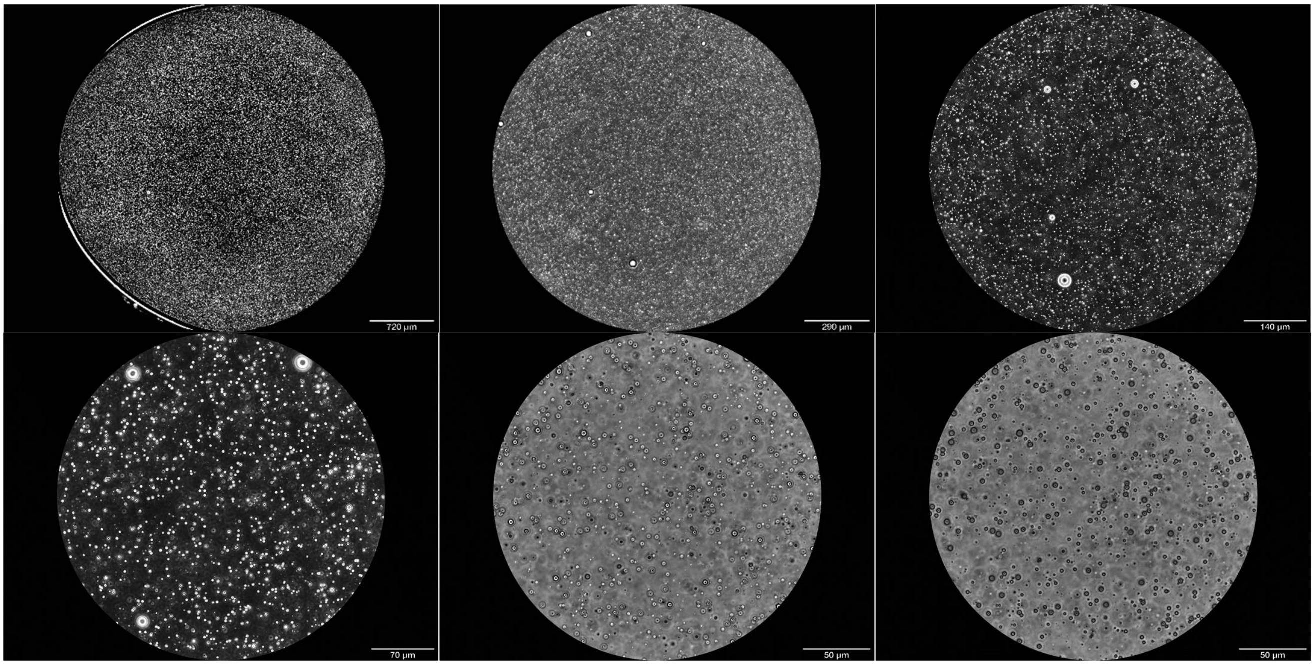

Supplementary Figure S218. Brightfield microscopy imaging of depsipeptides resulting from dry-down reactions of Phenyllactic acid and γ-aminopentanoic acid. Microscopy of sample NA6- Phenyllactic acid and γ-aminopentanoic acid, resuspended in 80% deionized water and 20% acetonitrile (v/v), at five different magnifications - x4, x10, x20, x40 and x60.

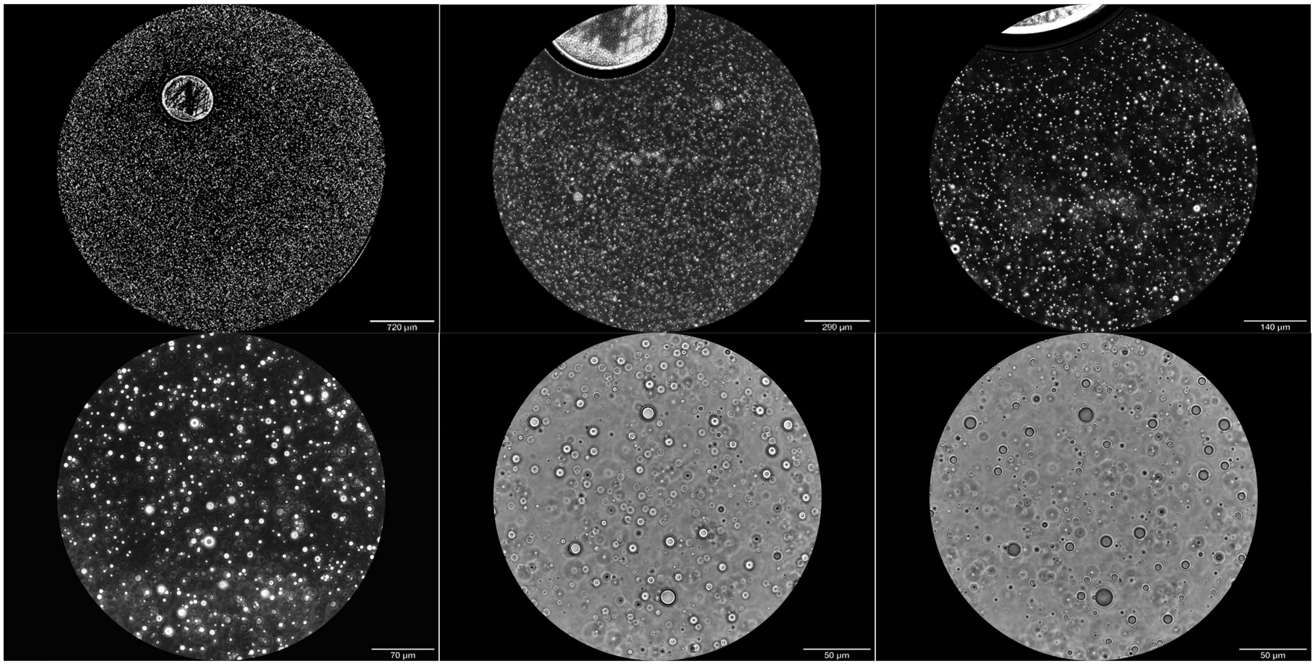

Supplementary Figure S219. Brightfield microscopy imaging of depsipeptides resulting from dry-down reactions of Leucic acid and Glycine. Microscopy of sample NA7- Leucic acid and Glycine, resuspended in 80% deionized water and 20% acetonitrile (v/v), at five different magnifications - x4, x10, x20, x40 and x60.

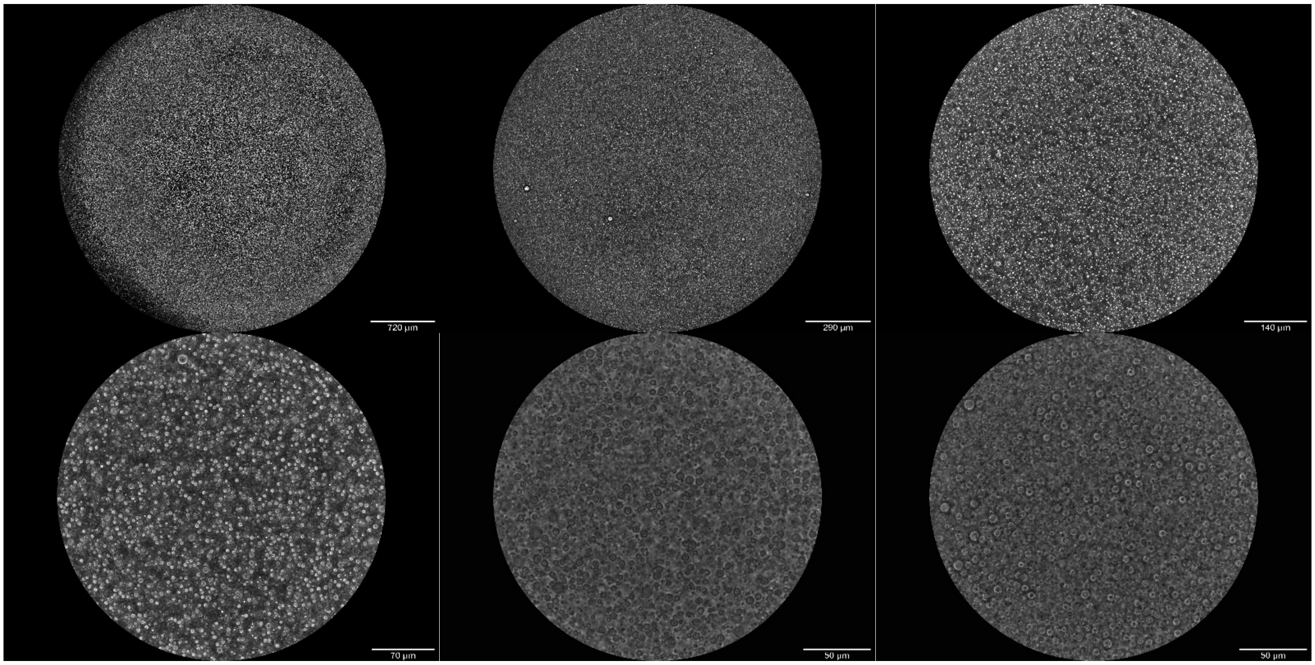

Supplementary Figure S220. Brightfield microscopy imaging of depsipeptides resulting from dry-down reactions of Leucic acid and L-alanine. Microscopy of sample NA8- Leucic acid and L-alanine, resuspended in 80% deionized water and 20% acetonitrile (v/v), at five different magnifications - x4, x10, x20, x40 and x60.

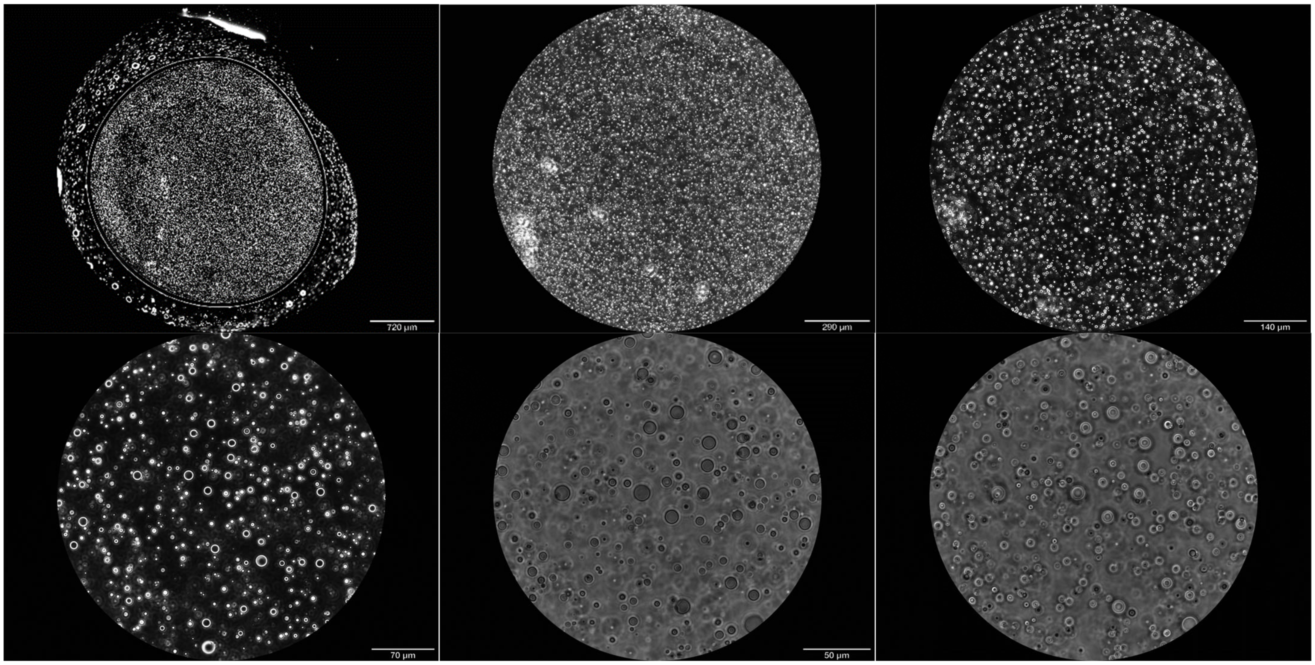

Supplementary Figure S221. Brightfield microscopy imaging of depsipeptides resulting from dry-down reactions of Leucic acid and β-alanine. Microscopy of sample NA9- Leucic acid and β-alanine, resuspended in 80% deionized water and 20% acetonitrile (v/v), at five different magnifications - x4, x10, x20, x40 and x60.

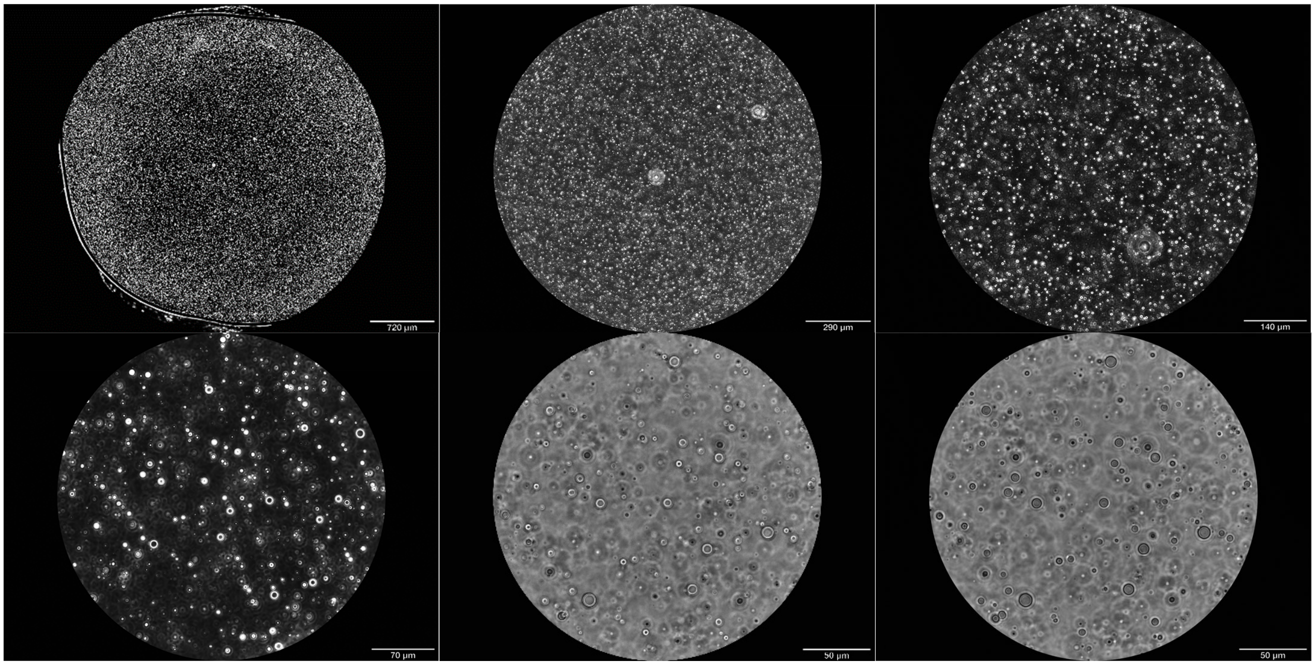

Supplementary Figure S222. Brightfield microscopy imaging of depsipeptides resulting from dry-down reactions of Leucic acid and β-aminobutyric acid. Microscopy of sample NA10- Leucic acid and β-aminobutyric acid, resuspended in 80% deionized water and 20% acetonitrile (v/v), at five different magnifications - x4, x10, x20, x40 and x60.

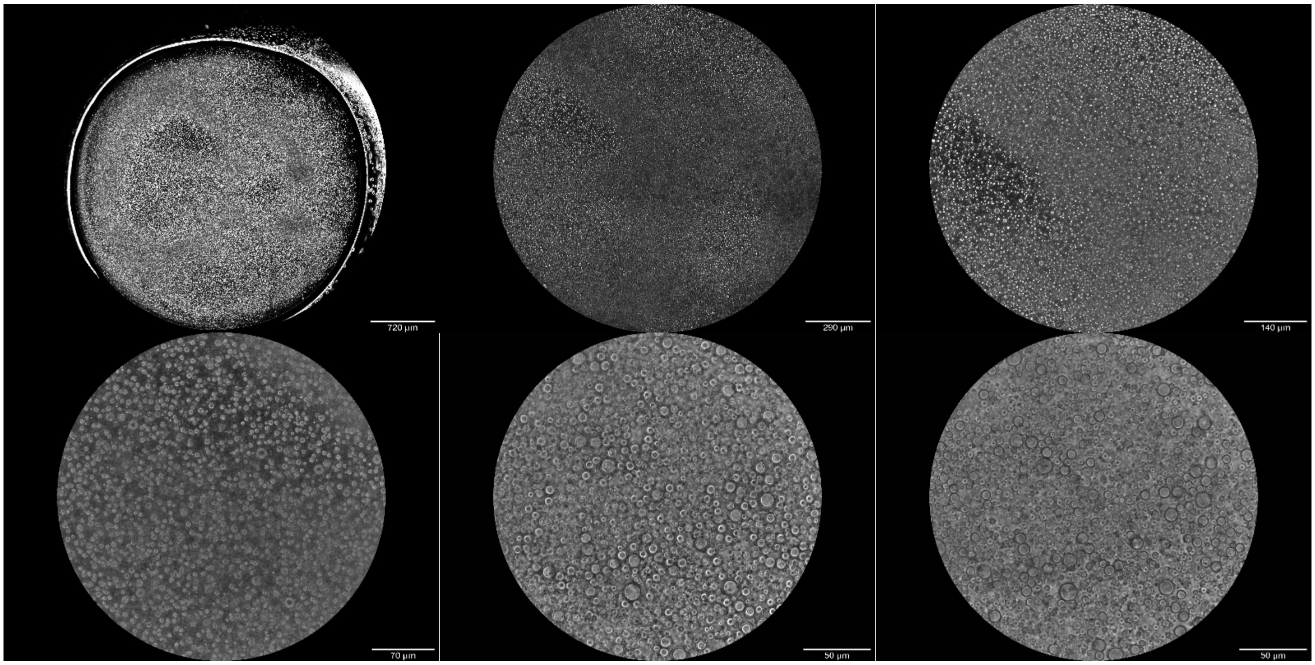

Supplementary Figure S223. Brightfield microscopy imaging of depsipeptides resulting from dry-down reactions of Leucic acid and γ-aminobutyric acid. Microscopy of sample NA11- Leucic acid and γ-aminobutyric acid, resuspended in 80% deionized water and 20% acetonitrile (v/v), at five different magnifications - x4, x10, x20, x40 and x60.

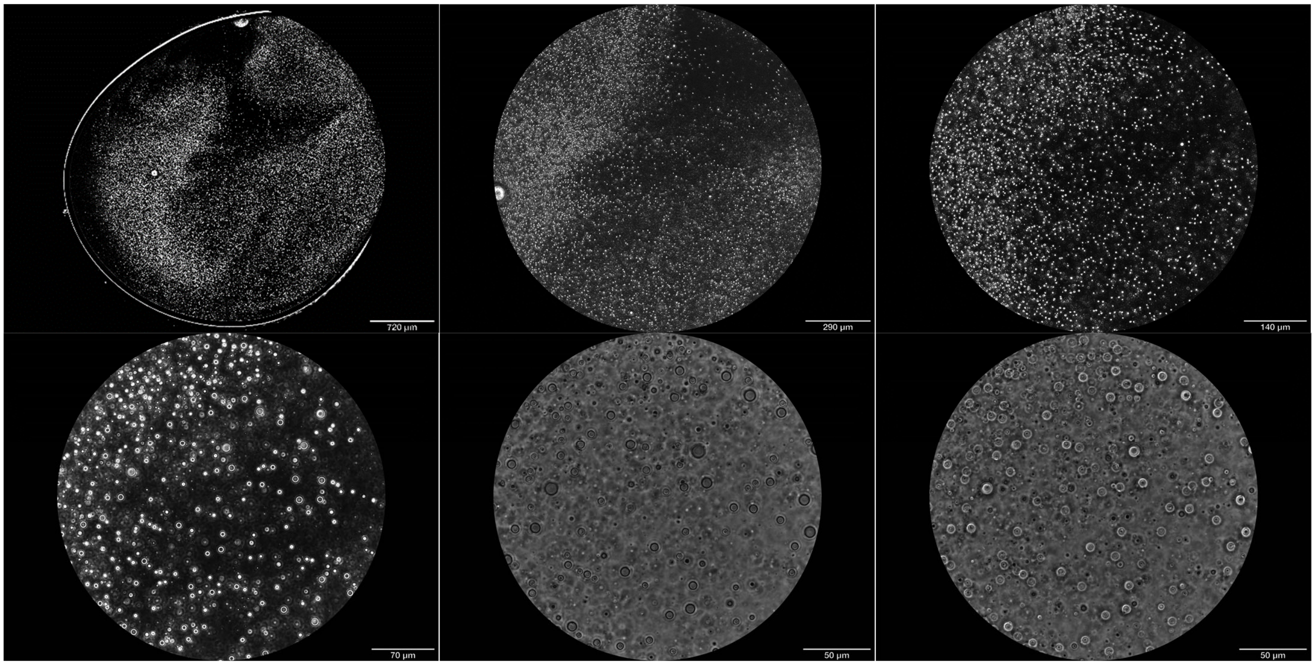

Supplementary Figure S224. Brightfield microscopy imaging of depsipeptides resulting from dry-down reactions of Leucic acid and γ-aminopentanoic acid. Microscopy of sample NA12- Leucic acid and γ-aminopentanoic acid, resuspended in 80% deionized water and 20% acetonitrile (v/v), at five different magnifications - x4, x10, x20, x40 and x60.

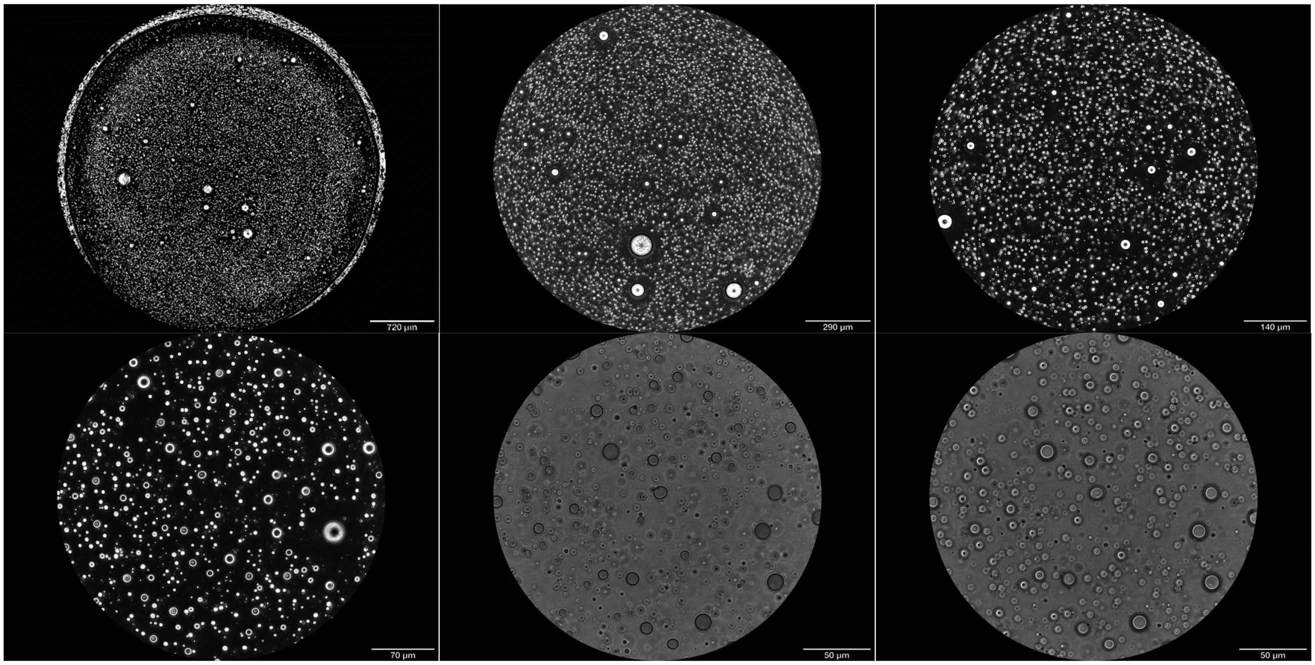

Supplementary Figure S225. Brightfield microscopy imaging of depsipeptides resulting from dry-down reactions of β-Phenyllactic acid and Glycine. Microscopy of sample NA13- β-Phenyllactic acid and Glycine, resuspended in 80% deionized water and 20% acetonitrile (v/v), at five different magnifications - x4, x10, x20, x40 and x60.

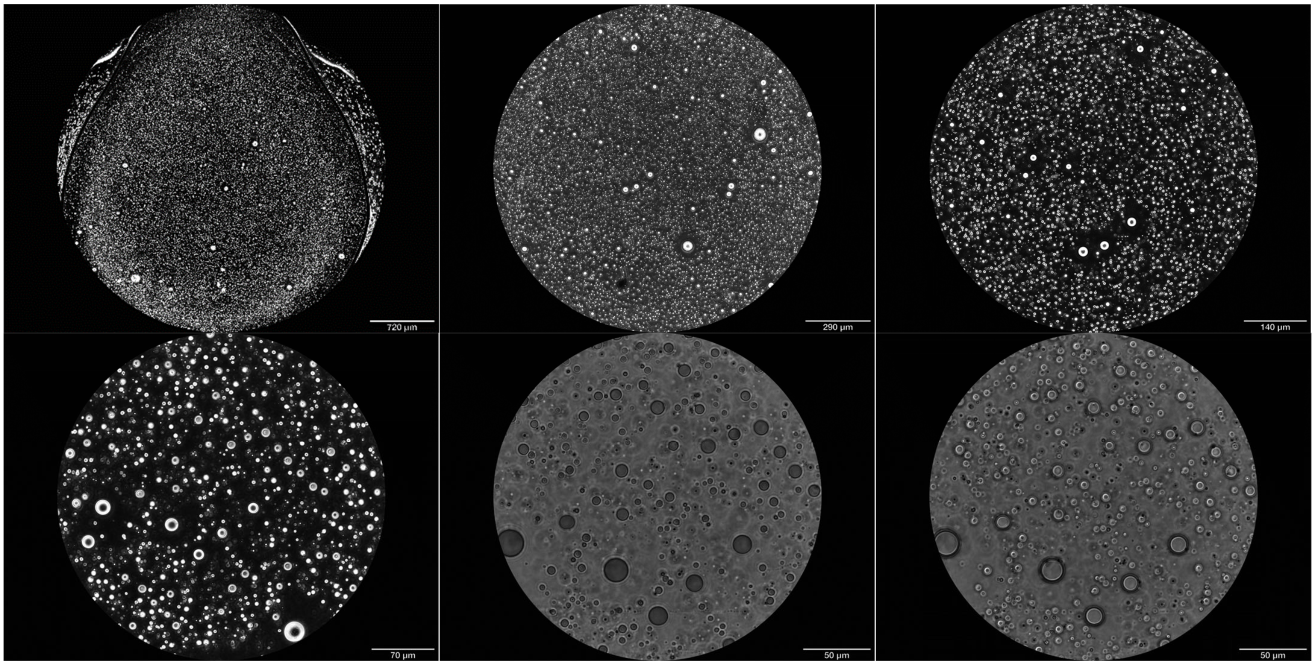

Supplementary Figure S226. Brightfield microscopy imaging of depsipeptides resulting from dry-down reactions of β-Phenyllactic acid and L-alanine. Microscopy of sample NA14- β-Phenyllactic acid and L-alanine, resuspended in 80% deionized water and 20% acetonitrile (v/v), at five different magnifications - x4, x10, x20, x40 and x60.

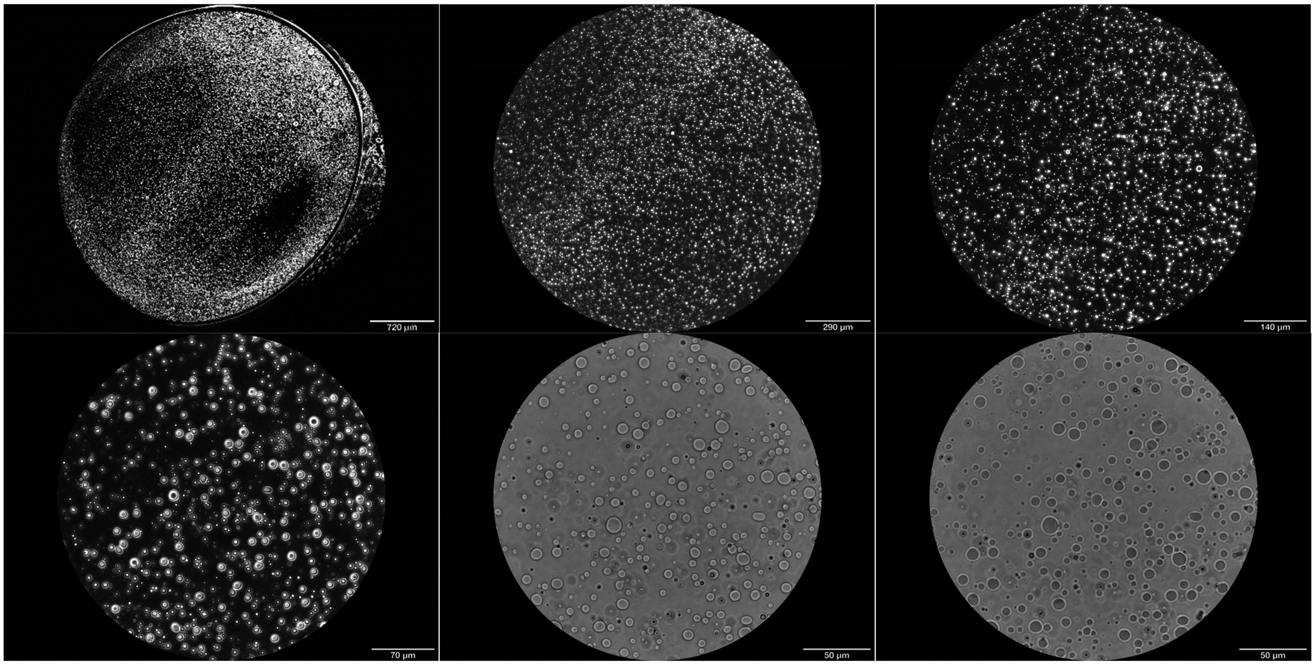

Supplementary Figure S227. Brightfield microscopy imaging of depsipeptides resulting from dry-down reactions of β-Phenyllactic acid and β-alanine. Microscopy of sample NA15- β-Phenyllactic acid and β-alanine, resuspended in 80% deionized water and 20% acetonitrile (v/v), at five different magnifications - x4, x10, x20, x40 and x60.

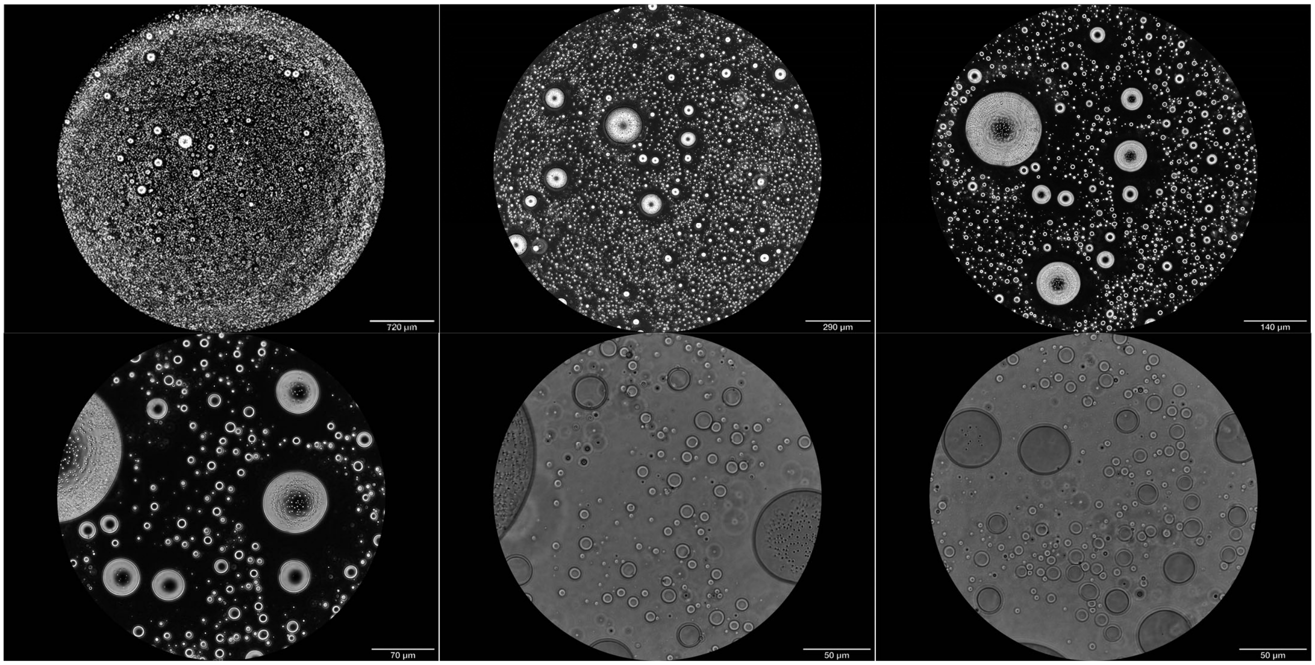

Supplementary Figure S228. Brightfield microscopy imaging of depsipeptides resulting from dry-down reactions of β-Phenyllactic acid and β-aminobutyric acid. Microscopy of sample NA16- β-Phenyllactic acid and β-aminobutyric acid, resuspended in 80% deionized water and 20% acetonitrile (v/v), at five different magnifications - x4, x10, x20, x40 and x60.

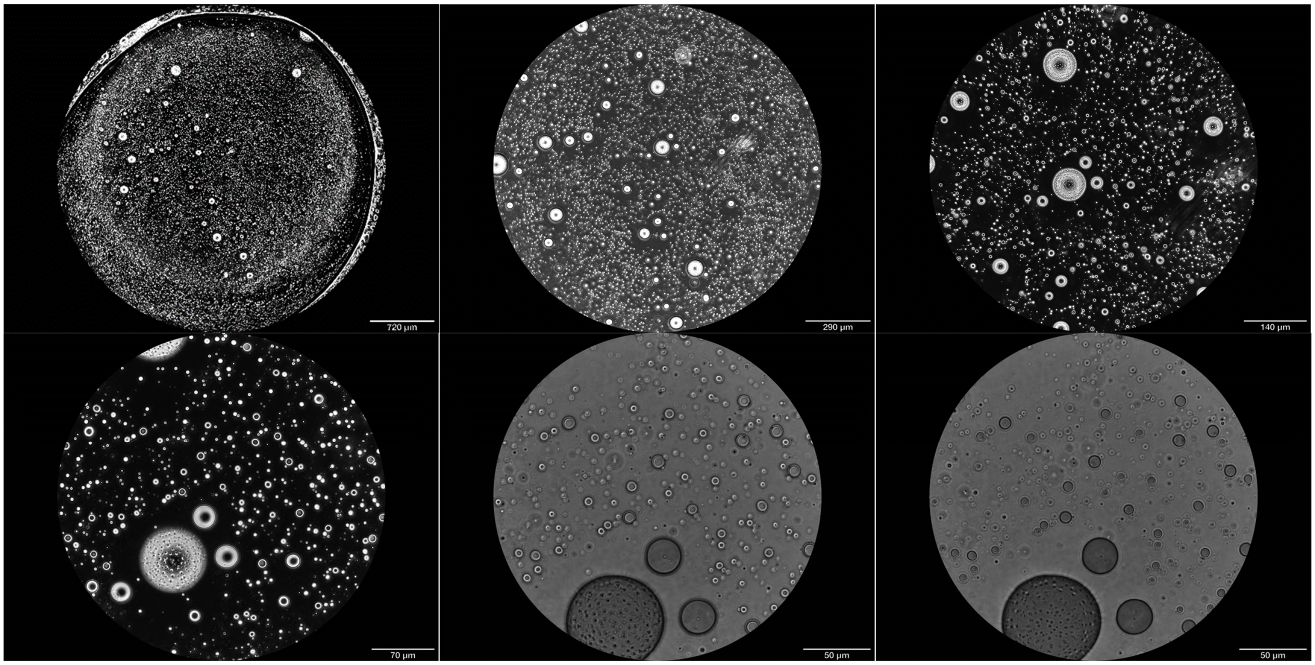

Supplementary Figure S229. Brightfield microscopy imaging of depsipeptides resulting from dry-down reactions of β-Phenyllactic acid and γ-aminobutyric acid. Microscopy of sample NA17- β-Phenyllactic acid and γ-aminobutyric acid, resuspended in 80% deionized water and 20% acetonitrile (v/v), at five different magnifications - x4, x10, x20, x40 and x60.

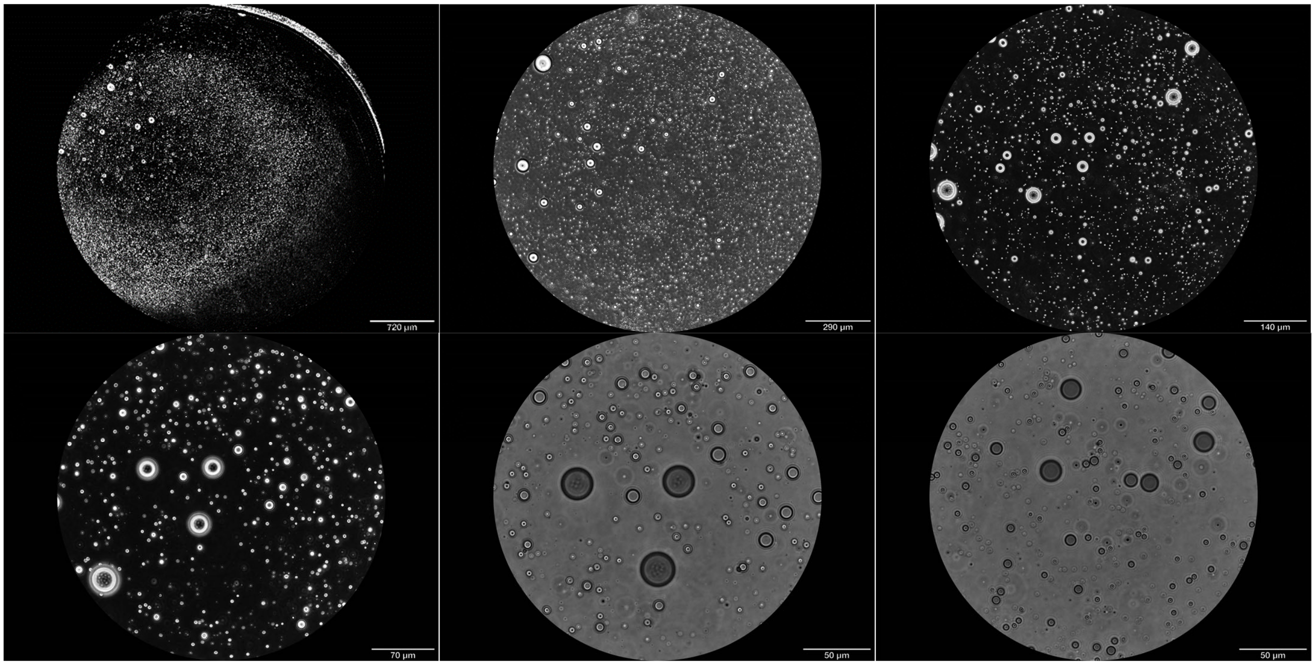

Supplementary Figure S230. Brightfield microscopy imaging of depsipeptides resulting from dry-down reactions of β-Phenyllactic acid and γ-aminopentanoic acid. Microscopy of sample NA18- β-Phenyllactic acid and γ-aminopentanoic acid, resuspended in 80% deionized water and 20% acetonitrile (v/v), at five different magnifications - x4, x10, x20, x40 and x60.

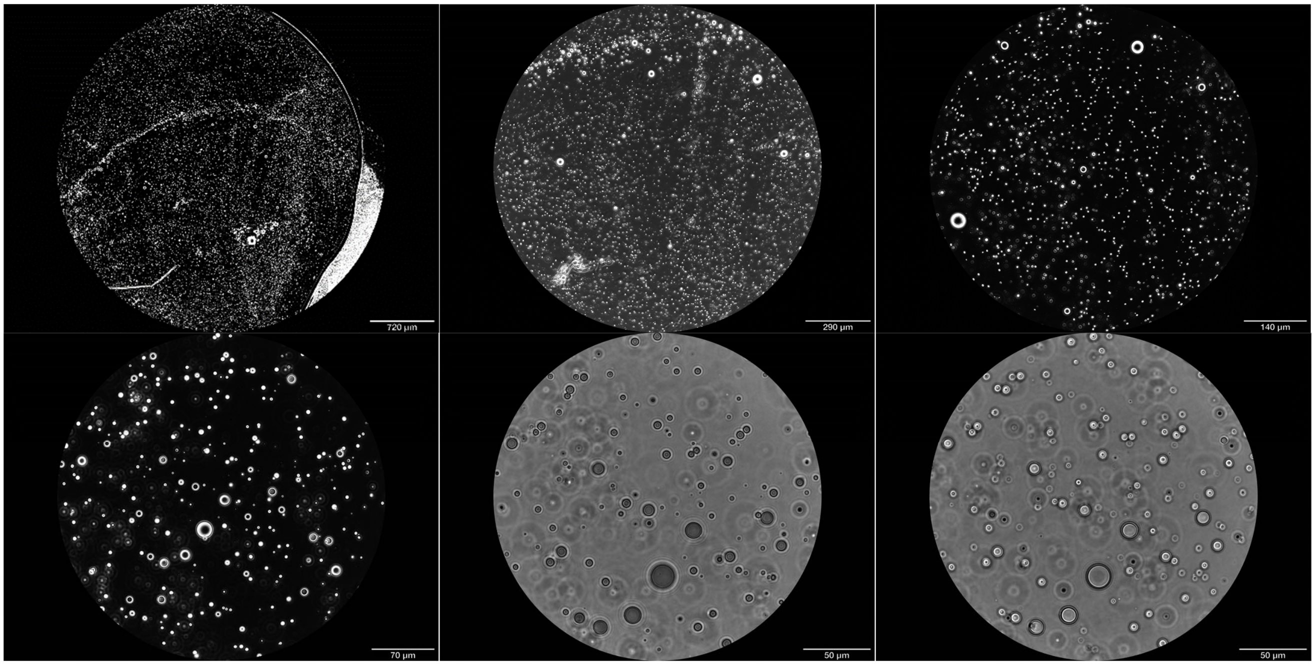

Supplementary Figure S231. Brightfield microscopy imaging of depsipeptides resulting from dry-down reactions of β-Leucic acid and Glycine. Microscopy of sample NA19- β-Leucic acid and Glycine, resuspended in 80% deionized water and 20% acetonitrile (v/v), at five different magnifications - x4, x10, x20, x40 and x60.

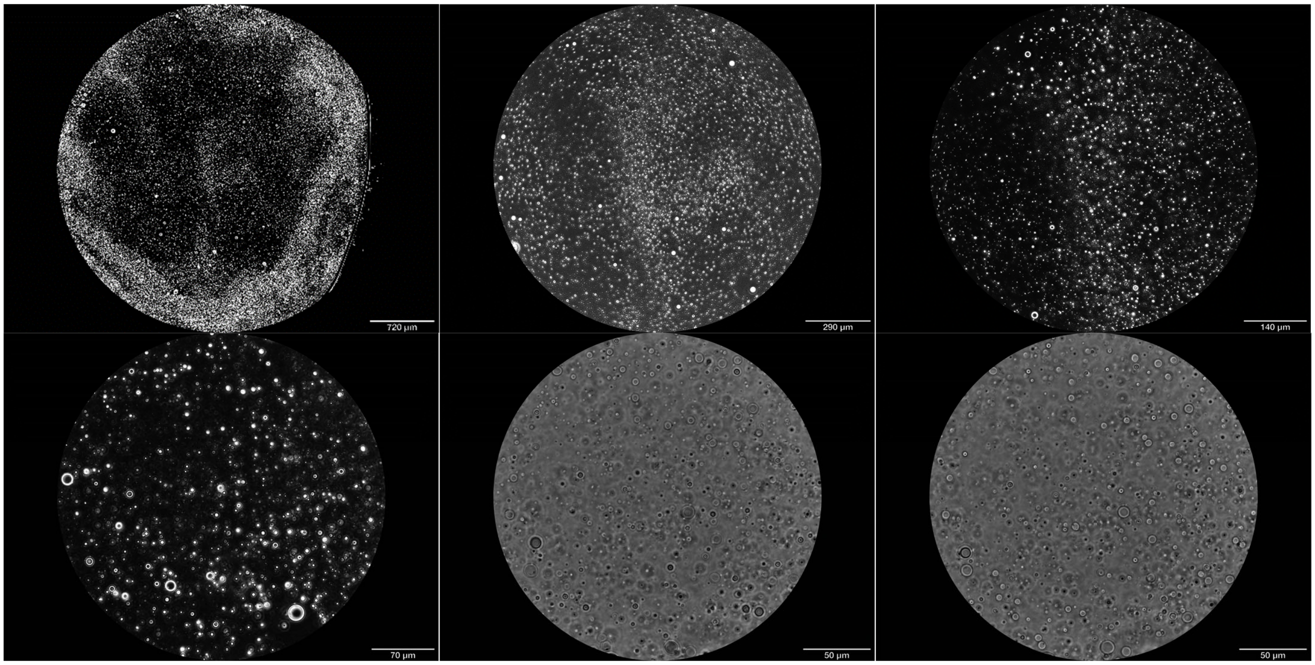

Supplementary Figure S232. Brightfield microscopy imaging of depsipeptides resulting from dry-down reactions of β-Leucic acid and L-alanine. Microscopy of sample NA20- β-Leucic acid and L-alanine, resuspended in 80% deionized water and 20% acetonitrile (v/v), at five different magnifications - x4, x10, x20, x40 and x60.

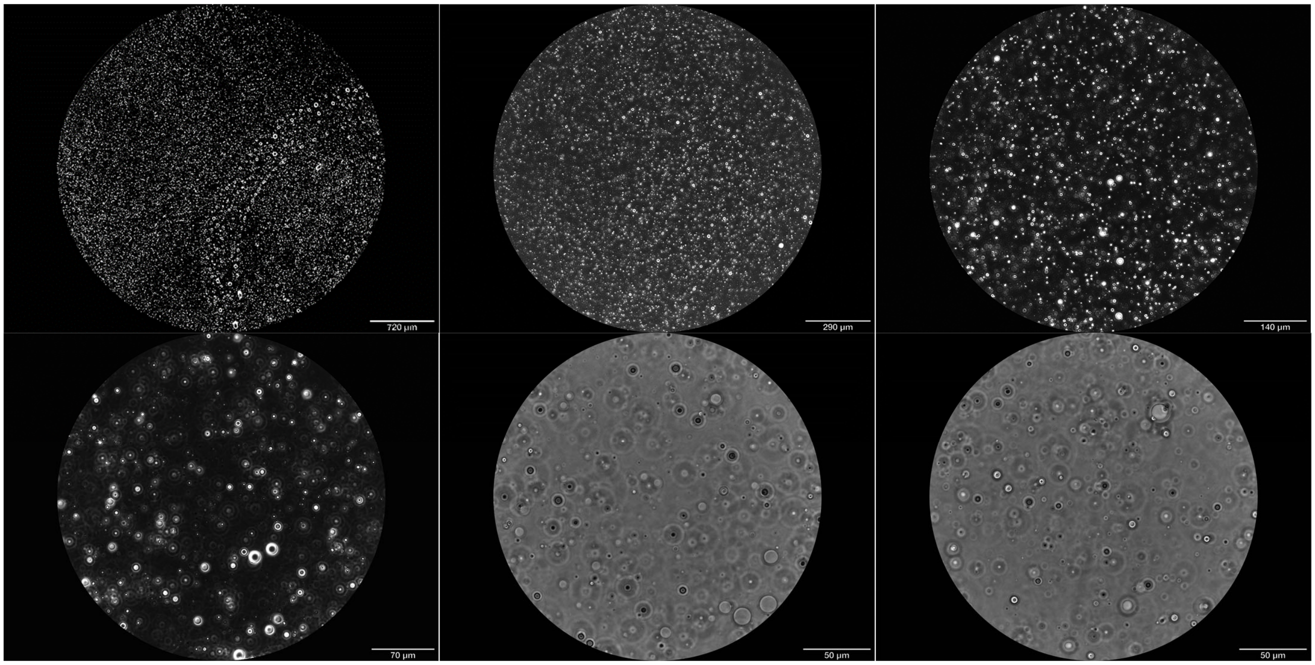

Supplementary Figure S233. Brightfield microscopy imaging of depsipeptides resulting from dry-down reactions of β-Leucic acid and β-alanine. Microscopy of sample NA21- β-Leucic acid and β-alanine, resuspended in 80% deionized water and 20% acetonitrile (v/v), at five different magnifications - x4, x10, x20, x40 and x60.

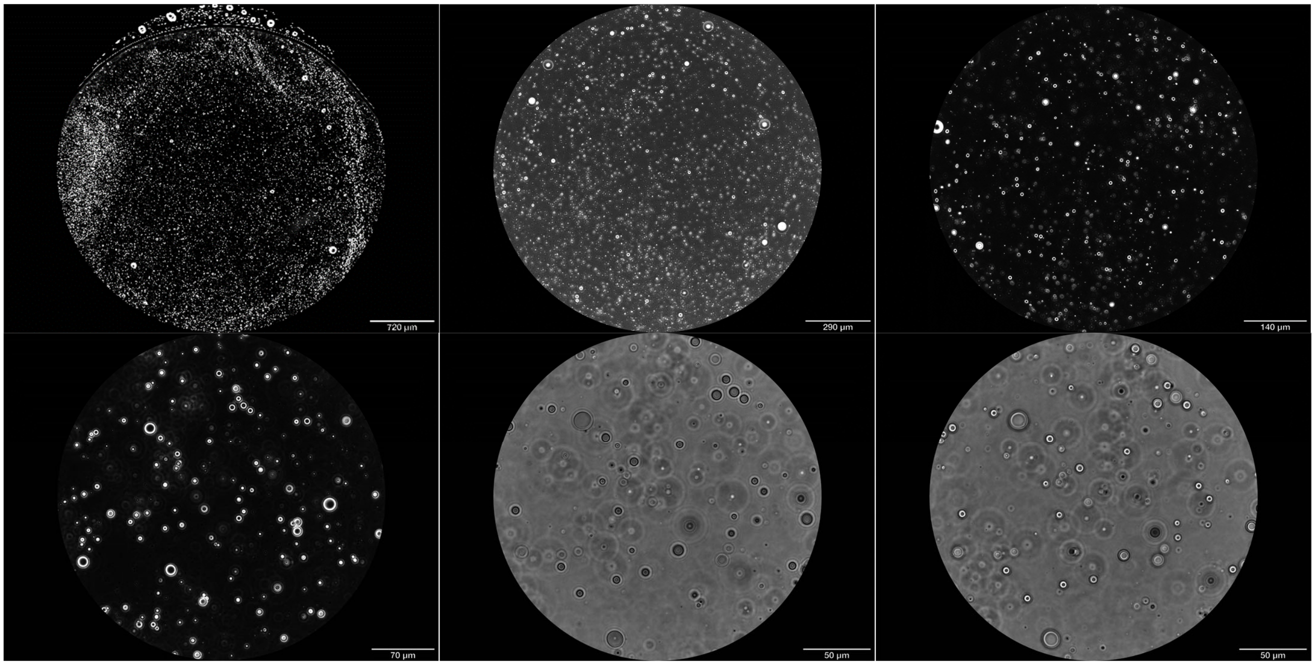

Supplementary Figure S234. Brightfield microscopy imaging of depsipeptides resulting from dry-down reactions of β-Leucic acid and β-aminobutyric acid. Microscopy of sample NA22- β-Leucic acid and β-aminobutyric acid, resuspended in 80% deionized water and 20% acetonitrile (v/v), at five different magnifications - x4, x10, x20, x40 and x60.

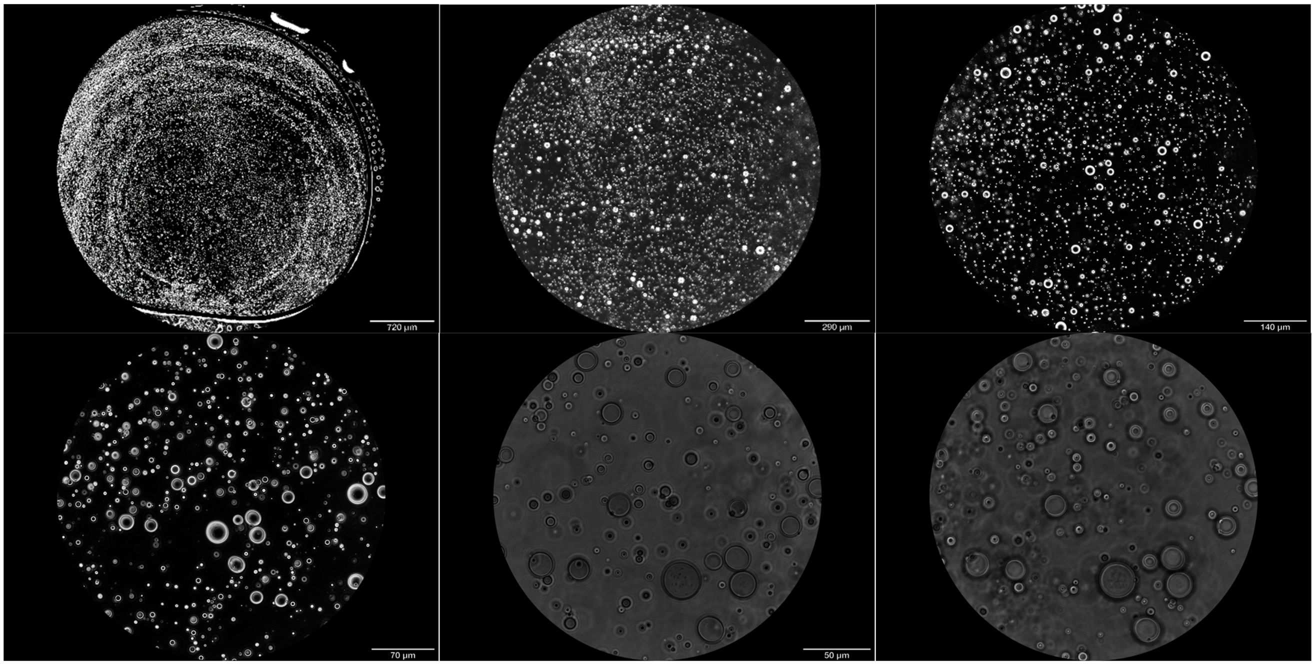

Supplementary Figure S235. Brightfield microscopy imaging of depsipeptides resulting from dry-down reactions of β-Leucic acid and γ-aminobutyric acid. Microscopy of sample NA23- β-Leucic acid and γ-aminobutyric acid, resuspended in 80% deionized water and 20% acetonitrile (v/v), at five different magnifications - x4, x10, x20, x40 and x60.

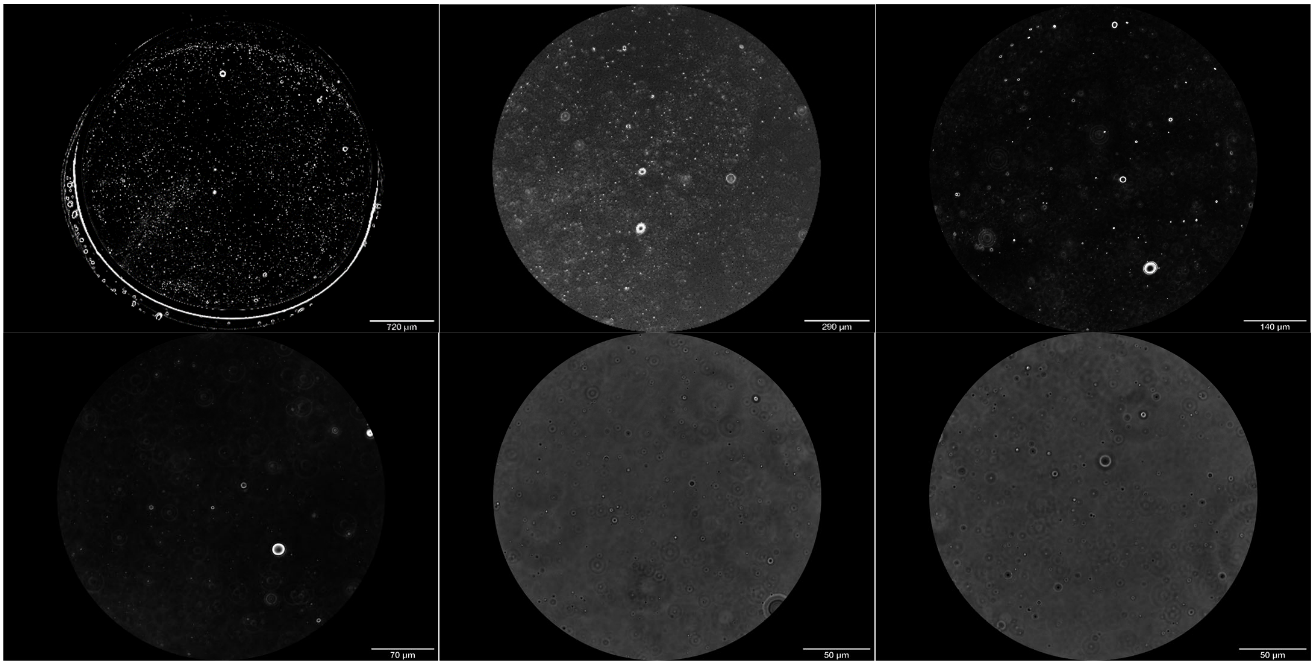

Supplementary Figure S236. Brightfield microscopy imaging of depsipeptides resulting from dry-down reactions of β-Leucic acid and γ-aminopentanoic acid. Microscopy of sample NA24- β-Leucic acid and γ-aminopentanoic acid, resuspended in 80% deionized water and 20% acetonitrile (v/v), at five different magnifications - x4, x10, x20, x40 and x60.

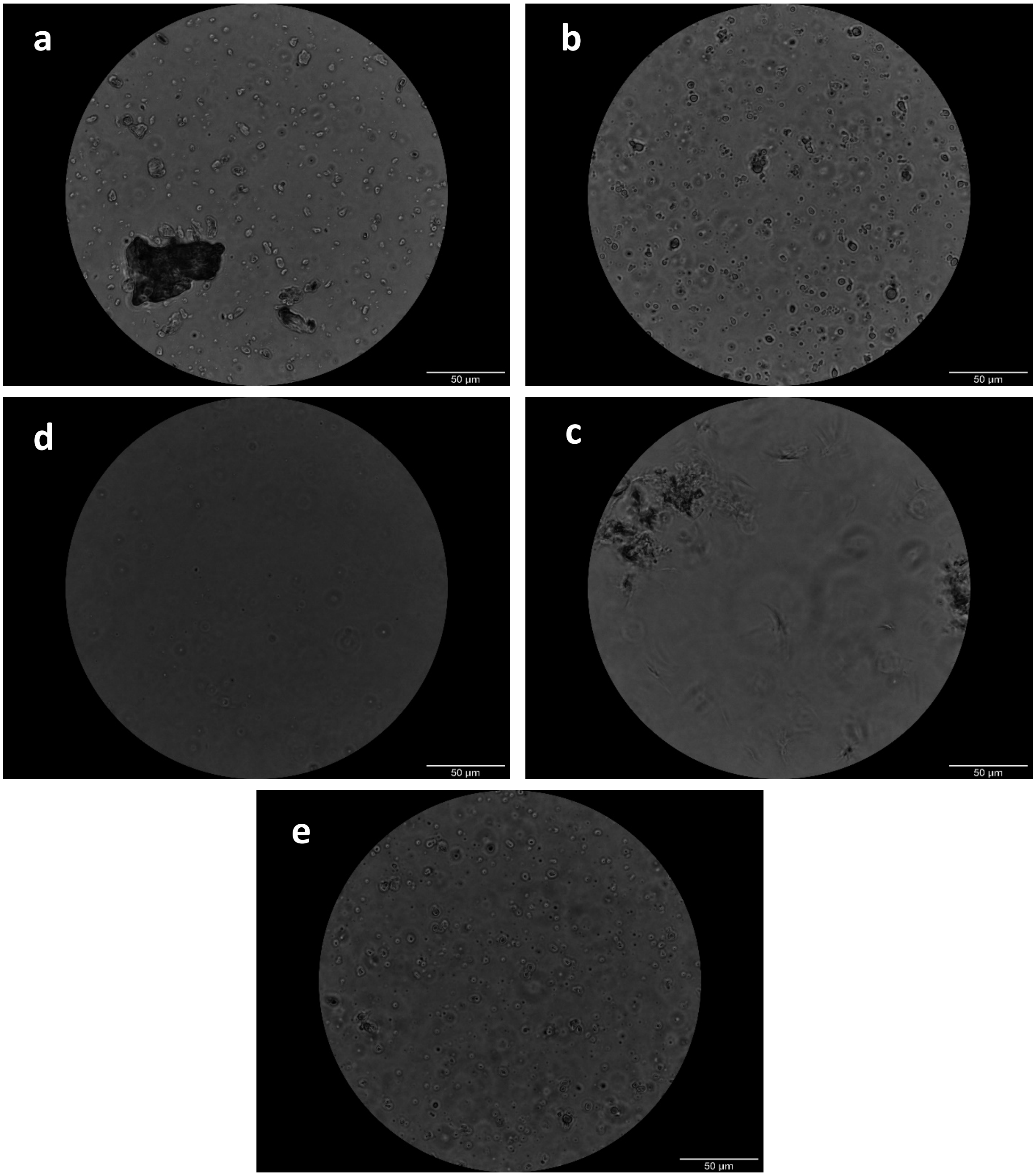

Supplementary Figure S237. Brightfield microscopy imaging of polyesters resulting from dry-down reactions of hydroxy acid from the first batch. **a)** glycolic acid, **b)** L-lactic acid, **c)** 3-hydroxybutyric acid, **d)** hydroxypentanoic acid, **e)** DL-lactic acid. All were resuspended in a solution of 80% deionized water and 20% acetonitrile (v/v).

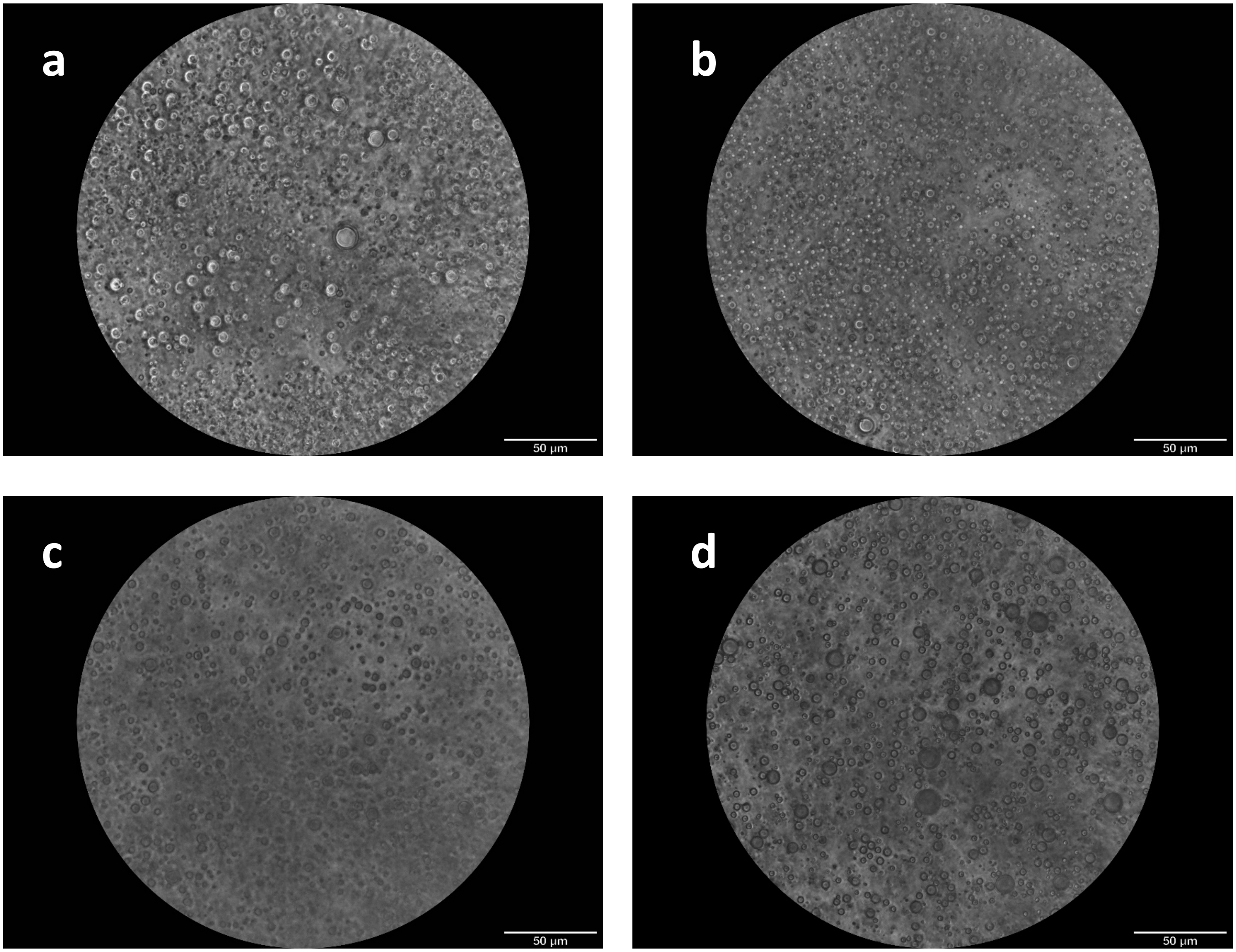

Supplementary Figure S238. Brightfield microscopy imaging of polyesters resulting from dry-down reactions of hydroxy acid from the second batch.
a) β-Leucic acid, **b)** β-Phenyllactic acid, **c)** α-Leucic acid, **d)** α-Phenyllactic acid. All were resuspended in a solution of 80% deionized water and 20% acetonitrile (v/v).

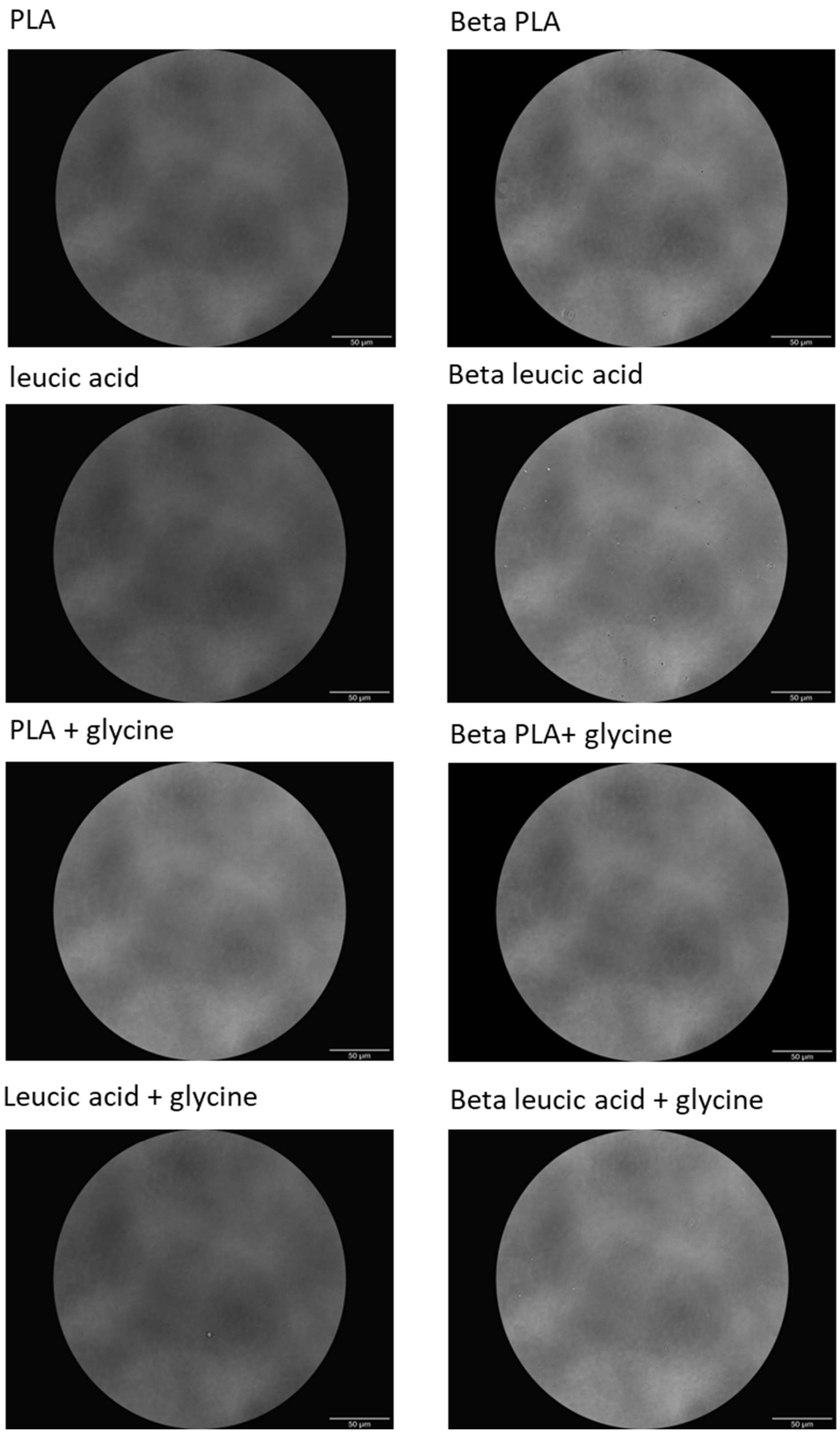

Supplementary Figure S239. Brightfield microscopy imaging of fresh hydroxy acid control samples. **a)** β-Leucic acid, **b)** β-Phenyllactic acid, **c)** α-Leucic acid, **d)** α-Phenyllactic acid, alone and with glycine. Samples were resuspended in a solution of 80% deionized water and 20% acetonitrile (v/v).

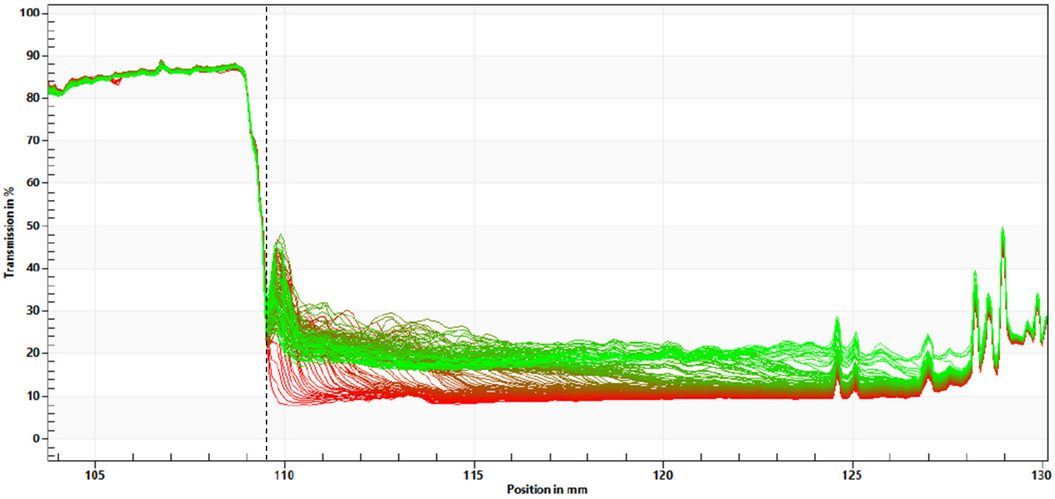

Supplementary Figure S240. LUMiSizer® transmission profile of α-Phenyllactic acid dry down products. Transmission profiles obtained for α-pla dry-down product following redispersion in water:ACN 4:1. 150 profiles were recorded every 5 seconds at 865nm. Each line in the profile represents the NIR light transmission (y-axis) at a single time point and within a range of positions along the cell as indicated by x-axis. Sample's meniscus is represented by the vertical line. High transmission values correspond with low turbidity while lower values indicate high turbidity. The color of the lines indicates the time point at which the profile was recorded. Red lines represent earlier stages of the measurements while green lines represent later time points. As indicated by the transmission profile, the sample initially exhibited low transmission which only mildly increased over the course of the measurement suggesting that the sample underwent partial phase separation.

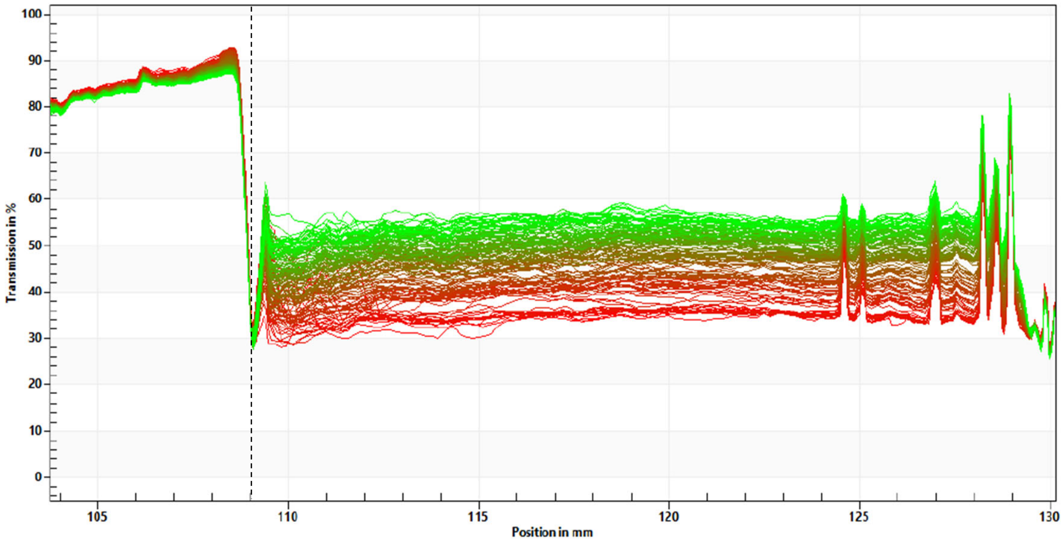

Supplementary Figure S241. LUMiSizer® transmission profile of β-Phenyllactic acid dry down products. Transmission profiles obtained for β-pla dry-down product following redispersion in water:ACN 4:1. 150 profiles were recorded every 5 seconds at 865nm. Each line in the profile represents the NIR light transmission (y-axis) at a single time point and within a range of positions along the cell as indicated by x-axis. Sample's meniscus is represented by the vertical line. High transmission values correspond with low turbidity while lower values indicate high turbidity. The color of the lines indicates the time point at which the profile was recorded. Red lines represent earlier stages of the measurements while green lines represent later time points. As indicated by the transmission profile, the sample initially exhibited low transmission which gradually increased over the course of the measurement suggesting that the sample underwent phase separation.

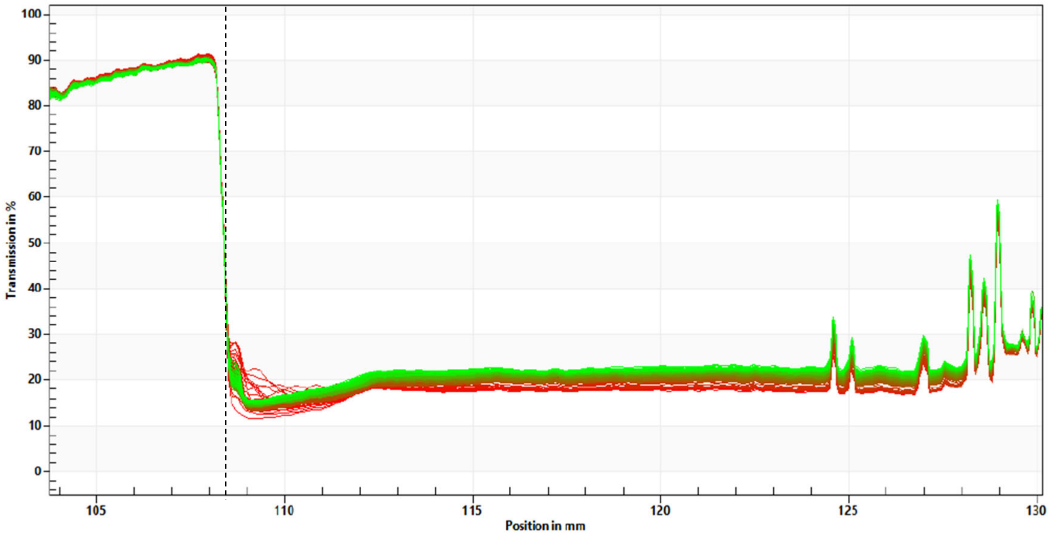

Supplementary Figure S242. LUMiSizer® transmission profile of α-Leucic acid dry down products. Transmission profiles obtained for α-leu dry-down product following redispersion in water:ACN 4:1. 150 profiles were recorded every 5 seconds at 865nm. Each line in the profile represents the NIR light transmission (y-axis) at a single time point and within a range of positions along the cell as indicated by x-axis. Sample's meniscus is represented by the vertical line. High transmission values correspond with low turbidity while lower values indicate high turbidity. The color of the lines indicates the time point at which the profile was recorded. Red lines represent earlier stages of the measurements while green lines represent later time points. As indicated by the transmission profile, the sample initially exhibited low transmission. At the end of the measurement only minor changes in the transmission profile were observed suggesting that the sample remained relatively physically stable after being subjected to the centripetal forces.

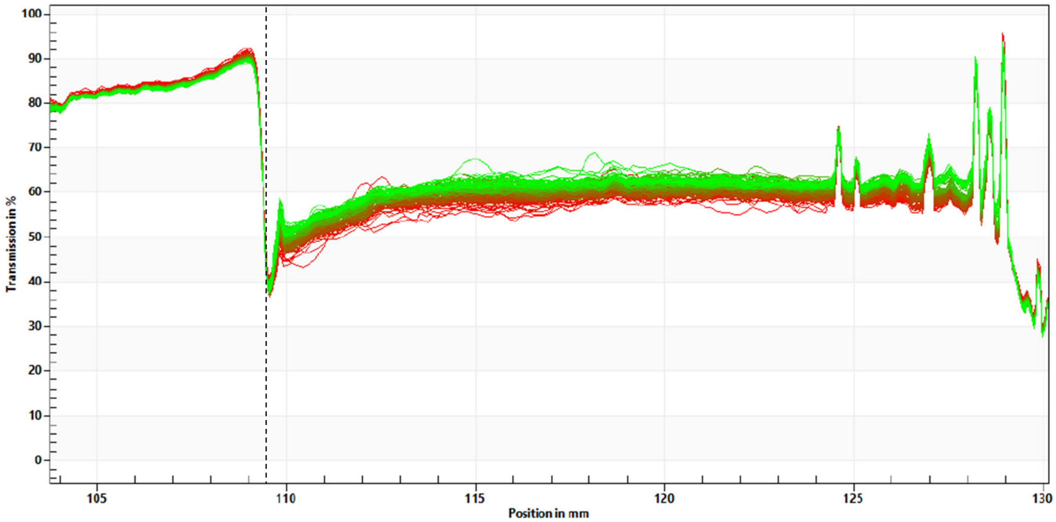

Supplementary Figure S243. LUMiSizer® transmission profile of β-Leucic acid dry down products. Transmission profiles obtained for β-leu dry-down product following redispersion in water:ACN 4:1. 150 profiles were recorded every 5 seconds at 865nm. Each line in the profile represents the NIR light transmission (y-axis) at a single time point and within a range of positions along the cell as indicated by x-axis. Sample's meniscus is represented by the vertical line. High transmission values correspond with low turbidity while lower values indicate high turbidity. The color of the lines indicates the time point at which the profile was recorded. Red lines represent earlier stages of the measurements while green lines represent later time points. As indicated by the transmission profile, the sample initially exhibited moderate transmission which slightly increased over the course of the measurement suggesting that the sample exhibited only small physical change.

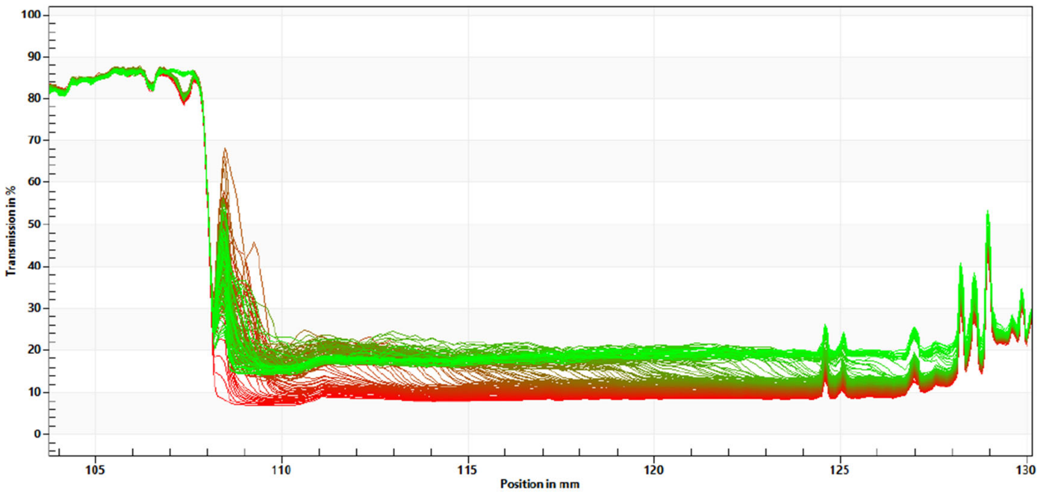

Supplementary Figure S244. LUMiSizer® transmission profile of α-Phenyllactic acid + Glycine dry down products. Transmission profiles obtained for α-pla and Gly dry-down product following redispersion in water:ACN 4:1. 150 profiles were recorded every 5 seconds at 865nm. Each line in the profile represents the NIR light transmission (y-axis) at a single time point and within a range of positions along the cell as indicated by x axis. Sample's meniscus is represented by the vertical dashed line. High transmission values correspond with low turbidity while lower values indicate high turbidity. The color of the lines indicates the time point at which the profile was recorded. Red lines represent earlier stages of the measurements while green lines represent later time points. As indicated by the transmission profile, the sample initially exhibited low transmission which gradually increased over the course of the measurement suggesting that the sample underwent partial phase separation.

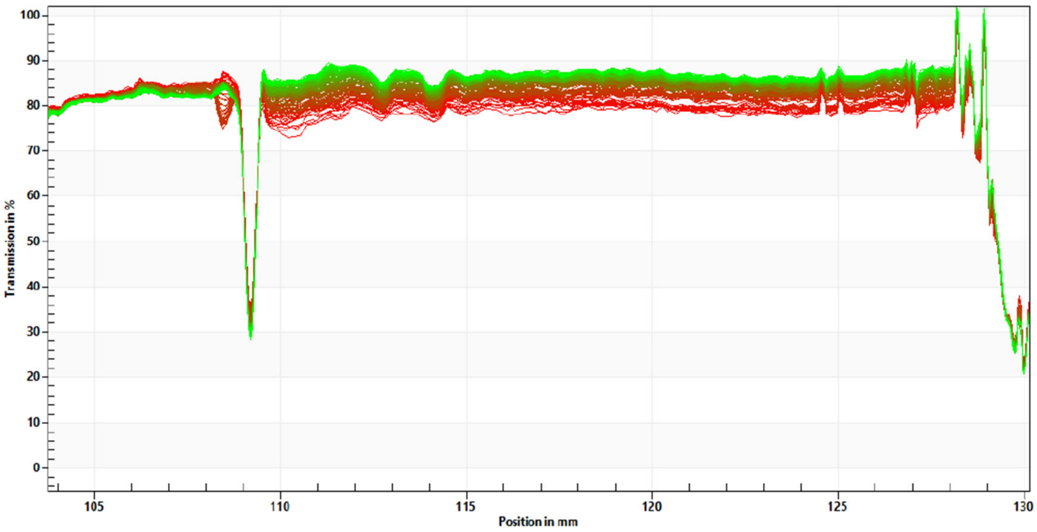

Supplementary Figure S245. LUMiSizer® transmission profile of β-Phenyllactic acid + Glycine dry down products. Transmission profiles obtained for β-pla and Gly dry-down product following redispersion in water:ACN 4:1. 150 profiles were recorded every 5 seconds at 865nm. Each line in the profile represents the NIR light transmission (y-axis) at a single time point and within a range of positions along the cell as indicated by x-axis. Sample's meniscus is represented by the vertical line. High transmission values correspond with low turbidity while lower values indicate high turbidity. The color of the lines indicates the time point at which the profile was recorded. Red lines represent earlier stages of the measurements while green lines represent later time points. As indicated by the transmission profile, the sample initially exhibited high transmission which gradually increased over the course of the measurement reaching almost maximal transmission. This indicates that the sample was closed to undergo complete phase separation.

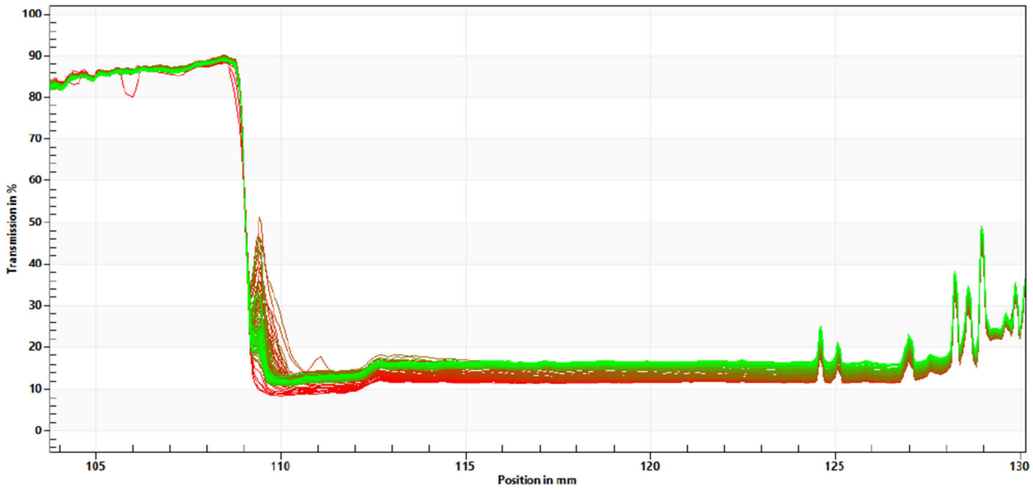

Supplementary Figure S246. LUMiSizer® transmission profile of α-Leucic acid + Glycine dry down products. Transmission profiles obtained for α-leu and Gly dry-down product following redispersion in water:ACN 4:1. 150 profiles were recorded every 5 seconds at 865nm. Each line in the profile represents the NIR light transmission (y-axis) at a single time point and within a range of positions along the cell as indicated by x axis. Sample's meniscus is represented by the vertical dashed line. High transmission values correspond with low turbidity while lower values indicate high turbidity. The color of the lines indicates the time point at which the profile was recorded. Red lines represent earlier stages of the measurements while green lines represent later time points. As indicated by the transmission profile, the sample initially exhibited low transmission which only slightly increased over the course of the measurement. This indicates that the sample was relatively physically stable under the tested conditions.

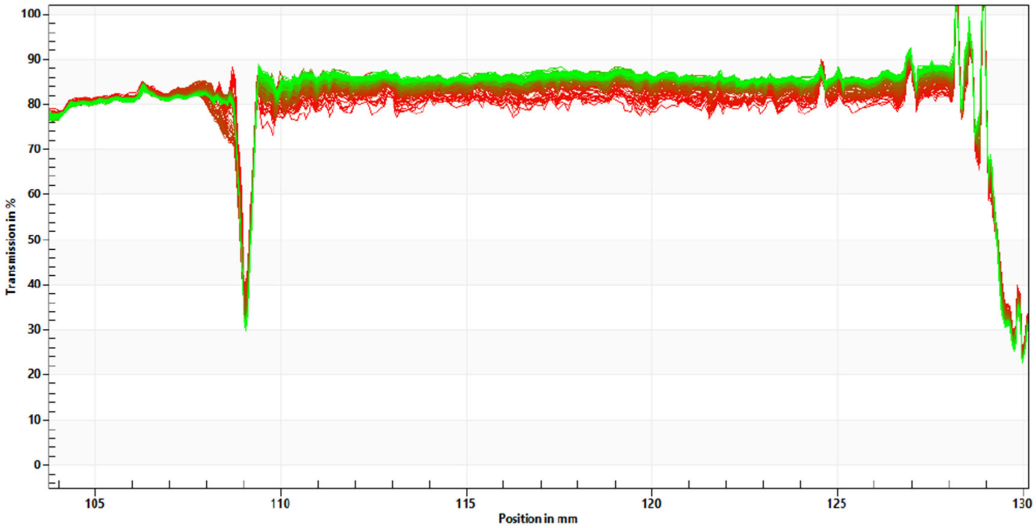

Supplementary Figure S247. LUMiSizer® transmission profile of β-Leucic acid + Glycine dry down products. Transmission profiles obtained for β-leu and Gly dry-down product following redispersion in water:ACN 4:1. 150 profiles were recorded every 5 seconds at 865nm. Each line in the profile represents the NIR light transmission (y-axis) at a single time point and within a range of positions along the cell as indicated by x-axis. Sample's meniscus is represented by the vertical line. High transmission values correspond with low turbidity while lower values indicate high turbidity. The color of the lines indicates the time point at which the profile was recorded. Red lines represent earlier stages of the measurements while green lines represent later time points. As indicated by the transmission profile, the sample initially exhibited high transmission which gradually increased over the course of the measurement reaching almost maximal transmission. This indicates that the sample was closed to undergo complete phase separation.
